# Reviewing the role of the environment in the talent development of a professional soccer club

**DOI:** 10.1371/journal.pone.0246823

**Published:** 2021-02-25

**Authors:** Vincent Gesbert, Fabienne Crettaz von Roten, Denis Hauw

**Affiliations:** Institute of Sport Sciences, University of Lausanne, Lausanne, Vaud, Switzerland; Jacobs University Bremen, GERMANY

## Abstract

This two-part study examined the perceptions of talented Swiss soccer players about their talent development environment. The first study presented the translation and validation of the Talent Development Environment Questionnaire (TDEQ) into French using a recommended methodology for translating and culturally adapting questionnaires. Two hundred and three Swiss athletes (M = 16.99 years old) responded to the 25 items of the TDEQ-5. One item was excluded due to low factor loadings, and the descriptive statistics showed that the re-specified TDEQ-5 instrument had acceptable global model fit according to the thresholds in the literature (χ2 (df = 17) = 484.62, p<0.001, CFI = 0.91, TLI = 0.90, RMSEA = 0.07, SRMR = 0.06). This adaptation is thus valid for assessing the effectiveness of talent development processes. For the second study, a holistic design was used to examine the perceptions of a set of players embedded in a top-level Swiss soccer academy (i.e., 64 elite soccer players from 14 to 18 years old) by using the TDEQ-5. The results showed some relative strengths (i.e., F1-Long-Term Focus for the M15 and M16 age-groups) and weaknesses (i.e., F2-Alignment of Expectations for the M17 and M18 age -groups and F3-Communication for M17). They also highlighted that the talent pathways of these Swiss soccer players could not be summarized by a single type of transition toward a professional team. Rather, there were context-specific requirements, such as the critical period between the M15-M16 and M17-M18 age-groups, suggesting that when the players first entered their TDE they experienced a set of affordances to develop and flourish, which thereafter were perceived as less rich and/or abundant. These results offer a starting point for optimizing talent pathways.

## Introduction

### The talent development process

Training future generations of professional soccer players is of considerable importance to professional clubs to ensure both talent for their top teams and financial profit [1–3]. Overall, two programs are key to this process: talent identification and talent development. Talent identification (TI) is the process of recognizing and selecting exceptionally talented soccer players in order to provide them opportunities to accelerate their development [4–6]. Many club teams invest substantial financial and human resources in identifying these high-potential players. Although most TI systems use the current junior performance as the main selection criterion, some studies have pointed out that these indicators do not significantly predict long-term success [7, 8]. For instance, the younger the players are when they enter a talent development center, the sooner they leave. This underlines the multidimensional and dynamical nature of talent. In other words, talented aspiring soccer players do not automatically translate into high-level performers, but rather they continuously develop through numerous interactions with the environment [9, 10]. This is referred to as talent development (TD) and it suggests that talented soccer players need to be prepared if they are to translate their potential into situated achievement through flourishing environments and opportunities for the most appropriate learning [11–13].

### The talent development environment as the most controllable dimension in TD

It is thus important to grasp the features of the talent development environment (TDE) because this environment is the most controllable dimension in the TD process [14, 15]. Although the literature on the role of the environment in athletes’ development is ample [16–20], it should be noted that the environment can encompass a very great number of factors, including birthplace, socioeconomic status, sporting policy, support from parents, family, siblings or peers, coaches and staff, and training/development programs. In the present study, the environment is defined as encompassing all coaching situations [14] and the players’ immediate surroundings where athletic, psychosocial and personal development is likely to occur [21]. It is widely acknowledged that aspiring athletes need to develop both key psycho-behavioral and coping skills and adequate social support along their career pathways if they are to interpret the inevitable challenges as positive growth experiences and deal with them effectively [22, 23]. If coaches neglect to design and structure coherent and appropriate experiences within their environments, aspiring athletes cannot be automatically transformed into world-class performers [24, 25].

The relationships with parents, siblings or peers at different stages of athletes’ development may also favor or impede their development. Lauer et al. [26] examined the positive and negative behaviors that shaped athletes’ development during the junior tennis years. By scrutinizing trends across developmental stages, they observed that parents generally built positive experience with their children in the early years but that more conflict emerged in the middle years. The middle years were characterized by increasing pressure on the athletes to improve, and many parents displayed controlling and even pushy behaviors regarding training and competition (e.g., emphasis on immediate results, lack of support, over-involvement, restricted social life). Taylor et al. [27] explored the impact of sibling interactions on the TD process and found that sibling rivalry provoked adaptive behaviors with outcomes that facilitated development (e.g., increased motivation to train harder).

Last, it seems intuitive that the development environments of some soccer academies would be more successful than others in helping their talented players to exploit and efficiently develop their potential for future expert performance [15, 17, 28]. From this perspective, understanding the player’s worldview in order to safeguard his or her wellbeing, creating a player-driven environment to promote self-responsibility (e.g., encouraging their ideas/feedback), and establishing explicit pathways to the senior level with a focus on long-term education and development are considered developmental catalysts that distinguish good and less good soccer academies [15, 29].

More broadly, previous research has sought to characterize key TDE factors for effectively nurturing gifted athletes over the long term [14, 19]. Four characteristics of an effective TDE emerged: (i) long-term aims and methods, (ii) coherent support and messages, (iii) emphasis on appropriate development (not early success or performance at a given instant), and (iv) individualized and ongoing development. These key features formed the foundation upon which the Talent Development Environment Questionnaire (TDEQ) was developed [30] The TDEQ enables practitioners and researchers to measure athletes’ perceptions of their experiences against criteria shown to be important for shaping, challenging and supporting the development of talent for future success.

### TDEQ as a resource for identifying areas for improvement

Previous research using the TDEQ across a variety of sports has identified the positive impact of the TDE on important player outcomes, such as the progression from academy to elite status [29], on achievement goals and life aspirations [31], and the development of mental toughness [32] and wellbeing [33]. It can also help prevent burnout [33, 34].

Within soccer, Ivarsson and colleagues [16] described how the TDE, as measured by the TDEQ, was connected to important and positive player outcomes in soccer (i.e., wellbeing and perceived stress). The perceptions of 195 young elite players in Swedish soccer academies regarding their environment, wellbeing and stress levels were collected. Statistical analysis revealed three classes of players with distinct perceptions of their TDE (high, moderate and poor quality). A high-quality TDE depicted through a long-term development focus, a well-established support network, and high-quality coaching and practice was linked with higher levels of wellbeing and lower initial levels of stress. In contrast, a low-quality TDE was characterized by player perceptions of no strong long-term focus, difficulties in communicating with the coach or deficiencies in their support network, and their coach not being involved in the goal-setting process. These outcomes were linked with lower levels of wellbeing. The results suggested the association between features in the TDE and the wellbeing of young elite players. Consequently, it is important for clubs to promote high-quality environments, as the association with higher levels of wellbeing is likely to increase the players’ opportunities to flourish, develop and demonstrate expertise. For this reason, it is useful and relevant to measure the strengths and weaknesses of environments using the TDEQ. In fact, this has been done twice in soccer contexts, with the findings highlighting important areas for development.

The first study to use the TDEQ in the soccer context examined the perceptions of 50 young elite soccer academy players (16–18 years old) about their TDE at a crucial stage in their developmental pathway [35]. Although these players’ perceptions revealed key strengths in (i) coaching practices (i.e., training plans, promotion of self-responsibility) and (ii) organizational and sports-related support areas (i.e., access to a variety of professionals), they also highlighted deficiencies in (i) understanding the athletes (i.e., taking an interest in the players’ lives outside of soccer, their wellbeing), (ii) ensuring contacts with more senior players (i.e., youth and senior team operations were not well-integrated), and (iii) key stakeholder relationships (i.e., coaches did not take sufficient time to talk with the parents about the child’s/player’s development). The authors offered evidence-based strategies to help address these concerns and improve the sports development pathways and practices, one recommendation being mentoring opportunities to enhance the links between junior and senior players.

The second study examined the perceptions of female soccer players in 14 UK-based female soccer TDEs [36] and identified three areas for improvement. The first area concerned the planning for soccer-specific development and career progression. In this regard, one of the most significant results was that developing players are sometimes written off before they have had the opportunity to fulfill their potential. The second area concerned communication with the key social agents supporting the players, such as the parents and the wider support network, especially about what the players were trying to achieve in football. The third area concerned holistic player development and wellbeing through a focus on understanding the players outside of soccer contexts (e.g., life outside of soccer) and taking an interest in their overall wellbeing and psychosocial development.

Taken together, these studies emphasized the impact of TDEs on important player outcomes such as progression, wellbeing and mental toughness. By measuring the relative strengths and weaknesses of TDEs, the TDEQ enables practitioners and coaches to identify priorities in development and thus to promote the emergence of these positive player outcomes. Yet, by focusing primarily on the perceptions of players in periods that are considered culturally critical in soccer (i.e., transition to the professional team) [35] and/or on the perceptions of players embedded within several and different academies [16, 36], the studies omitted a part of the player development journey and were unable to provide insight into the coherence of a specific academy with its unique characteristics [25].

### TDEQ as a relevant tool for characterizing and optimizing the coherence of a talent pathway?

Although each country differs somewhat, the age of entry to the academies is usually between 14 and 15 years and exit is as late as 21 years for the International Centre for Sports Studies (S1 File). In the present study, the academy corresponds to what the Swiss Football Association defines as the performance center. This center is a specific training structure within clubs from M15 to M21 age-groups, meeting requirements specification defined by the Swiss Football Association. By focusing primarily on the perceptions of players in periods considered culturally critical in soccer (i.e., 17–18 years old), part of the player development journey is therefore obscured. Collecting the perceptions of all the players embedded in a given academy might offer a more holistic vision of the talent pathways.

Despite strong evidence of athletes’ changing needs along their developmental journey [14, 37, 38], no study has yet sought to describe how the set of the athletes in an academy perceive, or do not, whether their development environment is adapted to their situated needs. A closer look at these players’ perceptions and an analysis of TDE strengths and areas for improvement might thus be informative on the coherence of the talent pathways. For Webb et al. [25], the coherence of effective talent pathways is generally defined by structured and complementary actions framed against long-term agendas. Although the organizational and coaching environment is probably the most influential external factor to promote or disrupt coherence, few studies have explored how an entire set of coaches in one organization can shape smart, adaptable and resilient soccer players. In this regard, the TDEQ appears to be a reliable tool both to measure the extent to which elite soccer players experience the features of effective practice as they interact with their TDEs [29] and to examine the coherence of the talent pathways within a specific academy [12, 25] to promote positive player outcomes.

While following the line of research suggested by several authors to prioritize research quality and the applied impact, which requires contextually situated studies [39–42], this study aimed to examine the perceptions of elite soccer players embedded in a single Swiss academy concerning their TDE and then indicate how the TDEQ could also be used to identify priorities so that the academy could improve its program both within and across age-groups.

### The need for a French speaking-version of the TDEQ-5

Although the TDEQ has already been used with English-, Chinese- and Spanish-speaking athletes [43–46], it has never been used in a French-speaking population. Yet, French is the official language of 300 million people living in 32 countries (fifth most spoken language in the world) according to the International Organization of La Francophonie (2018 report). This version will thus be a major step in expanding the international scientific community that deals with TD programs. Given the number of French-speaking countries that have effective TD programs in soccer (e.g., Belgium, Canada, Ivory Coast, France, Morocco, Senegal, Switzerland), a French language questionnaire would be useful.

In its first version, the TDEQ was composed of seven factors and 59 items [30]. This version was particularly used by previous studies in the soccer context [16, 35, 36]. However, other studies sought to refine it and facilitate its use [44]. The result was a revised version with five factors and a 25-item scale [45, 46]. The five factors are long-term focus (F1: LTF), alignment of expectations (F2: AOE), communication (F3: COM), holistic quality preparation (F4: HQP) and support network (F5: SN). LTF measures the extent to which development programs are specifically designed to facilitate athletes’ long-term success (e.g., fundamental training and well-rounded development, ongoing opportunities, and de-emphasis on winning). AOE measures the extent to which goals for sports development are coherently set and aligned (e.g., goal-setting, goal-review, and individualized goals). COM measures the extent to which the coach communicates effectively with the athletes in both formal and informal settings (e.g., development path, rationale for training, and feedback). HQP measures the extent to which intervention programs are prepared both inside and outside of sports settings (e.g., caring coach, clear guidance, mental preparation, and balanced life). SN measures the extent to which a coherent, approachable, and wide-ranging support network is available for the athletes in all areas (e.g., professionals, parents, coaches and schools).

This revised 25-item five-factor model was first validated in talented Singaporean youth athletes [44]. Their results supported the adequate model fit, internal reliability (α = .79 to .86), convergent validity, and discriminant validity of the TDEQ-5. Two other studies then also examined the psychometric properties of the TDEQ-5 with 25 items in different contexts and specific athlete populations. The first one aimed to validate the TDEQ-5 with talented Chinese youth athletes [45]. Their confirmatory factor analysis revealed adequate model fit of the scale and supported internal reliability (α = .66 to .89). The second one [46] examined the psychometric properties of the TDEQ-5 in talented youth track and field athletes coming from six English-speaking Caribbean countries. Although their first confirmatory factor analysis did not meet all of the thresholds of good fit (i.e., several items had relatively weak standardized loadings), the model fit of the re-specified model was improved, especially by adding covariances between some items. Regarding internal reliability, two subscales, F1-LTF (α = .42) and F2-AOE (α = .57) had moderate to low internal reliability, and all the other subscale scores were deemed acceptable (α>.70). Consequently, even though the re-specified TDEQ-5 should be used with some caution with regard to the interpretation of results for F1-LTF and F2-AOE, it was considered a reliable measurement tool in the Caribbean context. In keeping with previous studies, this study aimed to validate the psychometric properties of the 25-item TDEQ-5 in talented Swiss athletes.

In conclusion, the aims of this study were twofold: first, to examine the psychometric properties of a French-language version of the 25-item TDEQ-5; and second, to examine how elite soccer players embedded in a Swiss soccer academy (from 14 to 18 years old) perceived the relative strengths and weaknesses of their TDE in order to identify the priorities for how the academy can be improved.

## Study 1. Adaptation and translation of the talent development environment questionnaire into French culture and language for talented athletes

### Method

A recommended methodology for the translation and cultural adaptation of questionnaires was applied (see e.g., [47]).

### Translation and cultural adaptation process

First, two native French translators with knowledge about the area of research conducted two forward translation versions of the TDEQ: each translator independently produced a forward translation of the instructions, original items, and answer options. Both translators then discussed the two translations and agreed on a single version. This process permitted additional changes to the original version where words or concepts were untranslatable, or where words or terms had a specific meaning in one language but a semantically different or secondary meaning in the French language (e.g., *"I would be given good opportunities"*). The following step was to compare each of the translated items to the original items and decided whether there were any discrepancies. A bilingual sports psychologist thus ensured that the translation of technical terms respected the original meanings. Few modifications were made (e.g., from “*My progress and personal performance is reviewed regularly on an individual basis”* to *"Mes progrès et mes performances sont régulièrement évalués individuellement"*). The next step was to administer the translated questionnaire to a sample of 15 French-speaking aspiring athletes between 12 and 20 years old to determine whether the items were clear on a 7-point Likert scale (1: very clear to 7: not clear at all) and to ask the athletes when items were scored above 4 to indicate issues in clarity. All the items were then modified. The next step was to back-translate the resulting French version into English by a professional native English-speaking translator who was also fluent in French. Last, the first and third researchers compared the final questionnaires in the two languages and decided on the final French version (S1 Appendix).

### Participants

Two hundred and three talented Swiss athletes, performing their sport at the regional (n = 21), national (n = 112) or international (n = 70) level, participated in this study. These athletes were considered talented on the basis of two criteria: (i) they were in possession of a Swiss Olympic Card, which rewards achievements in competition and/or attests to the potential for participation in the Swiss Olympics, and/or (ii) they played their sport at the highest possible level in Switzerland. Among them, 111 (55%) played a team sport (i.e., football, basketball, volley, hockey, curling, artistic swimming and rowing) and 92 (45%) an individual sport (i.e., skiing, athletics, equitation, swimming, cycling and judo). Our sample was composed with 63 females (31%) and 140 males (69%). The mean age was 16.99 years (age range: 13–21 yrs; SD = 2.46). There is no consensus regarding sample size calculation using confirmatory factor analysis (CFA): empirical rules vary from 5 to 10 subjects per item [48]. Since there were 25 items, the sample size (n = 203) fulfilled the empirical rule of at least 8 subjects per item. According to Swiss law, only the studies dealing with health data must be submitted for authorization to an Ethics Commission (Loi de recherche sur l’être humain). Since our study did not deal with health data, it has not been subjected to this legal requirement. However, data collection respected the common ethics rules used in psychology. For instance, procedures for data collection and analysis were explained to the athletes, who then gave written informed consent to participate, as did parents/guardians for those under 18 years. The athletes’ anonymity was guaranteed by an anonymous login created by each athlete and only the first researcher knew the link between the athlete and the login.

### Data analysis

All reverse-scored items were re-coded before data analysis. There were few missing data (8 values, corresponding to 0.16%). Little’s MCAR test revealed that the missing values were missing at random (χ2 = 150, p = .810). Therefore, they were imputed by the Expectation-Maximization (EM) algorithm of Little and Rubin [49]. Confirmatory factor analyses were performed to test the factorial validity of the TDEQ-5, treating item data as categorical ordinals. The estimation of the model used robust weighted least-squares estimators with adjustments for the mean and variance (WLSMV). The hypothesized five factors were tested. The evaluation of model fit was performed with χ2/df and completed with measures selected from other classes of fit indices [50]: the comparative fit index (CFI) and the Tucker-Lewis index (TLI), the root mean square error of approximation (RMSEA), 90% CI of RMSEA, and the standardized root mean square residual (SRMR). From the abundant but not fully consonant literature, we chose the following criteria to consider the model fit as acceptable: χ2/df <2 [51], CFI and TLI ≥ .90 and RMSEA and SRMR ≤ .08 [52]. Although these criteria were established for continuous indicators, simulation studies suggest that they work reasonably well with categorical ordinal indicators [53, 54].

To examine scale reliability, the internal consistency coefficients (Cronbach’s alphas) were calculated. DeVellis [48] defined a Cronbach’s alpha between .65 and .70 as a "minimally acceptable" threshold for a scale, while he defined a scale with thresholds greater than .70, .80 and .90, respectively, as "respectable," "very good" and "excellent." Following McNeisch [55], McDonald’s model-based Omega (ω) coefficient was also provided. For all tests, significance was set at *p* < .05. All data were analyzed using Jamovi (version 1.6.3) and Mplus statistical package (version 8.4).

### Results

As shown in Table 1, the first CFA indicated almost adequate fit, with only the CFI and TLI coefficients slightly less adequate. An examination of the factor loadings indicated that the loading was not significant for item 8 in F2-AOE (standardized estimate = 0.153); therefore, this item was removed. An examination of the modification indices indicated that two covariate links had to be added (between items 5–7 and items 12–24 of F4-HQP). The fit indices of the improved model were satisfactory according to the thresholds in the literature.

**Table 1:** Fit indices of the CFA models for TDEQ-5 (n = 203).

Model	χ^2^	*Df*	χ^2^ / *df*	*p*	CFI	TLI	RMSEA 90%CI	SRMR
Five-factor model	553.01	265	2.09	< .001	.89	.88	.07	.07
							.06 - .08	
Five-factor model improved	484.62	240	2.02	< .001	.91	.90	.07	.06
							.06 - .08	

*Note*. χ^2^: chi-square; df: degrees of freedom; *p*: significance level; CFI: comparative fit index; TLI: Tucker-Lewis index; RMSEA: root mean square error of approximation; SRMR: standardized root mean square residual.

The standardized loadings of this improved model were all higher than .30 (83% of them were higher than 0.40) and were all significant (Table 2).

**Table 2:** Characteristics of the five-factor model improved (n = 203).

Factor	Item #	Stand. Estimate	*p*	Cronbach’s α	McDonlad’s ω	M	SD
F1 –LTF				.70	.72	4.87	0.80
	2	.56	< .001				
	6	.53	< .001				
	18	.75	< .001				
	19	.76	< .001				
	21	.44	< .001				
F2- AOE				.71	.72	4.14	1.09
	15	.64	< .001				
	20	.57	< .001				
	23	.58	< .001				
	25	.80	< .001				
F3—COM				.77	.76	4.24	1.12
	3	.74	< .001				
	9	.77	< .001				
	17	.73	< .001				
	22	.56	< .001				
F4—HQP				.63	.65	4.41	0.76
	1*	.34	< .001				
	5*	.38	< .001				
	7 *	.31	< .001				
	11 *	.45	< .001				
	12 *	.63	< .001				
	16 *	.54	< .001				
	24 *	.66	< .001				
F5—SN				.61	.61	4.25	0.98
	13	.39	< .001				
	14	.45	< .001				
	4	.54	< .001				
	10	.62	< .001				

* Reversed items.

Participants had moderate to high scores on all factors (*M* = 4.14–4.87, Table 2), with similar standard deviations among factors (*SD* = 0.76–1.12). Cronbach’s alpha designated F1-LTF, F2-AOE and F3-COM as respectable scales, F4-HQP as minimally acceptable, and F5-SN as quasi-minimally acceptable. The results were roughly the same if the omega reliability coefficient was used.

### Discussion

The aim of this study was to adapt and validate a French version of the TDEQ-5 to give French-speaking sports practitioners and researchers a practical and reliable measure of effective talent development processes. The TDEQ is a quantifiable evidence-based approach to examine the TDE and its impact on athletes [30, 43, 45, 46], but its validity and applicability in the Swiss context and other French-speaking countries has been unknown.

The re-specified TDEQ-5 instrument has acceptable global model fit according to the thresholds in the literature. One item (Q8) were removed due to low factor loading. This item ("The advice my parents give me fits well will the advice I get from my coaches") should have been related to F2-AOE, which captures the extent to which goals for sports development are coherently set and aligned, but it was removed. The examination of the item loadings suggested that the items of this factor all concerned coaches’ and athletes’ alignment on and management of two essential matters: the athletes’ goals and the progress toward them. Indeed, item 25 (“I regularly set goals with my coach that are specific to my individual development”) loaded higher on this factor, and items 20 and 23 also significantly contributed (respectively, “My progress and personal performance is reviewed regularly on an individual basis” and “I am involved in most decisions about my sport development”). Item 15 (“My coaches make time to talk to my parents about me and what I am trying to achieve”) also loaded on this factor and, because it focuses on parents, may be linked to item 8. However, item 8 presents a different perspective by focusing on the role of the parents’ advice, while item 15 and the others focus on the role of the coach and athlete regarding the parents. This would explain why this item did not fit well with this factor.

The model re-specification, however, added two covariate links. Possible explanations for the item correlations are as follows. First, item 5 ("The guidelines in my sport regarding what I need to do to progress are not very clear") and item 7 ("I’m not taught that much about how to balance training, competition and recovery") also seemed to assess academy support for progress. Second, item 12 ("My coach doesn’t appear to be that interested in my life outside of sport") and item 24 ("My coach rarely talks to me about my wellbeing") were correlated, as both assess coach caring.

Although internal reliability was adequate for four of the TDEQ-5 subscales (i.e., F1-LTF, F2-AOE, F3-COM and F4-HQP), this was only slightly the case for F5-SN (0.61). The relatively low internal reliability was similar to findings reported in previous studies [43] of talented youth athletes in Spain (SN α = 0.65). A possible explanation may be that the item contents were not related between each other. In Switzerland, public policies tend to focus on recreational sport for health and very high-level performance (S2 and S3 Files), which may explain why many national-level athletes with limited financial support do not benefit from extended support networks and why these items may not be fully meaningful for them. Although a coherent, approachable and wide-ranging support network is required for the athlete’s development [14], what this network actually looks like may be quite different for individual versus team sports and for major versus minor sports, depending on the culture of the country.

To summarize, this French-adaptation of the TDEQ-5 appears to be a valuable instrument for assessing the effectiveness TD processes, even though some difficulties/problems with the cross-cultural adaptation occurred, as in previous research on Caribbean and Spanish athletes [43, 46].

## Study 2. Holistic analysis of talented soccer players’ perceptions of their development environment. A case study in a Swiss soccer academy

### Method

#### Participants

Sixty-four elite soccer players belonging to the same Swiss soccer academy participated in the study. This academy is considered one of the most effective in Switzerland and has obtained the label of "Performance Center" by the Swiss Soccer Association. This quality label requires that academies meet stringent criteria in relation to the quality of their youth development programs. The players were 14–18 years old (M age: 16.41 years, SD: 1.16). Nineteen players were on the M15 team, sixteen on the M16 team, thirteen on the M17 team, and sixteen on the M18 team. These four teams corresponded to four levels of competition and represented the pinnacle of elite youth soccer in Switzerland.

#### Procedure

In the context of an audit on TD in a Swiss professional soccer club, the club talent manager and the coaches agreed to have us present the objective of this study to their players. According to Swiss law, since our study did not deal with health data, it has not been submitted for authorization to an Ethics Commission (i.e., Loi de recherche sur l’être humain). However, data collection respected the common ethics rules used in psychology Before data collection commenced, informed consent was obtained from the players and their parents and/or guardians, which confirmed their understanding of the purpose of the study and their agreement to participate. The players’ anonymity was then guaranteed by an anonymous login created by each athlete and only the first researcher knew the link between the athlete and the login (see the CODE Column, S1 Data).

A data collection session was then organized. Administration of the French version of the TDEQ (TDEQ-FV) took place at the academy’s training facilities in a quiet auditorium under the supervision of the researchers, coaches and talent manager. Sixty-four players completed the questionnaire. The supervised on-site data collection ensured that all questions (i.e., each of the subscales) on the questionnaire had been answered. At the onset of collection, players were also informed that there were no right or wrong answers, given assurances about the anonymity of their responses (i.e., creation of personal identifiers), and encouraged to provide honest and direct answers. To further reduce social desirability, the participants were not asked to provide any identifiable details and were assured that any information emanating from the questionnaires would only be displayed as a group average. The questionnaires took approximately 7–10 minutes to complete. For ease of interpretation and in line with Mills and colleagues [35], all items in the present study were coded from 1 (strongly disagree) to 6 (strongly agree). For the negatively worded items corresponding to F4-HQP, the scores were reversed after the players had filled in the questionnaire (S1 Data). Thus, higher scores indicated perceptions of higher quality experience for the set of the five factors.

### Data analysis

The internal reliability of the TDEQ-FV was established with Cronbach’s alpha of the factors, and previous studies were followed to examine the players’ perspectives on their development environment. The data were analyzed with a four-step process.

First, descriptive statistics of subscale scores were calculated at the academy level (encompassing all the competition age-groups). Although there is no consensus on how to determine the dimensions of development or the relative strengths or weaknesses of the environment, the assumption was made that an average less than 4 indicated an area for improvement [36] and an average close to or greater than 5 as a relative strength of the environment from the players’ perspective.

Second, these mean subscale scores were calculated for each age-group embedded in the development environment (distinguishing M15, M16, M17 and M18 teams). The same standards as presented above were used to examine the teams’ scores.

Third, differences in subscale scores between teams (M15, M16, M17 & M18) were assessed with multivariate analysis of variance (MANOVA) and then univariate analyses with a Tukey correction to protect against type 1 error. The applicability of the multivariate analysis was confirmed with Box’s M-test for the homogeneity of covariance matrices and the Shapiro-Wilk normality test, and Levene’s test for the equality of variances was used for the univariate test. For one-way analysis of variance, Tukey post hoc tests were used. For all tests, significance was set at *p* < .05.

Fourth, given the recommendations of Martindale et al. [29, 30] to use item scores in conjunction with subscale scores when exploiting the TDEQ in applied research, all mean item scores <4 were identified to facilitate the identification of priority areas for improvement during the TD process. To do so, a second dimension was added: more than 50% of the age-group had to score the item ≤3, corresponding to “disagree a little bit”, “disagree” or “totally disagree” with the associated statement. Based on previous studies [30, 56], a qualitative content analysis of these statements was conducted for each age-group in order to produce meaningful applied opportunities for enhancing this specific TDE.

### Results

#### Players’ perceptions of the talent development environment

To add validity and accuracy to the interpretation of the data, we initially assess the reliability of the French version of the TDEQ (TDEQ-FV) using Cronbach’s alpha and McDonalds omega. This preliminary statistical analysis determined the internal consistency of the instrument factors. In the present study, three subscale alpha coefficients were found to be adequate and ranged between .62 and .68 (long-term development focus: α = .68; alignment of expectations: α = .65; communication: α = .62) (Table 3). Congruent with previous studies using the TDEQ [16, 35], the holistic quality preparation (α = .36) and support network (α = .45) factors demonstrated low internal reliability and were thus omitted for interpretation at the subscale level.

**Table 3:** TDEQ-FV subscale analysis (*n* = 64).

TDEQ subscale	Number of items	Subscale M	SD	Cronbach’s α	McDonlad’s ω
F1 –LTF	5	4.72	0.85	.68	.71
F2- AOE	4	3.89	0.93	.65	.66
F3—COM	4	4.06	0.97	.62	.64
F4 –HQP	7	4.29	0.74	.36	.44
F5 –SN	4	4.39	0.87	.45	.47

After establishing reliability, our focus was only on the mean scores for the first three factors. At the academy level, the players’ perceptions of long-term development focus were the most positive (M = 4.72, SD = 0.85), whereas the least positive perceptions centered on communication (M = 4.06, SD = .97) and on alignment of expectations (M = 3.89, SD = .93). As a reminder, a subscale mean score of <4 was taken to indicate an area for improvement in the TDE [36, 56].

#### Players’ perception of the talent development environment from each age-group

MANOVA with Pillai’s Trace showed a significant difference between age-groups in the first three subscales of the TDEQ-FV: V = .43, F(15, 174) = 1.92, *p* = .024, ηp2 = .142). Separate analyses on the outcome variables revealed significant differences in the first three subscales between age-groups of the TDEQ-FV (Table 4). The results of the post-hoc analyses presented in Table 4 indicated significant differences in the TDEQ subscales between M15 and both M17 and M18 for F1 (LTF), F2 (AOE) and F3 (COM), and between M16 and M17 for F1 (LTF).

**Table 4:** TDEQ-FV by age-group ((mean and standard deviation, ANOVA test *p*-value, n = 64).

Factor	M15	M16	M17	M18	*p*
	n = 19	n = 16	n = 13	n = 16	
F1-LTF	5.11 (0.57)	5.05 (0.59)	4.18 (0.85)	4.38 (1.01)	.002^a^
F2-AOE	4.46 (0.86)	3.97 (1.08)	3.38 (1.01)	3.55 (0.83)	.008^b^
F3-COM	4.61 (0.85)	4.08 (0.93)	3.38 (1.02)	3.81 (0.76)	.003^b^
F4-HQP	4.32 (0.97)	4.49 (0.61)	4.09 (0.64)	4.22 (0.61)	----
F5-SN	4.42 (1.05)	4.31 (0.74)	4.46 (0.89)	4.39 (0.80)	----

a M15 significantly different from M17 and M18, M16 significantly different from M17.

b M15 significantly different from M17 and M18.

#### Differences in the perceptions of players in different age-groups

As a reminder, this analysis is based only on the three first factors that showed adequate reliability (i.e., F1-LTF; L2-AOE; F3-COM). For all the competition age-groups (Table 4), the most positive perception was for long-term development focus. Although this subscale mean was ≥5 for M15 and M16 (i.e., high positive perceptions), it was ≤5 for M17 and M18. The least positive perception for all age-groups was centered on the alignment of expectations even if the perceptions for this factor differed according to age-group. For M15 the mean was between 4 and 5, whereas for M16, M17 and M18 they were respectively close to 4 then <4.

The analysis of the subscale means for each age-group also indicated a continuous decrease in the players’ positive perceptions of their development environment from M15 to M17, with a much sharper decrease for M17. Then, although perceptions improved somewhat for M18, the level remained lower than those of M16 and M15.

#### Priority areas for improvement

A content analysis of the areas for improvement as perceived by each age-group resulted in them being grouped as priority opportunities for development in this Swiss TDE (mean item score <4 and more of 50% of players in each age-group scored this item as ≤3 corresponding to totally disagree, disagree or disagree a little bit with the associated statement, see Table 5 and S1 Data).

**Table 5:** TDEQ-FV item analysis for each age-group.

Factor	TDEQ statements	M15	M16	M17	M18
		M (SD)	M (SD)	M (SD)	M (SD)
**F1**	I spend most of my time developing skills and attributes that my coach tells me I will need if I am to compete successfully at the professional level	5.36 (0.83)	4.93 (0.85)	3.84 (1.14)	4.75 (1.06)
	I would be given good opportunities even if I experienced a dip in performance	5.05 (1.02)	5.31 (0.94)	4 (1.58)	4.25 (1.77)
	My coach allows me to learn through making my own mistakes	5.84 (0.37)	5.5 (0.63)	4.3 (1.49)	4.81 (1.21)
	My training is specifically designed to help me develop effectively in the long-term	4.94 (1.23)	4.31 (0.70)	4 (1.15)	4.06 (1.61)
	My coach emphasizes that what I do in training and competition is far more important than winning	4.21 (1.52)	4.18 (1.22)	4.76 (1.48)	4 (1.46)
**F2**	My coaches make time to talk to my parents about me and what I am trying to achieve	4.05 (1.50)	4 (1.31)	3.07 (1.55)	3.12 (1.85)
	My progress and personal performance are reviewed regularly on an individual basis	4.61 (0.95)	4.18 (1.22)	4.07 (1.38)	4.12 (1.31)
	I am involved in most decisions about my sport development	4.77 (1.06)	4.06 (1.23)	3.53 (1.19)	3.93 (1.23)
	I regularly set goals with my coach that are specific to my individual development	4.52 (1.21)	3.81 (1.55)	2.84 (1.67)	3 (1.31)
**F3**	My coach explains how my training and competition program work together to help me develop	5.42 (0.83)	5.25 (0.85)	3.46 (1.56)	4.81 (0.98)
	My coach and I regularly talk about things I need to do to progress to the top level in my sport	4.94 (1.10)	3.93 (1.18)	3.46 (1.80)	3.43 (1.50)
	My coach and I often try to identify what my next big test will be before it happens	3.83 (1.58)	3.37 (1.54)	2.84 (1.40)	3.37 (1.25)
	My coach and I talk about what current and/or past world-class performers did to be successful	4.26 (1.55)	3.75 (1.57)	3.76 (1.36)	3.62 (1.40)
**F4**	I am rarely encouraged to plan for how I would deal with things that might go wrong	4.05 (1.55)	3.5 (1.46)	3.53 (0.87)	3.31 (1.66)
	The guidelines in my sport regarding what I need to do to progress are not very clear	4.94 (1.26)	5.31 (1.13)	4.30 (1.18)	4.93 (1.34)
	I am not taught that much about how to balance training, competition and recovery	4.63 (1.21)	4.06 (1.38)	4.53 (1.50)	4.43 (1.20)
	My coach rarely takes the time to talk to other coaches who work with me	3.89 (1.76)	4.81 (1.10)	4.61 (0.86)	4.25 (1.29)
	My coach does not appear to be that interested in my life outside of sports	4 (1.49)	4.5 (1.26)	3.84 (1.40)	3.87 (1.25)
	I don’t get much help to develop my mental toughness in sport	4.26 (1.72)	3.87 (1.74)	3.69 (1.49)	3.56 (1.36)
	My coach rarely talks to me about my wellbeing	4.15 (1.21)	4.37 (0.95)	3.53 (0.87)	4.25 (1.23)
**F5**	My coaches talk regularly to the other people who support me in my sport about what I am trying to achieve	3.78 (1.47)	3.37 (1.36)	3.30 (1.79)	3.68 (1.25)
	Those who help me in my sport seem to be on the same wavelength as each other when it comes to what is best for me	4.94 (1.10)	4.56 (1.15)	4.46 (1.26)	4.93 (1.34)
	Currently I have access to a variety of different types of professionals to help my sports development	4.05 (1.71)	3.93 (1.56)	4.76 (1.36)	4.43 (1.54)
	I can pop in to see my coach or other support staff whenever I need to	4.89 (1.24)	5.37 (0.88)	5.30 (1.10)	4.5 (2)

#### Areas for improvement for the M15 age-group

For M15, players reported on average that they neither agreed nor disagreed (i.e., item mean score between 3.5 and 4) with three statements: "My coaches talk regularly with the other people who support me in my sport about what I am trying to achieve" (M = 3.78; 37% of players scored this item ≤3); "My coach and I often try to identify what my next big test will be before it happens" (M = 3.83; 32% of players scored this item ≤3); and "My coach rarely takes the time to talk to other coaches who work with me" (M = 3.89; 37% of players scored this item ≤3). Even though with our preestablished criteria, no area for improvement for this age-group was identified, the relatively weakest statements described above suggest to improve the communications between coaches as well as the identification of the next big challenge for each player.

#### Areas for improvement for the M16 age-group

For this age-group, players reported on average that they disagreed a little bit (i.e., item mean score between 3 and 3.5) with the two following statements: "My coach and I often try to identify what my next big test will be before it happens" (M = 3.37; 62% players scored this item ≤3) and "My coach talks regularly with the other people who support me in my sport about what I am trying to achieve" (M = 3.37; 50% players scored this item ≤3). The content analysis of these statements resulted in them being grouped under one higher-order theme: communication regarding achievements with players and their support network. This theme was interpreted as the main opportunity for development of the M16 age-group.

On the contrary, whether players reported on average that they were between "disagree a little bit" and "agree a little bit" (i.e., item mean score between 3.5 and 4) for the statements "I am rarely encouraged to plan for how I would deal with things that might go wrong" (M = 3.5); "My coach and I talk about what current and/or past world-class performers did to be successful" (M = 3.75), and "I regularly set goals with my coach that are specific to my individual development" (M = 3.81), 44% of them scored these items ≤3, which indicates that more than half the players agreed a little bit or agreed with these statements.

#### Areas for improvement for the M17 age-group

For M17, players reported that on average they disagreed (i.e., item mean score <3) that that they and their coach regularly set goals that are specific to their individual development (M = 2.84) and often try to identify what the next big test will be before it happens (M = 2.84): 69% of the players scored these two statements ≤3, which indicated that two thirds of the players disagreed with these statements.

Players also reported that on average they disagreed a little bit (i.e., item mean score between 3 and 3.5) that their coach does not make time to talk regularly with their parents (M = 3.07) and the other people who support them about what they are trying to achieve (M = 3.30). For each of these associated statements, 62% of players scored these items ≤3, which indicated that two thirds of the players disagreed or disagreed a little bit with these statements. The content analysis of these statements resulted in them being grouped under one higher-order theme: planning for football-specific development. This theme was interpreted as the primary opportunity for development of the M17 age-group.

The M17 players also reported that on average they were between "disagree a little bit" and "agree a little bit" (i.e., item mean score close or between 3.5 and 4) with six other statements: "My coach explains how my training and competition program work together to help me develop" (M = 3.46; 69% of players scored this item ≤3); "My coach and I regularly talk about things I need to do to progress to the top level in my sport (M = 3.46; 54% of players scored this item ≤3); "I am involved in most decisions about my sport development" (M = 3.53; 62% of players scored this item ≤3); "My coach rarely talks to me about my wellbeing" (M = 3.53; 38% of players scored this item ≤3); and "I am rarely encouraged to plan for how I would deal with things that might go wrong" (M = 3.53; 39% of players scored this item ≤3). The content analysis of these statements resulted in them being grouped under one higher-order theme: communication regarding the player’s development plan. This theme was interpreted as the secondary opportunity for development for this specific age-group.

#### Areas for improvement for the M18 age-group

For the M18 age-group, players reported that on average they disagreed a little bit (i.e., item mean score under or close to 3.5) that they and their coach regularly set goals that are specific to their individual development (M = 3; 62% of players scored this item ≤3), that their coach makes time to talk regularly with their parents (M = 3.12; 75% of players scored this item ≤3), that they are encouraged to plan for how they would deal with things that might go wrong (M = 3.31; 56% of players scored this item a ≤3); that they and their coach regularly talk about things they need to do to progress to the top level in their sport (M = 3.43; 56% of the players scored this item ≤3), and that they get a good amount of help to develop their mental toughness in sports (M = 3.56; 56% of the players scores this item ≤3). The content analysis of these statements resulted in them being grouped under two higher-order themes: (i) planning for individual football-specific development and (ii) focusing on the player’s psychosocial development. The first theme contained goal-setting and identified areas for progress. The second theme contained the opportunity to develop psychosocial and coping skills to prepare players for future challenges. They were interpreted as the primary opportunities for development for this specific age-group.

The M18 players also reported that on average they were between "disagree a little bit" and "agree a little bit" for three statements (i.e., item mean score between 3.5 and 4): "My coaches talk regularly with the other people who support me in my sport about what I am trying to achieve" (M = 3.68; 50% of the players scored this item ≤3); "My coach and I talk about what currents and/or past world-class performers did to be successful" (M = 3.62; 56% of the players scored this item ≤3); and "My coach does not appear to be that interested in my life outside of sport" (M = 3.87; 50% of the players scored this item ≤3). The content analysis of these statements resulted in them being grouped under two higher-order themes: (i) communication with key social agents and more especially with parents and the support network about what the players are trying to achieve and (ii) focus on the player’s holistic development. These themes were interpreted as the secondary opportunities for development for the M18 age-group.

### Discussion

The aim of this study was to examine elite young soccer players’ perceptions of their TDEs with a view to providing an understanding of the strengths and areas for improvement within this specific TDE and how the academy can improve both within age-groups and coherently across groups.

Although there are still no evidence-based standards for defining cut-offs to indicate the relative strengths or weaknesses within an academy, a subscale/factor mean of 4/6 or above would indicate a relative strength in the TDE [35, 36]. From this perspective, all the players embedded in this Swiss soccer academy had mostly positive perceptions of the long-term development focus, and the least positive perceptions centered on communication and the alignment of expectations. The F3-COM and F2-AOE subscale mean scores could be associated with “agree a little bit” (i.e., score of 4) or were between “disagree a little bit” and “agree a little bit” (i.e., score between 3.5 and 4), suggesting opportunities for development at the academy level. More specifically, these opportunities were more strongly associated with the M17 and M18 age-groups, which scored F2-AOE factor <3.5 and F3-COM factor <4.

With its holistic and original design, this study examined all the perceptions of players’ embedded in a single TDE. By age-group (i.e., M15 to M18), the results indicated some relative strengths of the F1-LTF factor for the M15 and M16 groups (i.e., the subscale mean score was >5). Moreover, the score for the M15 age-group was significantly different from the scores for the M17 and M18 age-groups, and this was also observed for M16 compared to M17. These results suggest that players in the M15 and M16 age-groups thought that the coach and the whole academy placed sufficient and meaningful emphasis on their longer-term athletic development [14]. In comparison, the players of the M17 and M18 age-groups perceived their coach and the academy as affording them far fewer opportunities to develop resources to facilitate long-term success (i.e., ongoing opportunities, clear expectations, fundamental training and well-rounded development, de-emphasis of winning, etc.).

This finding means that for talented Swiss soccer players, a meaningful transition was perceived between the M15-M16 and M17-M18 age-groups. The players’ situated and evolving needs along their development pathway [37, 38] were not fully met by the academy, which was seen as needing to provide more and adaptable support to the players. The results for the M17 age-group were particularly striking as the players felt that the coach communicated less or not at all about the reasons for training sessions and gave inadequate or insufficient support during challenging situations. This may explain why the players experienced these interactions as unfruitful. Even though these results echo the challenges of coaching Gen Z athletes [57] and the role of social support in coping with challenging situations [58], they merit more in-depth examination to better grasp what is at stake during this critical transition from the players’ perspective.

Overall, these findings raise questions about the predominance of studies that focused only on the transition from youth soccer teams to professional teams [38, 59] and generally downplayed the long pathway before this momentous transition. These results also seem to be linked to the Swiss training model, which shifts the training objectives for the M17 and M18 age-groups (i.e., focused on reaching the professional level). This transition probably requires more from the players and a change in coaching methods, for which the players do not seem sufficiently prepared. The results therefore seem to question the way this transition is anticipated and collaboratively prepared in the Swiss context. It seems particularly important to access and clarify the players’ expectations about their personal objectives at the risk of amplifying some of the features inherent to all athletes belonging to Gen Z, such as their limited ability to deal with adversity (e.g., some had never been exposed to adversity prior this transition). It can also be assumed that the strategies of the academy’s coaches to facilitate this transition were insufficient and/or ineffective from the players’ perspective.

The results also indicated how the alignment of expectations and communication were characterized by significantly lower scores for M17 and M18 in comparison with M15. These results suggest that when the players first entered their TDE they experienced a set of affordances to develop and flourish, which thereafter were perceived as less rich and/or abundant. More specifically, the M17 group’s very low score for F3-COM (i.e., score <3.5) suggest that this factor should be a priority for improvement for this academy and the coaches responsible for this age category. Indeed, it is widely acknowledged that the inefficacy of coach-player communications can foster conflict or impede the player’s long-term development [60]. In Switzerland, the M17 age-group was created for players considered insufficiently "ready" to immediately enter into M18; they are thus able to remain in a more suitable competition group within the academy. This points to the two parallel paths for reaching a high level: a direct path by joining M18 and a diverted path by joining M17 before potentially joining M18. This latter path has the merit of giving the academy an extra year to work with potentially top players. However, the results and negative perceptions concerning this factor (i.e., score <3.5) indicated that the M17 players understood the situation quite differently, probably considering this fork in the development path as a significant transition in their development.

Moreover, the results also highlighted very low scores for F2-AOE (i.e., score ≤3.5) for the M17 and M18 age-groups. A specific item of this factor assesses the communication between coaches, players and players’ parents. The entourage is very important to these young players, even though it may create additional challenges for coaches [61]. The over-involvement of parents is, for example, a paradigmatic example during the athlete’s development [62]. In fact, none of the stakeholders in the player’s network can be set aside, despite being sources of frustration. It is nevertheless crucial for the players to minimize sources of confusion between these stakeholders. Also, parental involvement in their child’s sports project could encourage the emergence of a social support network that is consistent with the coach’s actions. Once again, this suggests that there is no single transition for youth soccer players toward the professional environment, but rather a set of transitions, specific to the sports culture and the structuring of the academies [24, 53]. Coaches and those in charge of TDEs need to identify and support players in managing these transitions. To achieve this objective, the TDEQ offers useful information to adjust/adapt the environment to players’ situated and evolving needs.

#### Practical implications

TDEQ is a tool that can help academies meet the needs of developing players along their developmental pathway [29]. In this regard, the findings offer two practical implications.

First, they underline how the TDEQ can be used to identify priorities for improvement, in this case for a Swiss soccer academy to improve its program across age-groups. More specifically, the findings described the need to (better) manage the transition for the M17 age-group along the Swiss players’ developmental pathway. Two key opportunities for development can be suggested to support this specific transition: (i) planning for football-specific development and (ii) communication regarding the player’s development plan. A specific individual development plan would give players a clear perspective on the stages and/or challenges they are likely to encounter and the skills/resources they will need to develop to successfully deal with them [15, 35, 38]. The coach’s regular communication on and review of this plan with the players would help the players to better understand the justifications and/or explanations for their training sessions, as well as provide them with the possibility of following their own progress [12, 15, 17]. In a time when pressures and doubts may appear to be greater and/or more intense, helping players to focus on what they can do to develop would thus be beneficial by creating a more positive motivational climate.

Second, the findings also underline how the TDEQ can be used to identify and inform on group-specific priorities so that the academy can improve coherently across age-groups (M15, M16, M17 & M18). This tool is particularly useful for tracking gaps or weakening in the essential coherence. A key point of this study was the tendency for the M16 and M17 players to gradually perceive their interactions with the environment less positively for the F1-LTF, F2-AOE and F3-COM factors. These talented soccer players seemed to have different and situated needs over the course of their pathways and/or it may have been that the resources afforded by the TDE became progressively insufficient and/or inadequate from their perspective. This raises questions about the coherence of talent pathways as programs designed to continuously support athletes with the potential to reach the professional level [25]. For example, even though the M17 age-group average for F1-LTF could be considered satisfactory, it was statistically different from those of the M15 and M16 age-groups, which means that the M17 players perceived fewer opportunities to develop and flourish over the long term. Since coherence in a TDE can be characterized by progressive, combined coaching methods adapted and applied to players’ situated and evolving needs [12], this result raises questions. In this specific case, it appears crucial to understand why players in one age-group perceived significantly fewer long-term development opportunities compared to the two younger age-groups.

## General discussion

The aim of this study was to examine talented soccer players’ perceptions about their development environment.

For psychometric reasons, the TDEQ-5 subscales were adapted to be suitable for a French-speaking context. A holistic design was then adopted to examine the perceptions of a set of players embedded in the same environment, which revealed the strength and relevance of this instrument to assess the perceived quality of the environment for different competition age-groups. These age-group-related differences between the players’ perceptions were quickly and easily identified, and the results may be a starting point for optimizing talent pathways by increasing the effectiveness of TD processes. This instrument was especially suitable for identifying the players’ difficult interactions with the environment. One of the main results was that the development pathways of soccer players cannot be summarized by a single transition toward a professional team. Rather, context-specific requirements come into play in the Swiss TD field, notably a critical period between M16 and M17.

Another conclusion is that coaches’ competencies should be supported and enhanced [61] by expanding the resources afforded by TDEs to meet players’ situated needs (e.g., through greater knowledge-sharing on developing mental toughness, fostering wellbeing, providing guidelines on the balance between training, competition and recovery, maintaining the athlete’s commitment, adapting one’s coaching style, etc.). Finally, psychometric concerns with the adaptation of the questionnaire emerged through the difficulties the players expressed in assessing the quality of their TDE. In this obvious complexity, it can be assumed that some perceptions evolved over time and some interactions probably did not have the same meaning for the players all along their pathway.

Future studies that use the TDEQ should focus on a set of athletes embedded in the same TDE, as did Thomas et al. [46] with Caribbean athletes, and should assess athletes’ perceptions according to the age-group or stage of development. This would undoubtedly lead to a more in-depth understanding of the good practices experienced by athletes along their development pathways. Finally, as exploited by Hall et al. [56], TDEQ might also be used to structure and drive practical interventions as well as to monitor the impact of these interventions over time.

## Supporting information

S1 File CIES football observatory monthly report (Issue 33)—a comparative analysis of club-trained players in Europe. (PDF)

S2 File Activités et consommation sportives de la population suisse (Office Fédéral du Sport, 2014). (PDF)

S3 File Le sport d’élite en suisse—etat des lieux (SPLISS = Sports Policy Factors Leading to International Sporting Success) (Haute Ecole Fédérale de Sport de Macolin, 2014). (PDF)

S1 Appendix The 5-factor talent development environment questionnaire. (DOCX)

S1 Data The Swiss elite players’ perception of the talent development environment from each age-group. (XLSX)

## References

[1] Bennett KJM, Vaeyens R, Fransen J. Creating a framework for talent identification and development in emerging football nations. Science and Medicine in Football. 2019;3(1):36–42.

[2] Relvas H, Littlewood M, Nesti M, Gilbourne D, Richardson D. Organizational Structures and Working Practices in Elite European Professional Football Clubs: Understanding the Relationship between Youth and Professional Domains. European Sport Management Quarterly. 2010;10(2):165–87.

[3] Richardson D, Gilbourne D, Littlewood M. Developing support mechanisms for elite young players in a professional soccer academy: Creative reflections in action research. European Sport Management Quarterly. 2004;4(4):195–214.

[4] Johnston K, Wattie N, Schorer J, Baker J. Talent Identification in Sport: A Systematic Review. Sports Med. 2018;48(1):97–109. 10.1007/s40279-017-0803-2 29082463

[5] Vaeyens R, Lenoir M, Williams AM, Philippaerts RM. Talent Identification and Development Programmes in Sport. Sports Medicine. 2008;38(9):703–14. 10.2165/00007256-200838090-00001 18712939

[6] Williams AM, Reilly T. Talent identification and development in soccer. Journal of Sports Sciences. 2000;18(9):657–67. 10.1080/02640410050120041 11043892

[7] Johnston K, Baker J. Waste Reduction Strategies: Factors Affecting Talent Wastage and the Efficacy of Talent Selection in Sport. Frontiers in psychology. 2020;10:2925. 10.3389/fpsyg.2019.02925 31998188 PMC6967295

[8] Güllich A. Selection, de-selection and progression in German football talent promotion. European Journal of Sport Science. 2014;14(6):530–7. 10.1080/17461391.2013.858371 24245783

[9] Abbott A, Button C, Pepping GJ, Collins D. Unnatural selection: talent identification and development in sport. Nonlinear Dynamics Psychol Life Sci. 2005;9(1):61–88. 15629068

[10] Till K, Baker J. Challenges and [Possible] Solutions to Optimizing Talent Identification and Development in Sport. Frontiers in psychology. 2020;11:664. 10.3389/fpsyg.2020.00664 32351427 PMC7174680

[11] Barab SA, Plucker JA. Smart People or Smart Contexts? Cognition, Ability, and Talent Development in an Age of Situated Approaches to Knowing and Learning. Educational Psychologist. 2002;37(3):165–82.

[12] Collins D, MacNamara A. Talent development: A practitioner guide. Abingdon; Oxfordshire:: Routledge; 2017.

[13] Davids K, Güllich A, Shuttleworth R, Araújo D. Understanding environmental and task constraints on talent development: Analysis of micro-structure of practice and macro-structure of development histories. Routledge handbook of talent identification and development in sport: Routledge; 2017. p. 192–206.

[14] Martindale RJ, Collins D, Daubney J. Talent development: A guide for practice and research within sport. Quest. 2005;57(4):353–75.

[15] Mills A, Butt J, Maynard I, Harwood C. Toward an understanding of optimal development environments within elite English soccer academies. The Sport Psychologist. 2014;28(2):137–50.

[16] Ivarsson A, Stenling A, Fallby J, Johnson U, Borg E, Johansson G. The predictive ability of the talent development environment on youth elite football players’ well-being: A person-centered approach. Psychology of Sport and Exercise. 2015;16:15–23.

[17] Larsen CH, Alfermann D, Henriksen K, Christensen MK. Successful talent development in soccer: The characteristics of the environment. Sport, Exercise, and Performance Psychology. 2013;2(3):190–206.

[18] Li C, Wang CJ, Pyun DY. Talent development environmental factors in sport: A review and taxonomic classification. Quest. 2014;66(4):433–47.

[19] Martindale RJ, Collins D, Abraham A. Effective talent development: The elite coach perspective in UK sport. Journal of applied sport psychology. 2007;19(2):187–206.

[20] Sarmento H, Anguera MT, Pereira A, Araújo D. Talent identification and development in male football: a systematic review. Sports Medicine. 2018;48(4):907–31. 10.1007/s40279-017-0851-7 29299878

[21] Henriksen K. The ecology of talent development in sport: A multiple case study of successful athletic talent development environments in Scandinavia. 2010.

[22] Collins D, MacNamara Á. The rocky road to the top. Sports medicine. 2012;42(11):907–14. 10.1007/BF03262302 23013519

[23] Dohme L-C, Bloom GA, Piggott D, Backhouse S. Development, Implementation, and Evaluation of anAthlete-Informed Mental Skills Training Program for EliteYouth Tennis Players. Journal of Applied Sport Psychology. 2019:1–21.

[24] Collins DJ, Macnamara A, McCarthy N. Putting the bumps in the rocky road: optimizing the pathway to excellence. Frontiers in psychology. 2016;7:1482. 10.3389/fpsyg.2016.01482 27733841 PMC5039182

[25] Webb V, Collins D, Cruickshank A. Aligning the talent pathway: exploring the role and mechanisms of coherence in development. Journal of sports sciences. 2016;34(19):1799–807. 10.1080/02640414.2016.1139162 26788766

[26] Lauer L, Gould D, Roman N, Pierce M. Parental behaviors that affect junior tennis player development. Psychology of sport and exercise. 2010;11(6):487–96.

[27] Taylor RD, Collins D, Carson HJ. Sibling interaction as a facilitator for talent development in sport. International Journal of Sports Science & Coaching. 2017;12(2):219–30.

[28] Wormhoudt R, Savelsbergh GJ, Teunissen JW, Davids K. The athletic skills model: optimizing talent development through movement education: Routledge; 2017.

[29] Martindale RJ, Collins D, Douglas C, Whike A. Examining the ecological validity of the Talent Development Environment Questionnaire. Journal of sports sciences. 2013;31(1):41–7. 10.1080/02640414.2012.718443 22917218

[30] Martindale RJ, Collins D, Wang JC, McNeill M, Lee KS, Sproule J, et al. Development of the talent development environment questionnaire for sport. Journal of sports sciences. 2010;28(11):1209–21. 10.1080/02640414.2010.495993 20694933

[31] John Wang CK, Sproule J, McNeill M, Martindale RJ, Lee KS. Impact of the talent development environment on achievement goals and life aspirations in Singapore. Journal of Applied Sport Psychology. 2011;23(3):263–76.

[32] Li C, Martindale R, Sun Y. Relationships between talent development environments and mental toughness: The role of basic psychological need satisfaction. Journal of sports sciences. 2019;37(18):2057–65. 10.1080/02640414.2019.1620979 31126227

[33] Thomas CE, Gastin PB, Abbott G, Main LC. Impact of the talent development environment on the wellbeing and burnout of caribbean youth track and field athletes. European Journal of Sport Science. 2020;20:1–29. 10.1080/17461391.2019.1606286 32460638

[34] Li C, Wang C, Pyun DY. The roles of the talent development environment on athlete burnout: A qualitative study. International Journal of Sport Psychology. 2017;48:143–64.

[35] Mills A, Butt J, Maynard I, Harwood C. Examining the development environments of elite english football academies: The players’ perspective. International Journal of Sports Science & Coaching. 2014;9(6):1457–72.

[36] Gledhill A, Harwood C. Toward an understanding of players’ perceptions of talent development environments in Uk female football. Journal of Applied Sport Psychology. 2019;31(1):105–15.

[37] Blijlevens SJ, Elferink-Gemser MT, Wylleman P, Bool K, Visscher C. Psychological characteristics and skills of top-level Dutch gymnasts in the initiation, development and mastery stages of the athletic career. Psychology of Sport and Exercise. 2018;38:202–10.

[38] Dowling C, Reeves MJ, Littlewood MA, Nesti M, Richardson D. Developing individuals whilst managing teams: perspectives of under 21 coaches within English Premier League football. Soccer & Society. 2018;19(8):1135–50.

[39] Collins D, MacNamara Á, Cruickshank A. Research and practice in talent identification and development—Some thoughts on the state of play. Journal of Applied Sport Psychology. 2019;31(3):340–51.

[40] Gesbert V, Durny A. A Case Study of Forms of Sharing in a Highly Interdependent Soccer Team During Competitive Interactions. Journal of Applied Sport Psychology. 2017;29(4):466–83.

[41] Hauw D. Énaction et intervention en psychologie du sport chez les sportifs élites et en formation. Canadian Journal of Behavioural Science/Revue Canadienne des Sciences du Comportement. 2018;50(1):54.

[42] Villemain A, Hauw D. A situated analysis of football goalkeepers’ experiences in critical game situations. Perceptual and Motor skills. 2014;119(3):811–24. 10.2466/25.30.PMS.119c30z0 25456246

[43] Brazo-Sayavera J, Olivares PR, Andronikos G, Martindale RJ. Spanish version of the Talent Development Environment Questionnaire for sport: Cultural adaptation and initial validation. PloS one. 2017;12(6). 10.1371/journal.pone.0177721 28582387 PMC5459334

[44] Li C, Wang CKJ, Pyun DY, Martindale R. Further development of the talent development environment questionnaire for sport. Journal of sports sciences. 2015;33(17):1831–43. 10.1080/02640414.2015.1014828 25774438

[45] Li C, Martindale R, Wu Y, Si G. Psychometric properties of the Talent Development Environment Questionnaire with Chinese talented athletes. Journal of sports sciences. 2018;36(1):79–85. 10.1080/02640414.2017.1282619 28134001

[46] Thomas CE, Abbott G, Gastin PB, Main LC. Construct validity and reliability of the Talent Development Environment Questionnaire in Caribbean youth track and field athletes. Plos one. 2020;15(1):e0227815. 10.1371/journal.pone.0227815 31978072 PMC6980498

[47] Gesbert V, von Roten FC, Hauw D. Validation of a French Version of the Psychological Characteristics of Developing Excellence Questionnaire (MacNamara & Collins, 2011): A Situated Approach to Talent Development. J Sports Sci Med. 2018;17(4):656–61. 30479535 PMC6243611

[48] DeVellis RF. Scale development: Theory and applications. Newbury Park: Sage publications; 2016.

[49] Little RJ, Rubin DB. Statistical analysis with missing data. New York: John Wiley; 1986.

[50] Nunnally J, Bernstein I. Psychometric theory, 3rd edn., internat. stud. ed.,[Nachdr.]. McGraw-Hill Series in Psychology. Tata McGraw-Hill, New Delhi; 2010.

[51] Ullman J. Structural equation modeling. U: BG Tabachnick, LS Fidel (ur.)-Using Multivariate Statistics. Allyn & Bacon, Needham Heights; 2001.

[52] Lt Hu, Bentler PM. Cutoff criteria for fit indexes in covariance structure analysis: Conventional criteria versus new alternatives. Structural equation modeling: a multidisciplinary journal. 1999;6(1):1–55.

[53] Yu C-Y. Evaluating cutoff criteria of model fit indices for latent variable models with binary and continuous outcomes: University of California, Los Angeles Los Angeles, CA; 2002.

[54] Muthén B. Mplus Technical Appendices. Los Angeles, CA: Muthén and Muthén; 2004.

[55] McNeish D. Thanks coefficient alpha, we’ll take it from here. Psychological Methods. 2018;23(3):412. 10.1037/met0000144 28557467

[56] Hall AJ, Jones L, Martindale RJ. The Talent Development Environment Questionnaire as a Tool to Drive Excellence in Elite Sport Environments. International Sport Coaching Journal. 2019;6(2):187–98.

[57] Gould D, Nalepa J, Mignano M. Coaching generation Z athletes. Journal of Applied Sport Psychology. 2020;32(1):104–20.

[58] Savage J, Collins D, Cruickshank A. Exploring traumas in the development of talent: what are they, what do they do, and what do they require? Journal of Applied Sport Psychology. 2017;29(1):101–17.

[59] Morris R, Tod D, Eubank M. From youth team to first team: An investigation into the transition experiences of young professional athletes in soccer. International Journal of Sport and Exercise Psychology. 2017;15(5):523–39.

[60] Davis L, Jowett S. Coach-athlete attachment and the quality of the coach-athlete relationship: implications for athlete’s well-being. J Sports Sci. 2014;32(15):1454–64. 10.1080/02640414.2014.898183 24713087

[61] Grecic D, Collins D. The epistemological chain: Practical applications in sports. Quest. 2013;65(2):151–68.

[62] Jowett S, Timson-Katchis M. Social networks in sport: Parental influence on the coach-athlete relationship. The Sport Psychologist. 2005;19(3):267–87.

