# Supplementary material for: Reviewing the role of the environment in the talent development of a professional soccer club
Source: PLoS One. 2021 Feb 25;16(2):e0246823. doi: 10.1371/journal.pone.0246823 (PMC7906372; doi:10.1371/journal.pone.0246823)
Supplement: S1 File — (PDF) [file pone.0246823.s001.pdf]

CIES Football Observatory Monthly Report
Issue 33 - March 2018

A comparative analysis of club-trained players in Europe

Drs Raffaele Poli, Loïc Ravenel and Roger Besson

1. Introduction

The analysis of the presence of club-trained players in squads across Europe is particularly interesting when it comes to comparing football cultures and strategies on an international level. Following UEFA and numerous national associations, the notion of club-trained players defines footballers who have spent at least three years between the ages of 15 and 21 in their team of employment.

The report covers first team squad members of clubs from 31 top division European leagues[1]. It relies on the annual census carried out by the CIES Football Observatory on the 1st of October of every year since 2009. To be included in the sample, a footballer should have already played in domestic league games during the season of reference, or, if this was not the case, to have taken part in adult championship matches during each of the two previous seasons. The second and third goalkeepers were considered in all cases.

1 [AUT] Austria, [BEL] Belgium, [BLR] Belarus, [BUL] Bulgaria, [CRO] Croatia, [CYP] Cyprus, [CZE] Czech Republic, [DEN] Denmark, [ENG] England, [ESP] Spain, [FIN] Finland, [FRA] France, [GER] Germany, [GRE] Greece, [HUN] Hungary, [ISR] Israel, [ITA] Italy, [NED] Netherlands, [NOR] Norway, [POL] Poland, [POR] Portugal, [ROM] Romania, [RUS] Russia, [SCO] Scotland, [SRB] Serbia, [SUI] Switzerland, [SVK] Slovakia, [SVN] Slovenia, [SWE] Sweden, [TUR] Turkey and [UKR] Ukraine.

2. Club-trained players' trends and gaps

Between 2009 and 2017, the proportion of club-trained players in the squads of teams analysed went down year by year from 23.2% to 18.4%. This finding highlights the intensification of the mobility of footballers from an early age, as well as the ineffectiveness of measures put into place to encourage the employment of home-grown footballers.

The average proportion of club-trained players among squad members in the leagues and period surveyed was 21.2%. The highest percentage was recorded in the Slovakian top division: 36.3%. This is four times more than in the Turkish Süper Lig (8.8%). Mediterranean countries occupy the bottom five positions of the rankings: Turkey, Italy, Portugal, Cyprus and Greece. This reveals the existence of a cultural approach that does not consider the promotion of local talents as a priority.

Figure 1: % of club-trained players in squads, 31 top division European leagues (2009-2017)

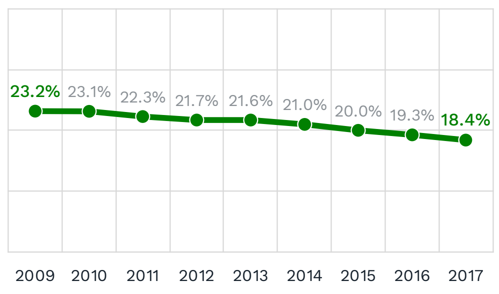

Figure 2: % of club-trained players in squads, by league (2009-2017)

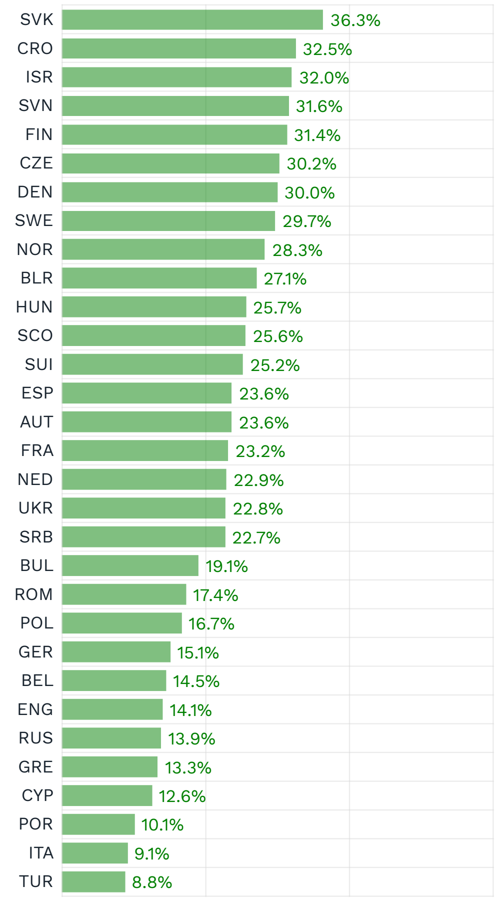

The average presence of club-trained players correlates with the average age of squads (fig. 3). Belgium and Portugal are the main residuals. In both cases, one would expect a significantly higher proportion of club-trained footballers with respect to the relatively young age of squad members. This finding mainly reflects the tendency for top division Belgian and Portuguese teams to import young talents from abroad. It also shows the high internal mobility of national players.

Conversely, in Slovakia, Israel and the Czech Republic, the proportion of club-trained players is much higher than the average age of squads would have suggested. In all these countries, expatriates represent a relatively small proportion of players. Furthermore, national transfers are not very frequent. Club-trained players tend thus to stay longer in the team that trained them compared to the situation observed at European level.

A significant gap in the percentage of club-trained footballers also exists between goalkeepers and outfield players. The lowest rate overall was recorded for forwards (17.7%). These gaps reflect different levels of mobility in the football players' labour market. Forwards are indeed much more mobile than goalkeepers, as well as, even though to a lesser extent, than defenders and midfielders.

Figure 3: average age and % of club-trained players, by league (2009-2017)

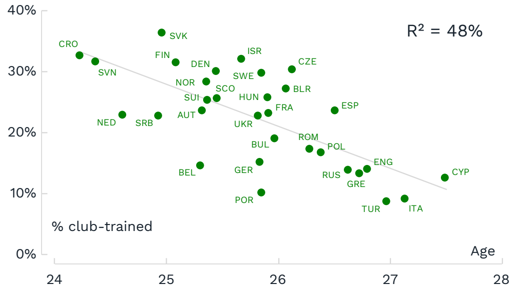

Figure 4: % of club-trained players in squads, by position (2009-2017)

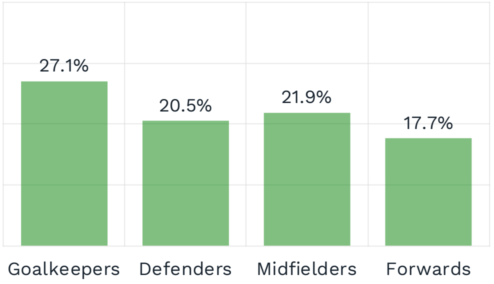

3. Club-trained players and success

A significant correlation was also measured between the relative presence of club-trained players in squads and the sporting level of a league (fig. 5). The proportion of club-trained footballers decreases in parallel with the increase in the sporting competitiveness of a league. Cyprus and Turkey are the main negative residuals. In both cases, national clubs under-perform in European competitions with respect to the very low level of club-trained players in their squads.

However, no correlation exists between the percentage of club-trained footballers and the sporting level of clubs. As illustrated in the figure 6, only the least competitive teams (CIES club coefficient <0.5) have a higher percentage of players from the youth academy than the very best European clubs (>1.25). The lowest proportion of club-trained footballers was recorded for teams in the two intermediate level categories, where transfer market speculation is the strongest.

Figure 5: % of club-trained players and average UEFA coefficient, by league (2009-2017)

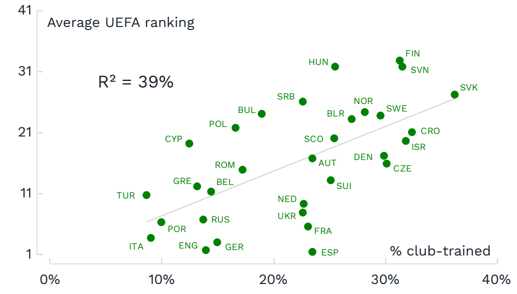

Figure 6: % of club-trained players and sporting level of teams* (2009-2017)

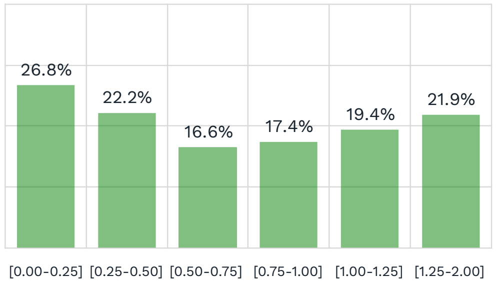

* CIES Football Observatory coefficient

On average, for all of the leagues and seasons covered, the average percentage of club-trained players among champion winning teams was 24.1%. This is a higher percentage than that measured for all teams as a whole (21.2%). This finding confirms the importance of being able to train top level footballers to achieve sustainable success. The highest percentages of club-trained footballers for a champion were measured for FC Barcelona (2012/13) and Viitorul Constanta (2016/17): 57.7% in both cases.

At the opposite end of the spectrum, since 2009, only five teams from the leagues surveyed won the title with no club-trained players in the squad: Ferencvárosi, Ludogorets, Olympiacos, Cluj and Salzburg. Juventus is the only big-5 league team in the top ten. This result also suggests that having at least a few home-grown footballers in the squad is important to achieve success regardless of competition level.

Figure 7: highest % of club-trained players, champions of 31 top division European leagues (2009-2017)

FC Barcelona (ESP)	2012	57.7%
FC Viitorul Constanța (ROM)	2016	57.7%
AFC Ajax (NED)	2010	55.2%
FC Koper (SVN)	2009	52.4%
Maccabi Haifa FC (ISR)	2010	52.4%
Aalborg BK (DEN)	2013	52.4%
Maccabi Haifa FC (ISR)	2009	52.4%
Malmö FF (SWE)	2014	50.0%
AS Trenčín (SVK)	2014	50.0%
IF Elfsborg (SWE)	2012	47.8%

Figure 8: lowest % of club-trained players, champions of 31 top division European leagues (2009-2017)

Ferencvárosi TC (HUN)	2015	0.0%
PFC Ludogorets Razgrad (BUL)	2013	0.0%
Olympiacos FC (GRE)	2013	0.0%
CFR 1907 Cluj (ROM)	2009	0.0%
FC RB Salzburg (AUT)	2009	0.0%
WKS Śląsk Wrocław (POL)	2011	3.7%
FC Porto (POR)	2011	3.8%
FC Basel 1893 (SUI)	2016	3.8%
Juventus FC (ITA)	2015	4.0%
FC Steaua Bucureşti (ROM)	2013	6.7%

The average percentage of club-trained players among champion teams per league also reveals the existence of great cultural differences in European football. At one extreme, Greek (mainly Olympiacos), Portuguese (mainly Porto and Benfica) and Bulgarian (mainly Ludogorets) champions relied on a very limited number of club-trained footballers. At the other, the latter had a much more crucial role in the Netherlands (mainly Ajax and PSV) and Spain (mainly Barcelona and Real).

Figure 9: average % of club-trained players for champions, by league (2009-2017)

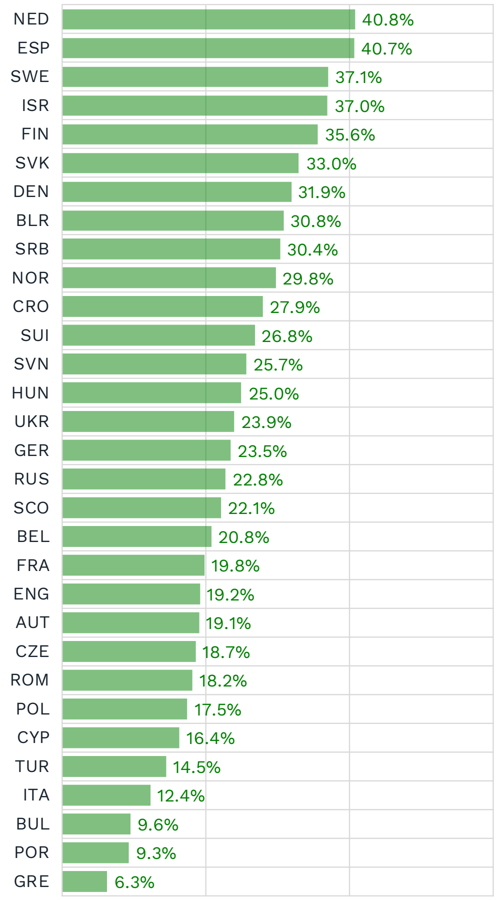

4. Conclusion

This report reveals that the ability to train top level footballers is part of the competitive advantage held by the most successful European clubs. However, this finding is partially related to the current club-trained player definition. Indeed, after three years, a player recruited up until the age of 18 can still be considered as a club-trained footballer for the team that signed him. This encourages top European clubs to lure the best talents initially trained by less competitive teams.

A change in the definition of club-trained or association-trained players could be useful to ensure a sounder development of the European game. Lowering the relevant age range from 15 to 21 years to 12 to 17 years would be helpful in limiting the increasing speculation around the transfer of minors[2]. Indeed, according to FIFA rules, communitarian players can only move abroad after their 16th birthday. This would de facto disallow recruiting teams or associations to acquire a training status.

While having a well performing youth academy does not lead directly to success, the report shows that it is a good indicator of the club's ability to look to the future. The existence of a powerful youth setting is also a good indicator of the club's strength as a territorially embedded organisation. Beyond short-term results, investing in youth training can be considered a gauge for the sustainable development of the club as an institution.

2 On this aspect, please refer issue number 20 of the **CIES Football Observatory Monthly report**.
