# Supplementary material for: Reviewing the role of the environment in the talent development of a professional soccer club
Source: PLoS One. 2021 Feb 25;16(2):e0246823. doi: 10.1371/journal.pone.0246823 (PMC7906372; doi:10.1371/journal.pone.0246823)
Supplement: S2 File — (PDF) [file pone.0246823.s002.pdf]

Sport Suisse 2014

Activité et consommation sportives de la population suisse

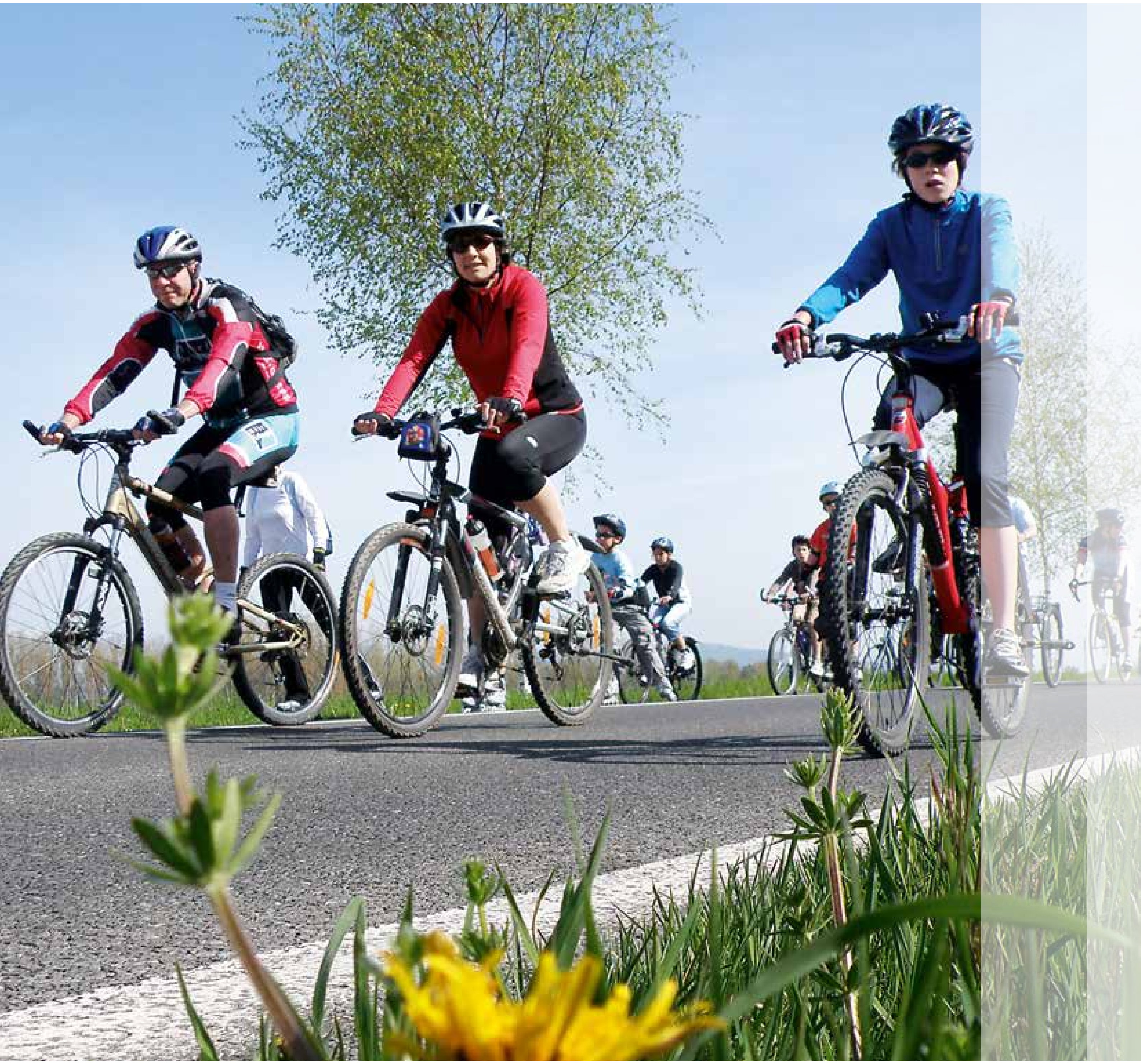

swiss olympic
for the spirit of sport

bpa

suvaliv
Vos loisirs en sécurité

Schweizerische Eidgenossenschaft
Confédération suisse
Confederazione Svizzera
Confederaziun svizra

Département fédéral de l'intérieur DFI
Office fédéral de la statistique OFS

Schweizerische Eidgenossenschaft
Confédération suisse
Confederazione Svizzera
Confederaziun svizra

Office fédéral du sport OFSPO

Sport Suisse 2014

Activité et consommation sportives de la population suisse

Markus Lamprecht, Adrian Fischer, Hanspeter Stamm

2014

Observatoire Sport et activité physique Suisse
c/o Lamprecht & Stamm Sozialforschung und Beratung AG

Office fédéral du sport OFSPO
bpa – Bureau de prévention des accidents
Suva
Swiss Olympic
Office fédéral de la statistique OFS

Référence proposée: Lamprecht, M., Fischer, A. & Stamm, H. P. (2014): Sport Suisse 2014: Activité et consommation sportives de la population suisse. Macolin: Office fédéral du sport OFSPO.

Table des matières

L'essentiel en bref 4

1. Introduction 6

2. Évolution de l'activité sportive 7

3. Comportement en matière d'activité sportive et physique 9

4. Différences selon l'âge et le sexe 11

5. Motivations et conception du sport 15

6. Sports et désirs 18

7. Différences régionales 22

8. Différences sociales 26

9. Les non-sportifs 30

10. Lieux et horaires de pratique sportive 33

11. Sport en club 35

12. Sport dans les centres de fitness 39

13. Sport et vacances 40

14. Blessures et accidents liés au sport 42

15. Intérêt porté aux sports médiatiques 44

16. Dépenses liées au sport 48

17. Encouragement du sport et défis à relever 50

18. Méthode de recherche et sondage 55

L’essentiel en bref

Les résultats présentés ici concernent la population résidante suisse âgée de 15 à 74 ans. Les points suivants correspondent aux principales analyses de l’étude. Nous avons choisi d’en récapituler les résultats clés:

- On assiste à une augmentation de l’activité sportive parmi la population suisse, avec un nombre sans cesse croissant de personnes qui pratiquent un sport de façon intensive. A contrario, la part des non-sportifs reste stable tandis que celle des sportifs occasionnels tend à disparaître. Soit la population pratique un sport, soit elle n’en fait pas du tout.
- Le sport favorise une bonne santé et une vie active. Les quatre cinquièmes de la population satisfont aux toutes dernières recommandations en matière d’activité physique, le rôle du sport en la matière étant fondamental. Une comparaison internationale fait apparaître que les Suisses sont très actifs.
- Les femmes font pratiquement autant de sport que les hommes. On relève toutefois des différences d’une classe d’âge à l’autre: les hommes jeunes sont particulièrement actifs. Avec l’âge, l’activité sportive ne cesse de s’éroder et augmente de nouveau à la retraite. Chez les femmes, on relève des creux d’activité plus fréquents. Aujourd’hui, on pratique toutefois beaucoup de sport tout au long de la vie. L’activité sportive des seniors a ainsi encore sensiblement progressé au cours des dernières années.
- Les motivations de la pratique d’un sport sont extrêmement variées. La proximité de la nature, la santé, le plaisir, le bien-être procuré par l’activité physique et le besoin de déconnexion sont des facteurs importants pour la quasi-totalité des sportifs. La convivialité et la poursuite d’objectifs de performance personnels sont davantage citées par les jeunes et perdent de l’importance avec l’âge. Environ un quart des sportifs participe à des compétitions et à des événements sportifs. Il s’agit généralement de personnes qui pratiquent un sport de façon intensive.
- L’offre sportive est de plus en plus diversifiée, et la population a tendance à faire plusieurs sports à la fois. Au total, plus de 250 sports ont été recensés. Les sports pouvant être pratiqués tout au long de la vie tels que la randonnée pédestre, le cyclisme, la natation et le ski sont particulièrement prisés et ont gagné en popularité depuis quelques années. Le choix d’un sport est fortement lié à l’âge et au sexe, mais aussi à la région, aux revenus du ménage et à la nationalité.
- On n’observe en revanche plus aucune différence selon que les pratiquants habitent en ville ou à la campagne. On relève toutefois des disparités d’une région linguistique à l’autre. Les Suisses alémaniques sont ainsi plus sportifs que les Suisses italophones et romands. Néanmoins, contrairement à la Suisse italophone, la Suisse romande s’est bien rattrapée ces dernières années. En Suisse romande, le boom du sport a touché les femmes et les seniors, qui se tournent de plus en plus vers des activités pouvant être pratiquées tout au long de la vie.
- Le comportement sportif varie selon le statut social, la catégorie socioprofessionnelle et la situation personnelle de chacun. Il est déterminé principalement par le niveau de revenu et la nationalité. Les personnes avec des revenus inférieurs à la moyenne ainsi que la population de nationalité étrangère font beaucoup moins de sport. Contrairement à l’évolution observée parmi les Suisses de souche, on observe encore des différences importantes entre les sexes au sein de la population immigrée, qui compte un grand nombre d’inactifs.
- Un bon quart de la population déclare ne pratiquer aucun sport, sans toutefois y être totalement hostile pour une majorité de personnes interrogées. Environ trois quarts de non sportifs ont fait du sport avec plaisir par le passé. Les deux tiers souhaiteraient même reprendre une activité sportive s’ils avaient plus de temps ou moins d’obligations professionnelles et familiales. Une majorité de non-sportifs a une opinion positive du sport et n’est pas inactive de manière générale. Les deux tiers d’entre eux remplissent même les exigences minimales relatives à une promotion de la santé par l’activité physique.
- Les sportifs disposent d’infrastructures variées dans leur commune ou à proximité de leur domicile et les utilisent activement. Les chemins de randonnée et les piscines ainsi que les salles de sport jouent ici un rôle clé.
- Un quart de la population est membre d’un club de sport. Ce sont surtout des hommes jeunes, mais les seniors aussi pratiquent encore beaucoup de sport en club. La camaraderie, la convivialité, une pratique régulière et la qualité des entraîneurs sont les principaux avantages cités en faveur des clubs sportifs. Quelque 7 % de la population s’engagent dans un club à titre bénévole. En moyenne, le bénévolat représente une activité de 2,5 heures par semaine, généralement non rémunérée, mais très épanouissante et satisfaisante. En dehors des clubs, plus d’un cinquième de la population suisse s’engage volontairement et bénévolement en faveur du sport.
- Un sixième de la population se rend régulièrement dans un centre de fitness privé. Une grande flexibilité, l’absence de contraintes et des horaires d’ouverture étendus comptent parmi les principales motivations de l’affiliation à un centre de fitness. Contrairement aux clubs sportifs, plus souvent fréquentés par des hommes, les centres de fitness et les diverses offres proposées accueillent une majorité de femmes.

- Les deux cinquièmes de la population ont passé des vacances dans lesquelles le sport jouait un rôle majeur au cours des 12 derniers mois. Ces vacances se sont déroulées en Suisse plutôt qu'à l'étranger. Dans ce dernier cas, elles étaient toutefois plus longues. Les vacances sportives restent majoritairement consacrées au ski et à la randonnée pédestre.
- Quelque 8 % de la population se blessent chaque année en faisant du sport. Une grande majorité des blessures liées au sport sont légères et peuvent être traitées par des soins ambulatoires. Les hommes sont davantage victimes d'accidents de sport que les femmes. Les accidents de ski et de football sont les plus fréquents.
- Les quatre cinquièmes de la population environ suivent l'actualité sportive dans les médias. Les sports médiatisés suscitent l'intérêt de catégories de population très variées. La télévision et les journaux restent les principaux canaux d'information, même si Internet joue évidemment un rôle de plus en plus important ces dernières années. Le football, le ski alpin, le tennis et le hockey sur glace sont les sports les plus populaires.
- Les Suisses consacrent un budget de 2500 francs par an et par tête au sport. Un quart de cet argent est dépensé à l'étranger. Les hommes déboursent plus que les femmes pour le sport. L'essentiel de ces dépenses est dédié aux voyages et aux équipements sportifs ainsi qu'à l'habillement.
- Les Suisses estiment clairement que le sport doit être encouragé dans une large mesure. De nombreuses voix se prononcent notamment en faveur d'un renforcement de la promotion de la relève et du sport auprès des jeunes, mais aussi des handicapés, des femmes et des seniors, sans oublier le sport populaire et le sport d'élite professionnel. Cette large adhésion au sport s'explique par la conviction qu'il s'agit d'un facteur clé de socialisation et d'intégration, mais aussi de prévention et de promotion de la santé. Les zones d'ombre citées sont le dopage, la commercialisation, la recherche excessive de la performance et du risque ainsi que le hooliganisme et la corruption. Des mesures fortes sont réclamées pour lutter contre ces fléaux.

1. Introduction

L'enquête «Sport Suisse», menée pour la troisième fois après les éditions de 2000 et de 2008, est un instrument d'observation majeur du sport en Suisse. L'objectif principal de l'étude est de dresser un état des lieux détaillé du sport et de son évolution dans le temps. La constitution d'une base de données fiable sur le comportement et les besoins sportifs de la population suisse ainsi que sur l'intérêt manifesté pour le sport donne des clés de lecture scientifique essentielles pour la planification et les décisions politiques en la matière.

L'enquête a été coordonnée et financée par l'Office fédéral du sport conjointement avec Swiss Olympic, le Bureau de prévention des accidents (bpa), la Suva et l'Office fédéral de la statistique. Ont également participé à l'enquête Suisse Rando, SuisseMobile et Antidoping Suisse. L'Observatoire Sport et activité physique Suisse (c/o Lamprecht & Stamm, Sozialforschung und Beratung) a assuré la planification, l'accompagnement scientifique, l'analyse des résultats et la rédaction du rapport en étroite collaboration avec ses soutiens et ses partenaires. L'institut LINK a réalisé un sondage par téléphone auprès d'un échantillon représentatif de la population, suivi d'une enquête en ligne (cf. chapitre 18).

Les cantons d'Argovie, de Bâle-Campagne, de Genève, des Grisons, de Saint-Gall et de Zurich ainsi que les villes de Saint-Gall, Winterthour et Zurich ont participé au projet via des enquêtes régionales complémentaires. L'objectif de ces études approfondies est de mettre en évidence les spécificités régionales et les problèmes particuliers en matière de comportements sportifs, et de les comparer avec les données recueillies pour l'ensemble de la Suisse.

Le présent rapport de base présente les principaux résultats de l'enquête menée auprès de la population résidante suisse âgée de 15 à 74 ans. Plusieurs rapports détaillés suivront au cours des mois à venir. Au niveau national, un rapport consacré aux enfants et aux jeunes sera en outre publié. Un rapport accompagné de fiches d'information sur les différents sports ainsi qu'une étude sur la consommation sportive doivent également paraître. Des rapports spéciaux sur tous les comptes rendus cantonaux et communaux ainsi que sur ceux de Suisse Rando, SuisseMobile et Antidoping Suisse sont par ailleurs prévus.

Nous profitons de l'occasion pour remercier chaleureusement tous les soutiens et les partenaires de l'initiative Sport Suisse pour leur formidable engagement et leur précieuse collaboration. Nous souhaitons adresser des remerciements particuliers aux 10652 adolescents et adultes ainsi qu'aux 1525 enfants qui ont accepté de donner des informations détaillées sur leurs comportements et leurs besoins en matière de sport ainsi que sur leur consommation sportive.

2. Évolution de l'activité sportive

Le boom du sport se poursuit

La population suisse est très sportive: 44 % des personnes âgées de 15 à 74 ans déclarent pratiquer un sport plusieurs fois par semaine, à raison d'un minimum de trois heures d'exercice (cf. graphique 2.1). Quelque 25 % font du sport au moins une fois par semaine tandis que 5 % déclarent pratiquer une activité sportive de manière occasionnelle ou rarement. Les 26 % restants se décrivent comme des non-sportifs.

Le graphique 2.1 montre aussi que l'activité sportive de la population suisse a fortement progressé depuis 2000. La part des sportifs très actifs n'a en effet cessé de croître, passant de 36 % en 2000 à 44 % en 2014. Tandis que la part des non-sportifs est restée stable, celle des sportifs occasionnels a diminué de moitié (recul de 10 à 5 %).

Les derniers chiffres semblent confirmer la tendance à long terme (cf. graphique 2.2). Depuis 20 ans, la part des personnes qui pratiquent un sport plusieurs fois par semaine augmente tandis que celle des sportifs occasionnels est en net recul. En revanche, la proportion de non sportifs est stable et se situe à un niveau équivalent à celui de la fin des années 1970. On distingue donc aujourd'hui principalement deux catégories de personnes: celles qui pratiquent assidûment un sport et celles qui n'en font pas du tout. Le boom du sport a atteint son point culminant au milieu des années 1990, la courbe tendant actuellement à s'aplanir. Cela s'explique notamment par le fait que les sportifs occasionnels susceptibles de devenir des sportifs réguliers se font de plus en plus rares. En outre, la part des non-sportifs est extraordinairement stable et n'a pas baissé, en dépit du boom du sport.

G 2.1: Activité sportive en fonction de la fréquence et de la durée en 2000, en 2008 et en 2014 (en % de la population résidante suisse âgée de 15 à 74 ans)

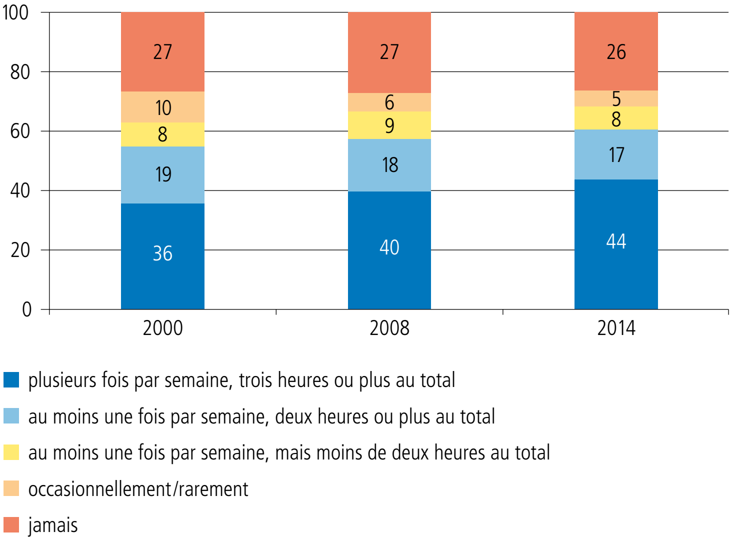

Remarque: nombre de personnes interrogées: 2000: 2058, 2008: 10 242, 2014: 10 622. Par la suite, nous appellerons «sportifs très actifs» toutes les personnes qui font du sport plusieurs fois par semaine, à raison de trois heures ou plus. Les autres personnes qui déclarent pratiquer un sport, quel qu'il soit et sous quelque forme que ce soit, seront désignées comme des «sportifs». Enfin, les «non-sportifs» englobent les personnes qui affirment n'avoir aucune activité sportive.

G 2.2: Évolution de l'activité sportive en Suisse entre 1978 et 2014 (en %)

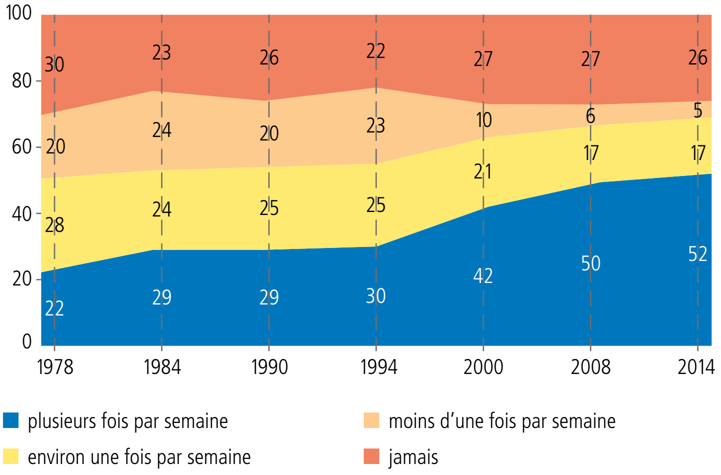

Remarque: les chiffres préalables à la création de l'initiative Sport Suisse en 2000 s'appuient sur des sondages menés auprès de la population par Swiss Olympic. Dans les éditions antérieures, la différence entre «(quasi) quotidiennement» et «plusieurs fois par semaine» n'existait pas. De plus, alors que les premiers sondages étaient effectués auprès d'un échantillon de 1000 personnes au plus, 2064 personnes ont été interrogées en 2000, 10 262 en 2008 et 10 652 en 2014.

Comparaison internationale

Au niveau international, la Suisse est une nation extrêmement sportive. En Europe, seuls les pays scandinaves comptent davantage de sportifs réguliers que la Suisse. En ce qui concerne les non-sportifs, le bilan n'est pas aussi positif: là aussi, la Suisse reste bien placée, en milieu de peloton, avec l'Irlande, les Pays-Bas, la Belgique, la Slovénie ou l'Autriche (cf. graphique 2.3).

G 2.3: Activité sportive en Europe, comparaison avec les données d'Eurobaromètre (en %)

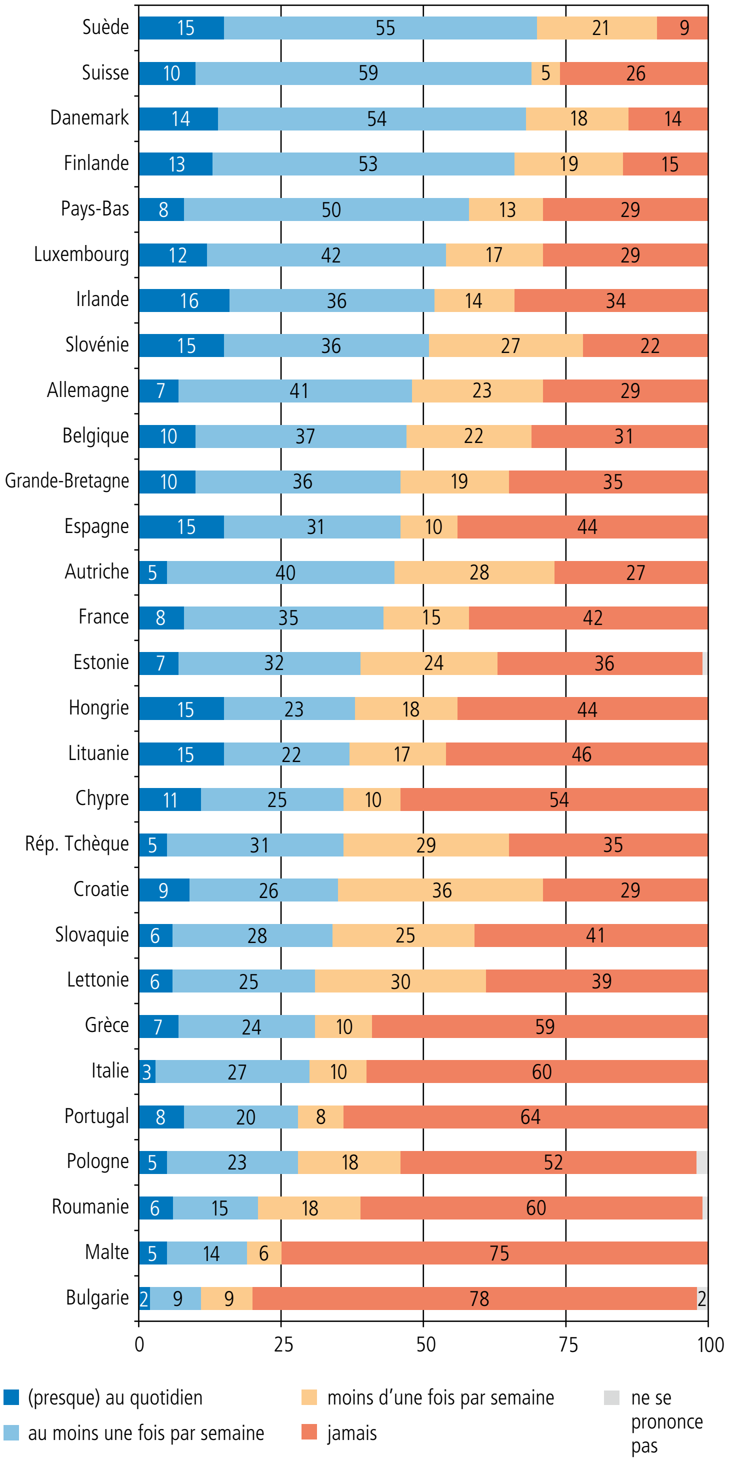

Remarque: source des données relatives aux pays européens: Eurobaromètre Spécial 334 (2010), «Sport et activités physiques», Commission européenne.

3. Comportement en matière d'activité sportive et physique

Élargissement de la notion de «sport»

Établir si la population d'un pays est plus ou moins sportive et déterminer l'évolution de l'activité sportive au fil du temps est une gageure. Cela dépend en effet de ce qu'on entend exactement par «sport». Combien de longueurs de bassin faut-il effectuer pour qu'une simple baignade devienne de la natation? Que faut-il pour que la pratique du vélo devienne un sport: une roue en carbone très chère, une vitesse donnée ou une tenue vestimentaire particulière? Comment faire la distinction entre une simple promenade et le walking, et qu'est-ce qui différencie le walking de la randonnée pédestre? Les échecs sont-ils considérés comme un sport? Qu'en est-il de l'agility, de la luge ou de la pêche?

Avec le développement du sport au cours des 20 dernières années, la notion de sport a elle aussi évolué et s'est élargie. De nouveaux besoins ainsi que de nouvelles motivations, offres et pratiques sont venus enrichir le sport. Le concept même de sport est donc plus vaste et varié, mais aussi plus opaque et diffus. De nombreux facteurs déterminent aujourd'hui ce qu'est le sport et ce qu'il n'est pas, parmi lesquels la qualité et l'intensité de l'activité, ses motivations, les objectifs poursuivis et les normes qui la régissent, mais aussi le lieu et l'environnement. Les critères cités entrent dans la définition courante donnée en sciences du sport: une activité motrice caractérisée par ses valeurs propres et régie par des normes sociales.

A cet égard, il faut préciser que l'élargissement du terme a fortement contribué au boom du sport: d'une part, l'apparition de nouvelles activités a attiré de nouvelles catégories de consommateurs. D'autre part, des activités existantes ont été requalifiées en sports alors qu'elles n'étaient pas encore considérées comme tels il y a 20 ans. Autrement dit: le boom du sport s'explique par l'arrivée de nouvelles catégories de consommateurs, mais aussi par une certaine dilution du terme.

Comment mesure-t-on l'ampleur du sport?

Dans le cadre de la présente étude, en accord avec la communauté internationale de la recherche, il a été sciemment laissé à l'appréciation des personnes interrogées le soin de définir ce qu'il faut entendre par sport. Dans un deuxième temps seulement, il leur a été demandé de préciser les activités classées parmi les sports ainsi que les motivations et les objectifs poursuivis dans ce cadre (cf. chapitres 5 et 6).

L'indicateur évoqué en introduction, qui vise à mesurer l'ampleur de l'activité sportive, repose sur deux questions simples: A quelle fréquence faites-vous du sport? Combien d'heures par semaine cela représente-t-il? Les réponses obtenues à ces deux questions sont regroupées dans le tableau 3.1. On constate que la fréquence de la pratique sportive a plus fortement progressé depuis 15 ans que la durée de l'activité en question. La proportion de personnes qui pratiquent un sport plusieurs fois par semaine a sensiblement augmenté entre 2000 et 2014, passant de 42 % à 52 %. La part des personnes qui font du sport trois heures ou plus chaque semaine a quant à elle progressé de 46 % à 50 %.

T 3.1: Profil des sportifs en fonction de la fréquence et de la durée de leur activité sportive en 2000, en 2008 et en 2014 (en % de la population résidante suisse âgée de 15 à 74 ans)

		2000	2008	2014
Fréquence de l'activité sportive	jamais	27	27	26
	occasionnellement/rarement	10	6	5
	environ une fois par semaine	21	17	17
	plusieurs fois par semaine	30	38	42
	(quasi) quotidiennement	12	12	10
Nombre d'heures par semaine	jamais	27	27	26
	moins de deux heures	12	12	10
	deux heures	15	15	14
	trois à quatre heures	20	22	24
	cinq à six heures	12	13	12
	sept heures et plus	14	13	14
Nombre de personnes interrogées		2058	10242	10616

T 3.2: Activité sportive: modèle d'activité en fonction de la fréquence et de la durée (en % de la population résidante suisse âgée de 15 à 74 ans)

	jamais	moins de 2 heures	2 heures	3 à 4 heures	5 à 6 heures	7 heures et plus	Total
jamais	26						26
occasionnellement/ rarement		3	1	1	0	0	5
une fois par semaine maximum		6	7	3	1	0	17
plusieurs fois par semaine		1	6	18	9	8	42
(quasi) quotidiennement		0	0	2	2	6	10
Total	26	10	14	24	12	14	100

Remarque: nombre de personnes interrogées: 10613. Les couleurs correspondent aux catégories du graphique 2.1.

Le tableau 3.2 permet de relier les paramètres de fréquence et de durée. Il permet en outre d'appréhender le principe de l'indicateur de l'ampleur du sport. En toute logique, la durée totale augmente avec la fréquence de l'activité. Certaines personnes pratiquent toutefois un sport plusieurs fois par semaine en suivant un programme de gymnastique ou de renforcement musculaire sans atteindre une durée totale d'activité de deux heures par semaine. On recense aussi des personnes qui font du sport occasionnellement, mais suffisamment longtemps (randonnée à pied ou à vélo, p. ex.) pour totaliser une durée d'activité moyenne de trois à quatre heures par semaine.

La relation entre la fréquence et la durée de la pratique sportive est restée stable entre 2008 et 2014. Le tableau 3.2 permet de différencier les sportifs très actifs et montre qu'un quart de la population pratique un sport plusieurs fois par semaine, à raison de plus de cinq heures d'activité totale. Quelque 5 % de la population font même du sport quasi quotidiennement à raison de plus de six heures hebdomadaires.

La pratique régulière d'un sport permet de respecter les recommandations en matière d'activité physique

De nombreuses activités physiques ne sont pas considérées comme des sports. Le sport joue néanmoins un rôle central dans la promotion de l'activité physique, comme le montre le graphique 3.1. L'enquête Sport Suisse s'intéresse non seulement au sport, mais également aux activités physiques quotidiennes réalisées au travail ou pendant les loisirs qui font transpirer ou, du moins, provoquent un certain essoufflement. Les réponses fournies ont été comparées avec les recommandations en matière d'activité physique formulées par l'Office fédéral du sport, l'Office fédéral de la santé publique, Promotion Santé Suisse, la Suva, le bpa et le réseau HEPA. Les toutes dernières recommandations préconisent un minimum de deux heures et demie ou 75 minutes d'activité intensive par semaine – un total respecté par 83 % de la population. Seulement 8 % des sondés ont une activité physique partielle, 9 % se disant pour ainsi dire inactifs.

G 3.1: Relation entre activité sportive et respect des recommandations en matière d'activité physique (en %)

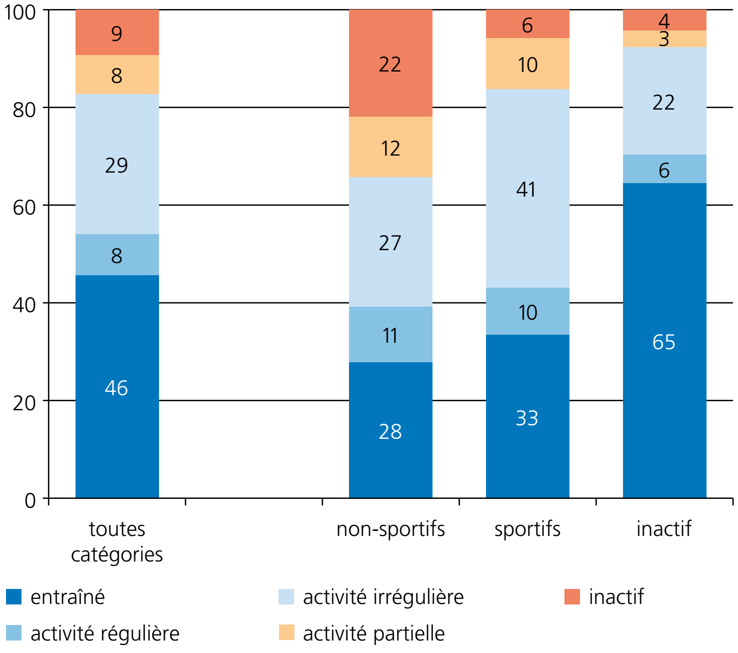

Remarque: nombre de personnes interrogées: 10 429. Catégories d'activité sportive: Non-sportifs: aucune activité sportive; Sportifs très actifs: plusieurs fois par semaine, 3 heures ou plus au total; Sportifs: toutes les autres personnes qui pratiquent un sport. Catégories de comportements en matière d'activité physique: entraîné: min. 3 jours de transpiration (activités intensives) par tranches de 30 minutes min.; activité régulière: min. 5 jours par semaine d'une activité physique modérée (essoufflement) par tranches de 30 minutes min.; activité irrégulière: activité modérée > 150 minutes ou intensive > 75 minutes ou 2 jours par semaine act. intensive + modérée > 150 minutes; activité partielle: activité modérée < 150 minutes ou intensive < 75 minutes; inactif: aucune activité. Consulter également l'indicateur «Promotion du sport et de l'activité physique» sur www.sportobs.ch.

L'exercice physique est naturellement étroitement lié au volume d'activité sportive. Une personne qui fait beaucoup de sport satisfait généralement aux recommandations en matière d'activité physique. La condition à remplir est de pratiquer une activité régulière à raison d'au moins 30 minutes par séance. Le fait que la catégorie des sportifs très actifs englobe des personnes qui ne respectent pas les recommandations en matière d'activité physique souligne qu'il existe des sports qui ne provoquent ni essoufflement ni transpiration. A l'inverse, une proportion significative de non-sportifs satisfait aux recommandations en matière d'activité physique. Cela démontre qu'on peut transpirer ou être essoufflé au travail ou pendant ses loisirs, même sans faire de sport. Le chapitre 9 met en évidence le fait que plusieurs activités physiques pratiquées par des non-sportifs peuvent être assimilées à des activités sportives.

4. Différences selon l'âge et le sexe

Les femmes font aujourd'hui autant de sport que les hommes

Le champ des consommateurs s'est étendu avec le boom du sport et l'élargissement de la définition du sport. L'apparition de nouveaux sports et de nouvelles offres en matière d'activité physique a attiré de nouvelles catégories d'utilisateurs. Si le sport était encore l'apanage des hommes jeunes il y a une trentaine d'années, les seniors et les femmes sont aujourd'hui des sportifs tout aussi enthousiastes. Les graphiques suivants mettent en évidence et décrivent les différences éventuelles en matière d'activité sportive liées à l'âge et au sexe.

Dans un premier temps, le graphique 4.1 indique que la proportion de non-sportifs est équivalente chez les femmes et les hommes, et stable depuis une quinzaine d'années. Par contre, on relève une augmentation des sportifs très actifs, la tendance étant plus marquée chez les femmes que chez les hommes sur la période examinée. Cette catégorie est un peu moins importante chez les femmes que chez les hommes. On compte également légèrement plus de femmes ayant une activité modérée. Les écarts sont toutefois minimes depuis quelques années.

G 4.1: Activité sportive en fonction du sexe, 2000, 2008, 2014 (en %)

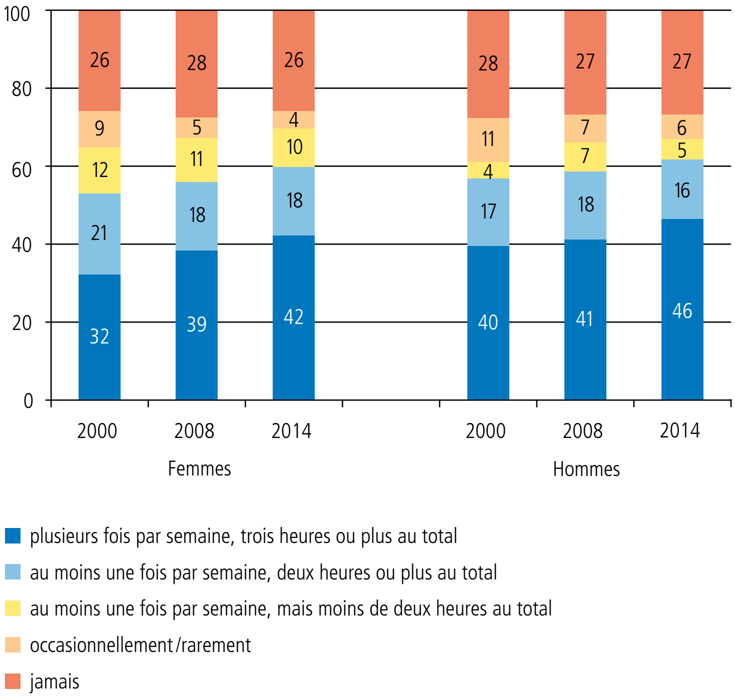

Remarque: nombre de personnes interrogées: 2000: 2058; 2008: 10 242; 2014: 10 622

Augmentation de l'activité sportive chez les plus de 65 ans

Les différences liées à l'âge se sont elles aussi réduites (cf. graphique 4.2): par rapport à 2008, on constate une légère augmentation de l'activité sportive dans toutes les classes d'âge. Les seniors enregistrent toutefois une progression supérieure à la moyenne. Les plus jeunes (15-24 ans) comprennent toujours le plus grand nombre de sportifs très actifs et la plus faible proportion d'inactifs. Le sport n'est cependant définitivement plus l'apanage des jeunes. Les seniors sont désormais des sportifs accomplis. Ainsi, on ne relève plus qu'une faible baisse de l'activité sportive chez les personnes d'âge moyen et mûr. La part des sportifs très actifs n'a en effet cessé de croître parmi les seniors, passant de 34 % en 2008 à 42 % aujourd'hui. Cette croissance s'explique avant tout par l'évolution observée chez les femmes seniors, parmi lesquelles la proportion de sportives très actives est passée de 30 à 41 %, contre une hausse de 39 à 44 % chez les hommes de 65 à 74 ans.

G 4.2: Activité sportive en fonction de l'âge, 2008 et 2014 (en %)

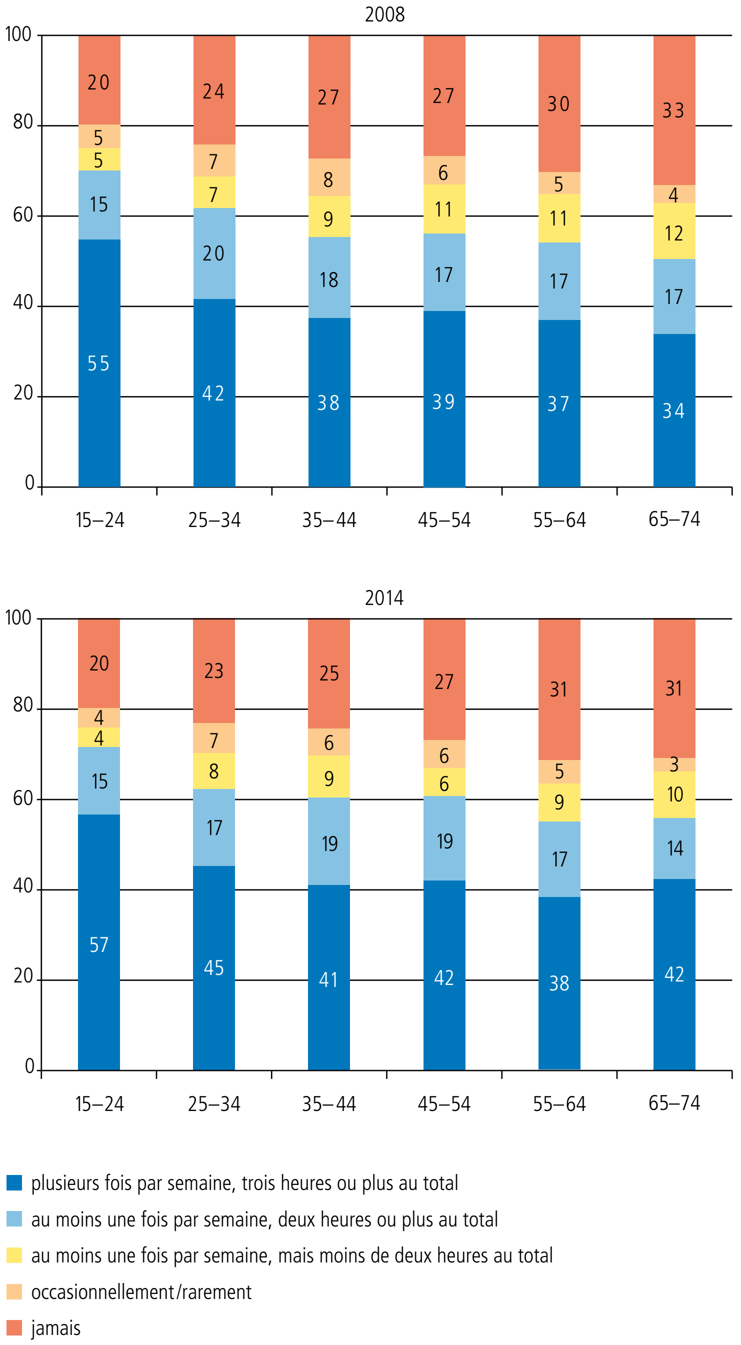

Remarque: nombre de personnes interrogées: 2008: 10 242; 2014: 10 622

Différences liées au sexe en termes de biographie sportive

Il est intéressant de souligner que l'évolution de l'activité sportive au long de la vie n'est pas la même chez les hommes et les femmes (cf. graphique 4.3). Après 24 ans, elle recule constamment chez les hommes avant d'augmenter de nouveau à partir de 65 ans. Le temps gagné avec le départ à la retraite est également investi en activités sportives. Les femmes jeunes sont un peu moins sportives que les hommes. De plus, elles font beaucoup moins de sport que leurs homologues masculins après 25 ans. Leur activité sportive augmente cependant de nouveau dès l'âge de 40 ans, soit plus tôt que les hommes. Qui plus est, elle est nettement supérieure à celle des hommes entre 45 et 64 ans. On n'observe toutefois pas de recrudescence de l'activité sportive à l'âge de la retraite chez les femmes.

Les chiffres relatifs à l'activité sportive reflètent les différences liées au sexe en termes d'évolution personnelle et professionnelle. Les femmes ont tendance à réduire leurs activités sportives pour élever des enfants et se consacrer à leur vie familiale. Elles refont plus de sport quand leurs enfants sont grands. L'activité sportive baisse également chez les jeunes pères, mais pas dans les mêmes proportions que chez les mères. Parmi les femmes qui ont des enfants de moins de six ans, on compte 35 % d'inactives (contre 27 % pour les jeunes papas) et seulement 33 % de sportives très actives (contre 36 % chez les hommes). Les écarts entre les parents de sexes différents s'effacent dès que les enfants atteignent l'âge de six ans (cf. graphique 8.1).

G 4.3: Activité sportive en fonction du sexe et de l'âge en 2014 (en %)

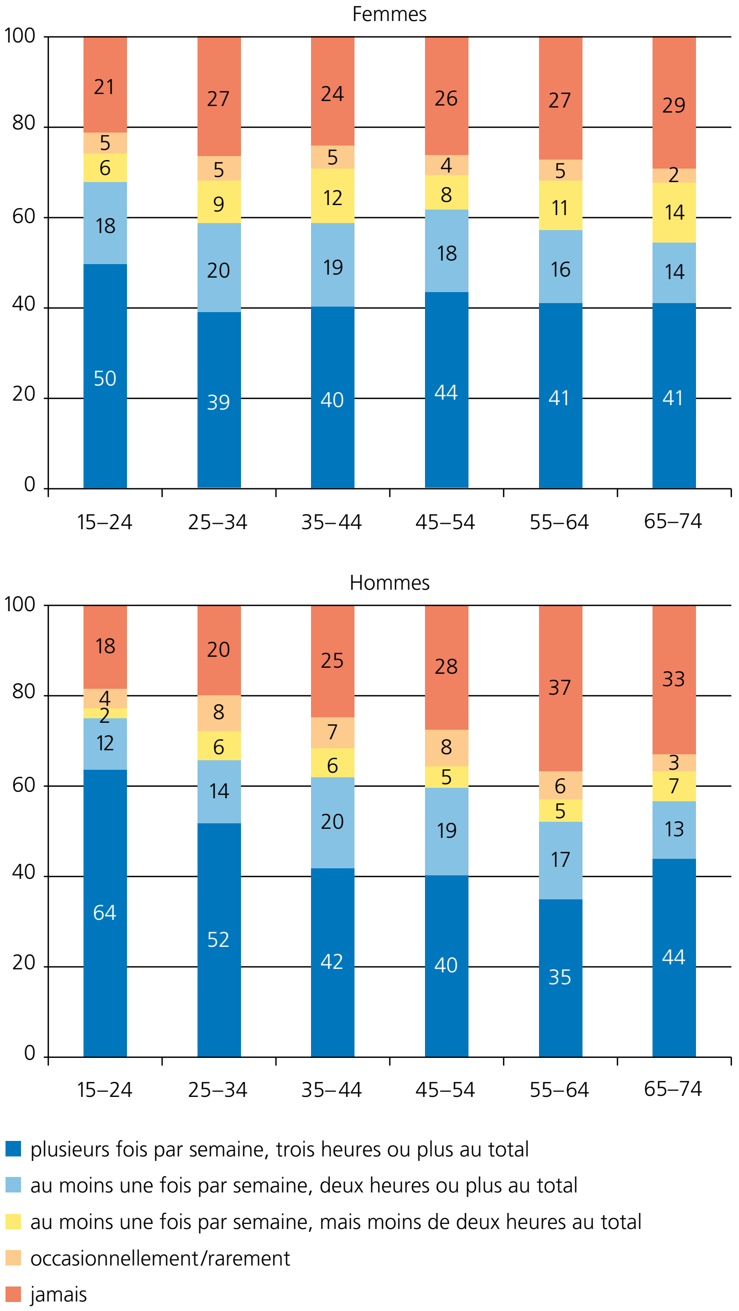

Remarque: nombre de personnes interrogées: 10 622

L’enthousiasme pour le sport comme garant de la pratique d’une activité sportive tout au long de la vie

Ce schéma se confirme lorsqu’on interroge les sondés sur leur passé de sportif (cf. graphique 4.4). Plus de 40 % des hommes âgés de 45 à 54 ans déclarent avoir pratiqué plus de sport par le passé. Seul un cinquième d’entre eux réussit à maintenir son engagement sportif dans la durée. Ceux qui réussissent à le renforcer sont rares. Les femmes ont les mêmes difficultés à maintenir leur engagement sportif au long de la vie, mais le font plus souvent évoluer que les hommes en fonction de la phase dans laquelle elles se trouvent. De plus, elles parviennent mieux à se remobiliser entre 45 et 64 ans.

Eu égard à l’encouragement du sport et de l’activité physique, il faut par ailleurs préciser que les personnes qui déclarent pratiquer un sport depuis toujours et être d’une nature sportive sont très actives en la matière. Leur enthousiasme pour le sport explique que ce dernier les accompagne au cours des différentes phases de leur vie.

G 4.4: Évolution de l’activité sportive au cours de la vie en fonction du sexe et de l’âge (en %)

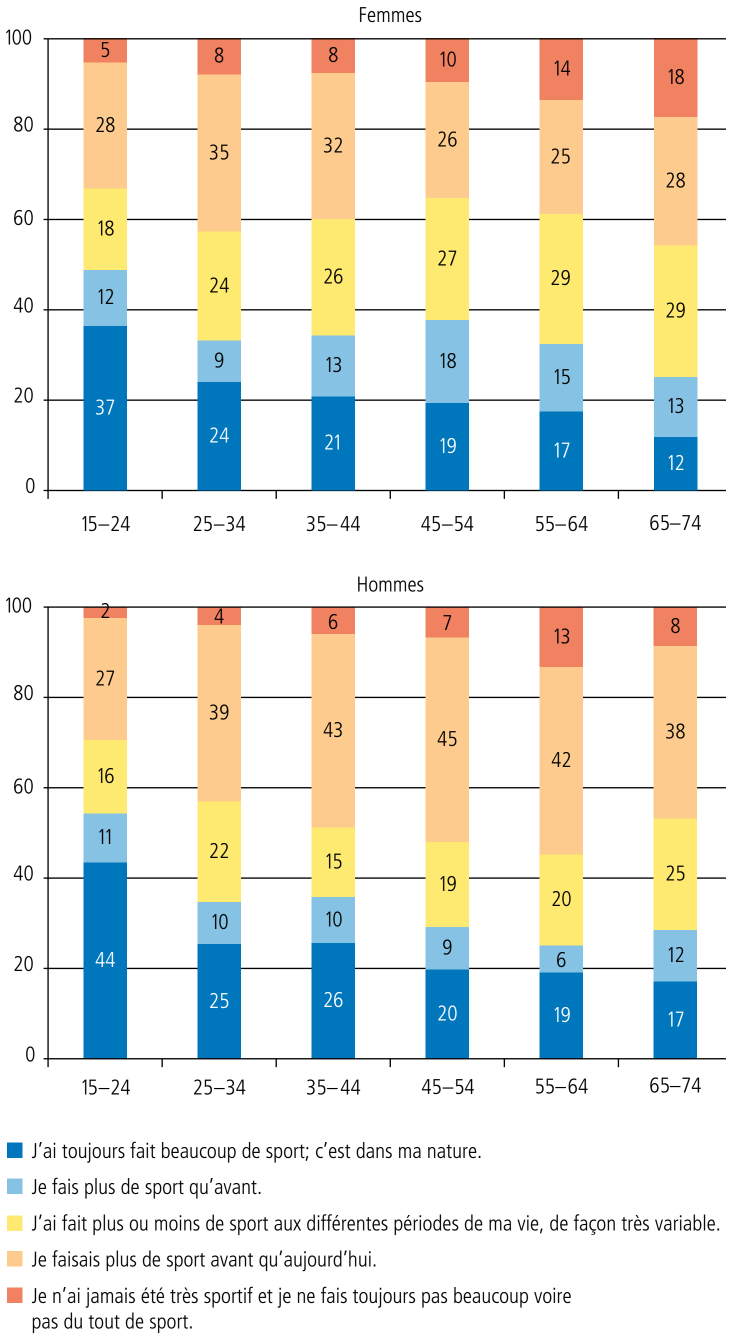

Remarque: nombre de personnes interrogées: 6704 (enquête en ligne).
Question posée: «Avez-vous fait beaucoup de sport par le passé? Merci de choisir la réponse la plus adaptée à votre cas parmi les cinq propositions suivantes.»

5. Motivations et conception du sport

La santé et le plaisir plus souvent cités que la recherche de la performance et l'esprit de compétition

L'élargissement de la notion de sport et l'ouverture de la pratique sportive vont de pair avec une conception plus vaste du sujet. L'apparition de nouveaux sports et de nouvelles formes d'activité physique, pratiqués par de nouvelles catégories de consommateurs, avec des besoins redéfinis et une conception inédite du sport, a déclenché une dynamique forte. Cette dynamique a enrichi et diversifié le sport, mais l'a aussi rendu plus opaque, au détriment de son homogénéité. Les motivations classiques de la pratique d'un sport, comme la recherche de la performance et l'esprit de compétition, sont actuellement supplantées et complétées par beaucoup d'autres facteurs.

Le graphique 5.1 donne un aperçu des raisons qui poussent la population suisse à faire du sport. La proximité de la nature, la santé, le plaisir ou encore le bien-être procuré par l'activité physique sont des motivations (très) importantes pour la quasi-totalité des sportifs. Le besoin de déconnexion et de détente joue également un rôle déterminant, tout comme la volonté de s'entretenir et d'être en bonne forme physique, de partager des moments de convivialité et de faire des rencontres, sans oublier la quête de moments uniques. Plus de la moitié des sportifs souhaitent par ailleurs améliorer leur pouvoir de séduction en restant minces et en forme. La recherche classique de la performance arrive en fin de liste. Si environ la moitié des sportifs poursuit encore des objectifs de performance personnels, se mesurer aux autres ne motive plus qu'un cinquième d'entre eux.

Ce constat n'est pas nouveau: dès les éditions 2000 et 2008 de l'enquête, la santé, le plaisir ou la camaraderie se classaient devant la performance. Les objectifs de performance personnels et les résultats obtenus jouaient déjà un rôle plus important que la volonté de se mesurer aux autres. Les motivations des sportifs n'ont pas évolué autant depuis 2000 que dans les années 1990, qui marquent le début du boom du sport. Si la volonté de se mesurer aux autres et la convivialité ont perdu du terrain entre 2000 et 2008, l'importance de ces facteurs s'est stabilisée, voire a augmenté depuis six ans. Les aspects liés à la volonté de vivre des moments uniques, d'entretenir sa forme physique et son pouvoir de séduction sont jugés un peu plus importants qu'auparavant.

G 5.1: Importance des différentes motivations du sport (en % de sportifs)

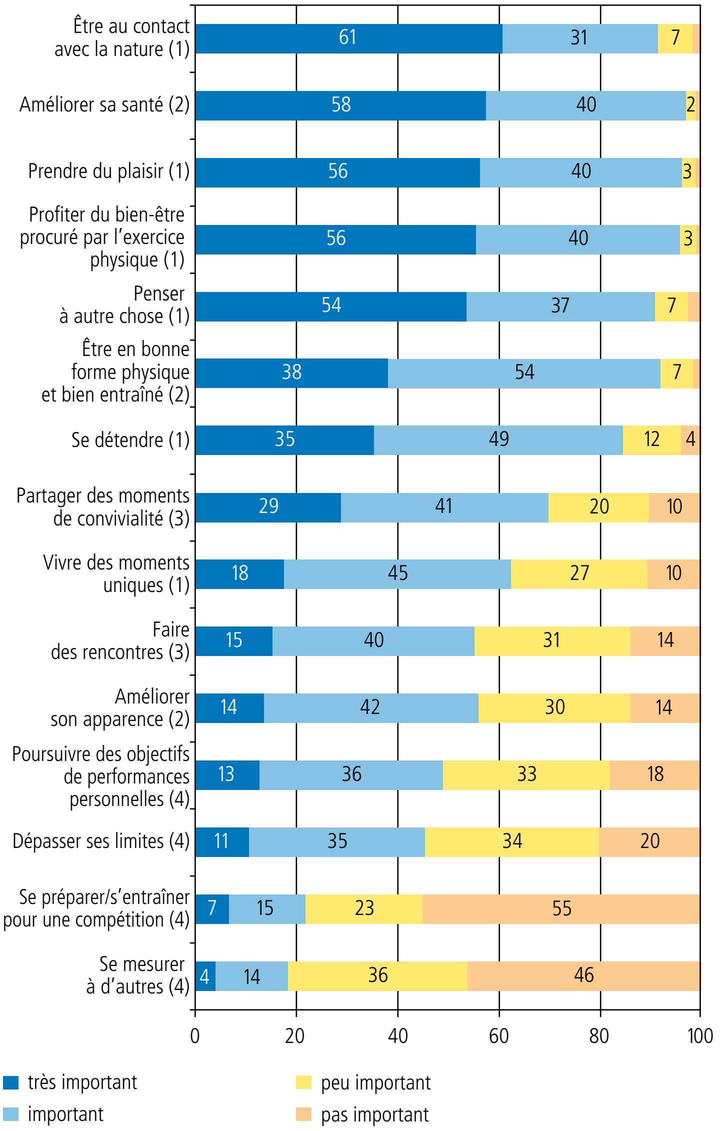

Remarque: nombre de personnes interrogées (sportifs uniquement): entre 7887 (moments uniques) et 7956 (contact avec la nature). Les chiffres entre parenthèses indiquent l'affectation des motivations aux quatre catégories évoquées.

Santé, plaisir, forme physique, détente et performance ne sont pas incompatibles

Il ressort notamment que la relation entre les différentes motivations est presque exclusivement positive. Une motivation n'exclut pas l'autre. Autrement dit, plaisir et performance, santé et esprit de compétition ou encore forme physique et détente ne sont pas incompatibles. Un aspect n'est pas forcément jugé moins important qu'un autre.

Bien au contraire: il existe des combinaisons très variées de motivations, toutes considérées comme majeures. Cela dit, on peut déduire des réponses fournies que certaines motivations sont plus étroitement corrélées. Une analyse factorielle permet ainsi de constituer 4 grandes catégories de motivations: plaisir/besoin de déconnexion/recherche de moments uniques (1), forme physique/santé/apparence (2), convivialité (3) et quête de performance (4) – se reporter aux chiffres entre parenthèses dans le graphique 5.1. L'examen du nombre total de voix obtenues par les quatre catégories confirme qu'une motivation n'exclut pas l'autre et que la quête de performance et de convivialité sont particulièrement proches. A noter également que les motivations varient légèrement en fonction du sexe et de l'âge.

Le graphique 5.2 révèle que les catégories de motivations plaisir/besoin, déconnexion/recherche de moments uniques et forme physique/santé/apparence jouent un rôle majeur pour les femmes comme pour les hommes, toutes classes d'âge confondues. Pour les hommes et les femmes jeunes, la convivialité est un facteur déterminant, puis perd de son importance avant de revenir au premier plan à partir de 55 ans. La recherche de la performance est plus cruciale pour les hommes que pour les femmes, mais perd du terrain avec le temps.

Les sportifs en quête de performance et les compétiteurs sont particulièrement actifs

La pondération de la recherche de la performance varie non seulement selon l'âge et le sexe, mais surtout en fonction de l'activité sportive. Parmi les sportifs très actifs, on compte deux fois plus de personnes (27 %) qui considèrent la recherche de la performance comme un facteur primordial que parmi les autres sportifs (14 %). Il convient donc de relativiser le constat selon lequel cet aspect a perdu du terrain. La quête de performance et, surtout, la volonté de se mesurer aux autres ne sont plus considérées comme des aspects déterminants que par une minorité de sportifs. Ces derniers aiment néanmoins particulièrement le sport et sont très actifs.

L'examen de la participation des sondés à des compétitions et autres événements sportifs confirme cette analyse (cf. tableau 5.1). Un petit quart de sportifs, soit un sixième de la population, participe à des compétitions ou à des événements sportifs. Ce chiffre est légèrement supérieur à celui de

G 5.2: Importance des différentes motivations de la pratique d'un sport en fonction de l'âge et du sexe (en % de sportifs)

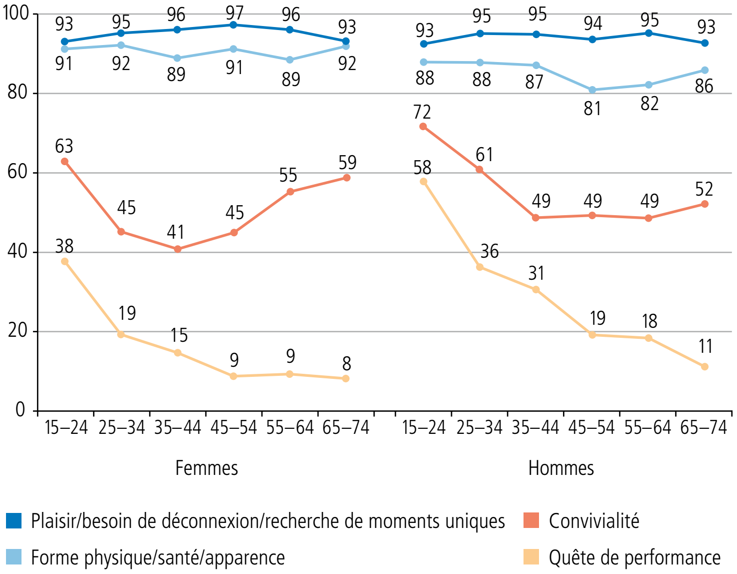

Remarque: nombre de personnes interrogées: 7951 (sportifs uniquement).

T 5.1: Participation à des compétitions et à des manifestations sportives

	en % de sportifs	ø Nbre de compétitions/an	Part des femmes en %	ø Age en nbre d'années	ø Nbre d'heures pratiquées par semaine
Participation à des compétitions/ manifestations sportives	22,9	10,5	37	37	5,7
Aucune participation	77,1	0,0	58	46	4,0
Championnats interclubs (p. ex. championnats de football ou de volleyball)	12,1	15,8	32	34	5,9
Autres compétitions avec classement (p. ex. marathons, triathlons, courses de ski de fond, de vélo ou de VTT)	10,4	6,2	37	39	5,8
Manifestations sportives sans classement (sans chronométrage, traversée d'un lac, SlowUp)	3,1	4,6	64	41	5,1

Remarque: nombre de personnes interrogées: 7964 (sportifs uniquement). Chiffres en pourcentage ou sous forme de moyenne arithmétique. Les personnes interrogées pouvaient citer plusieurs compétitions; le nombre de compétitions par an ne tient compte que des événements entrant dans la catégorie concernée.

2008. A l'époque, les sondés n'ont toutefois été interrogés que sur leur participation à des compétitions sportives. Les autres événements sportifs n'étaient pas pris en compte. Dans leur grande majorité, il s'agit toutefois de compétitions avec classement, championnats interclubs en tête (représentent la majorité des compétitions sportives). Avec une moyenne de six participations par an, les concurrents des autres compétitions avec classement (marathons, triathlons, courses de ski de fond, de vélo ou de VTT, p. ex.) se révèlent également extrêmement actifs. La moyenne élevée obtenue s'explique cependant par le nombre de personnes qui font plus de 20 compétitions par an, soit près de 5 % de tous les participants. Un quart des compétiteurs qui sont membres d'un club se contente d'une manifestation par an, les deux tiers disputant jusqu'à quatre compétitions annuelles. Pour ce qui est des manifestations sportives sans classement, un tiers des participants s'inscrivent à un événement par an, la moitié allant jusqu'à deux.

Les compétiteurs sont principalement des hommes jeunes, qui font environ six heures de sport par semaine. Les championnats interclubs enregistrent la plus faible proportion de femmes de même que l'âge moyen le plus bas. Enfin, on constate que le sport de compétition n'a pas reculé récemment et que les manifestations et les événements sportifs non organisés par des clubs attirent de plus en plus de femmes et de seniors. Il ressort qu'une majorité de femmes s'intéresse aux manifestations sportives sans classement.

Perception positive de la notion de sport

Afin de compléter les informations sur les motivations des sportifs et en apprendre davantage sur leur conception du sport, une liste de 30 termes a été soumise aux participants à l'enquête en ligne. Il leur a été demandé de cocher ceux qu'ils associaient personnellement au sport. Le graphique 5.3 indique le pourcentage de réponses obtenues par chacun des termes. Tandis que la santé (80 %), l'activité physique (79 %) ou le plaisir (71 %) ont été retenus par une grande majorité de participants, un minimum de 50 % de la population associent également le sport aux aspects suivants: forme physique (58 %), entraînement (57 %), connaissance de son corps (53 %), effort/transpiration (52 %), détente (50 %) et camaraderie/convivialité (59 %). Les caractéristiques négatives telles que la contrainte (4 %), l'agressivité/la violence (5 %), la tricherie (6 %), la corruption (8 %) ou le risque (9 %) ont recueilli nettement moins de voix. Pour la majorité des personnes interrogées, le sport a donc une connotation positive. La conception du sport n'a donc pas beaucoup évolué depuis la dernière enquête sur le sujet (édition de 2000). Les aspects négatifs ne sont pas plus fréquemment cités qu'il y a 15 ans. En revanche, les termes suivants ont été cochés beaucoup plus souvent: liberté (+17 %), détente (+12 %), effort/transpiration (+12 %), performance (+11 %), autodiscipline (+10 %) et apparence/beau physique (+9 %). Cela confirme également que même si la notion de sport s'est élargie, la recherche de la performance n'a pas disparu.

Une analyse factorielle permet d'identifier les combinaisons de termes fréquentes, mises en évidence par les couleurs dans le graphique 5.3. Les personnes qui associent le sport au dopage lient ainsi également souvent le terme à la corruption, au business, à la tricherie, à l'agressivité ou aux blessures. De même, lorsque le sport est associé à l'entraînement, il est aussi souvent corrélé avec la forme physique, la performance, la transpiration, l'autodiscipline et un beau physique. Autres combinaisons fréquentes: proximité de la nature, détente, santé, connaissance de son corps, activité physique, plaisir et expérience, d'une part, réalisation de soi, liberté, découverte de ses limites, style de vie et individualisme d'autre part. Les plus jeunes évoquent un peu plus souvent la réalisation de soi, la liberté et le style de vie, mais aussi l'entraînement, la performance et la forme physique. Les seniors retiennent quant à eux davantage les caractéristiques négatives du sport, mais aussi la proximité de la nature, la détente et la santé. Ce dernier aspect est généralement souligné majoritairement par les femmes, les hommes faisant ressortir légèrement plus la camaraderie et le jeu. A noter que les non-sportifs ont une conception du sport assez comparable à celle des sportifs. Le chapitre 9, consacré aux non-sportifs, revient sur cette question.

G 5.3: Termes associés au sport par la population suisse

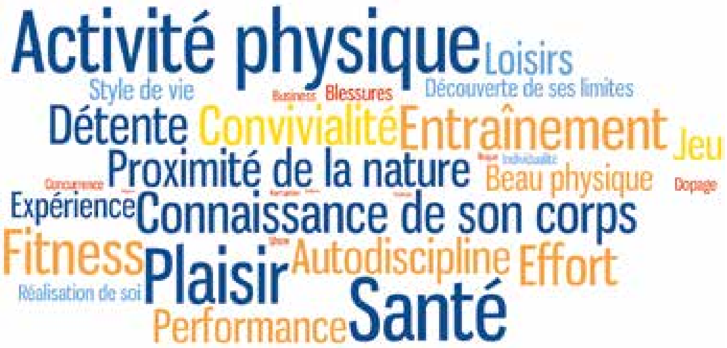

Remarque: nombre de personnes interrogées: 6877 (enquête en ligne). Nuage de mots-clés représentés en fonction de la fréquence de sélection des termes par les personnes interrogées. Les couleurs renvoient à une analyse factorielle des combinaisons de termes fréquentes.

6. Sports et désirs

Une population suisse de plus en plus «polysportive»

Après les questions d'ordre général sur leur activité sportive, les personnes interrogées ont été priées de fournir des informations détaillées sur les sports et les activités physiques qu'elles pratiquent. Les réponses obtenues permettent de dresser un tableau très riche aux multiples facettes du paysage sportif suisse. Quelque 250 sports ont été répertoriés, de l'aérobic à l'agility en passant par le monocycle et l'escrime, sans oublier le «Platzgen» (jeu de palet), le walking et la zumba. Les sportifs interrogés ont cité en moyenne 3,8 sports. Cette valeur est supérieure à celles de 2000 (3,1) et de 2008 (3,3) et montre que la population suisse est désormais encore plus polysportive. Un quart des sportifs pratique au moins 5 sports différents.

Randonnée pédestre, cyclisme, natation et ski: le «combiné helvétique»

La randonnée pédestre, le cyclisme, la natation et le ski arrivent en tête des sports les plus souvent cités et sont pratiqués par plus d'un tiers de la population suisse (cf. tableau 6.1). Depuis l'enquête de 2008, ce «combiné helvétique» est devenu encore plus populaire. Le développement des sports pouvant être pratiqués tout au long de la vie s'explique, d'une part, par une proportion croissante de seniors dans la population ainsi que par l'activité sportive accrue de cette catégorie de consommateurs (cf. chapitre 4). Une analyse plus détaillée révèle en outre que le ski, la randonnée pédestre et la natation ont également marqué des points auprès des plus jeunes.

Le jogging et le fitness figurent parmi les sports les plus prisés et ont aussi bénéficié d'un fort regain de popularité depuis 2008. Près d'un quart de la population suisse pratique le jogging ou la course à pied, une personne sur cinq déclarant faire du fitness ou une forme d'aérobic. La poursuite du boom du jogging est surtout imputable aux femmes, dont la proportion a grimpé de 5 points de pourcentage depuis 2008 et atteint désormais 50 %. L'âge moyen des personnes qui s'adonnent au jogging ou fréquentent un centre de fitness est d'environ 40 ans, contre 53 ans pour ceux qui font de la gymnastique. Cette dernière, en recul depuis six ans, reste néanmoins pratiquée par un dixième de la population. A ce titre, elle fait toujours partie des sports préférés des Suisses.

La fin du boom du walking

Le sport collectif le plus populaire est le football. Contrairement à d'autres sports de ballon comme le volleyball, le basketball ou le handball, le football affiche une légère progression depuis 2008. Les sports pouvant être pratiqués tout au long de la vie, mais aussi la danse, le yoga/tai chi/qi gong comptent parmi les activités qui enregistrent une augmentation de plus de deux points de pourcentage. En comparaison, le recul des autres sports est relativement faible. Outre la gymnastique, le nordic walking, le walking, l'inline-skating/le patinage sur roulettes et le volleyball/beachvolley sont ainsi en baisse de plus d'un demi-point de pourcentage. Le boom du nordic walking, révélé par l'enquête de 2008, appartient donc clairement au passé.

T 6.1: Pratique des différents sports en Suisse

	Activités citées (en % de la population)	Variation entre 2008 et 2014 (en points de pourcentage)	Fréquence de la pratique (nbre moyen de jours/an)	Age moyen (nbre d'années)	Part des femmes (in %)	Activité principale (en % de la population)
Randonnée pédestre, randonnée en montagne	44,3	+6,9	20	49	56	7,6
Cyclisme (hors VTT)	38,3	+2,7	45	44	53	7,0
Natation	35,8	+4,2	20	44	61	3,7
Ski alpin (hors randonnée à skis)	35,4	+8,8	10	42	49	3,0
Jogging, course à pied	23,3	+5,7	50	39	50	8,7
Fitness, aérobic	19,8	+7,2	90	41	58	9,6
Gymnastique	9,8	–2,1	45	53	73	4,1
Football	7,8	+0,4	45	30	11	3,2
Danse (y compris jazzdance)	7,8	+4,0	36	40	79	1,9
Walking, nordic walking	7,5	–1,3	45	52	84	2,9
Yoga, tai chi, qi gong	7,1	+3,2	45	46	88	3,0
VTT	6,3	+0,2	30	41	29	1,7
Tennis	5,2	+0,4	30	42	35	1,5
Snowboard (hors randonnée)	5,1	–0,1	10	27	44	0,3
Musculation, body-building	4,9	+1,5	90	40	43	1,3
Ski de fond	4,1	–0,4	10	50	54	0,2
Randonnée à skis/en snowboard, raquettes à neige	3,9	+1,2	10	48	56	0,2
Luge, bob	3,3	+3,1*	4	39	69	0,0
Badminton	3,2	+0,2	20	36	48	0,7
Inline-skating, patinage sur roulettes	3,0	–0,8	12	35	53	0,2
Volleyball, beach-volley	2,8	–0,6	40	32	57	1,1
Sports de combat, autodéfense	2,4	+0,5	52	32	31	1,3
Escalade, alpinisme	2,2	+0,5	15	34	42	0,5
Aqua-Fitness	1,9	–0,4	45	55	97	0,8
Golf	1,8	+0,6	40	51	43	0,8
Équitation, autres sports équestres	1,8	+0,1	90	34	85	1,1
Unihockey (y compris hockey sur gazon, rinkhockey)	1,7	–0,1	40	29	20	0,6
Basketball	1,6	–0,1	25	28	23	0,5
Squash	1,4	+0,1	15	36	23	0,3
Patinage	1,3	+0,5	5	40	72	0,0
Hockey sur glace	1,2	+0,2	25	33	2	0,4
Tir	1,1	0,0	40	46	13	0,3
Voile	1,1	+0,2	20	49	20	0,2
Tennis de table	0,9	+0,1	20	39	27	0,2
Plongée	0,9	+0,1	15	44	30	0,2
Aviron	0,6	+0,1	40	44	27	0,2
Planche à voile, surf, kitesurf	0,6	0,0	10	35	34	0,1
Athlétisme	0,6	–0,2	80	34	43	0,3
Handball	0,3	–0,4	70	22	24	0,2
Canoë, descentes d'eaux vives	0,2	0,0	10	42	67	0,0
Autres sports collectifs	0,9	–0,9	45	46	30	0,4
Autres sports d'aventure	1,2	+0,3	24	35	29	0,2
Autres sports d'endurance	0,6	–0,1	30	41	47	0,2
Autres sports	1,7	–0,3	30	46	29	0,4

Remarque: nombre de personnes interrogées: 10 652. Les valeurs indiquées tiennent compte des réponses fournies par les non-sportifs lorsque le sport en question a été cité parmi leurs activités physiques (cf. chapitre 9). La majorité des sports figurant dans le tableau regroupent des disciplines citées individuellement. Les catégories de sports ont été légèrement modifiées dans l'enquête Sport Suisse 2014. Aux fins de la comparaison avec les données de 2008, les chiffres ont été recalculés pour les deux enquêtes. Il faut préciser que les préférences des sportifs ne sont plus présentées séparément.
Les variations entre 2008 et 2014 qui n'ont pas d'importance statistique apparaissent en gris.
*La luge n'a pas été classée dans la même catégorie en 2014, ce qui explique qu'elle ait été plus fréquemment citée que lors de l'enquête 2008.

Tous les sports ne sont pas pratiqués avec la même assiduité. Alors que la luge et le patinage sont pratiqués quatre à cinq jours par an, les Suisses font du ski alpin, du snowboard et du ski de fond dix jours par an (valeurs moyennes). De nombreux sports sont pratiqués une fois par semaine en moyenne. Pour certains sports comme le fitness/l'aérobic, la musculation ou l'équitation, la fréquence est même de deux fois par semaine.

Il a été demandé aux personnes interrogées d'énumérer leurs activités sportives en commençant par la principale. La dernière colonne du tableau 6.1 révèle que le fitness/l'aérobic arrive devant le jogging, la randonnée pédestre, le cyclisme, la gymnastique, la natation et le football parmi les sports les plus fréquemment cités.

Les préférences varient selon le sexe et l'âge

Si les écarts entre les sexes se sont réduits depuis dix ans en termes d'ampleur de l'activité sportive (cf. chapitre 4), on relève encore des différences notables au niveau des sports pratiqués. Tandis que la proportion de femmes est de plus de 80 % dans les catégories aqua fitness, yoga/tai chi/qi gong, équitation et nordic walking, walking, elle est largement inférieure à 20 % pour le hockey sur glace, le football et le tir. De nombreux autres sports, tels que le ski alpin, le jogging ou le badminton, attirent autant de femmes que d'hommes.

Les sports de ballon comme le football, le basketball, le handball, mais aussi l'unihockey et le snowboard sont privilégiés essentiellement par les adolescents et les jeunes adultes. Dans ces catégories, l'âge moyen se situe entre 20 et 30 ans. Il convient de préciser que les activités sportives des enfants de moins de 15 ans ne sont pas prises en compte ici et feront l'objet d'un rapport séparé. A l'opposé, la moyenne d'âge des aficionados de ski de fond, de golf, de nordic walking, de walking, de gymnastique et d'aqua-fitness est de 50 ans.

G 6.1: Sports selon l'âge et le sexe (proportion de pratiquants en %)

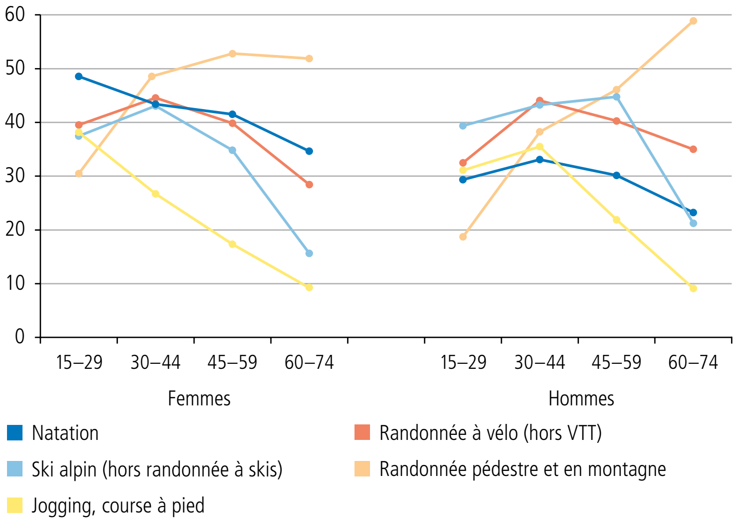

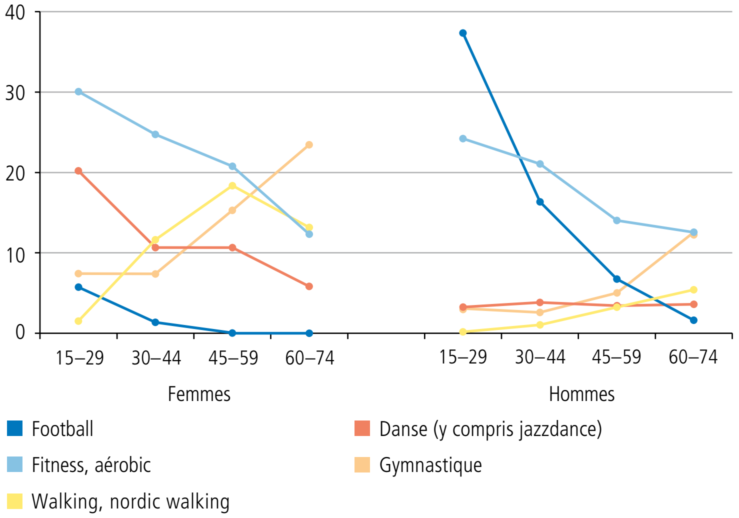

Remarque: nombre de personnes interrogées: 10 652

Le graphique 6.1 montre les dix sports de prédilection des Suisses en fonction de l'âge et du sexe. On relève des différences notables entre les sexes. La proportion de femmes qui pratiquent le jogging baisse continuellement avec l'âge. Chez les hommes en revanche, c'est dans la classe des 30-44 ans qu'on trouve le plus de joggers. La randonnée pédestre est particulièrement appréciée des femmes, y compris par les plus jeunes d'entre elles. Chez les hommes, l'intérêt pour ce sport augmente constamment avec l'âge pour atteindre son point culminant entre 60 et 74 ans. La tendance est radicalement inverse pour le fitness/l'aérobic, de moins en moins prisé avec l'âge, aussi bien par les femmes que par les hommes. Les différences liées à l'âge et au sexe sont particulièrement marquées dans le football, qui fait partie des sports préférés des hommes jeunes avec le ski alpin.

Les sports pouvant être pratiqués tout au long de la vie suscitent un véritable engouement parmi les sportifs

A la question de savoir s'il existe des sports qu'ils souhaiteraient commencer ou pratiquer davantage, un tiers des sportifs répondent «oui». Les femmes expriment ce désir de façon un peu plus marquée que les hommes (37 % contre 32 %). Au total, plus de 180 sports ont ainsi été cités. En tête de liste, la natation, le cyclisme, le fitness/l'aérobic, la danse et la randonnée pédestre côtoient de nombreux autres sports parmi les plus pratiqués. A noter que le nordic walking et le walking sont beaucoup moins souvent cités que lors de la précédente enquête. Arrivé en quatrième position en 2008, il se classe en 18e position seulement en 2014. Inversement, la natation, la randonnée pédestre et le cyclisme sont plus populaires qu'il y a six ans (cf. tableau 6.2).

La comparaison du nombre de personnes qui souhaitent pratiquer (ou pratiquer davantage) un sport avec le nombre de pratiquants actuels permet d'établir que c'est la catégorie planche à voile/surf/kitesurf qui revêt le potentiel de croissance le plus élevé. Cela s'explique non seulement par le succès récent du kitesurf, actuellement très tendance, mais aussi par les valeurs sûres que sont la planche à voile et le surf, qui fascinent tout autant les foules. L'aviron, les sports de combat, la voile, la plongée et le golf présentent eux aussi un potentiel de croissance considérable. Cela étant dit, compte tenu des paramètres financiers et du temps requis par ces activités, un certain nombre de ces souhaits devraient ne pas se concrétiser.

T 6.2: Activités que les sportifs souhaiteraient pratiquer (ou pratiquer davantage)

	Activités citées (en % de tous les sportifs)	Variation entre 2008 et 2014 (en points de pourcentage)	Age moyen (nbre d'années)	Part des femmes (en %)
Natation	4,4	+1,8	43	61
Cyclisme (hors VTT)	4,1	+1,2	48	53
Fitness, aérobic	3,7	+0,9	39	65
Danse (y compris jazzdance)	2,9	+1,0	37	88
Randonnée pédestre, randonnée en montage	2,8	+1,4	50	62
Yoga, tai chi, qi gong	2,5	+1,1	44	90
Tennis	2,4	+0,7	37	53
Ski alpin (hors randonnée à skis)	2,3	+0,8	41	54
Jogging, course à pied	2,1	+0,6	40	69
Sports de combat, autodéfense	2,1	+0,5	29	46
Ski de fond	1,6	+0,8	49	60
Escalade, alpinisme	1,6	+0,5	32	48
Gymnastique	1,2	+0,2	48	85
Golf	1,1	–0,1	48	27
Randonnée à skis/en snowboard, raquettes à neige	1,1	+0,3	45	61
Planche à voile, surf, kitesurf	1,1	+0,4	37	40
Équitation, autres sports équestres	1,0	+0,3	39	83
Walking, nordic walking	0,9	–1,6	53	89
Volleyball, beach-volley	0,8	+0,1	31	75
VTT	0,7	+0,1	39	61
Badminton	0,7	+0,1	37	74
Voile	0,7	+0,1	43	32
Football	0,6	+0,1	31	17
Inline-skating, patinage sur roulettes	0,6	–0,3	39	44
Musculation, body-building	0,6	+0,1	37	42
Aqua-fitness	0,6	–0,2	51	93
Aviron	0,5	+0,1	41	51
Plongée	0,5	+0,1	39	36
Snowboard (hors randonnée)	0,3	0,0	29	48
Basketball	0,3	+0,2	26	35
Squash	0,3	+0,1	32	59
Unihockey (y compris hockey sur gazon, rinkhockey)	0,3	0,0	26	14

Remarque: nombre de personnes interrogées: 7964 (sportifs uniquement). Les variations entre 2008 et 2014 qui n'ont pas d'importance statistique apparaissent en gris.

7. Différences régionales

L'activité sportive ne varie pas selon que les pratiquants habitent en ville ou à la campagne

A l'instar de l'âge et du sexe, la différence entre ville et campagne n'est plus un facteur déterminant de la diversité de l'activité sportive. Dans les villes, on fait aujourd'hui quasiment autant de sport que dans les communes d'agglomération et les communes rurales (cf. graphique 7.1). On relève certaines disparités en fonction de la taille des communes sans toutefois qu'un schéma clair puisse en être déduit. Les facteurs structurels déterminent davantage les différences constatées que le nombre d'habitants. Ces facteurs sont décrits plus en détail au chapitre suivant et sont partiellement en relation avec la taille de la commune. Les communes à revenu élevé comptent ainsi un nombre particulièrement élevé de sportifs très actifs, tandis que la proportion des non-sportifs est très importante dans les petites communes agricoles. Le premier constat s'explique par une proportion élevée de personnes aux revenus confortables, le second par la présence d'un grand nombre de travailleurs agricoles (cf. graphique 8.1).

Des sports de prédilection différents

Si l'activité sportive de la population ne dépend ni de l'environnement (ville ou campagne) ni de la taille de la commune, on observe néanmoins des différences intéressantes au niveau des préférences sportives. Autrement dit, si l'ampleur de l'activité sportive est plutôt homogène, on relève des spécificités au niveau des sports privilégiés. Le combiné helvétique est partout en tête de liste, avec une préférence plus marquée pour la randonnée pédestre et le ski alpin à la campagne. La ville se distingue quant à elle par un plus grand nombre de joggers. Le cyclisme est populaire en ville comme à la campagne, même si le VTT, le walking et l'équitation sont moins prisés dans les villes, où le football et la danse sont en revanche plus souvent pratiqués. Le tennis, le volleyball et le golf sont des sports très répandus dans les communes d'agglomération.

G 7.1: Activité sportive en fonction de l'environnement (ville-campagne), de la taille de la commune et de la région (en % de la population résidante suisse âgée de 15 à 74 ans)

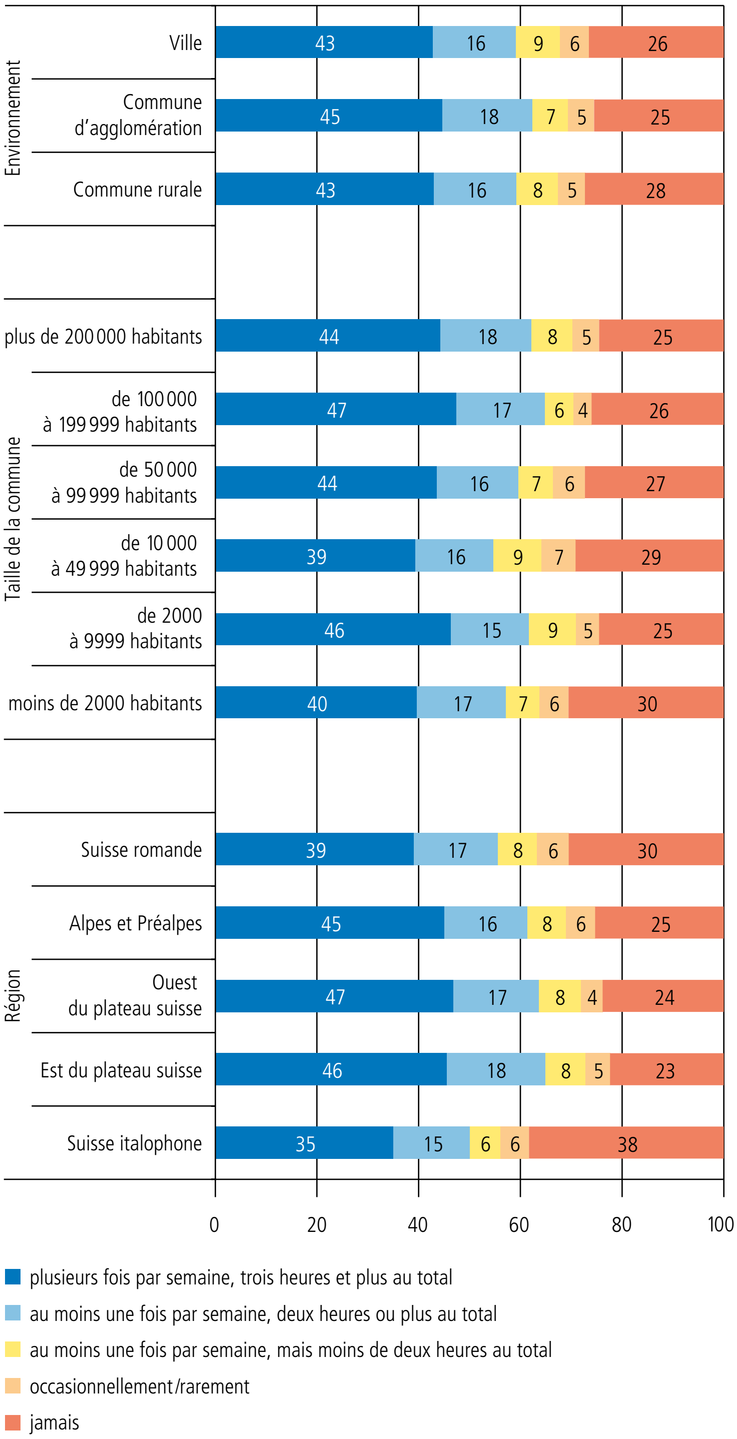

Remarque: nombre de personnes interrogées: 10 622. Taille de la commune et environnement (ville-campagne) selon la codification de l'Office fédéral de la statistique. Subdivision par régions REMP.

La Suisse alémanique est la plus sportive, talonnée par la Suisse romande

Le constat selon lequel il n'y a guère de disparités régionales en termes d'activité sportive concerne la distinction entre ville et campagne et la taille des communes, non les régions linguistiques. Le graphique 7.1 met en évidence que la population de Suisse romande et de Suisse italophone est moins sportive que celle de la Suisse alémanique, exceptionnellement homogène en la matière.

Les disparités entre régions linguistiques ne sont pas un fait nouveau. Elles ont déjà été analysées de façon approfondie lors des deux précédentes éditions de l'enquête Sport Suisse, en 2000 et en 2008. Par conséquent, ce n'est pas l'ampleur des écarts constatés qui est surprenante, mais plutôt le fait que les différences se sont considérablement amenuisées en six ans. Le graphique 7.2 montre que l'activité sportive a sensiblement augmenté en Suisse romande notamment, où la proportion de sportifs très actifs est désormais équivalente à la moyenne enregistrée pour la Suisse alémanique en 2000. La Suisse italophone affiche même une plus forte progression de l'activité sportive que la Suisse alémanique en 2000. Les chiffres ont donc tendance à converger.

Fait particulièrement réjouissant, le boom observé en Suisse romande concerne essentiellement les femmes et les seniors de sexe masculin, jusqu'ici peu actifs dans cette région (cf. graphiques 7.3 et 7.4). En Suisse romande, les femmes font beaucoup plus de sport qu'en 2008, toutes classes d'âge confondues. Chez les hommes, on observe pour la première fois la reprise typique de l'activité sportive à l'âge de la retraite.

G 7.2: Activité sportive dans les trois régions linguistiques de 2000 à 2014 (en %)

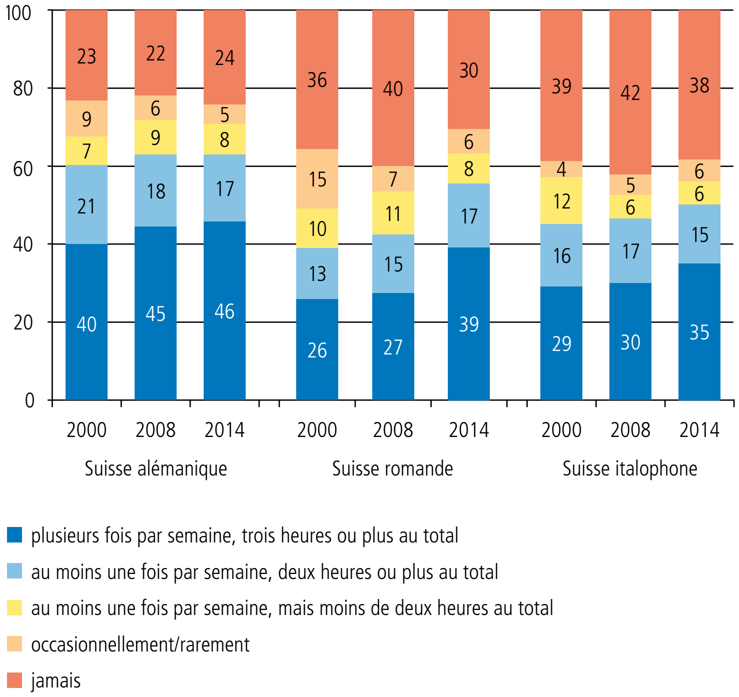

Remarque: nombre de personnes interrogées: 2000: 2058; 2008: 10 242; 2014: 10 622.

Le cyclisme particulièrement apprécié en Suisse alémanique

Le tableau 7.1 permet d'identifier les sports qui ont le vent en poupe en Suisse romande. La randonnée pédestre, le ski alpin, le fitness, le jogging et la natation ont progressé de plus de 5 points de pourcentage. Le jogging a également bénéficié d'un regain de popularité au Tessin, avec le ski alpin, la randonnée et le fitness. En dépit de cette évolution, les sports pouvant être pratiqués tout au long de la vie (cyclisme, randonnée pédestre, natation, mais aussi jogging, walking, gymnastique, musculation et snowboard) restent nettement plus répandus en Suisse alémanique qu'en Suisse romande et italophone. On relève ainsi des écarts particulièrement flagrants au niveau du cyclisme. Pratiqué par 43 % de la population en Suisse alémanique (soit presque autant que la randonnée), ce sport ne représente que 25 % de l'activité sportive en Suisse romande et italophone. Contrairement au snowboard, le ski alpin jouit en revanche d'une popularité équivalente en Suisse romande et en Suisse alémanique. Les sports plus pratiqués en Suisse romande qu'en Suisse alémanique comptent, entre autres, la randonnée à skis et en snowboard, le VTT et le tennis. Ce dernier attire également légèrement plus de sportifs en Suisse italophone qu'en Suisse alémanique.

T 7.1: Sports pratiqués dans les différentes régions linguistiques

	Suisse alémanique		Suisse romande		Suisse italophone	
	Activités citées (en % de la population)	Variation entre 2008 et 2014 (en points de pourcentage)	Activités citées (en % de la population)	Variation entre 2008 et 2014 (en points de pourcentage)	Activités citées (en % de la population)	Variation entre 2008 et 2014 (en points de pourcentage)
Randonnée pédestre, randonnée en montagne	45,7	+5,4	40,6	+11,5	41,0	+5,1
Cyclisme (hors VTT)	43,3	+3,1	25,7	+2,5	26,6	–1,3
Natation	38,3	+3,5	29,7	+6,6	29,7	+1,5
Ski alpin (hors randonnée à skis)	36,5	+8,5	34,4	+9,9	22,6	+5,8
Jogging, course à pied	26,0	+5,2	16,4	+7,1	18,0	+7,9
Fitness, aérobic	20,6	+7,3	18,5	+7,2	13,1	+5,1
Gymnastique	10,8	–2,7	7,3	–0,2	7,3	–3,9
Football	8,0	–0,2	7,3	+1,6	6,9	+2,6
Danse (y compris jazzdance)	8,7	+4,3	6,0	+3,4	2,7	+0,3
Walking, nordic walking	8,9	–1,5	4,3	–1,2	2,0	–0,4
Yoga, tai chi, qi gong	7,7	+4,0	5,3	+0,9	6,4	+2,1
VTT	5,8	-1,0	8,2	+3,1	5,1	+3,6
Tennis	4,7	+0,1	6,6	+1,1	6,0	+1,5
Snowboard (hors randonnée)	6,1	–0,4	2,3	+0,4	3,1	+1,6
Musculation, body-building	6,2	+1,4	1,4	+1,3	2,7	+2,0
Ski de fond	4,0	–1,0	4,7	+1,6	2,0	–1,0
Randonnée à skis/en snowboard, raquettes à neige	3,2	+0,7	6,6	+2,8	1,8	+0,7
Luge, bob	4,1	+3,8*	1,5	+1,5*	0,2	0,0
Badminton	3,1	+0,4	3,9	–0,7	0,4	+0,4
Inline-skating, patinage sur roulettes	3,6	–1,1	1,7	+0,3	0,2	–0,9
Volleyball, beach-volley	3,4	–0,4	1,6	–1,1	0,2	–1,1

	Suisse alémanique		Suisse romande		Suisse italophone	
	Activités citées (en % de la population)	Variation entre 2008 et 2014 (en points de pourcentage)	Activités citées (en % de la population)	Variation entre 2008 et 2014 (en points de pourcentage)	Activités citées (en % de la population)	Variation entre 2008 et 2014 (en points de pourcentage)
Sports de combat, autodéfense	2,4	+0,3	2,6	+1,2	1,8	+0,7
Escalade, alpinisme	2,3	+0,7	2,2	+0,3	0,9	−0,6
Aqua-Fitness	1,7	−0,5	2,5	0,0	2,0	+0,1
Golf	1,8	+0,3	2,1	+1,4	1,3	+0,9
Équitation, autres sports équestres	1,9	0,0	1,9	+0,2	1,3	+0,3
Unihockey (y compris hockey sur gazon, rinkhockey)	2,0	−0,1	0,7	0,0	0,9	0,0
Basketball	1,6	−0,2	1,8	0,0	1,3	+0,9
Squash	1,5	+0,2	1,4	−0,1	0,7	0,0
Patinage	1,3	+0,6	1,1	+0,2	1,8	+0,5
Hockey sur glace	1,4	+0,3	0,8	0,0	0,7	−0,4
Tir	1,3	0,0	0,8	+0,1	0,7	−0,4
Voile	1,1	+0,2	1,0	+0,1	1,1	+0,5
Tennis de table	1,0	+0,2	0,8	−0,3	0,9	+0,2
Plongée	0,9	0,0	0,9	+0,5	0,2	−0,6
Aviron	0,7	+0,1	0,2	−0,1	0,2	0,0
Planche à voile, surf, kitesurf	0,6	+0,1	0,4	+0,1	0,2	0,0
Athlétisme	0,6	−0,2	0,4	0,0	0,0	−0,9
Handball	0,4	−0,5	0,2	0,0	0,2	0,0
Canoë, descentes d'eaux vives	0,2	0,0	0,1	+0,1	0,0	−0,2
Autres sports collectifs	0,9	−1,0	0,9	−0,4	1,3	0,0
Autres sports d'aventure	1,2	+0,1	1,4	+0,8	0,7	+0,2
Autres sports d'endurance	0,7	+0,1	0,5	−0,6	0,4	−0,4
Autres sports	1,9	−0,1	1,3	−0,5	0,7	−1,9

Remarque: nombre de personnes interrogées: Suisse alémanique: 7995; Suisse romande: 2065; Suisse italophone: 592. Outre les activités citées par les sportifs, les valeurs indiquées tiennent compte également des réponses fournies par les non-sportifs lorsque le sport en question figure parmi leurs activités physiques (cf. chapitre 9). La majorité des sports figurant dans le tableau regroupent des disciplines citées individuellement. Les catégories de sports ont été légèrement modifiées dans l'enquête Sport Suisse 2014. Aux fins de la comparaison avec les données de 2008, les chiffres ont été recalculés pour les deux enquêtes. Pour l'essentiel, l'évolution observée en Suisse italophone entre 2008 et 2014 n'est pas pertinente d'un point de vue statistique en raison du faible nombre de cas. Cette évolution doit donc être interprétée avec circonspection (cf. chapitre 18). Les variations entre 2008 et 2014 qui n'ont pas d'importance statistique apparaissent en gris.
*La luge n'a pas été classée dans la même catégorie en 2014, ce qui explique qu'elle ait été plus fréquemment citée que lors de l'enquête 2008.

8. Différences sociales

L'activité sportive dépend du statut social

Malgré l'essor du sport et son ouverture à un plus vaste public depuis quelques décennies, toutes les catégories de la population ne sont pas égales en la matière, que ce soit en termes d'assiduité ou de type d'activité. Si les différences sociodémographiques se sont sensiblement atténuées entre les sexes, les différentes classes d'âge ou les régions, les disparités socioéconomiques persistent.

Le graphique 8.1 montre l'influence sur l'activité sportive du niveau de formation, de la situation au regard de l'emploi, de la profession, du secteur d'activité, du revenu du ménage et de la situation personnelle. En matière de formation, le constat le plus flagrant est la faible activité sportive des personnes qui ont suivi uniquement la scolarité obligatoire. Près de la moitié d'entre elles ne pratique aucun sport. Plus le niveau de formation est élevé, plus la personne est active. A cet égard, il n'y a guère de différence entre les diplômés des hautes écoles et les universitaires.

Influence de la situation professionnelle et personnelle sur le comportement sportif

Les personnes qui sont encore en formation se distinguent, notamment en raison de leur jeune âge, par une activité sportive importante. A contrario, les retraités sont soit des sportifs très actifs, soit inactifs. L'activité sportive des ménagères est inférieure à la moyenne (s'agissant de femmes dans 98 % des cas, il est possible de faire l'économie de la forme masculine). Elle est liée à la présence d'enfants de moins de six ans dans le ménage. Dans ce cas, la proportion d'inactifs est en effet supérieure à un tiers (35 %). Lorsque les enfants sont plus grands ou si le ménage ne compte pas d'enfants, elle tombe à un quart (cf. chapitre 4). Le chômage concerne trois pourcent des personnes interrogées. Les personnes sans emploi se distinguent par une faible activité sportive (54 % d'inactifs), notamment lorsque le chômage est lié à des raisons de santé. L'activité sportive des autres chômeurs est comparable à celle des retraités.

G 8.1: Activité sportive selon le niveau de formation, la situation au regard de l'emploi, la catégorie socioprofessionnelle, le revenu et la composition du ménage (en %)

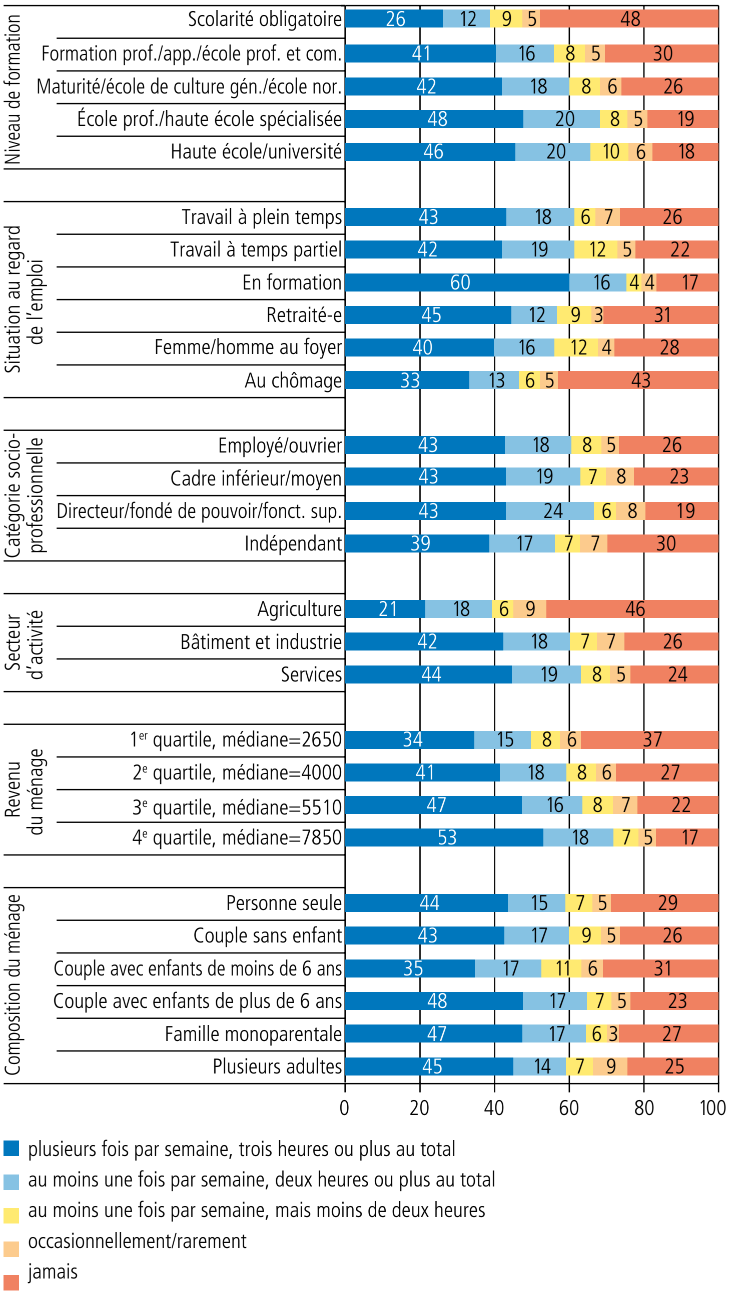

Remarque: niveau de formation: correspond au niveau de formation achevée le plus élevé ou à la formation en cours; concerne uniquement les plus de 24 ans, n=7982; catégorie socioprofessionnelle et secteur d'activité: concerne uniquement les personnes qui ont une activité rémunérée, n=5709/6352; revenu (mensuel net en CHF): le revenu par équivalent adulte indique le budget disponible pour chaque membre du ménage. Les quartiles englobent quatre catégories de revenu d'égale importance: le 1er quartile représente le quart des personnes au revenu le plus faible, le 2e quartile le quart suivant, etc. n=7949; composition du foyer: n=10 597.

La catégorie socioprofessionnelle a surtout une influence sur la proportion d'inactifs: plus on grimpe dans la hiérarchie, plus le nombre d'inactifs régresse. Ce sont les travailleurs indépendants qui affichent la plus faible activité sportive. A noter que ceux qui n'ont pas d'employés et les conjoints collaborateurs comptent souvent parmi les non-sportifs (respectivement 33 % et 47 % d'inactifs). Le secteur d'activité est plus déterminant que la catégorie socioprofessionnelle. Les travailleurs du secteur primaire (agriculture) sont moins sportifs que ceux des secteurs secondaire et, surtout, tertiaire. On observe une relation étroite et linéaire avec le revenu: plus ce dernier est élevé, plus les membres du ménage font de sport.

Le revenu constitue le facteur d'influence prédominant

Les effets du niveau de formation, du statut professionnel, du revenu ou de la situation personnelle, mais aussi de l'âge et du sexe s'additionnent et se renforcent ou, par contre, se neutralisent. Partant de là, lorsqu'on tente d'identifier les facteurs d'influence déterminants à l'aide d'une méthode statistique complexe (analyse de variance multivariée), il apparaît que c'est le revenu qui prédomine, suivi du niveau de formation (cf. graphiques 8.2 et 8.3). Indépendamment de l'âge et du sexe, il ressort que les personnes qui ont un revenu très confortable sont plus sportives que celles dont le revenu est inférieur à la moyenne. Parmi ces dernières, la proportion d'inactifs est beaucoup plus élevée, quel que soit leur sexe et toutes classes d'âge confondues. Leur part est presque deux fois plus élevée chez les hommes entre 30 et 59 ans. Les effets sont comparables du côté des sportifs très actifs, avec toutefois certaines différences notables: chez les femmes, c'est sur les plus jeunes et les seniors que les écarts de revenu ont la plus grande d'influence; chez les hommes, elle est plus marquée sur les classes d'âge moyennes. Ainsi, la tendance à la baisse d'activité liée aux aléas de la vie et la reprise ultérieure d'une pratique sportive sont particulièrement nettes parmi les femmes les mieux loties. A contrario, Le modèle masculin type, caractérisé par une baisse importante de l'activité jusqu'à 60 ans suivie d'une reprise à la retraite, concerne plus particulièrement les hommes à faible revenu.

G 8.2: Proportion d'inactifs parmi les catégories de revenu supérieure et inférieure en fonction de l'âge et du sexe (en %)

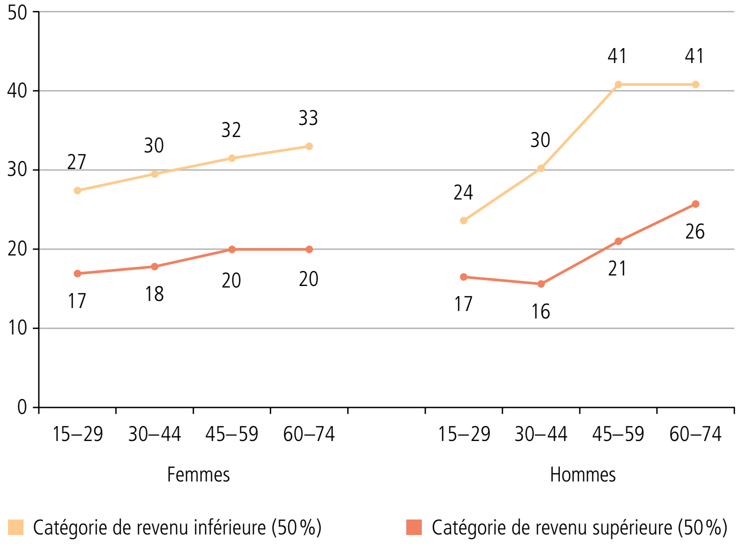

Remarque: revenu par équivalent adulte, nombre de personnes interrogées: 7949

G 8.3: Proportion de sportifs très actifs parmi les catégories de revenu supérieure et inférieure en fonction de l'âge et du sexe (en %)

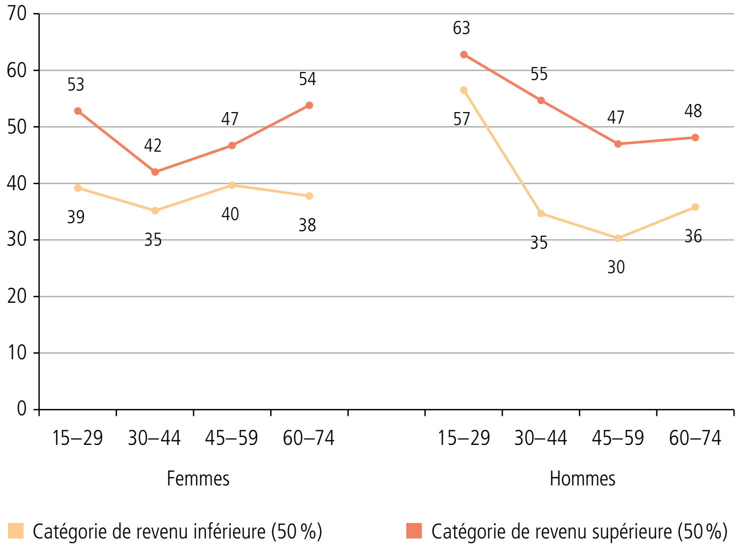

Remarque: revenu par équivalent adulte, nombre de personnes interrogées: 7949

Le revenu joue également un rôle majeur dans le choix du sport. Les sports suivants sont pratiqués deux fois plus souvent par les personnes avec un revenu supérieur à la moyenne que par les catégories les moins aisées: golf (facteur de 2,8), voile (2,6), plongée sous-marine (2,6), tennis (2,2), squash (2,2) et aviron (2,2). Parmi les sports populaires qui peuvent être pratiqués tout au long de la vie, le ski alpin et le jogging sont ceux dont le choix est le plus influencé par le niveau de revenu. Inversement, les activités comme la gymnastique, la luge et le tir sont davantage prisées par les personnes avec un revenu inférieur à la moyenne.

Influence de la nationalité sur l'activité sportive

La nationalité est un facteur d'influence important de l'activité sportive. Les Suisses font nettement plus de sport que la population résidante immigrée. Tandis qu'un quart des Suisses de souche ne pratique aucun sport, les deux cinquièmes de la population étrangère sont inactifs (cf. graphique 8.4). Une analyse détaillée par pays d'origine révèle des disparités importantes entre les différentes populations immigrées. Les étrangers venant d'Europe de l'Ouest ou du Nord sont plus sportifs que les Suisses, alors que ceux en provenance des Balkans, de Turquie ainsi que d'Europe de l'Est et du Sud sont nettement moins actifs.

Les disparités observées au niveau des différentes nationalités sont aussi fort probablement imputables aux facteurs d'influence présentés dans le graphique 8.1 (niveau de formation, catégorie socioprofessionnelle et revenu). Autrement dit, la faible activité sportive de la population immigrée en provenance d'Europe du Sud s'expliquerait par des facteurs liés, non pas à l'origine, mais à des niveaux de formation et de revenu moindres ainsi qu'au statut professionnel. En appliquant une méthode statistique complexe (analyse de variance multivariée) tenant compte de la situation sociale disparate des diverses nationalités, les différences entre les Suisses et la population immigrée d'Europe de l'Ouest et du Nord s'effacent, mais elle demeurent avec les étrangers en provenance des Balkans, de Turquie et d'Europe du Sud et de l'Est, même si elles sont moins importantes.

G 8.4: Activité sportive selon la nationalité et la région d'origine (en %)

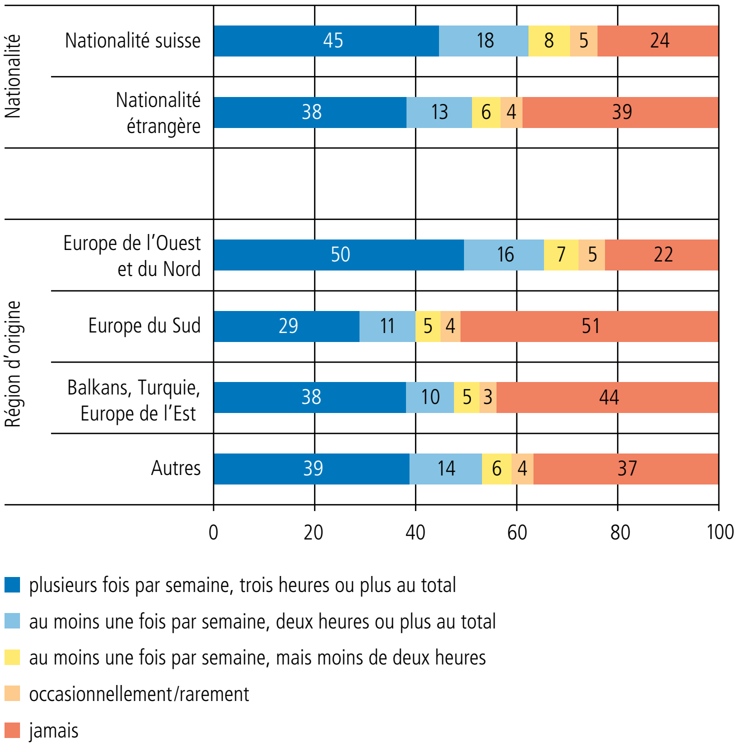

Remarque: nationalité: n=10614; région d'origine: uniquement personnes de nationalité étrangère n=2965.

Plus la durée de séjour augmente, plus les comportements sportifs se nivellent

Les disparités constatées reflètent donc des différences d'ordre socioéconomique, mais également culturel entre les régions d'origine. Comme le montre le graphique 2.3, qui permet de comparer les données au niveau européen, les pays situés au sud et à l'est de la Suisse se caractérisent par une activité sportive moindre. Il est intéressant de constater que ces différences en termes de «culture du sport» s'atténuent à mesure que la durée de séjour en Suisse augmente. Le comportement sportif de la population immigrée naturalisée qui réside en Suisse depuis de nombreuses années se rapproche progressivement de celui des Suisses de souche. De même, l'activité sportive des citoyens au bénéfice d'une double nationalité est très proche de celui des Suisses de souche. On relève toutefois un nombre d'inactifs légèrement supérieur parmi les personnes naturalisées en provenance des Balkans, de Turquie et d'Europe de l'Est ainsi que, dans une moindre mesure, d'Europe du Sud.

Des différences importantes entre les sexes au sein de la population immigrée

Les écarts entre les sexes méritent une attention particulière dans le cadre d'une analyse différenciée de l'activité sportive de la population résidante d'origine étrangère. Si les courbes d'activité sportive des Suissesses et des Suisses se sont considérablement rapprochées depuis 20 ans (cf. chapitre 4), les différences entre les sexes restent marquées au sein de la population résidante d'origine étrangère (cf. graphiques 8.5 et 8.6). Les femmes d'origine étrangère font moins de sport que leurs homologues masculins, toutes classes d'âge confondues. L'activité sportive des hommes d'origine étrangère est quant à elle équivalente à celle des Suisses de la gent masculine, notamment chez les plus jeunes et parmi les sportifs très actifs. On compte deux fois plus d'inactives parmi les femmes d'origine étrangère que parmi les Suissesses, toutes classes d'âge confondues. Il faut souligner le faible pourcentage de sportives très actives parmi les jeunes étrangères. Les écarts seraient plus marqués encore si l'analyse ne tenait compte que des étrangères en provenance des Balkans, de Turquie et d'Europe de l'Est et du Sud. Parmi celles-ci, la part de sportives très actives n'est que de 22 % chez les plus jeunes, pour une proportion d'inactives de 55 %. Leurs pendants masculins affichent quant à eux une activité sportive comparable à celle des Suisses.

La nationalité a, au même titre que le revenu, une incidence sur le choix du sport pratiqué. Le tir, le hockey sur glace et l'unihockey ainsi que, dans une moindre mesure, le volleyball, le walking, l'aqua-fitness, la gymnastique, le ski alpin, la randonnée à skis et le ski de fond attirent particulièrement peu les étrangers. A l'inverse, on trouve un nombre très important de personnes de nationalité étrangère dans le football et, dans une moindre mesure, dans les sports de combat et le basketball.

G 8.5: Proportion d'inactifs selon la nationalité, l'âge et le sexe (en %)

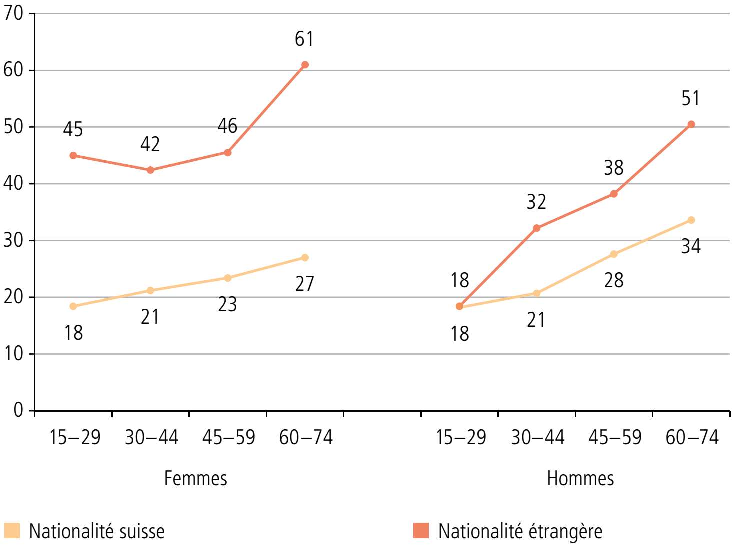

Remarque: nombre de personnes interrogées: 10 614

G 8.6: Proportion de sportifs très actifs selon la nationalité, l'âge et le sexe (en %)

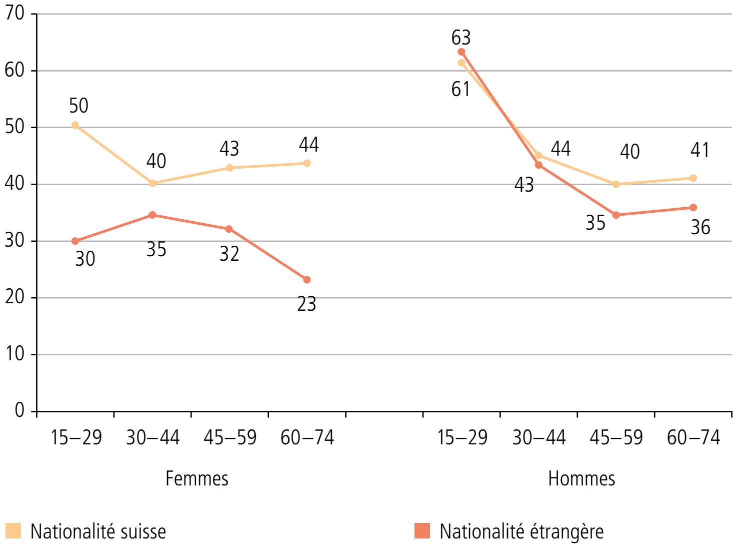

Remarque: nombre de personnes interrogées: 10 614

9. Les non-sportifs

L'inactivité est favorisée par des facteurs sociaux

Un bon quart de la population suisse peut être classé parmi les non-sportifs. Il s'agit des personnes qui déclarent ne faire aucun sport (cf. chapitre 2). La proportion de non-sportifs est pour ainsi dire équivalente chez les femmes et les hommes. Parmi les plus jeunes, on compte un peu plus de femmes inactives. En revanche, à partir de 50 ans, la proportion d'hommes est plus élevée (cf. graphique 4.3). La part des non-sportifs augmente légèrement avec l'âge. Pour autant, l'inactivité et l'âge ne sont pas directement liés. Les facteurs sociaux tels que le niveau de formation, le revenu ou la nationalité ont une plus grande incidence sur le comportement sportif que l'âge (cf. chapitre 8). En principe, les personnes qui n'ont pas fait beaucoup d'études, dont le revenu est inférieur à la moyenne ou qui sont de nationalité étrangère font plutôt partie de la catégorie des non-sportifs. Il faut toutefois se garder de faire des généralisations hâtives: la majorité des non-sportifs sont suisses et appartiennent à la classe moyenne, qui est très large.

Une perception positive du sport

Ni la proportion de non-sportifs, ni leur profil social n'ont beaucoup évolué en 15 ans. De là à en déduire qu'environ un quart de la population ne s'intéresse pas au sport et qu'il s'agit d'un fait immuable, il n'y a qu'un pas... qu'il vaut mieux ne pas franchir. Un examen approfondi des expériences passées, des motivations et des désirs des non-sportifs révèle que ces derniers ne sont pas définitivement fermés à toute activité sportive et n'émettent aucune réserve de principe sur le sport.

En définitive, l'idée que les non-sportifs se font du sport n'est pas si différente de la perception qu'en ont les sportifs (cf. chapitre 5). Par rapport à ces derniers, les non-sportifs associent un peu moins le sport à la proximité de la nature, à la détente, au plaisir, à la connaissance de son corps, à la santé et à la liberté, mais un peu plus au business, aux blessures et au dopage. La santé, l'exercice physique et le plaisir n'en sont pas moins le trio de tête dans le classement des termes les plus fréquemment cités (cf. graphique 9.1). Si les aspects négatifs comme la contrainte, la violence, la tricherie ou la corruption reviennent un peu plus souvent, ils restent rarement évoqués.

Quelque trois quarts des non-sportifs (71 %) ont fait du sport par le passé en dehors du cadre scolaire. Une très grande majorité d'entre eux (92 %) a pratiqué cette activité régulièrement, soit au moins une fois par semaine. Les deux cinquièmes de la catégorie des non-sportifs ont été membres d'un club sportif par le passé, dont la moitié de plusieurs clubs. Ces chiffres sont confirmés par les réponses fournies à la question relative à l'ancien parcours de sportif des sondés. Un large quart de non-sportifs déclare n'avoir jamais fait (beaucoup) de sport. Environ la moitié d'entre eux évoque un véritable passé de sportif. Enfin, près d'un quart des inactifs déclare avoir fait du sport sporadiquement à différentes phases de la vie.

Les expériences qu'ils ont faites dans ce cadre étaient majoritairement positives. Seuls 5 % des non-sportifs pointent une expérience négative du sport et 11 % ont des sentiments mitigés sur leurs activités sportives passées et en gardent de bons comme de mauvais souvenirs. Les 84 % restants évoquent une expérience positive.

G 9.1: Termes associés au sport par les non-sportifs

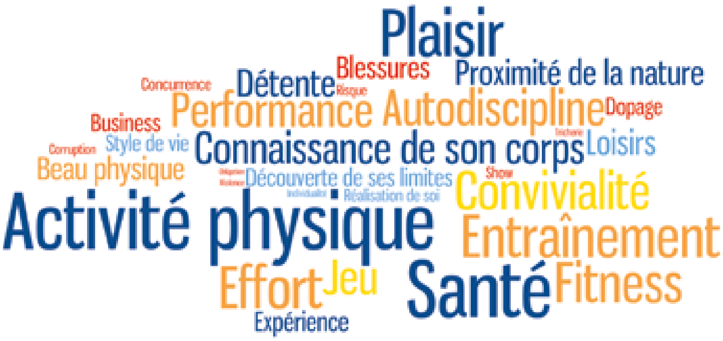

Remarque: nombre de personnes interrogées: 1809 (non-sportifs, enquête en ligne). Nuage de mots-clés représentés en fonction de la fréquence de sélection des termes par les personnes interrogées. Les couleurs renvoient à une analyse factorielle des combinaisons de termes fréquentes.

Les bons souvenirs sont surtout associés au sport en club (52 % des inactifs rapportant des expériences positives du sport), à une pratique «libre» (27 %) et au sport scolaire (25 %). Ce dernier est aussi le plus fréquemment cité en lien avec les mauvais souvenirs (57 % des inactifs relatant des expériences négatives du sport), également associés par certains au sport associatif (18 %). En revanche, la pratique libre, la famille, les événements sportifs, le sport scolaire facultatif, les centres de fitness ou autres prestataires privés ne sont que rarement à l'origine d'expériences négatives. Plus de la moitié (55 %) des non-sportifs qui ont fait du sport exclusivement dans un cadre scolaire en ont plutôt un bon souvenir. Quelque 17 % des personnes qui n'ont connu que le sport scolaire en ont une expérience mitigée à négative, tandis que 11 % déclarent ne pas avoir bénéficié de cours de sport. Il s'agit généralement de femmes d'âge mûr ayant interrompu leur scolarité très tôt et issues de l'immigration.

Les expériences négatives associées au sport scolaire, mais aussi au sport pratiqué en club et dans d'autres cadres, sont liées essentiellement à un «mauvais» professeur ou entraîneur. Les blessures, une pression excessive et les mésententes avec des coéquipiers figurent parmi les autres sources d'insatisfaction citées. Certains pointent également l'absence de plaisir lors de la pratique et de l'entraînement ou estiment qu'ils n'étaient pas à la hauteur des exigences.

L'inactivité n'est pas un style de vie

Les non-sportifs ne sont pas fermés à toute forme de sport, comme le prouve l'analyse des motifs invoqués par les personnes interrogées pour justifier leur inactivité (cf. tableau 9.1). C'est avant tout par manque de temps («surcharge de travail», «horaires de travail étendus ou défavorables» ou «Sport au détriment de la vie de famille») qu'elles renoncent au sport. Un autre groupe d'individus, beaucoup plus réduit, pointe l'absence de plaisir associée à la pratique d'un sport, lui préfère d'autres centres d'intérêt, ne se sent pas fait pour le sport ou justifie son inactivité par un trop grand âge. Les raisons de santé figurent également en bonne place dans la liste ou encore les blessures. Enfin, certains déclarent faire suffisamment d'exercice sans pratiquer un sport et s'adonner à d'autres activités physiques. A noter que l'offre est très peu mise en cause. Les motifs tels que l'absence d'une offre adaptée, les problèmes d'accessibilité ou l'incompatibilité des horaires d'ouverture sont rarement évoqués.

Il n'est donc pas surprenant que les non-sportifs n'émettent aucune réserve de principe sur le sport. Si l'on se réfère aux toutes dernières recommandations en matière d'activité physique, il ressort nettement que 66 % des non-sportifs font de l'exercice au quotidien sur leur lieu de travail et satisfont aux préconisations minimales en la matière (cf. graphique 3.4). Comme le met en évidence le tableau 9.2, certaines de ces activités peuvent être assimilées à un «sport». Un tiers des non-sportifs fait ponctuellement de la randonnée, un quart fréquente occasionnellement les piscines ou fait du vélo de temps en temps. Enfin, un cinquième des inactifs pratique le ski alpin à l'occasion. La promenade du chien, la danse, la marche et la luge figurent parmi les autres activités physiques pratiquées par les non-sportifs à titre ponctuel. L'intensité ou l'ampleur de ces activités ne permet pas à ceux qui les pratiquent de les déclarer comme un sport. Au total, trois quarts des non-sportifs disent pratiquer ce type d'activité.

T 9.1: Raisons avancées par les non-sportifs pour expliquer leur inactivité

	en % des non-sportifs
Manque de temps	40,3
Manque d'envie et de plaisir	18,2
Raisons de santé	18,2
Activité physique suffisante, se sent en bonne forme	9,4
Surcharge de travail, excès de fatigue	7,7
A d'autres centres d'intérêt	4,9
Horaires de travail étendus ou défavorables	8,9
A d'autres activités physiques	3,8
Sport au détriment de la vie de famille	3,4
Ne se sent pas fait pour le sport, n'en retire aucun bien-être	2,7
Age	3,2
Abandon suite à une blessure	2,0
Raisons financières, sport trop cher	1,5
Aucune offre adaptée	0,5
Mauvais souvenirs, expériences négatives	0,1
Temps de trajet entre le domicile et le lieu de travail	0,5
Horaires d'ouverture incompatibles, mauvaise accessibilité des installations sportives	0,5
Autres motifs	7,0

Remarque: nombre de personnes interrogées: 2687 (non-sportifs). Plusieurs réponses étaient possibles, ce qui explique que le total ne corresponde pas à 100 %.

T 9.2: Formes d'activité physique et sportive pratiquées par les non-sportifs

	Activités citées (en % des non-sportifs)	Age moyen (nbre d'années)	Part des femmes (en %)
Randonnée pédestre, randonnée en montagne	37,6	50	52
Natation	26,8	44	59
Cyclisme (hors VTT)	26,7	44	49
Ski alpin (hors randonnée à skis)	20,9	41	41
Promenade (sortie du chien)	9,6	49	60
Danse (y compris jazzdance)	6,4	42	75
Jogging, course à pied	5,4	37	53
Luge, bob	4,0	39	61
Football	2,9	31	9
Fitness, aérobic	2,5	48	65
Snowboard (hors randonnée)	2,2	25	43
Walking, nordic walking	1,9	51	79
VTT	1,7	38	17
Ski de fond	1,3	53	27
Randonnée à skis/en snowboard, raquettes à neige	1,0	48	34
Inline-skating, patinage sur roulettes	1,0	32	54

Remarque: nombre de personnes interrogées: 2687 (non-sportifs). Formes d'activité physique et sportive citées par au moins 1 % des non-sportifs.

40 % des non-sportifs souhaitent pratiquer ou reprendre un sport

De nombreux non-sportifs souhaiteraient par ailleurs intensifier ou mieux cibler leurs activités. Quelque 38 % d'entre eux déclarent ainsi vouloir reprendre un sport ou s'adonner à d'autres activités physiques. Ce chiffre est exactement le même qu'en 2008. De plus, 29 % des non-sportifs envisagent de s'inscrire/se réinscrire dans un club. Les sports visés ne sont ni particulièrement originaux, ni extrêmement prestigieux ou coûteux. La natation, le fitness, le cyclisme, la randonnée pédestre, le jogging, la danse, le yoga, la gymnastique et le ski alpin, soit des sports courants pouvant être pratiqués tout au long de la vie et auxquels s'adonnent souvent déjà ponctuellement les non-sportifs, figurent sur la liste (cf. tableau 9.3). Interrogés sur ce qui les empêche de commencer tout de suite, les non-sportifs invoquent des raisons liées à leur situation personnelle plutôt qu'à l'offre. La moitié d'entre eux pointe le manque de temps ou les contraintes professionnelles ou familiales, un cinquième leur état de santé. Quelque 10 % de non-sportifs seraient plus motivés si un ou une ami les accompagnait. Les autres motifs comme les raisons financières, l'absence d'une offre adaptée ou encore le besoin de recommandations médicales ou d'objectifs clairs, jouent en revanche un rôle secondaire.

Le désir de reprendre une activité sportive baisse avec l'âge. Il est plus marqué chez les femmes, chez les personnes bénéficiant d'un niveau de formation moyen à supérieur et parmi la population de nationalité étrangère. Parmi les femmes de nationalité étrangère, quelque 60 % souhaiteraient ainsi se mettre au sport.

T 9.3: Formes d'activité physique et sportive que les non-sportifs aimeraient pratiquer (ou pratiquer davantage) à l'avenir

	**Activités citées** (en % des non-sportifs)	**Age moyen** (nbre d'années)	**Part des femmes** (en %)
Natation	7,3	45	66
Fitness, aérobic	7,1	39	70
Cyclisme (hors VTT)	5,8	47	52
Randonnée pédestre, randonnée en montagne	4,6	52	62
Jogging, course à pied	3,2	37	53
Danse (y compris jazzdance)	2,9	36	94
Yoga, tai chi, qi gong	2,9	42	95
Gymnastique	2,7	54	81
Ski alpin (hors randonnée à skis)	2,4	44	34
Tennis	1,8	38	49
Sports de combat, autodéfense	1,6	28	43
Football	1,5	35	16
Walking, nordic walking	1,3	47	86
Musculation	1,0	44	18

Remarque: nombre de personnes interrogées: 2687 (non-sportifs). Formes d'activité physique et sportive citées par au moins 1 % des non-sportifs.

10. Lieux et horaires de pratique sportive

Les sentiers pédestres et les piscines sont les lieux les plus fréquemment usités, les salles de gymnastique et de sport publiques les lieux les plus régulièrement fréquentés

La plupart des pratiques sportives requièrent des infrastructures adaptées. Afin d'en apprendre davantage sur l'utilisation des infrastructures, une liste a été soumise aux participants de l'enquête en ligne, invités à préciser la fréquence et les lieux d'utilisation de diverses installations sportives (cf. graphique 10.1). Le plus souvent, le sport est pratiqué en plein air. Il n'est donc pas surprenant que les sports pouvant être pratiqués tout au long de la vie, comme la randonnée pédestre, le cyclisme, le ski alpin, le nordic walking, le walking ou le jogging, soient si prisés. S'ils nécessitent une infrastructure adaptée, ils se pratiquent en effet «en plein air». L'utilisation d'une infrastructure centrale pour la pratique d'un sport en plein air (sentiers pédestres balisés, p. ex.) arrive deuxième. Ce type d'infrastructure est utilisé par 80 % de la population suisse. Environ 80 % des sondés ont recours aux différents types d'infrastructures de baignade. Dans ce domaine, la part des utilisateurs ponctuels est toutefois sensiblement plus élevée que pour les sentiers pédestres. Les trois quarts des Suisses font au moins un peu de sport à domicile de temps à autre, jusqu'à plusieurs fois par semaine pour un quart d'entre eux. En termes de régularité, les salles de gymnastique et de sport publiques ainsi que les centres de fitness et de sport privés jouent un rôle important. Parmi la population suisse âgée de 15 à 74 ans, une personne sur cinq fréquente une salle de gymnastique ou de sport publique, une sur six un centre de fitness ou de sport privé.

G 10.1: Lieux de pratique et utilisation des infrastructures sportives (en % de la population résidante suisse âgée de 15 à 74 ans)

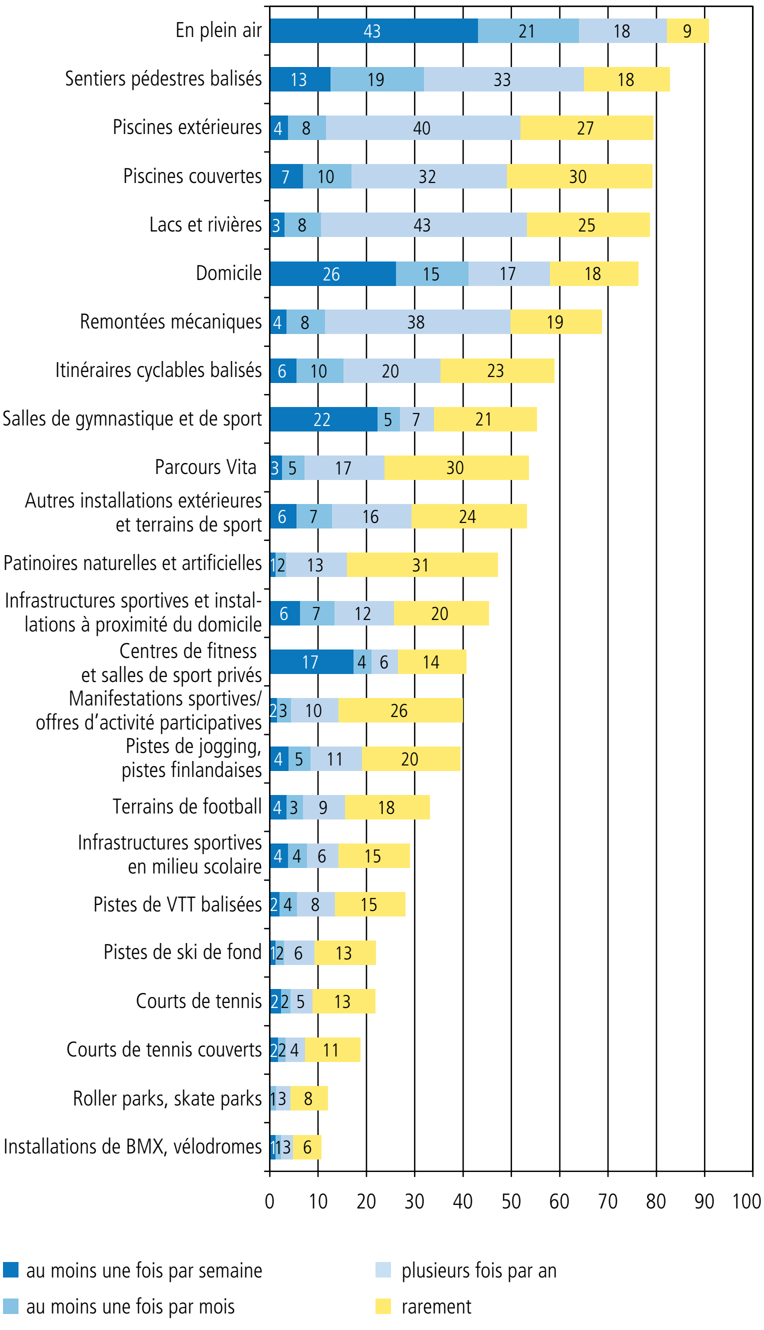

Remarque: nombre de personnes interrogées: 6686 (enquête en ligne).

T 10.1: Lieu d'utilisation d'une sélection d'infrastructures sportives (part de l'ensemble des utilisateurs d'une infrastructure donnée en %)

	Commune de résidence	Environnement proche	Autre endroit	Variable
Salles de gymnastique et de sport publiques	44	36	11	17
Terrains de football	40	35	9	26
Courts de tennis	29	38	16	23
Courts de tennis couverts	20	41	18	26
Piscines couvertes	24	48	16	20
Piscines en plein air	29	44	14	23
Centres de fitness et salles de sport privés	32	45	15	12

Remarque: réponses à la question «De manière générale, à quel endroit utilisez-vous cette infrastructure?» Plusieurs réponses étaient possibles, ce qui explique que le total ne corresponde pas à 100 %. Nombre de personnes interrogées: entre 1511 et 5517 selon l'infrastructure.

Une vaste offre d'infrastructures dans la commune de résidence et l'environnement proche

Les utilisateurs des différentes infrastructures sportives ont été invités à indiquer le lieu d'utilisation habituel de celles-ci. Le tableau 10.1 révèle que les salles de gymnastique et de sport publiques ainsi que les terrains de football sont souvent fréquentés dans la commune de résidence. Le fait qu'un bon tiers (36%) des personnes interrogées s'y rende à pied et que 28% fassent le trajet à vélo témoigne de la densité élevée de ces infrastructures. Un utilisateur sur cinq se rend dans les salles de gymnastique et de sport avec les transports publics, 44% utilisant un véhicule motorisé comme la voiture ou la moto. En règle générale, les courts de tennis indoor et outdoor ainsi que les centres de fitness et de sport privés sont plus éloignés. Cette règle s'applique aussi et surtout aux piscines couvertes et extérieures. La majorité de la population bénéficie cependant d'une offre adaptée dans son environnement proche.

T 10.2: Moment de la journée consacré au sport (part de sportifs en %)

	Tous les sportifs	Hommes	Femmes
Tôt le matin	9	9	8
Dans la matinée	28	35	20
A midi	10	9	11
L'après-midi	26	27	26
En soirée	56	53	60
Pendant la nuit	1	1	1
Variable	10	9	10

Remarque: La question posée était: «A quel moment de la journée faites-vous habituellement du sport?» Plusieurs réponses étaient possibles, ce qui explique que le total ne corresponde pas à 100 %. Nombre de personnes interrogées: 7964 (sportifs).

Des horaires de pratique qui varient selon le statut d'activité

Quelque 56 % des sportifs s'adonnent à leur sport en soirée (cf. tableau 10.2). Un bon quart fait du sport le matin et l'après-midi. Un sportif sur dix fait du sport très tôt le matin ou durant la pause de midi. Les femmes consacrent plus souvent la matinée au sport que les hommes. Les différences dépendent toutefois essentiellement de l'activité principale et du temps de travail. Les femmes actives à plein temps font surtout du sport le soir (68 %), plus rarement le matin (16 %). Ce moment de la journée est plus propice à la pratique d'un sport pour les travailleurs à temps partiel (35 %) et les femmes au foyer (51 %). Les retraités font souvent de l'exercice le matin (48 %) voire très tôt (13%), beaucoup moins en soirée (25 %). En revanche, les personnes en formation montrent une nette préférence pour le soir (74 %).

11. Sport en club

Un quart de la population fait du sport en club

La Suisse compte plus de 20 000 clubs sportifs, dont l'offre, vaste, est très utilisée. Comme on peut le voir sur le graphique 11.1, un quart de la population résidante suisse âgée de 15 à 74 ans fait du sport en club. Viennent s'ajouter 4 % de membres passifs. Au cours des 15 dernières années, la proportion de membres actifs n'a guère évolué dans les clubs sportifs (2000: 24 %, 2008: 25 %). En raison de la croissance démographique, le nombre de membres actifs dans les clubs a progressé depuis quelques années.

Il s'agit toujours d'une majorité d'hommes. Après une légère hausse entre 2000 et 2008, la proportion de membres actifs masculins est désormais constante (2000: 26 %, 2008: 31 %, 2014: 31 %). Chez les femmes, elle est stable depuis 15 ans (2000: 20 %, 2008: 19 %, 2014: 20 %). Le sport en club séduit surtout les jeunes adultes. Avec l'âge, la proportion de membres actifs recule. Cependant, la catégorie des 45-59 ans et celle des 60-74 ans comptent encore un bon cinquième de membres de clubs sportifs. A noter que la part de membres passifs n'augmente pas de façon significative avec l'âge.

G 11.1: Membres d'un club sportif selon le sexe, l'âge, la région linguistique, le revenu et la nationalité (en %)

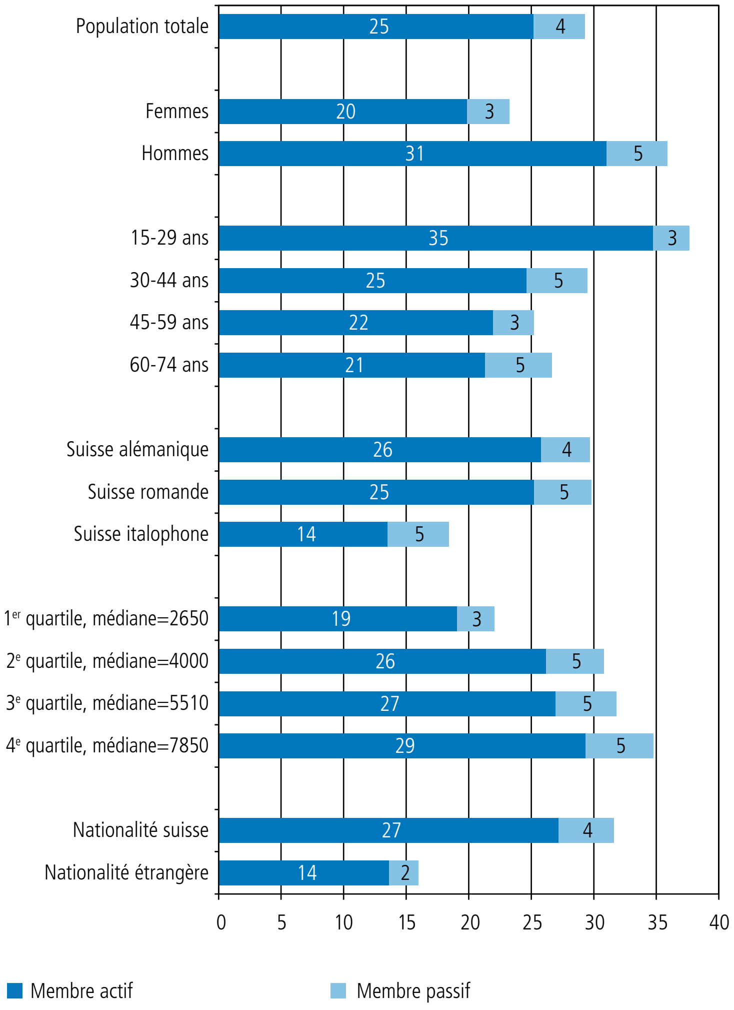

Remarque: nombre de personnes interrogées: 10 651; revenu du ménage (net mensuel en CHF): le revenu par équivalent adulte indique le budget disponible pour chaque membre du ménage. Les quartiles englobent quatre catégories de revenu d'égale importance: le 1er quartile représente le quart des personnes au revenu le plus faible, le 2e quartile le quart suivant, etc.

Moins de membres au Tessin et au sein de la population immigrée

Le sport associatif est aussi populaire en Suisse alémanique qu'en Suisse romande. Au Tessin, proportionnellement, le nombre de sportifs inscrits dans un club est beaucoup moins important. Depuis 2008, le sport en club a légèrement gagné du terrain en Suisse romande (membres actifs en 2008: 22 %, 2014: 25 %), mais il a reculé en Suisse italophone (membres actifs en 2008: 19 %, 2014: 14 %).

Plus le revenu est élevé, plus la probabilité d'adhésion à un club sportif augmente. La principale différence réside entre la tranche de revenu inférieure et les trois catégories supérieures. On observe par ailleurs des disparités flagrantes selon la nationalité. Les Suisses fréquentent presque deux fois plus les clubs sportifs que les étrangers. Comme pour l'activité sportive, on constate des différences majeures selon la région d'origine: la population immigrée issue d'Europe de l'Ouest et du Nord compte 19 % de membres actifs contre une proportion nettement inférieure pour les personnes provenant d'autres régions (Europe du Sud: 9 %, Balkans/Turquie/Europe du Sud: 14 %, hors Europe: 13 %). Au sein de la population immigrée, ce sont surtout les hommes jeunes qui fréquentent activement les clubs sportifs.

Les clubs sportifs représentent l'essentiel de l'offre

Les chiffres suivants permettent d'appréhender le poids des clubs dans le paysage de l'offre: 61 % des sportifs ont été membres d'un club par le passé, même s'ils ne le sont plus aujourd'hui. Autrement dit, les clubs sportifs jouent un rôle important en termes de socialisation par le sport. Généralement, les personnes qui font du sport en club se distinguent par une activité sportive élevée: 84 % des personnes qui fréquentent un club pratiquent leur sport au moins deux heures par semaine. Il n'est pas rare qu'elles soient membres de plusieurs clubs à la fois (deux clubs: 20 %, trois clubs ou plus: 6 %). Les hommes sont plus concernés par cette tendance (27 %) que les femmes (19 %).

La camaraderie et la convivialité: des motivations déterminantes pour l'adhésion à un club

Les principales raisons qui sous-tendent la pratique d'un sport en club sont la camaraderie et la convivialité (46 % des membres actifs) ou la compagnie d'amis et de collègues (31 %). Outre les motivations sociales, la régularité (22 %) de l'entraînement, le fait que le sport concerné se pratique essentiellement en club (21 %) ainsi que la qualité des prestations et de l'entraîneur (14 %) sont des arguments décisifs en faveur de l'adhésion à un club. La possibilité de participer à des compétitions et de se mesurer à d'autres (8 %), la tradition et l'habitude (6 %), un bon rapport prix-performance (3 %) ou encore la proximité géographique (2 %) recueillent moins de voix. On n'observe pas de disparités flagrantes entre les sexes: les femmes évoquent un peu plus la compagnie des autres, la régularité de l'entraînement et le caractère économique de l'offre. Les hommes s'intéressent davantage à l'aspect compétition et mettent en avant le fait que leur sport est essentiellement pratiqué en club.

Une offre très vaste et aucun signe de lassitude

Les sports en club les plus prisés sont la gymnastique, le football, le tennis, les sports de combat comme le kick-boxing ou la boxe thaï, le judo, le ju-jitsu et le karaté, mais aussi le volleyball, l'unihockey, le tir, le ski alpin, le hockey sur glace, la natation et le golf. Le fitness/l'aérobic, le yoga/tai chi/qi gong et la danse sont surtout pratiqués dans les centres de fitness ou dans d'autres infrastructures privées. On trouve parmi les pratiquants de ces sports une proportion considérable de personnes qui s'y adonnent en club. Comme le ski alpin, les randonnées sont principalement organisées individuellement. Grâce à la forte popularité de la randonnée et à l'offre des clubs en la matière, cette activité compte néanmoins aussi parmi les sports associatifs préférés. Parmi les sports affichant le plus fort taux de pratiquants membres d'un club, citons la balle au poing et la balle au panier (96 %), le curling (94 %), le judo et le ju-jitsu (84 %), la course d'orientation (82 %), le tir (79 %), le handball (70 %), la lutte suisse (70 %) et l'athlétisme (64 %).

Un bon tiers (37 %) des personnes qui ne sont pas membres d'un club sportif actuellement n'exclut pas de le (re)devenir. Parmi les sportifs non-membres d'un club, 40 % ne sont pas opposés à le devenir, un chiffre qui atteint tout de même 29 % pour les non-sportifs. En outre, plus de la moitié des membres passifs (53 %) envisage de participer activement au fonctionnement du club.

De nombreux engagements volontaires

Un membre actif sur deux ne se contente pas de faire du sport en club, mais s'est aussi engagé volontairement au cours des 12 derniers mois en exerçant une fonction à titre bénévole ou comme personnel auxiliaire (cf. tableau 11.1). Il s'agit généralement d'assurer une fonction au sein du club ou de contribuer aux services auxiliaires de celui-ci. D'autres activités extérieures au club peuvent prendre la forme d'un engagement volontaire dans un événement sportif.

L'engagement volontaire n'est pas réservé aux membres actifs. Un tiers de membres passifs et un non-membre sur dix se sont ainsi volontairement engagés en faveur du sport au cours des 12 derniers mois. Une personne sur huit qui se mobilise en faveur du sport cite plusieurs formes d'engagement en ce sens. Le tableau 11.1 révèle que les hommes occupent plus généralement une fonction au sein du club, les femmes intervenant plutôt comme personnel auxiliaire.

Les personnes qui exercent une fonction au sein d'un club interviennent le plus souvent comme entraîneur ou comme moniteur. Aux côtés des éducateurs sportifs et des moniteurs pour adultes et seniors, près de la moitié de ces personnes assurent une fonction d'entraînement. Deux personnes sur cinq engagées à titre bénévole interviennent dans la direction d'un club, 5 % en tant qu'arbitre. Une sur quatre cite d'autres fonctions comme la direction d'événements au sein du club ou la contribution à l'organisation de manifestations sportives. Près d'un bénévole sur cinq remplit deux ou plusieurs fonctions.

T 11.1: Engagement bénévole et volontaire dans le sport

	Tous		Femmes		Hommes	
	en % de la population	en % de membres actifs	en % de la population	en % de membres actifs	en % de la population	en % de membres actifs
Fonction exercée au sein du club	7	25	5	22	10	27
Fonction exercée en dehors du club	2	4	1	4	2	5
Service auxiliaire du/pour le club	10	27	9	28	12	26
Service auxiliaire en dehors du club	6	9	5	9	7	8
Engagement volontaire dans le sport	22	52	18	51	26	54

Remarque: nombre de personnes interrogées: 10 651 ou 2761 (membres actifs). Plusieurs formes d'engagement peuvent être déclarées.

Le bénévolat implique un investissement élevé, non rémunéré

L'investissement en temps associé à une activité bénévole représente en moyenne 2,5 heures par semaine. Cette valeur est en léger recul depuis 2008 (2,8 heures). Elle varie cependant considérablement d'un club et d'une fonction à l'autre. Elle s'élève ainsi à 10 heures et plus pour 4 % des personnes exerçant une fonction au sein d'un club. A côté de cela, 9 % déclarent que la fonction qu'ils assument à titre bénévole prend très peu de leur temps. La moitié des bénévoles n'intervient pas plus d'une heure et demie par semaine. Indépendamment de la charge de travail, pour l'essentiel, cet engagement n'est pas rétribué: plus de deux tiers (69 %) de bénévoles ne touchent absolument aucune indemnité. Un bon quart (28 %) perçoit une rémunération partielle. Enfin, 3 % sont en grande partie, voire entièrement défrayés. Généralement, l'indemnité ne dépasse pas 1000 francs par an. Seuls 4 % des personnes qui exercent une fonction donnée ou 14 % de l'ensemble des intervenants rétribués perçoivent plus de 1000 francs.

Le temps d'investissement du personnel auxiliaire (au sein du club et à l'extérieur) et des personnes qui exercent des fonctions en dehors du club est de 1,1 heure par semaine en moyenne. La moitié des personnes qui s'engagent dans ce domaine ne s'investit pas plus d'une demi heure par semaine. Une grande majorité de ces activités (89 %) est entièrement bénévole.

Le plaisir procuré par l'activité et les motivations d'ordre social sont les principales raisons qui sous-tendent un engagement volontaire

Interrogées sur la raison de leur engagement dans le club sportif, les bénévoles citent majoritairement le plaisir procuré par leur activité. Pour de nombreux bénévoles, les motivations sociales jouent également un rôle majeur: à travers son engagement, un bénévole sur quatre souhaite apporter son soutien au club ou à ses collègues au sein du club. Un sur cinq met en avant la collaboration avec autrui et la possibilité de faire œuvre utile sur le plan social. Quelque 11 % de bénévoles déclarent qu'ils étaient seuls à vouloir assurer leur fonction. Le plaisir lié aux responsabilités endossées et au pouvoir de décision, la reconnaissance et l'image véhiculée par la fonction occupée ainsi que les bénéfices retirés pour d'autres champs d'activité dans un cadre professionnel ou politique sont plus rarement mentionnés. Les considérations financières ne constituent pas une motivation centrale de l'exercice d'une fonction au sein d'un club sportif.

Un degré de satisfaction élevé et un potentiel inexploité

Interrogés sur le degré de satisfaction que leur procure leur engagement volontaire en faveur du sport, les bénévoles et le personnel auxiliaire se disent (très) satisfaits à 91 %, partiellement satisfaits à 7 % et (plutôt) insatisfaits à 2 %. En moyenne, les personnes qui exercent une fonction au sein d'un club sont plus satisfaites que celles qui assurent une fonction en dehors du club ou interviennent comme personnel auxiliaire. Neuf bénévoles sur dix qui exercent une fonction au sein d'un club ne changeraient rien si c'était à refaire: 70 % sans aucune réserve, 20 % sous certaines conditions.

De plus, un tiers (32 %) des membres actifs qui n'occupent actuellement aucune fonction au sein de leur club envisagent de le faire prochainement. Une bonne moitié d'entre eux (56 %) n'a jamais été sollicitée par le club dans ce but. Ceux qui l'ont été et ont décliné la proposition l'ont fait principalement par manque de temps (56 %) ou pour privilégier d'autres intérêts ou fonctions (9 %). Le manque de temps est également la première raison invoquée par les membres actifs qui n'envisagent absolument pas le bénévolat dans un avenir proche (51 %). Les autres obstacles sont l'âge (trop vieux/trop jeune: (19 %), d'autres intérêts, fonctions et obligations (12 %) ainsi que le sentiment de ne pas avoir les qualités requises (6 %).

12. Sport dans les centres de fitness

Une croissance constante de l'activité des centres de fitness privés

Outre les clubs sportifs, les centres de fitness privés se sont imposés comme des prestataires sportifs majeurs en Suisse: 16 % de la population suisse sont membres d'un centre de fitness privé ou ont souscrit un abonnement qui leur en ouvre les portes régulièrement (abonnement annuel ou mensuel). La part des membres a encore légèrement progressé depuis 2008 (14 %). Contrairement aux clubs de sport, qui attirent une majorité d'hommes, les centres de fitness sont un peu plus appréciés des femmes (graphique 12.1). Plus l'âge augmente, plus le nombre d'adhésions baisse. Chez les plus jeunes, on compte deux fois plus de personnes qui fréquentent un centre de fitness que dans la tranche des 60-74 ans. Plus le revenu est élevé, plus la probabilité d'adhésion à un centre de fitness augmente. Dans la catégorie de revenu supérieure, une personne sur quatre possède un abonnement, contre une sur dix dans la tranche inférieure. Les écarts entre les différentes catégories de revenu sont plus marqués que pour les clubs sportifs. En revanche, on ne relève aucune différence de comportement entre les Suisses et les étrangers.

Les avantages par rapport à la concurrence: une grande flexibilité, peu de contraintes et des horaires d'ouverture étendus

En plus des cours de fitness, de la musculation et de l'entraînement physique, les centres de fitness proposent également des séances d'aérobic, de zumba, de pilates, de yoga, de spinning, de danse, de gymnastique (du dos), d'aqua-fitness ou encore de natation. Une grande flexibilité (citée par 22 % des membres), l'absence de contraintes (21 %), des horaires d'ouverture plus adaptés (15 %) et la meilleure qualité de l'offre (10 %) sont les principaux avantages qui sous-tendent une adhésion à un centre de fitness. La qualité de l'encadrement (9 %), l'infrastructure (6 %) et l'accessibilité (2 %) sont plus rarement évoqués. Alors que les hommes s'attachent davantage à l'absence de contraintes et à la qualité de l'encadrement, les femmes sont plus sensibles à la flexibilité et aux horaires d'ouverture.

Des offres ouvertes à tous qui séduisent avant tout les femmes

Outre les clubs et les centres de fitness, il existe d'autres prestataires privés qui offrent la possibilité de pratiquer et de s'initier à différents sports et activités physiques. En Suisse, près d'une personne sur dix a recours à ce type d'offre, qui séduit une nette majorité de femmes (14 % contre 4 % d'hommes). Dans ce cadre, le yoga et la danse sont les principales activités pratiquées. Ces dernières comptent également le pilates, la gymnastique (du dos), la zumba, l'aqua-fitness, le tennis, le fitness, le golf, la randonnée pédestre et divers sports de combat.

Enfin, 7 % de la population résidante ont recours à différentes offres sportives ouvertes à tous. Deux fois plus de femmes que d'hommes se tournent vers ces offres (10 % contre 5 % d'hommes). L'aqua-fitness, la randonnée pédestre et la gymnastique sont les plus prisées. Le jogging, le football, le fitness, le nordic walking, le walking, la natation et le cyclisme séduisent également beaucoup de sportifs.

G 12.1: Proportion de membres d'un centre de fitness selon le sexe, l'âge, la région linguistique, le revenu et la nationalité (en %)

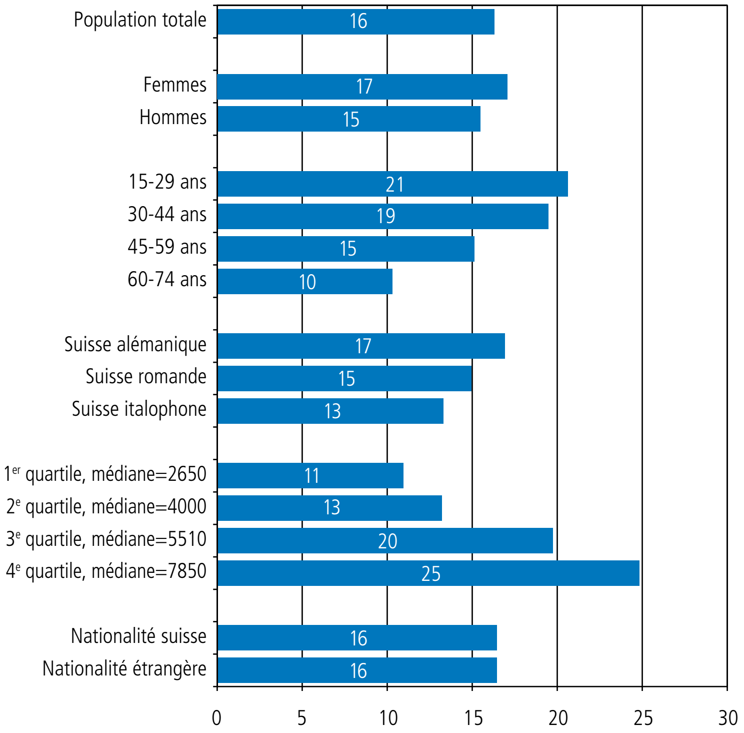

Remarque: nombre de personnes interrogées: 10 649; revenu du ménage (net mensuel en CHF): le revenu par équivalent adulte indique le budget disponible pour chaque membre du ménage. Les quartiles englobent quatre catégories de revenu d'égale importance: le 1er quartile représente le quart des personnes au revenu le plus faible, le 2e quartile le quart suivant, etc.

13. Sport et vacances

Les vacances sportives sont très appréciées des Suisses

De nombreux Suisses ne se contentent pas de pratiquer un sport le matin ou le soir, mais mettent également à profit leurs congés pour se consacrer plus longuement et de façon plus intensive à leur activité, dans un lieu dédié et agréable. La combinaison du sport et des vacances est une stratégie gagnante: on trouve aujourd'hui d'innombrables offres dans des lieux très divers, qui concernent des sports très différents et séduisent de nombreux sportifs. Quelque 40 % de la population suisse ont passé des vacances sportives au cours des 12 derniers mois (cf. graphique 13.1). Ont été comptabilisés tous les séjours de plus d'une journée mettant les activités sportives au premier plan. Sans surprise, ce type de vacances se déroule beaucoup plus souvent en Suisse qu'à l'étranger.

Les femmes et les hommes en consomment à peu près autant. Les hommes se rendent toutefois un peu plus souvent à l'étranger. Les vacances sportives sont appréciées dans toutes les classes d'âge. Ainsi, un tiers des 60-74 ans a passé des vacances sportives au cours des 12 derniers mois. Ce type de séjour est plus populaire en Suisse alémanique qu'en Suisse romande et italophone.

Le choix de vacances sportives, l'intensité de l'activité et la destination sont étroitement liés au revenu: plus le revenu du ménage est élevé, plus les vacances sportives sont fréquentes, le temps de séjour est long et la destination choisie a tendance à être à l'étranger. Les vacances sportives sont moins répandues parmi les résidents étrangers. Dans la moitié des cas, cette frange de la population se rend à l'étranger.

Les vacances sportives passées en Suisse représentent un total de 9,4 nuitées par an, contre 1,5 pour les séjours à l'étranger. En extrapolant ces valeurs à l'ensemble de la population, on aboutit à la conclusion que chaque habitant consomme des vacances sportives à raison de 5,6 nuitées par an, dont 3,1 passées en Suisse et 2,5 à l'étranger.

G 13.1: Le sport en vacances (proportion de personnes ayant dédié leurs vacances au sport au cours des 12 derniers mois, une nuitée min., en %)

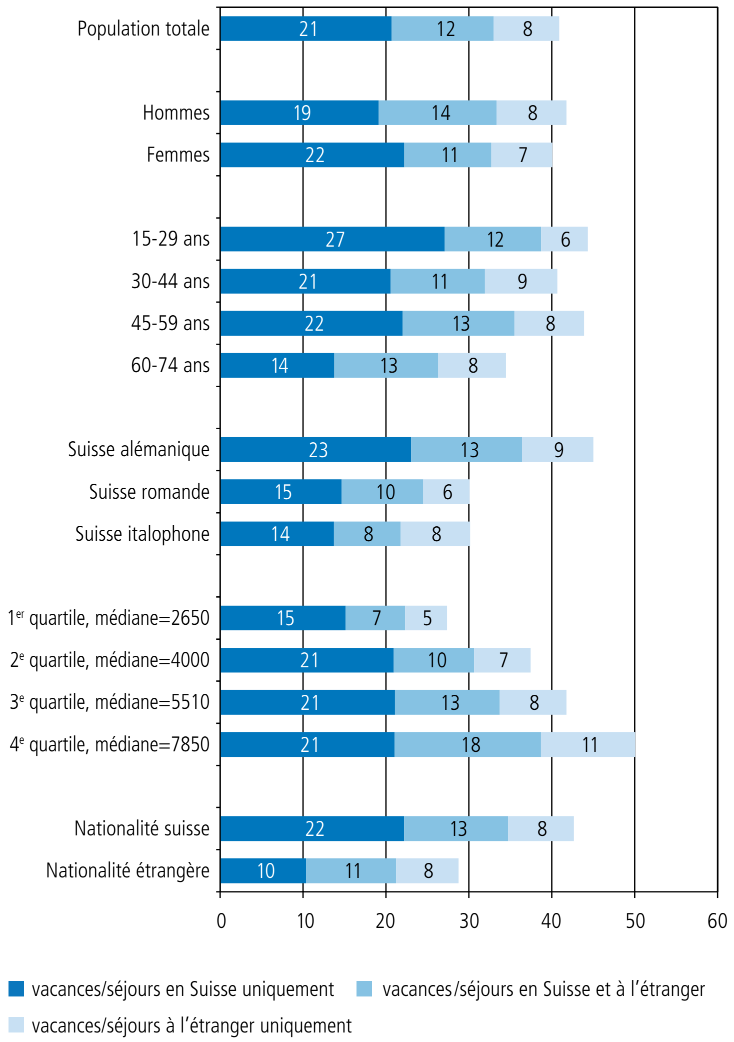

Remarque: nombre de personnes interrogées: 6397 (enquête en ligne); revenu du ménage (net mensuel en CHF): le revenu par équivalent adulte indique le budget disponible pour chaque membre du ménage. Les quartiles englobent quatre catégories de revenu d'égale importance: le 1er quartile représente le quart des personnes au revenu le plus faible, le 2e quartile le quart suivant, etc.

Les grands classiques: les vacances dédiées au ski et à la randonnée

Le tableau 13.1 fournit un aperçu des principaux sports pratiqués pendant les séjours sportifs. Premier constat flagrant: l'étendue de la palette d'offres et des possibilités offertes. Ensuite, on observe que les deux grands classiques suisses que sont le ski et la randonnée pédestre arrivent en tête des sports pratiqués. Près d'un quart de la population totale est parti en vacances de ski au cours des 12 derniers mois. A noter, qu'il peut s'agir d'un week-end de ski, dans la mesure où la condition à remplir est de passer plus d'une nuit sur place. Une personne sur six résidant en Suisse fait de la randonnée pédestre pendant ses vacances. La natation, le cyclisme, le jogging, randonnée à skis, les raquettes à neige et le VTT figurent aussi parmi les activités prisées pendant les vacances.

T 13.1: Principaux sports pratiqués dans le cadre de vacances sportives

	Activités citées (en % de la population)	Age moyen (nbre d'années)	Part des femmes (en %)
Ski/ski alpin	23,1	44	49
Randonnée pédestre/ randonnée en montagne	17,6	49	53
Natation	10,0	48	53
Cyclisme/randonnée à vélo	6,7	48	50
Jogging/marche/running	6,5	42	42
Ski de fond	4,4	48	52
Randonnée à skis/ raquettes à neige	4,1	49	62
VTT	4,0	44	28
Fitness/aérobic	3,4	43	44
Snowboard	3,3	27	54
Escalade/alpinisme	2,8	42	41
Randonnée à skis/ en snowboard	2,6	42	46
Nordic walking, walking	2,6	54	66
Plongée/snorkeling	2,3	44	47
Tennis	2,2	42	41
Football	1,8	31	31
Golf	1,7	53	36
Vélo de course	1,5	47	21
Voile	1,5	46	46
Danse	1,4	42	53
Moto	1,4	43	27
Volleyball/beach-volley	1,4	30	48
Yoga/tai chi/qi gong	1,2	47	77
Canoë/kayak/rafting	0,8	49	48
Surf	0,8	31	51
Planche à voile, kitesurf	0,8	37	39
Équitation/autres sports équestres	0,7	36	71
Sports de combat/ autodéfense	0,5	33	31
Delta plane/parapente/ montgolfière	0,4	43	42
Aviron	0,3	38	17
Planeur/avion motorisé/ vol à voile	0,2	46	21
Autres sports	2,7	39	38

Remarque: nombre de personnes interrogées: 6397 (enquête en ligne)

14. Blessures et accidents liés au sport

Les hommes sont plus souvent victimes d'accidents de sport que les femmes

Le sport n'est pas uniquement synonyme de plaisir, de détente, de convivialité et d'expériences uniques; il a également un revers: les accidents. Selon les statistiques du Bureau de prévention des accidents (bpa) et du Service de centralisation des statistiques de l'assurance-accidents LAA (SSAA), au sein de la population résidante suisse, plus de 400 000 personnes sont chaque année victimes d'un accident lié au sport.

Au cours des 12 mois précédant l'entretien, 8 % des personnes interrogées ont été victimes d'une blessure liée au sport, qui a nécessité un traitement médical ou une hospitalisation (cf. tableau 14.1). De façon générale, une seule blessure a été évoquée; 12 % en ont déclaré deux et 7 % trois ou plus. La proportion d'hommes blessés au cours de l'année écoulée pendant la pratique d'un sport est de 10 %, contre 6 % de femmes.

Comme le montre le graphique 14.1, la part de blessés recule sensiblement avec l'âge. Elle est particulièrement élevée chez les hommes jeunes. Parmi ces derniers, près d'une personne sur cinq s'est blessée au cours de l'année en faisant du sport. On observe des différences entre les sexes et les différentes classes d'âge en termes de comportement sportif et de prise de risque. Les écarts selon l'âge et le sexe sont restés stables depuis 2008, de même que la proportion de blessés dans la population totale.

Plus une personne fait de sport, plus elle a tendance à se blesser. Parmi les sportifs très actifs qui s'entraînent au moins trois heures par semaine, on relève ainsi 13 % de blessés chaque année, contre 6 % pour la catégorie des non-sportifs. Enfin, parmi ces derniers, 2 % se blessent chaque année au cours d'une activité sportive pratiquée ponctuellement. Les blessures des non-sportifs sont cependant généralement plus graves et débouchent sur une durée d'hospitalisation plus longue.

T 14.1: Blessures ayant nécessité des soins médicaux

		en % de la population	en % de blessés
Blessure survenue au cours des 12 derniers mois	Blessure	8,0	100,0
	Pas de blessure	92,0	
Survenance de la blessure	Blessure soudaine	6,4	79,4
	Blessure chronique/ ancienne	1,5	19,2
	Les deux/indéterminé	0,1	1,4

Remarque: nombre de personnes interrogées: 10 649

G 14.1: Proportion de personnes victimes d'une blessure survenue dans le cadre de la pratique d'un sport au cours des 12 derniers mois selon l'âge et le sexe (uniquement blessures ayant nécessité un traitement médical, en %)

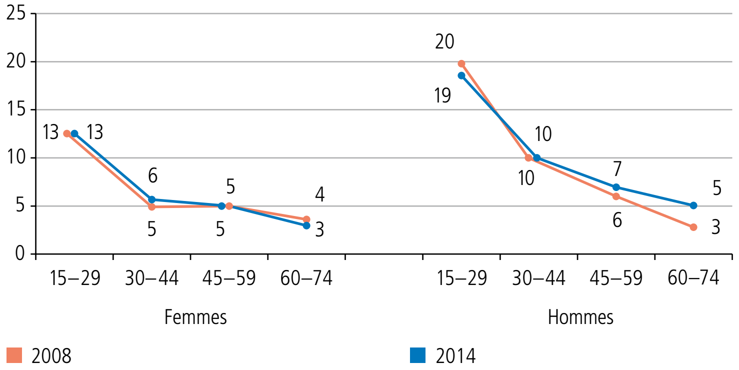

Remarque: nombre de personnes interrogées: 10 649

Le football et le ski alpin à l'origine du plus grand nombre d'accidents

En toute logique, les sports les plus fréquemment pratiqués provoquent plus d'accidents, football et ski en tête. Comptant de nombreux aficionados, le jogging, le cyclisme, le fitness et la randonnée pédestre ne sont pas en reste. En rapportant le nombre de blessés à celui de pratiquants, il apparaît que les sports de ballon comme le football, le handball, le basketball, le rugby, le football américain ou la balle au poing, mais aussi le hockey sur glace, la gymnastique artistique et les différents sports de combat, présentent un risque de blessure relativement élevé, avec plus d'un blessé sur dix pratiquants. Pour le (beach-)volley, l'équitation et l'unihockey, le chiffre est de près d'un blessé sur dix pratiquants. La randonnée pédestre, le ski de fond, l'aqua-fitness, le yoga/tai chi/qi gong, le nordic walking, le walking, la natation, les raquettes à neige et la gymnastique sont les activités les moins risquées, avec moins d'un blessé sur 100 pratiquants.

Quatre personnes sur cinq qui se blessent au cours d'une pratique sportive sont victimes d'un accident proprement dit, survenu soudainement. Une blessure sur cinq concerne des problèmes (chroniques) faisant suite à une pratique excessive ou à une ancienne blessure (tableau 14.1).

Fort heureusement, la majorité des blessures liées au sport sont légères: l'hospitalisation n'a été nécessaire que pour une fraction de blessés, une majorité d'entre eux ayant pu regagner leur domicile au bout d'un maximum de trois jours. Quatre blessés sur dix ont été provisoirement en arrêt de travail, pour une durée moyenne d'un mois (29 jours). Environ 40 % d'entre eux ont été en arrêt de travail entre 1 et 7 jours ou entre 8 et 31 jours et 20 % plus d'un mois.

15. Intérêt porté aux sports médiatiques

Un intérêt appuyé pour le sport malgré une tendance à la saturation

La population suisse passe la majorité de son temps libre, non pas à faire du vélo ou de la randonnée pédestre ni même de la natation ou encore du ski, mais devant un écran de télévision ou d'ordinateur. Elle consacre également beaucoup de temps à lire des journaux ou des magazines. Le sport joue aussi un rôle majeur dans la consommation médiatique de la population. La télévision a ainsi été désignée comme le matériel de sport préféré des Suisses. La population suisse manifeste un grand intérêt pour le sport (cf. tableau 15.1); seul un cinquième de la population déclare ne pas s'intéresser à l'actualité sportive. Avec l'essor massif des reportages et des comptes rendus sportifs, le sport a suscité un véritable engouement jusqu'au début des années 2000. Depuis quelque temps, on assiste toutefois à une certaine saturation: de 2008 à 2014, le pourcentage de personnes qui manifeste un grand intérêt pour le sport est en léger recul.

T 15.1: Intérêt de la population suisse pour le sport en 2000, 2008 et 2014 (en % de la population résidante suisse âgée de 15 à 74 ans)

	2000	2008	2014
Grand intérêt	32,5	35,2	31,3
Intérêt moyen	45,1	45,0	47,2
Aucun intérêt	22,4	19,8	21,4

Remarque: nombre de personnes interrogées: 2000: 2063; 2008: 10 255; 2014: 10 625

G 15.1: Intérêt manifesté pour le sport selon l'activité sportive, le sexe, l'âge, la région linguistique, la nationalité et le revenu (en %)

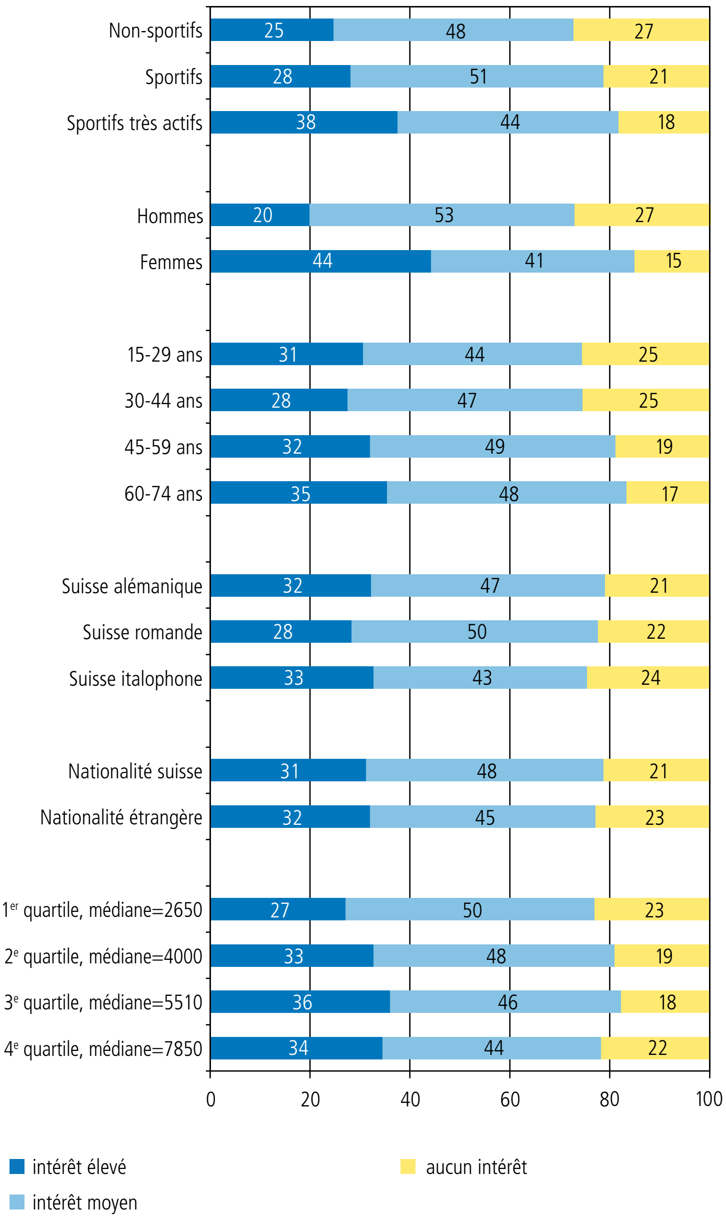

Remarque: nombre de personnes interrogées: 10 625. Revenu du ménage (net mensuel en CHF): le revenu par équivalent adulte indique le budget disponible pour chaque membre du ménage. Les quartiles englobent quatre catégories de revenu d'égale importance: le 1er quartile représente le quart des personnes au revenu le plus faible, le 2e quartile le quart suivant, etc.

Les sports médiatiques ne connaissent pas de frontières sociales

La recette du succès consiste à fédérer des catégories de population très variées. Comme l'illustre le graphique 15.1, les non-sportifs aussi suivent l'actualité sportive. Si les sportifs très actifs se démarquent légèrement, les trois quarts des inactifs s'intéressent néanmoins au sport. A contrario de l'activité sportive, au niveau de la consommation sportive, on ne constate pas de différence entre les Suisses de souche et les résidents étrangers. De même, la consommation médiatique est moins influencée par le statut social (déterminé par le niveau de revenu ou de formation – non représenté ici – et par la catégorie socioprofessionnelle) et la région linguistique que l'activité sportive.

On ne distingue que deux facteurs d'influence notables sur la totalité des critères qui jouent potentiellement un rôle: l'âge et, surtout, le sexe. Les seniors et les hommes s'intéressent un peu plus au sport que les plus jeunes et les femmes. Ce sont donc majoritairement les hommes qui suivent le sport dans les médias. Tandis que les femmes ont rattrapé les hommes en termes d'activité sportive depuis 20 ans, les différences relevées au niveau de l'intérêt manifesté pour le sport sont restées stables. Les écarts entre les femmes et les hommes sont exactement les mêmes qu'en 2000. Les trois quarts des femmes suivent l'actualité sportive. On recense néanmoins deux fois plus d'hommes que de femmes parmi les personnes qui s'intéressent aux sports médiatiques.

Les principales sources d'information sont la télévision et la presse écrite

L'analyse des modes de diffusion de l'actualité sportive (cf. graphique 15.2) fait apparaître que les quotidiens, les émissions télévisées et les comptes rendus sportifs à la radio et sur Internet sont les principales sources d'information. Les deux tiers de la population consultent au moins une fois par semaine le cahier des sports de leur quotidien, un quart le lisant même chaque jour. Plus de la moitié de la population regarde une émission consacrée au sport à la télévision au moins une fois par semaine. Les retransmissions d'événements en direct attirent également un large public.

G 15.2: Utilisation des sources d'information et fréquentation des manifestations sportives (en % de la population résidante suisse âgée de 15 à 74 ans)

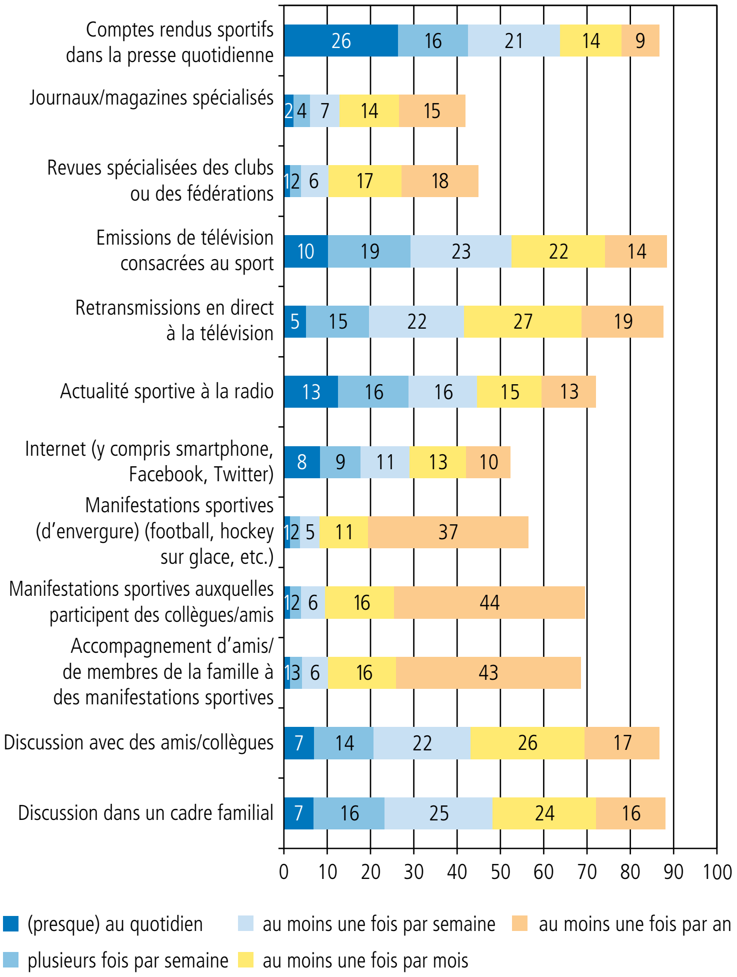

Remarque: nombre de personnes interrogées: 6717 (enquête en ligne)

Au regard de l'investissement que cela représente, logiquement, les Suisses se déplacent plus rarement pour assister à des événements sportifs. Un quart de la population fait toutefois le déplacement une fois par mois pour assister à une manifestation sportive et encourager des collègues ou des amis qui y participent, ou encore pour accompagner des membres de leur famille. A peine un cinquième de la population assiste une fois par mois à des manifestations d'envergure telles que des matches de football ou de hockey sur glace au plus haut niveau. Une bonne moitié de la population se rend dans une grande manifestation sportive une fois par an.

Contrairement à des pays comme la France ou l'Italie, en Suisse, les journaux et les magazines spécialisés dans le sport ne servent de support pour suivre l'actualité sportive quotidienne que de façon très marginale. Cette presse (à l'instar des journaux publiés par les clubs et les fédérations) s'adresse à un public d'initiés qui s'intéresse à un sport en particulier. Qui plus est, le sport est un sujet de discussion très apprécié, qui fait l'objet de fervents débats entre amis ou dans le cercle familial.

L'Internet progresse au détriment de la radio et de la presse écrite sportive

Les différentes sources d'information utilisée pour suivre l'actualité sportive n'ont guère évolué depuis 15 ans, à deux exceptions près. L'une d'entre elles est l'Internet, qui a connu un formidable essor en tant que source d'information. En 2000, seuls 8 % de la population consultaient l'actualité sportive sur Internet une fois par semaine. L'autre exception concerne les retransmissions d'événements en direct à la télévision, également en hausse depuis 2000. A noter que le développement de l'Internet ne se fait pas au détriment de la presse écrite sportive quotidienne, aussi consultée qu'il y a 15 ans. Par contre, les journaux spécialisés dans le sport et la radio attirent moins de lecteurs et d'auditeurs.

Le football domine nettement

Le football arrive clairement en tête des sports de prédilection de la population, suivi du ski alpin, du tennis et du hockey sur glace (cf. tableau 15.2). Entre 5 % et 10 % de la population suivent également l'athlétisme, le ski nordique, le cyclisme et les sports mécaniques. Arrive ensuite une longue liste de sports, qui ne sont toutefois suivis que par une petite minorité de personnes. Il semblerait que l'absence d'informations sur ces sports n'explique que partiellement ce constat. A la question de savoir quels sont les sports sur lesquels ils souhaiteraient obtenir davantage d'informations, deux cinquièmes des sondés citent plusieurs sports, parmi lesquels on retrouve étonnamment les stars comme le football, le ski alpin, le tennis et le hockey sur glace. On peut en conclure que les consommateurs de sport souhaitent en apprendre plus sur des sports qui bénéficient déjà d'une large couverture médiatique. Un grand nombre d'autres sports a toutefois été cité, ce qui indique une demande accrue d'informations dans ce domaine. Cela dit, l'étude n'a pas permis d'isoler un sport pour lequel l'écart entre la réalité et les attentes soit particulièrement important.

A noter qu'il existe des préférences liées à l'âge et au sexe: tandis que les femmes sont plus attirées par le patinage (fréquentation féminine à 95 %), la natation (79 %), l'équitation (72 %) et la gymnastique artistique (70 %), les hommes se tournent davantage vers les sports mécaniques, le rugby, le football américain, la boxe, le freestyle, le cyclisme, le VTT, le golf et le handball. Pour ce qui est des écarts liés à l'âge: tandis que l'âge moyen des amateurs de snowboard, de boxe, de freestyle, de volleyball et de basketball est plutôt bas, il est supérieur à la moyenne du côté des spectateurs du curling, du cyclisme, du ski nordique, du patinage et du golf.

Le suspense, des stars et la participation des Suisses sont les principaux moteurs

Interrogés sur les raisons pour lesquelles ils s'intéressent au sport, les sondés citent principalement le suspense (55 % de la population), les performances de stars comme Roger Federer (51 %), l'intérêt pour les résultats dans certains sports (50 %), la participation des Suisses (48 %), la technique et le talent (46 %) ainsi que l'investissement et l'endurance des sportifs (33 %). En revanche, la mise en scène et l'ambiance (14 %) jouent un rôle annexe. Parmi les autres motivations fréquemment évoquées figurent les raisons d'ordre personnel. Il peut s'agir d'un sport anciennement pratiqué (28 %) ou de la volonté de soutenir un membre de la famille ou des amis (21 %). Un dixième des amateurs de sport déclare que la couverture médiatique ou le fait d'avoir assisté à des compétitions constitue une motivation très importante pour faire du sport. Un cinquième d'entre eux sont un peu moins influencés en ce sens. Les deux tiers restants de la population ne sont pas influencés par les événements sportifs, qui ne les poussent pas à entamer des activités sportives.

La moitié de la population est ravie des succès remportés par les sportifs ou les équipes suisses dans les compétitions internationales comme les Jeux Olympiques et les championnats d'Europe ou du monde. Un quart s'en réjouit au moins un peu. Les personnes qui s'intéressent au sport sont donc généralement heureuses des succès remportés par les sportifs suisses. Un quart de la population déclare être fan d'une équipe ou d'un sportif en particulier. Quelque 8 % de la population possèdent une carte d'abonnement annuelle ou saisonnière pour une équipe donnée, 4 % étant membres d'un fan-club. Enfin, 7 % ont souscrit un abonnement à une chaîne payante spécialisée dans le sport (Teleclub Sport, p. ex.).

T 15.2: Sports pour lesquels la population manifeste le plus d'intérêt (en % de la population résidante suisse âgée de 15 à 74 ans)

	Intérêt	En demande d'informations complémentaires
Football	45,7	8,4
Ski alpin (hors freestyle)	34,1	4,1
Tennis	28,2	4,1
Hockey sur glace	20,2	3,7
Athlétisme	9,9	1,4
Ski nordique (y compris biathlon)	7,1	0,9
Cyclisme (hors VTT)	6,9	1,3
Sports automobiles, formule 1	6,7	0,8
Patinage	3,6	1,0
Courses de moto	3,6	1,3
Natation	3,2	0,8
Équitation	2,7	1,8
Basketball	2,7	1,1
Jeux Olympiques	2,6	0,2
Gymnastique rythmique et sportive, gymnastique artistique	1,9	1,0
Handball	1,9	1,0
Lutte suisse	1,7	1,5
Volleyball, beach-volley	1,5	0,7
Boxe, kickboxing	1,3	0,5
VTT	1,1	0,9
Rugby, football américain	1,1	0,6
Unihockey	1,0	0,8
Curling	1,0	0,3
Snowboard (hors freestyle)	0,9	0,5
Freestyle (ski, snowboard, BMX)	0,9	0,9
Golf	0,8	0,4
Bob	0,8	0,3

Remarque: sports ou manifestations sportives d'envergure cité(e)s par au moins 0,8 % de la population. Nombre de personnes interrogées: 1056 (dans le cadre d'un module spécial). Réponses aux questions suivantes: «A quels sports vous intéressez-vous le plus?» et «Sur quels sports souhaiteriez-vous lire plus d'articles ou voir plus de reportages?»

16. Dépenses liées au sport

Un budget de 2500 francs par habitant et par an

Le sport est non seulement un loisir extrêmement populaire et un divertissement très prisé, mais également un facteur économique majeur. Selon les études menées par Rütter + Partner, le sport suisse pèse près de 18 milliards de francs et génère une valeur ajoutée brute d'environ 9 milliards. Le sport représente ainsi 1,7 % du produit intérieur brut suisse.

Dans le cadre du module d'enquête en ligne, les participants ont été priés de fournir des informations détaillées sur leurs dépenses liées au sport au cours des 12 derniers mois. Il en ressort que la population suisse âgée de 15 à 74 ans consacre en moyenne 2500 francs par an et par personne au sport (cf. tableau 16.1). Sur cette somme, 2000 francs sont dépensés en Suisse contre à peine 500 à l'étranger. Quelque 93 % de la population ont un budget dédié au sport. Ce n'est pas une surprise compte tenu du niveau d'activité et de la consommation sportive des non-sportifs (cf. chapitres 9 et 15). Les sportifs très actifs y consacrent ainsi 3531 francs par personne et par an, contre 1143 francs pour les non-sportifs. Cette dernière somme est affectée non seulement aux médias du sport ou à l'acquisition de billets pour des manifestations sportives, mais également à l'achat des vêtements et des chaussures nécessaires à la pratique d'activités physiques (cf. chapitre 9) ainsi qu'à l'accès aux infrastructures sportives.

Les hommes consacrent environ 700 francs de plus au sport par personne et par an que les femmes. Plus le niveau de revenu est élevé, plus les dépenses liées au sport sont importantes. Dans le quartile de revenu par équivalent adulte inférieur, les dépenses annuelles représentent en moyenne 1500 francs, contre 3700 francs dans le quartile supérieur.

Les vacances et les voyages sont les principaux postes de dépenses liés au sport

Les vacances sportives à destination de la Suisse et de l'étranger pèsent particulièrement lourd sur le budget consacré au sport. Elles représentent 319 francs par an pour la première catégorie, 368 francs pour la seconde. En tenant compte du fait que respectivement 26 % et 19 % de la population font état de ces dépenses, les sommes mobilisées par les vacances sportives à destination de la Suisse et de l'étranger s'élèvent en moyenne à 1227 et 1937 francs par an et par personne. Les postes de dépenses plus élevés concernent l'acquisition d'équipements et de vêtements de sport. Un tiers de la population déclare ainsi avoir acquis un équipement de sport au cours des 12 derniers mois. Les deux tiers ont par ailleurs acheté des vêtements ou des chaussures de sport.

L'analyse du contexte de la pratique sportive révèle que les Suisses dépensent 94 francs par an et par personne pour les cotisations de clubs sportifs, contre 159 francs pour les frais d'adhésion et les entrées des centres de fitness. Enfin, ils consacrent 149 francs à l'utilisation des infrastructures sportives. Compte tenu de la diversité du nombre d'utilisateurs, on peut affirmer que la pratique d'un sport en club revient à environ 300 francs par an, contre 700 francs pour un centre de fitness et 250 francs pour les autres installations sportives.

La consommation passive de sport est beaucoup moins onéreuse. En moyenne, les sondés consacrent 80 francs par an et par personne à l'achat de billets pour des manifestations sportives. Les médias du sport représentent quant à eux une dépense de 19 francs, contre 8 francs pour l'acquisition de produits dérivés. Enfin, comme pour l'activité sportive, des frais de transport s'ajoutent souvent à l'achat des billets d'entrée pour les manifestations sportives (115 francs par personne et par an en moyenne).

T 16.1: Dépenses consacrées au sport par la population résidante suisse âgée de 15 à 74 ans

		Dépenses moyennes par habitant (en CHF)	Part de la population faisant état de telles dépenses (en %)
Dépenses en Suisse	Vêtements de sport	215	69
	Chaussures de sport	160	64
	Équipement	246	33
	Locations et prêts d'équipements	38	17
	Équipement divers	46	26
	Entretien/réparation des équipements	57	32
	Fréquentation de manifestations sportives	80	34
	Excursions à caractère sportif	89	33
	Voyages/vacances à caractère sportif	319	26
	Frais de transport liés au sport	115	33
	Cotisations versées à des clubs sportifs	94	31
	Dons à des clubs, associations, fondations et manifestations	33	18
	Entrées et utilisation d'installations sportives (dont remontées mécaniques, etc.)	149	58
	Entrées et abonnements de centres de fitness	159	22
	Séances d'entraînement particulières, cours et formations	96	16
	Alimentation spécifique	24	14
	Services et produits médicaux axés sur le sport	62	16
	Presse et médias (magazines spécialisés, télévision à la demande, etc.)	19	13
	Produits dérivés/objets de collection	8	6
	Autres dépenses liées au sport (p. ex. assurances)	19	6
	**Dépenses totales en Suisse**	**2028**	**92**
Dépenses à l'étranger	Vêtements, chaussures, équipements, locations et prêts, équipements divers	67	18
	Fréquentation de manifestations sportives et excursions à caractère sportif	47	9
	Voyages/vacances à caractère sportif à l'étranger	368	19
	**Dépenses totales à l'étranger**	**482**	**32**
**Dépenses totales liées au sport (Suisse et étranger)**		**2510**	**93**

Remarque: nombre de personnes interrogées: 6195 (enquête en ligne). La première colonne indique le budget moyen consacré au sport par la population âgée de 15 à 74 ans. La deuxième colonne indique la part de la population (15-74 ans) qui fait état des dépenses concernées.

17. Encouragement du sport et défis à relever

Une profession de foi en faveur de l'encouragement du sport

«Admettons que vous disposiez de 1000 francs à dépenser pour des activités culturelles, sociales ou sportives: quelle somme consacreriez-vous à chaque domaine?» Cette question vise à établir comment la population suisse répartirait ses dépenses en cas de gain à la loterie nationale. Certaines personnes ont réparti les 100 francs équitablement sur les trois domaines. La majorité des personnes interrogées a toutefois procédé à une pondération réfléchie. En moyenne, 380 francs ont été attribués au domaine social, 345 francs au sport et 274 francs à la culture.

Cette «clé de répartition» repose sur une large base. Qui plus est, elle est en grande partie indépendante de la région d'appartenance ou du statut social d'une personne. On ne relève aucune différence notable entre les régions linguistiques. En d'autres termes, les Suisses alémaniques, romands et italophones consacreraient le même budget au sport. Les personnes disposant d'un niveau de formation supérieur dépenseraient un peu moins que celles bénéficiant d'un revenu élevé. Enfin, les résidents étrangers dépenseraient moins que la population de souche. Les hommes consacreraient une somme légèrement supérieure au sport que les femmes. Il en va de même pour les plus jeunes face aux seniors.

Globalement, les différences sont toutefois peu marquées. Les valeurs moyennes enregistrées pour les différentes catégories de la population oscillent toutes entre 300 et 380 francs. Seuls les non-sportifs affichent une valeur sensiblement inférieure, avec 288 francs. Cela s'explique surtout par le fait qu'ils comptent dans leurs rangs 15 % de personnes qui ne consacreraient aucune dépense au sport, soit deux fois plus que dans les autres catégories. Il faut préciser que les non-sportifs englobent également 17 % de personnes prêtes à dépenser plus de 400 francs pour le sport.

G 17.1: Appréciation relative à l'encouragement du sport (en % de la population résidante suisse âgée de 15 à 74 ans)

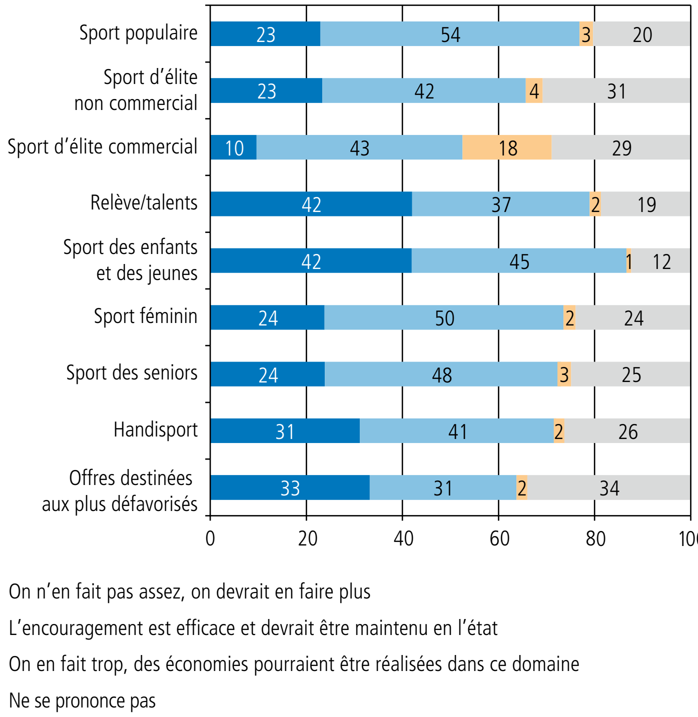

Remarque: nombre de personnes interrogées: 6742 (enquête en ligne)

Un appel au renforcement du sport des jeunes et de la promotion de la relève

Non seulement la population suisse pratique et consomme des activités sportives avec enthousiasme, mais elle est également prête à les soutenir par la mise à disposition des moyens financiers et des infrastructures nécessaires (cf. graphique 17.1). Les Suisses estiment en effet à une très large majorité que le sport doit continuer à être encouragé dans les mêmes proportions, voire davantage. Ils soutiennent notamment le sport des jeunes, qu'il s'agisse de l'encouragement du sport populaire ou d'une promotion ciblée de la relève.

L'importance du sport des jeunes est soulignée par le fait que 99 % de la population considèrent que le sport exerce une influence positive sur les jeunes. Selon les personnes interrogées, la détermination et la volonté, la capacité à surmonter les échecs et les défaites, le fair-play et la maîtrise de l'agressivité sont autant de qualités que le sport permet de développer plus particulièrement chez les jeunes. La pratique d'un sport contribuerait également de façon importante à la lutte contre le tabagisme, l'alcoolisme et la toxicomanie.

L'image positive du sport est confirmée par le graphique 17.2. Une nette majorité de sondés estime que le sport est un bon moyen de lutter contre le manque d'exercice physique et contribue de façon déterminante à la santé de la population. Outre l'argument de la santé, la contribution du sport à l'intégration, à l'atténuation des comportements agressifs et à une bonne entente entre les peuples est largement saluée. Seule une minorité de personnes interrogées (environ 15 %) estime que le sport attise (plutôt) les comportements agressifs et les rivalités ou favorise les préjugés nationalistes.

G 17.2: Validation de différentes affirmations contradictoires (en % de la population résidante suisse âgée de 15 à 74 ans)

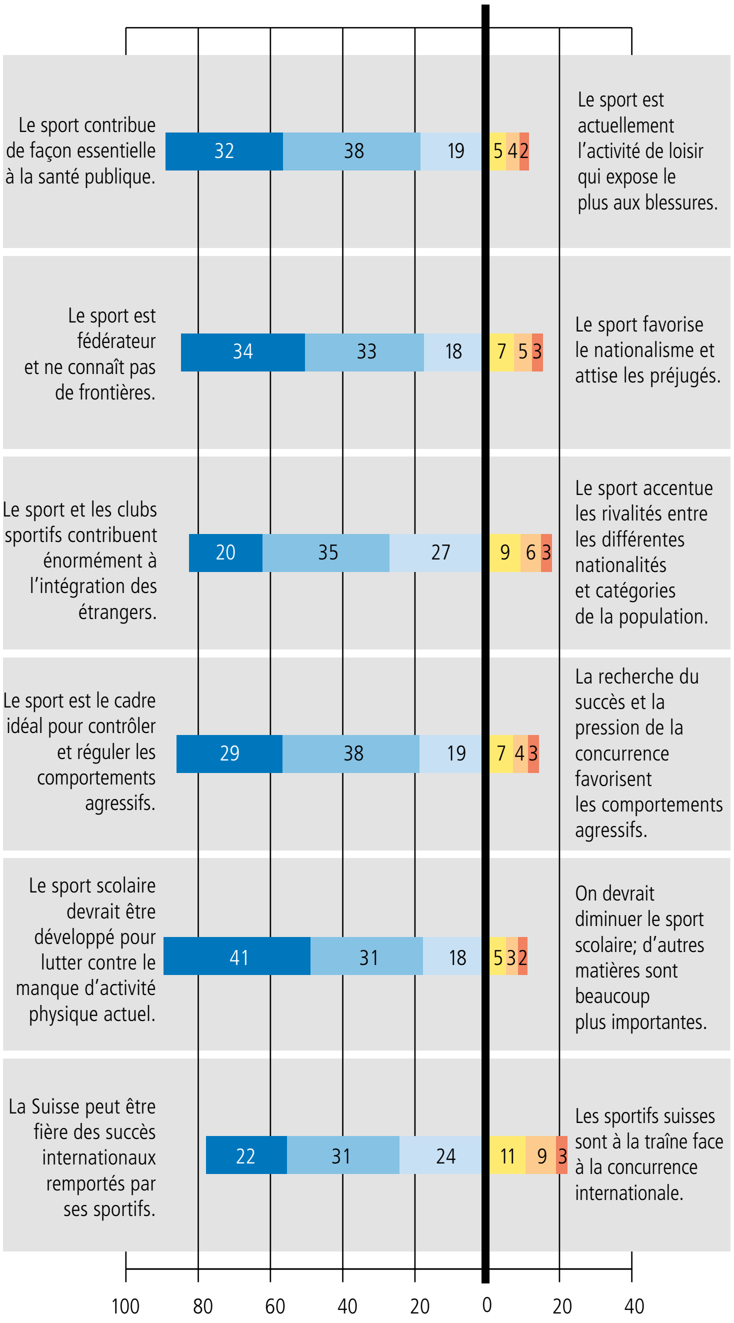

Remarque: nombre de personnes interrogées: 6806 (enquête en ligne). Le graphique indique le degré de validation des différentes affirmations sur une échelle à six niveaux. Plus on se rapproche d'un côté, plus le degré de validation de l'affirmation correspondante est important.

Le sport d'élite commercial et les grands événements sportifs sujets à caution

Dans ce contexte, il n'est pas surprenant que les personnes interrogées plébiscitent non seulement le sport des jeunes, mais également la promotion du handisport, du sport des seniors et du sport féminin ainsi que des offres à destination des catégories de population défavorisées et, plus généralement, du sport populaire (cf. graphique 17.1). Pour ce qui est du sport d'élite, les avis sont nuancés: si le niveau d'adhésion au sport d'élite commercial est similaire à celui remporté par le sport populaire, un nombre croissant de personnes estiment que des économies pourraient être faites dans ce domaine. Globalement, une majorité de voix se prononce néanmoins en faveur du maintien du soutien actuel, 10 % plaidant même en faveur d'un renforcement de l'encouragement du sport d'élite. Les quatre cinquièmes de la population estiment par ailleurs que la Suisse peut être fière des succès remportés par ses sportifs dans les compétitions internationales. Seul un cinquième des sondés pense que les sportifs suisses sont à la traîne par rapport à leurs concurrents internationaux.

Le graphique 17.3 montre que des opinions critiques ont été exprimées à l'égard du sport d'élite commercial et des manifestations sportives d'envergure comme les Jeux Olympiques ou les championnats du monde. Une majorité de la population continue néanmoins à accorder de l'importance aux succès obtenus au niveau international par les sportifs suisses. En outre, les trois quarts de la population environ reconnaissent la valeur des victoires remportées pour la Suisse comme en termes d'image du pays à l'étranger. Les personnes interrogées remettent également sérieusement en cause l'importance des grandes compétitions internationales. Tandis qu'une majorité d'entre elles estime qu'il est important pour la Suisse de participer à des événements comme les championnats d'Europe et du monde, pour ce qui est des Jeux Olympiques d'hiver, les avis sont plus partagés: la moitié des personnes interrogées pense qu'il est (plutôt) important pour la Suisse d'y participer, l'autre estime que c'est (plutôt) inutile. En comparant ces chiffres avec les résultats obtenus en 2000, on constate que l'importance accordée aux succès remportés par les sportifs suisses dans les compétitions internationales ainsi qu'aux manifestations d'envergure a plutôt diminué.

G 17.3: Appréciation de l'importance des grandes manifestations sportives (en % de la population résidante suisse âgée de 15 à 74 ans)

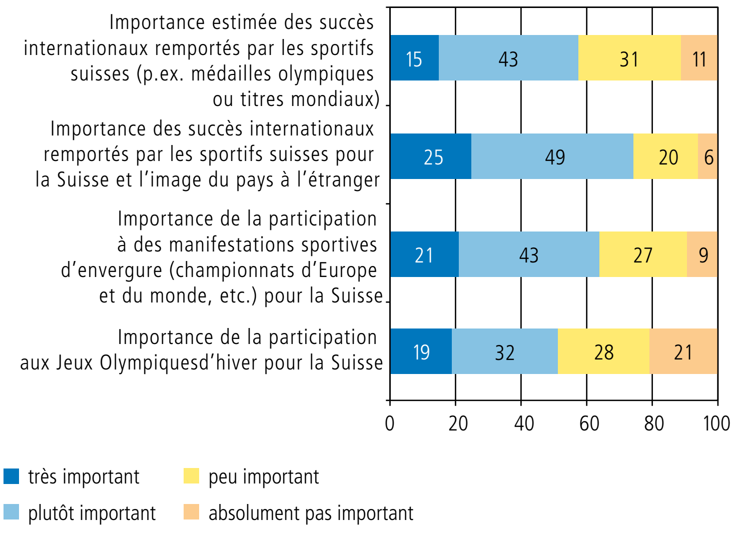

Remarque: nombre de personnes interrogées: de 6593 à 6640 (enquête en ligne)

Le problème majeur associé au sport: le dopage

Le fait que les Suisses émettent plus de réserves sur le sport d'élite commercial et sur les grands événements sportifs que sur le sport des jeunes et le sport populaire s'explique par les problèmes qui touchent ce milieu. Dans le cadre des entretiens téléphoniques, les personnes interrogées ont été priées d'indiquer quels sont les principaux problèmes du sport à l'heure actuelle selon elles. En regroupant les réponses spontanées obtenues par catégories, il apparaît que le dopage occupe la première place, suivi de la commercialisation croissante, de la «course à la performance», du hooliganisme, d'une prise de risque trop grande et de la corruption. Les problèmes identifiés concernent tous le sport d'élite commercial, très peu de sources de préoccupation étant associées au sport des jeunes et au sport populaire. La violence entre les sportifs, l'impact négatif du sport sur l'environnement, le racisme ou les abus sexuels ainsi qu'une mauvaise gestion des clubs ne sont que rarement pointés du doigt.

G 17.4: Problèmes associés au sport (part de la population ayant cité un problème donné, en %)

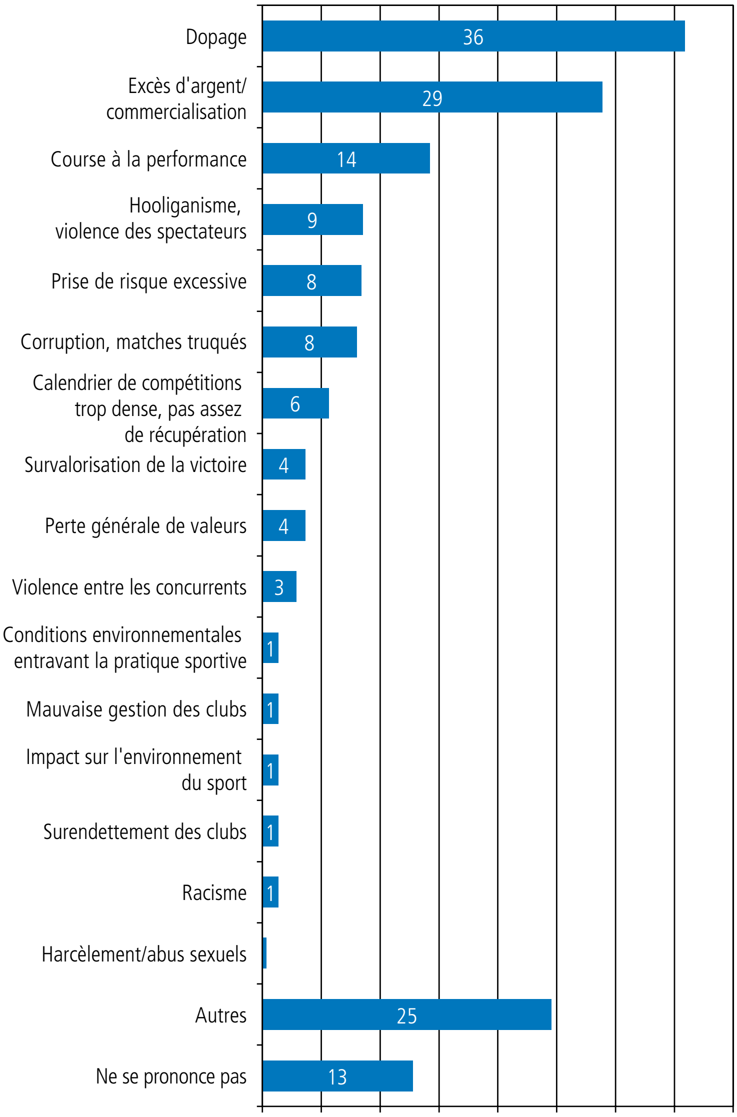

Remarque: nombre de personnes interrogées: 10 444

L'appréciation concrète des problèmes évoqués et des solutions proposées dans le cadre de l'enquête en ligne donne un tableau plus nuancé, mais au final similaire (cf. graphique 17.5). L'impact négatif sur l'environnement et la circulation occasionnée par le sport recueillent ainsi un peu plus d'attention. Cependant, une majorité de la population ne considère pas le sport comme un facteur aggravant des nuisances pour l'environnement. En ce qui concerne le dopage, les sondés distinguent la situation du sport international de celle du sport suisse. Dans le premier cas, une nette majorité de personnes est convaincue que le dopage reste répandu, contre une minorité dans le second. Peu de personnes se risquent toutefois à un avis sur l'étendue du dopage en Suisse. En revanche, la population approuve unanimement la nécessité d'une lutte rigoureuse contre le dopage, même si les résultats des sportifs suisses doivent s'en ressentir et si leurs carrières doivent en souffrir. (Les résultats détaillés obtenus font l'objet d'un rapport spécial sur le dopage mandaté par Antidoping Suisse.)

La population prône l'intransigeance face à la violence, le hooliganisme et la corruption

Parallèlement à un engagement clair en faveur d'une lutte rigoureuse contre le dopage, l'étude révèle que la reconnaissance du sport professionnel et (de la probité) des performances de pointe laisse à désirer en Suisse. Une part conséquente de la population estime que les performances sportives de pointe sont trop peu reconnues en Suisse et qu'il est difficile de faire une carrière dans le sport. Le graphique 17.1 indiquait déjà le potentiel d'amélioration qui existe dans le domaine du sport de la relève, de la promotion des talents et du sport d'élite (non commercial).

S'agissant de la lutte contre la violence, le hooliganisme et la corruption, une écrasante majorité de la population plaide en faveur d'une ligne politique dure: interdiction stricte des feux d'artifice dans les stades; lutte conséquente contre la violence lors des manifestations sportives assortie de punitions sévères; interdiction d'accès aux stades pour les fauteurs de trouble et les casseurs; mesures fortes contre la corruption dans le sport et interdiction des sociétés privées de paris sportifs.

G 17.5: Appréciation relative aux problèmes identifiés et aux mesures correctives préconisées (en % de la population résidante suisse âgée de 15 à 74 ans)

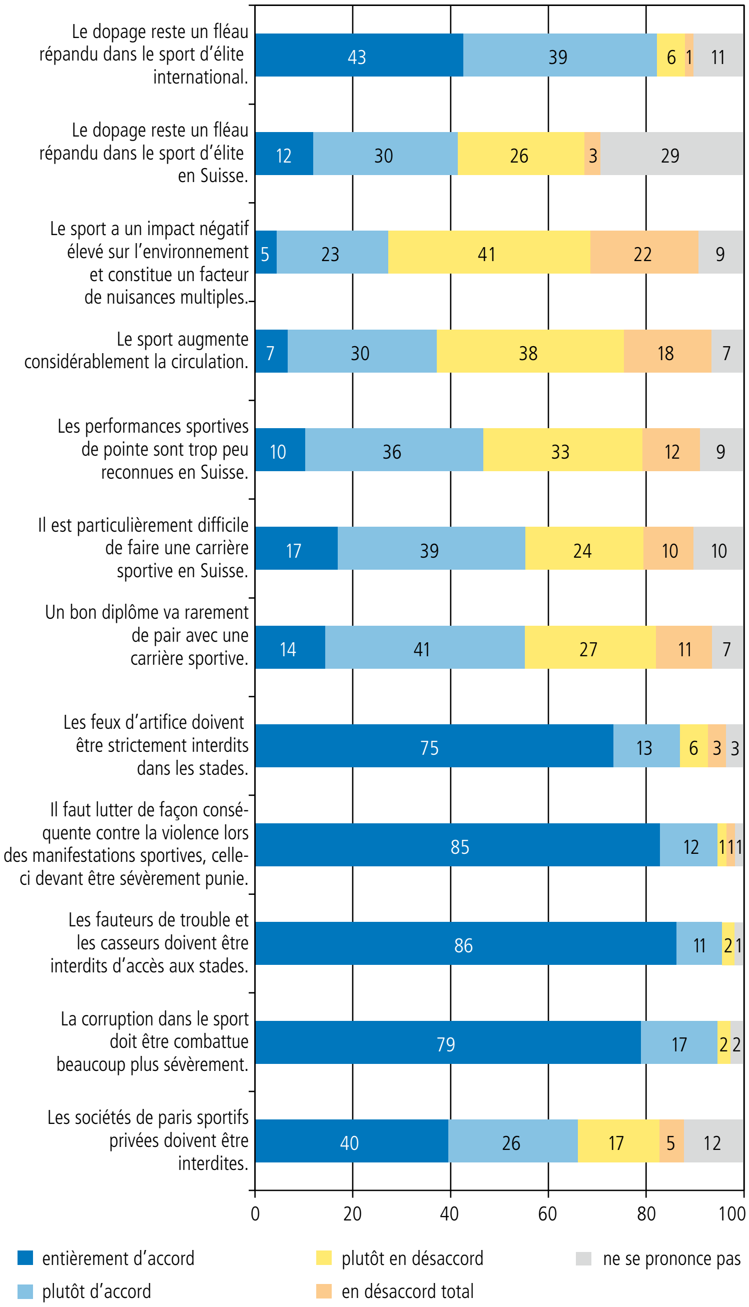

Remarque: nombre de personnes interrogées: 6770 (enquête en ligne)

Globalement, la population suisse met en avant les aspects positifs du sport et se prononce clairement en faveur d'une large promotion de ce secteur tout en préconisant une lutte déterminée contre ses zones d'ombre (dopage, violence ou corruption).

18. Méthode de recherche et sondage

L'enquête a été réalisée sous la forme d'enquêtes téléphoniques assistées par ordinateur (CATI), suivies d'une enquête en ligne. Elle a été menée par l'institut LINK à Lucerne, Zurich, Lausanne et Lugano. Le questionnaire a été élaboré par l'Observatoire Sport et activité physique Suisse sur la base de l'expérience accumulée lors des éditions 2000 et 2008, en collaboration étroite avec ses soutiens et ses partenaires. Il est disponible sur demande (www.sportobs.ch).

Deux tests préparatoires ont été effectués le 11 décembre 2012 et le 21 janvier 2013 pour évaluer le questionnaire et le déroulement de l'entretien. Tous les enquêteurs et leurs superviseurs ont suivi une formation approfondie en amont. Les entretiens ont été menés dans les trois langues nationales (allemand, français et italien). L'enquête de terrain a démarré le 15 février 2013: les entretiens téléphoniques se sont déroulés jusqu'au 22 juillet, l'enquête en ligne ayant été clôturée le 26 août. Afin d'exclure tout effet saisonnier, l'enquête s'est déroulée en plusieurs phases: 1re phase à partir du 15 février, 2e phase à partir du 2 avril, 3e phase à partir du 24 mai.

L'enquête Sport Suisse porte sur l'ensemble de la population résidante suisse âgée de 15 à 74 ans. Un échantillon représentatif de cette population a été sélectionné (module de base). Des échantillons complémentaires (adolescents ainsi que cantons et villes participants) ont été constitués (cf. tableau 18.1). Au total, 10 652 entretiens téléphoniques ont été réalisés. S'ajoute l'enquête en ligne, à laquelle 7104 personnes ont participé. Le taux de participation à l'enquête en ligne est compris entre 60 et 70 % selon les échantillons. En plus des personnes âgées de 15 à 74 ans, des enfants de 10 à 14 ans ont été interrogés par téléphone dans le cadre de l'enquête Sport Suisse, à l'aide d'un questionnaire distinct. Les résultats obtenus feront l'objet d'un rapport séparé consacré aux enfants et aux jeunes.

Les personnes interrogées ont été sélectionnées suivant le principe aléatoire du cadre de sondage pour les enquêtes auprès de la population mis à disposition par l'OFS. Un avis préalable à été envoyé par courrier à chaque personne par l'Office fédéral du sport pour l'informer de la réalisation de l'enquête, de l'intérêt de l'étude et de la politique de confidentialité appliquée dans ce cadre. Une hotline a par ailleurs été mise en place, et un site d'information dédié a été créé sur Internet. Le tableau 18.2 donne un aperçu des taux de participation pour les différents modules. Il indique également les entretiens qui n'ont pas pu aboutir. Grâce aux mesures d'accompagnement (courriers de rappel supplémentaires, rappels téléphoniques en cas de refus, etc.) et à la motivation des enquêteurs, un taux de retour de 65 % a été atteint. Les taux de participation sont particulièrement élevés parmi les enfants (78 %) et les adolescents (81 %). Les refus nets sont restés marginaux et ne représentent que 6 % en moyenne.

T 18.1: Vue d'ensemble du nombre d'entretiens réalisés dans les différents modules

	Téléphone	En ligne	En ligne en %
**Module de base Suisse (15-74 ans)**	**3557**	**2374**	**66,7**
**Module supplémentaire Adolescents (15-19 ans)**	**1011**	**1331**	**70,2**
Canton d'Argovie	761	696	66,7
Canton de Bâle-Campagne	728	583	69,3
Canton de Genève	686	605	60,1
Canton des Grisons	704	534	65,9
Canton de Saint-Gall (hors ville de Saint-Gall)	621	571	69,3
Canton de Zurich (hors Zurich/Winthertour)	721	780	67,6
Ville de Saint-Gall	399	288	66,8
Ville de Winterthour	747	530	66,9
Ville de Zurich	717	575	67,5
**TOTAL (15-74 ans)**	**10 652**	**7104**	**66,7**
Module supplémentaire Enfants (10-14 ans)	1525		

T 18.2: Vue d'ensemble du taux de participation à l'enquête par téléphone et des défaillances

	Module de base Suisse	Module supplé-mentaire Adoles-cents	Module supplé-mentaire Cantons	Total	Module supplé-mentaire Enfants
**Adresses conformes**	**5346**	**1254**	**9874**	**16 474**	**1952**
Bon contact	876	114	1582	2572	166
Refus	369	44	619	1032	134
Problèmes liés à la langue	246	7	610	863	57
Problèmes liés à l'âge/la santé	187	11	427	625	3
Absent/e pendant la durée de l'enquête	68	59	436	563	25
Autres défaillances	43	8	116	167	42
**Entretiens réalisés**	**3557**	**1011**	**6084**	**10 652**	**1525**
**Taux d'utilisation**	**67**	**81**	**62**	**65**	**78**
Durée de l'entretien: moy. arith. (en minutes)	24,1	25,9	26,1	25,4	32,7

La durée moyenne d'un entretien était de 25 minutes. Une grande majorité d'entretiens s'est déroulée en langue allemande (72 %) contre un cinquième en français et 8 % en italien. Le taux de participation des femmes et des hommes est équivalent et reflète bien la pyramide des âges de la Suisse, en tenant compte du fait qu'un échantillon supplémentaire d'adolescents a été constitué. Quelque 16 % des personnes interrogées sont de nationalité étrangère, soit une part inférieure à la proportion d'étrangers au sein de la population totale (23,3 % fin 2012). Cela s'explique principalement par le fait que l'enquête n'a été réalisée que dans les trois langues nationales. Dans le présent rapport, il est donc systématiquement fait référence à la population résidante assimilée linguistiquement âgée de 15 à 74 ans.

Afin que l'échantillonnage global composé des différents échantillons partiels soit représentatif de la population résidante suisse, les données ont été pondérées. La pondération tient compte de la taille variable des échantillons selon les régions et les classes d'âge, ainsi que d'un taux de participation à l'enquête en ligne légèrement différent selon l'activité sportive. Les présents chiffres se fondent sur des données pondérées. Le nombre de cas n'est pas pondéré. Il correspond au nombre effectif de personnes interrogées.

«Sport Suisse» est la plus vaste enquête sur le comportement sportif qui ait été menée en Suisse. Les données ont été recueillies et analysées sur la base de critères scientifiques très stricts. Malgré cela, il faut garder à l'esprit que les enquêtes par échantillonnage présentent toujours certaines marges d'erreur. La principale valeur de contrôle statistique de cette marge d'erreur est l'intervalle de confiance. Cet intervalle est calculé à l'aide de la formule suivante:

$IC = +/-2 \sqrt{(p(100-p)/n)}$

IC = intervalle de confiance
p = part de personnes interrogées ayant fourni une réponse donnée (en points de pourcentage)
n = taille de l'échantillon non pondérée

A titre d'exemple, si 44,3 % de personnes faisant partie de l'échantillon sélectionné déclarent faire de la randonnée, la valeur «réelle» extrapolée à l'échantillon global selon une probabilité de 95 % se situe entre 43,3 et 45,3 % (intervalle de confiance: +/–0,96 point de pourcentage). En ce qui concerne la voile, sport cité par 1,1 % de la population, la valeur «réelle» selon une probabilité de 95 % se situe entre 0,9 et 1,3 % (intervalle de confiance: +/–0,20 point de pourcentage). Par conséquent, il convient d'interpréter avec prudence les petites variations observées entre 2008 et 2014 (cf. tableau 6.1), dans la mesure où elles ne sont pas pertinentes d'un point de vue statistique. Ceci est valable en particulier pour les évolutions concernant la Suisse italophone (cf. tableau 7.1).

Les résultats présentés ont été confirmés à l'aide de diverses analyses de variance (multivariées) et contrôlées en vue d'en établir la pertinence statistique. Pour des raisons liées à des contraintes de place, les analyses en question ne figurent pas dans ce rapport. Seuls les résultats dont la pertinence statistique atteint 99 % ont été interprétés.

Selon les données de l'OFS, la population résidante suisse âgée de 15 à 74 ans représentait environ 6,2 millions de personnes en 2013. Compte tenu de l'intervalle de confiance, on peut donc affirmer qu'un pour-cent de sondés équivaut à 62 000 personnes.

Image: © www.slowUp.ch

Observatoire Sport et activité physique Suisse
c/o Lamprecht & Stamm Sozialforschung und Beratung AG
Forchstrasse 212
CH-8032 Zurich
info@sportobs.ch
www.sportobs.ch

Office fédéral du sport OFSPO
2532 Macolin
info@baspo.admin.ch
www.ofspo.ch

1404.018
