# Supplementary material for: Reviewing the role of the environment in the talent development of a professional soccer club
Source: PLoS One. 2021 Feb 25;16(2):e0246823. doi: 10.1371/journal.pone.0246823 (PMC7906372; doi:10.1371/journal.pone.0246823)
Supplement: S3 File — (PDF) [file pone.0246823.s003.pdf]

Haute école fédérale de sport de Macolin HEFSM

Le sport d'élite en Suisse

Etat des lieux SPLISS-CH 2011

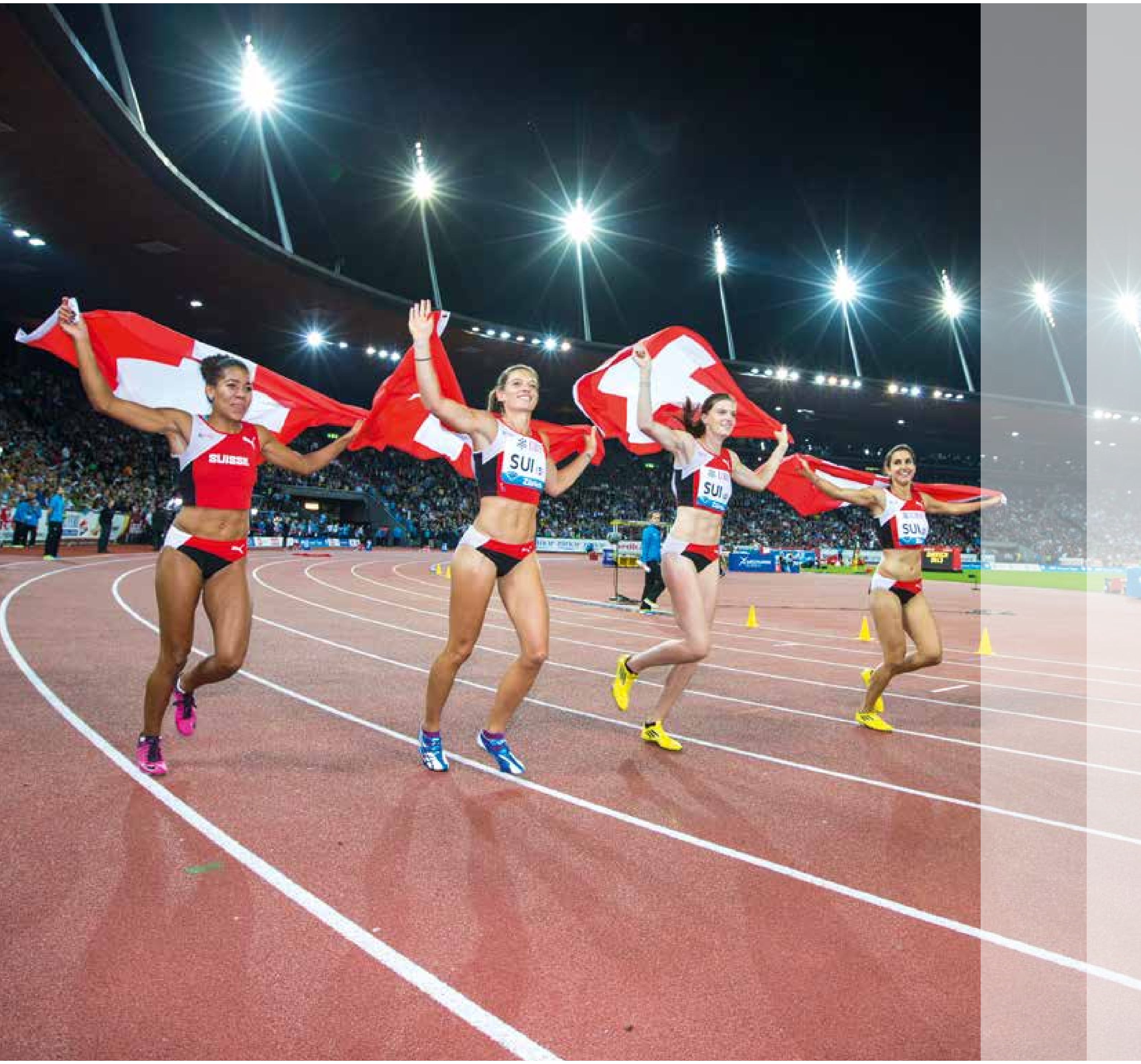

Schweizerische Eidgenossenschaft
Confédération suisse
Confederazione Svizzera
Confederaziun svizra

Office fédéral du sport OFSPO

OFSPO
2532 Macolin

Le sport d’élite en Suisse

Etat des lieux SPLISS-CH 2011
(SPLISS = Sports Policy factors Leading to International Sporting Success)

Macolin, mai 2014

Hippolyt Kempf
Andreas Christoph Weber
Anne Renaud
Marco Stopper

Charte Olympique: règle 57, tableau d'honneur

«Le CIO et le COJO n'établiront aucun classement global par pays. Un tableau d'honneur portant les noms des médaillés et des diplômés de chaque épreuve sera établi par le COJO et les noms des médaillés seront mis en évidence de façon permanente dans le stade principal.»

Source: Comité International Olympique (CIO) (2013a)
(COJO = Comité d'Organisation des Jeux Olympiques)

Table des matières

Liste des abréviations 6

L'essentiel en bref 9

1. Introduction 11

2. Le marché des médailles olympiques 12

3. Le sport suisse: présentation d'ensemble 16

4. Le financement du succès olympique 21

5. Détection et promotion des talents 28

6. Les athlètes et le sport d'élite 32

7. Entraîneurs, managers et dirigeants du sport 37

8. Recherche et développement 44

9. Installations sportives et centres d'entraînement 48

10. Compétitions internationales 53

11. Perspectives pour la nation sportive suisse 57

12. Concept d'étude et méthode 62

Annexe 64

Bibliographie 68

Remerciements 72

Pour des raisons de lisibilité, seule la forme masculine est employée dans ce rapport.

Liste des abréviations

4S	Société suisse des sciences du sport
ADEC	Association pour le développement de l'élevage et des courses
AISTS	Académie Internationale des Sciences et Techniques du Sport
ASF	Association suisse de football
ASS	Association suisse du sport
BMX	Bicycle Moto Cross
CDE	Cours d'entraîneur
CFP	Commission fédérale du sport
CIES	Centre international d'étude du sport
CIO	Comité International Olympique
CISIC	Conception des installations sportives d'importance cantonale
CISIN	Conception des installations sportives d'importance nationale
CNO	Comité National Olympique
CNSE	Comité national pour le sport d'élite
COJO	Comité d'Organisation des Jeux Olympiques
COS	Comité Olympique Suisse
CST	Centro sportivo nazionale della gioventù Tenero
CTI	Commission pour la technologie et l'innovation
DDPS	Département fédéral de la défense, de la protection de la population et des sports
EPFL	Ecole polytechnique fédérale de Lausanne
EPFZ	Ecole polytechnique fédérale de Zurich
EURO 08	Championnat d'Europe de football 2008
FEP	Formation d'entraîneur professionnel
FIFA	Fédération Internationale de Football Association
FIS	Fédération Internationale de Ski
FNS	Fonds national suisse
HEFSM	Haute école fédérale de sport de Macolin
IDHEAP	Institut de hautes études en administration publique
J+S	Jeunesse+Sport
JO	Jeux Olympiques

NL	National League, depuis 2011 Swiss Ice Hockey Federation
OAM	Ordonnance concernant l'appui d'activités civiles et d'activités hors du service impliquant des moyens militaires
OFFT	Office fédéral de la formation professionnelle et de la technologie, rattaché en 2013 au Secrétariat d'Etat à la formation, à la recherche et à l'innovation
OFS	Office fédéral de la statistique
OFSPO	Office fédéral du sport
Olymp.	Olympique
PISTE	Pronostic intégratif et systématique par l'estimation de l'entraîneur
R&D	Recherche et développement
SEFRI	Secrétariat d'Etat à la formation, à la recherche et à l'innovation
SFL	Swiss Football League
SIHA	Swiss Ice Hockey Association, depuis 2011 Swiss Ice Hockey Federation
SLF	Institut pour l'étude de la neige et des avalanches
SOA	Swiss Olympic Association (forme abrégée: Swiss Olympic)
SPLISS	Sports Policy factors Leading to International Sporting Success
SSMC	Swiss Sport Management Center
STG	Société du Sport-Toto
STS	SwissTopSport
Trad.	Traditionnel
UEFA	Union of European Football Associations
VMI	Verbandsmanagementinstitut
VTT	Vélo tout terrain

L’essentiel en bref

Dans le monde du sport, les Jeux Olympiques se trouvent au sommet de la pyramide. La multiplication des compétitions et l’intensification de la concurrence en font un enjeu d’une importance croissante pour les nations qui y participent. Une victoire olympique couronne en effet une carrière sportive et peut influencer de manière décisive le développement d’une discipline dans un pays. Le sport suisse encourage les athlètes et les fédérations en se concentrant principalement sur l’objectif de médailles olympiques fixé. De fait, à moins de disposer d’un soutien solide, il est impossible d’assurer un succès durable aux Jeux.

Le rapport «Le sport d’élite en Suisse – Etat des lieux SPLISS-CH 2011» présente de manière documentée le système du sport d’élite suisse et l’analyse de façon critique à la lumière des objectifs de médaille. Pour l’établir, les auteurs ont interrogé 959 athlètes, 682 entraîneurs et 58 chefs du sport d’élite, et ont confronté les réponses obtenues à d’autres sources bibliographiques, à des analyses de documents et à des avis d’experts recueillis au cours de 68 entretiens. Enfin, le rapport reprend des argumentations présentées lors de divers ateliers ainsi que dans des travaux de recherche internationale.

Ce travail permet un constat global: le soutien que la Suisse apporte au sport d’élite olympique est efficace sur bien des points. Les améliorations à apporter se concentrent principalement sur quatre domaines:

Améliorer le financement: l’argent joue un rôle clé dans le sport d’élite moderne. On trouvera dans ce rapport quelques indications sur le volume et la répartition des moyens financiers disponibles. Les athlètes, les entraîneurs et les responsables du sport d’élite s’accordent à dire que ces moyens sont insuffisants pour assurer un succès durable. La concentration des moyens sur un petit nombre de disciplines ne suffit pas.

Consolider sa carrière grâce au sport d’élite: la route qui mène au podium est extrêmement longue. Voilà pourquoi il est important d’orienter le plus tôt possible les jeunes talents vers le sport d’élite et de sensibiliser leur entourage proche à cet univers pour assurer une progression constante. L’autre étape décisive intervient un peu plus tard, lorsqu’il leur est demandé de se concentrer sur le sport. Parmi les athlètes interrogés, seuls 30 % sont des sportifs à plein temps. Il convient d’augmenter ce chiffre si l’on souhaite assurer le succès de la Suisse aux Jeux Olympiques. Par ailleurs, il est capital, pour tous les domaines de l’existence de l’individu, d’obtenir la reconnaissance de ses aptitudes professionnelles en tant qu’athlète de métier. Les lacunes dans ce domaine sont actuellement bien réelles.

Elargir les débouchés professionnels dans le sport d’élite: c’est l’association de diverses aptitudes et techniques qui mène les athlètes au plus haut niveau et leur permet de réussir aux Jeux Olympiques. A la base de ce succès, on trouve non seulement un bon entraîneur épaulé par une équipe de spécialistes, mais aussi un matériel performant, ainsi qu’un management réfléchi. La division du travail et la spécialisation se révèlent des facteurs de réussite durable sur le dur chemin qui mène à la victoire. Pourtant, en Suisse, trop peu de mesures sont mises en œuvre pour favoriser un succès systématique. Ainsi, les avantages offerts par certains sites, notamment en matière de recherche et de développement, sont insuffisamment exploités. Le secteur du sport d’élite n’est pas assez attractif pour de nombreux spécialistes très qualifiés.

Créer des plus-values grâce aux synergies: le rassemblement de plusieurs disciplines peut être source de plus-values. Les centres de compétences dédiés à l’entraînement peuvent constituer des lieux de partage du savoir. Le succès et la création de véritables centres d’entraînement passent par des infrastructures modernes et de bonne qualité. La Suisse doit s’efforcer de s’améliorer sur ce point.

1. Introduction

Le sport intéresse, les victoires passionnent. En Suisse, les principales compétitions disputées par des sportifs helvétiques sont généralement suivies par une large frange de la population. Depuis quelques années, le sport d'élite s'est internationalisé et représente maintenant un marché mondial, une évolution qui a durablement transformé son importance et son fonctionnement au niveau national. Il fait également l'objet d'une professionnalisation continue à tous les niveaux. Ces mutations influencent la politique nationale du sport.

Les acteurs du monde sportif – au premier rang desquels les athlètes, les entraîneurs, les managers et les dirigeants de fédération – font face à un défi difficile. Ils doivent faire preuve d'habileté et utiliser intelligemment les ressources dont ils disposent. Le système du sport d'élite suisse, confronté à des exigences croissantes, ainsi qu'à des défis de plus en plus importants, s'est sensiblement transformé. Diverses mesures ont ainsi été mises en œuvre au niveau national afin de maintenir la compétitivité de la Suisse dans les grandes manifestations internationales. On trouvera dans l'annexe 1 quelques-unes des étapes charnières de cette évolution depuis les années 1990.

Le présent rapport entend proposer un état des lieux de ce système tel qu'il fonctionnait en 2011. Il ne s'intéresse pas au sport paralympique. Le terme «sport d'élite» est employé au sens du rapport explicatif sur la révision totale de la loi fédérale encourageant la gymnastique et les sports (loi sur l'encouragement du sport) du 17 mars 1972. Le sport d'élite représente ici un sous-ensemble du sport. Il se caractérise par une compétitivité marquée, par la recherche de performances maximales et par une émulation internationale. Le rapport se base sur un inventaire. Les réponses données par les athlètes, les entraîneurs et les chefs du sport d'élite permettent de se faire une idée précise de l'atmosphère qui règne dans le monde du sport de haut niveau suisse. Des réflexions analytiques et une sélection de comparaisons internationales placées en fin de chapitres complètent ce tableau. Pour des raisons de lisibilité, les données SPLISS-CH 2011 (voir le chapitre 12) ne sont pas toujours explicitement citées.

Le rapport s'intéresse dans un premier temps à l'évolution du marché des médailles olympiques et aux objectifs de médailles en Suisse. Il présente ensuite les domaines où le système du sport d'élite suisse a remporté le plus de victoires. Il est souvent utile d'analyser séparément les sports d'hiver et d'été. Le rapport s'interroge également sur les liens et l'importance de ces domaines dans le succès de la Suisse; en revanche, il ne fait qu'effleurer la problématique de la finalité du sport d'élite et de son influence sur la société. Ce travail propose une présentation détaillée du système du sport d'élite suisse, offrant ainsi une base de discussion sur les moyens de favoriser sa pérennité.

2. Le marché des médailles olympiques

Les médailles olympiques constituent des distinctions très convoitées. Dans bien des sports, elles représentent la plus prestigieuse récompense qu'un athlète puisse obtenir au cours de sa carrière. Il n'est d'ailleurs pas rare d'évaluer l'efficacité du système de sport d'élite d'un pays à l'aune de ses victoires aux Jeux Olympiques. Les nations participantes et les représentants des différents sports s'efforcent dès lors de faire de leur mieux: l'honneur national est en jeu.

Evolution du marché des médailles olympiques

L'objectif de médailles olympiques n'est pas gravé dans le marbre. Il a ainsi évolué de façon très nette au cours des 25 dernières années. Depuis 1988, le nombre d'épreuves d'été comme d'hiver a sensiblement augmenté. De nouveaux sports ont été intégrés au programme olympique; d'autres, relevant de la plus pure tradition olympique, se sont enrichis de nouvelles disciplines. Très peu d'épreuves ont été supprimées. On trouvera la liste complète des épreuves olympiques en se reportant aux annexes 2 et 3.

La figure 2.1 montre que le nombre d'épreuves disputées aux Jeux Olympiques d'été a augmenté de 24 % entre 1988 et 2012, passant de 244 à 302. Un peu plus de la moitié de ces 58 nouvelles épreuves résultent de l'intégration de nouveaux sports (badminton, beachvolley, canoë/slalom, BMX, taekwondo, trampoline et triathlon). Durant la même période, certains sports (lutte gréco-romaine, tir et voile) ont vu le nombre de leurs épreuves olympiques diminuer. Pour d'autres encore, ce chiffre a connu des variations, commençant par augmenter avant de chuter à nouveau. Dans la figure 2.1, les sports ont été répartis en trois catégories: la première comprend les huit grands sports traditionnels («Grands sports trad.»), la deuxième, les 23 autres sports traditionnels («Autres sports trad.») et la troisième, les huit nouveaux sports («Nouveaux sports»).

Figure 2.1: Nombre d'épreuves disputées aux Jeux Olympiques d'été, 1988-2012

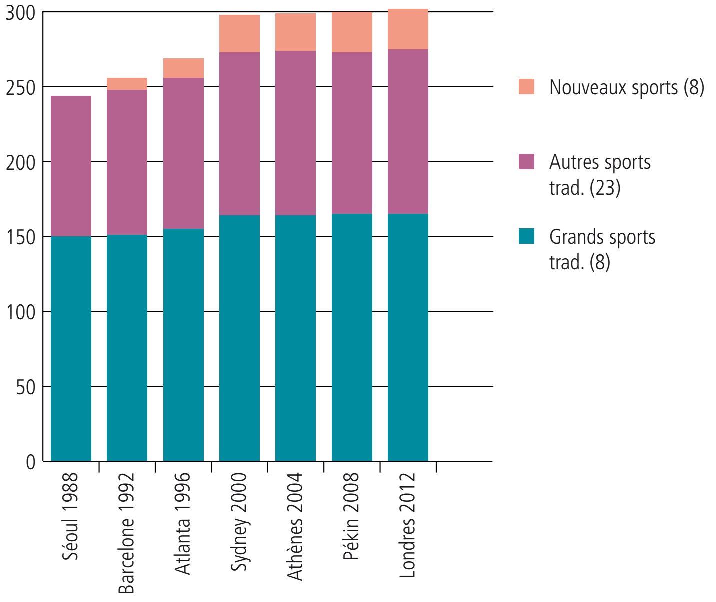

Source: graphique établi par les auteurs d'après les données du CIO (2012)

La figure 2.2 met en évidence l'évolution rapide du programme des Jeux Olympiques d'hiver. Le nombre d'épreuves disputées est passé de 46 à 98 entre 1988 et 2014, soit une augmentation de 113 %. Cette croissance phénoménale s'explique principalement par l'inscription de nouveaux sports au programme des Jeux (patinage de short track, ski acrobatique, snowboard, curling et skeleton). Un tiers des épreuves des Jeux Olympiques de Sotchi 2014 se disputent dans des sports n'ayant été intégrés au programme olympique qu'après les Jeux de Calgary, en 1988. Certains sports profitent plus que d'autres de cet élargissement du programme. Ainsi, le biathlon et le ski de fond sont les deux sports traditionnels qui affichent la plus forte croissance, tandis que le ski alpin est le seul à ne compter aucune nouvelle épreuve depuis 1988. Du côté des nouveaux sports, ce sont le ski acrobatique, le snowboard et le short track qui ont connu le développement le plus important. La figure 2.2 illustre ces évolutions en reprenant la répartition adoptée dans la figure 2.1.

Le programme des Jeux Olympiques d'été est plus dense que celui des Jeux Olympiques d'hiver. Ces derniers disposent encore d'une marge de progression concernant le nombre d'épreuves, de nations participantes, d'athlètes, etc. Ce n'est pas le cas des Jeux Olympiques d'été, dont le potentiel de croissance est épuisé. Par ailleurs, le nombre d'épreuves étant trois fois plus élevé en été qu'en hiver, l'ajout de nouvelles épreuves aux Jeux Olympiques d'hiver entraîne automatiquement une plus forte croissance relative de ces derniers.

Une tendance inflationniste?

Au cours des dernières années, le marché des médailles olympiques n'a cessé de s'étendre. On pourrait penser que cette multiplication des épreuves va de pair avec une dévalorisation des titres. Or le monde du sport ne montre pratiquement aucune tendance inflationniste de ce type. Il reste en effet très difficile de remporter une médaille, car au moment où celles-ci se sont multipliées, le nombre de nations participantes a lui aussi considérablement augmenté.

Ainsi, lors des Jeux d'été de 1988, on comptait 159 Comités Nationaux Olympiques (CNO) représentés, dont 52 ont remporté des médailles (33 %). En 2012, les CNO participants étaient au nombre de 204, dont 85 ont décroché au moins une médaille (42 %, voir la figure 2.3). On peut donc en conclure que les CNO sont toujours plus nombreux à se partager les médailles. La compétition n'en devient que plus forte parmi les CNO qui postulent aux médailles, ce qui accroît la valeur des récompenses.

Figure 2.2: Nombre d'épreuves disputées aux Jeux Olympiques d'hiver, 1988-2014

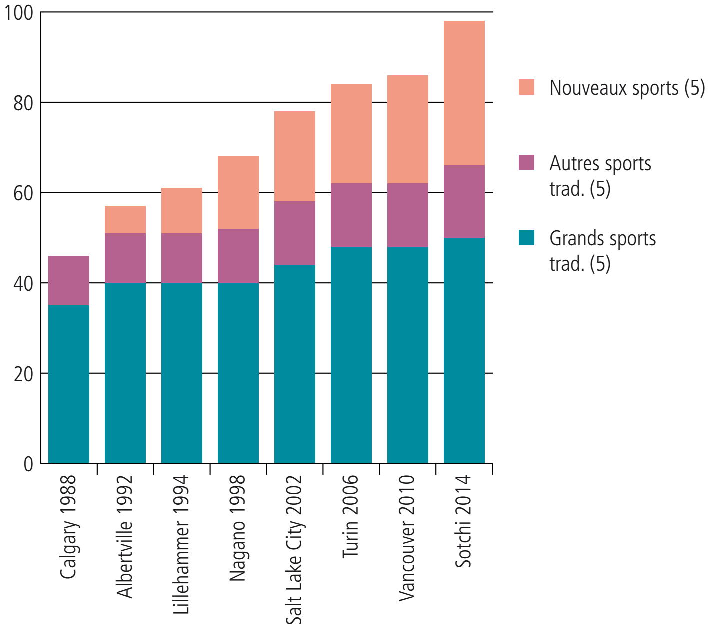

Source: graphique établi par les auteurs d'après les données du CIO (2012)

Figure 2.3. Evolution du nombre de CNO participant aux Jeux Olympiques d'été, 1988-2012

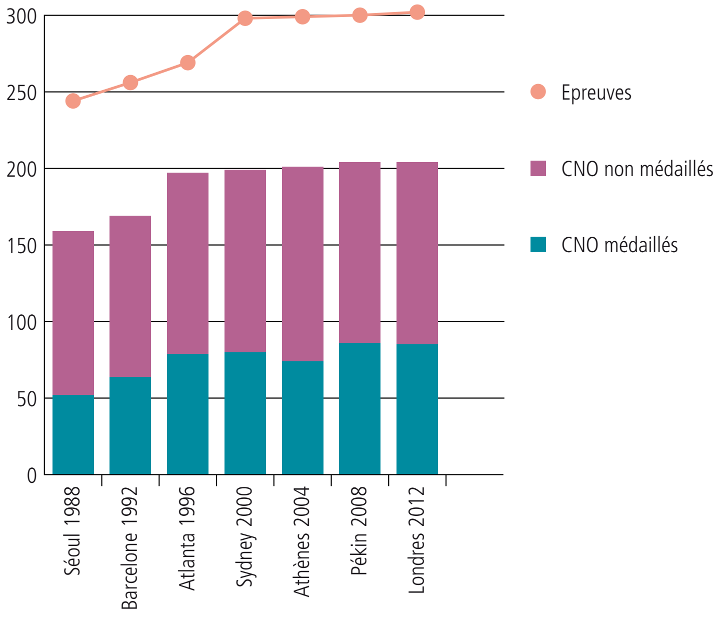

Source: graphique établi par les auteurs d'après les données du CIO (2012)

La figure 2.4 montre l'évolution du nombre de CNO participant aux Jeux Olympiques d'hiver. Parmi les 57 CNO présents aux JO de 1988, 17 ont obtenu des médailles, soit 30 % des participants. En 2010, ils étaient 82 à prendre part aux Jeux et 26 à remporter une médaille (31 %). La proportion de comités décorés est donc restée sensiblement la même sur toute la période, contrairement à ce que l'on observe pour les JO d'été. En effet, si les médailles en jeu sont plus nombreuses en hiver, le nombre de pays en mesure de les décrocher est en revanche moins important. Par conséquent, la valeur de chaque médaille diminue.

Si le CIO parvient assez facilement à recruter de nouveaux CNO susceptibles de remporter des médailles lors des Jeux Olympiques d'été, il rencontre plus de difficultés lorsqu'il s'agit des JO d'hiver. Les obstacles que les nouveaux CNO doivent surmonter pour entrer sur ce marché particulier sont en effet nettement plus importants (conditions géo-climatiques, infrastructures, connaissances techniques, méthodes d'entraînement, etc.). Les nations fortes d'une longue tradition de victoires olympiques aux JO d'hiver continuent donc à se partager le gâteau des médailles.

Approche stratégique du marché des médailles

Du fait de l'apparition de nouvelles disciplines et de nouveaux sports, les sports traditionnels perdent de leur importance sur le marché des médailles. Cette évolution est particulièrement nette pour les JO d'hiver. Les sports traditionnels que sont le ski alpin et le patinage de vitesse ont vu leur part reculer entre 1988 et 2014, chutant de 22 à 10 % pour la première et de 22 à 12 % pour la seconde. A l'inverse, les deux nouveaux sports olympiques – ski acrobatique et snowboard – sont ceux qui se sont le plus développés, passant respectivement de 4 à 10 % entre 1992 et 2014, et de 6 à 10 % entre 1998 et 2014.

Grâce à la pression exercée par les grandes chaînes de télévision, de nouveaux sports et disciplines trouvent peu à peu leur place dans le programme olympique. Ils attirent les médias, qui y voient une façon d'améliorer les scores d'audience internationaux des Jeux Olympiques. Toutefois, ces «nouvelles médailles» ne présentent pas le même intérêt pour tous les pays. Certaines sont plus convoitées que d'autres, en raison des traditions nationales ou de l'ancrage dans la société. De même, certaines nouveautés ne suscitent qu'un engouement limité dans une population donnée, si bien qu'une médaille ne produit pas les effets escomptés dans le pays concerné (enthousiasme, développement de la discipline). Conscientes de ces aspects, les nations mettent au point des stratégies différentes.

Plusieurs études ont mis en lumière la «course à l'armement» à laquelle se livrent de nombreux pays dans le domaine du financement et de l'encouragement du sport d'élite (De Bosscher et al., 2008; Hogan & Norton, 2000; Houlihan & Green, 2008). Les investissements de la France dans ce secteur spécifique du sport ont ainsi augmenté d'environ 137 % au cours des dix dernières années, ceux du Canada de 89 %, ceux de l'Australie de 86 % et ceux de la Finlande de 65 % (De Bosscher et al., 2013a). Dans la même période, l'augmentation des dépenses totales consenties par la Suisse en faveur de son sport d'élite est restée bien inférieure à ces chiffres. Si le pays veut pouvoir fêter d'autres victoires olympiques à l'avenir, il lui faut réagir à cette évolution internationale, car les moyens financiers constituent un facteur de réussite très puissant dans ce genre de compétitions.

Figure 2.4: Evolution du nombre de CNO participant aux Jeux Olympiques d'hiver, 1988-2010

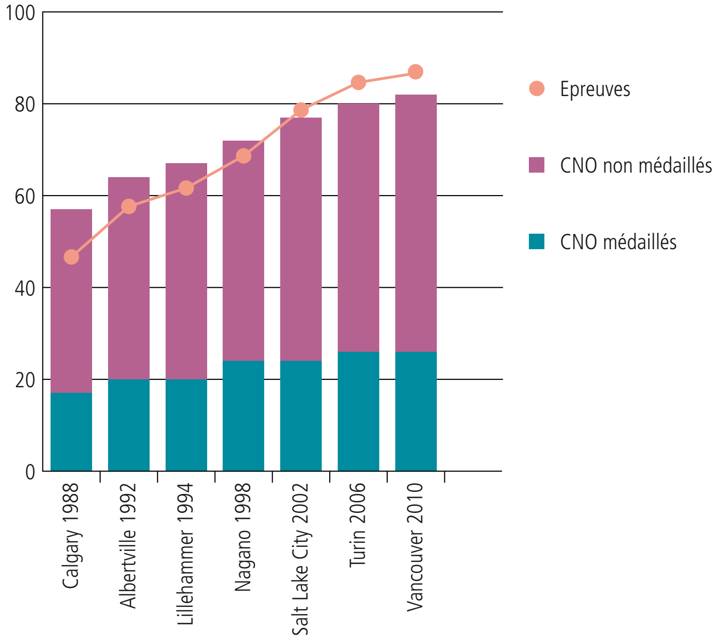

Source: graphique établi par les auteurs d'après les données du CIO (2012)

A cela s'ajoute le fait que les nouveaux sports olympiques ne jouissent pas de la même popularité sur la scène internationale. Leur développement demande parfois beaucoup de temps. Pourtant, au tableau des médailles, une récompense dans une discipline nouvelle telle que le ski cross a strictement la même valeur qu'une distinction en ski alpin. De même, la concurrence à l'œuvre dans les nouveaux sports est souvent bien moindre que celle qui règne dans les disciplines traditionnelles (Weber *et al.*, 2013). Enfin, une médaille récompensant un sport d'équipe comme le hockey sur glace ne compte que pour une récompense dans le tableau des distinctions. Mais son impact est décuplé par le fait que chaque athlète reçoit une médaille individuelle. Les CNO qui ne se préoccupent actuellement que de leur classement au tableau des médailles vont devoir prendre en compte ces éléments pour fixer leurs objectifs.

Avant de se lancer dans la course aux distinctions, de nombreux pays prennent d'abord le temps d'analyser le marché des médailles en fonction de l'impact et des niveaux d'influence de chaque sport et discipline. Ils s'appuient ensuite sur leurs conclusions pour tenter d'obtenir des parts de marché, comme le ferait une entreprise du secteur économique. C'est particulièrement vrai pour les JO d'hiver: le marché des médailles est ici en forte expansion et les pays doivent déterminer s'il convient toujours d'encourager les sports traditionnels, ou s'il est préférable de soutenir de façon ciblée le développement de nouveaux sports, où les récompenses sont nombreuses. Pour les Jeux d'été, en revanche, ils privilégient plutôt les points forts historiques, le développement du programme olympique étant ici moins dynamique.

Ce débat prend une importance croissante, en Suisse également. De fait, si les Helvètes veulent remplir leur objectif de médailles internationales, ils doivent élaborer une approche stratégique du marché. Pour cela, il leur faut mener une analyse approfondie de leurs objectifs, acquérir une connaissance spécifique des disciplines dans lesquelles ils souhaitent dorénavant conquérir des médailles et étudier la situation de concurrence. Par ailleurs, les succès sportifs ne constituent pas le seul critère pris en considération pour décider d'encourager plus ou moins, voire pas du tout, un sport. D'autres aspects entrent en compte, parmi lesquels l'importance sociale que le sport revêt dans le pays. Or, il va sans dire que la Suisse souhaite remporter les médailles chères à sa population.

3. Le sport suisse: présentation d'ensemble

Les médailles récompensent les vainqueurs du combat que se livrent les sports nationaux, guidés par des consignes précises. En Suisse, ce sont Swiss Olympic Association (ci-après dénommée Swiss Olympic) et les fédérations qui la composent qui tentent d'atteindre les objectifs de médailles fixés. Elles bénéficient du soutien subsidiaire de l'Office fédéral du sport (OFSPO) et d'autres partenaires de droit public. Les instances en charge du système sportif orientent leurs stratégies de manière à s'approcher au maximum des buts recherchés.

Performances du sport suisse

Les objectifs stratégiques de la Suisse dans le domaine du sport d'élite sont définis dans le Concept du sport d'élite suisse 2010. Concernant les Jeux Olympiques, la consigne est de se classer parmi les huit premières nations en hiver («Top 8») et parmi les 25 premières en été («Top 25») (Swiss Olympic, 2010a). Le total des médailles remportées est important.

La Suisse est par tradition un pays de sports d'hiver, comme le démontre le nombre de médailles obtenues entre 1988 et 2012. Durant cette période, les médailles en général et celles d'or en particulier ont été plus nombreuses en hiver qu'en été (68 contre 37 pour les premières, 25 contre 11 pour les secondes) (statistiques établies par les auteurs d'après les données du CIO, 2012). L'écart entre hiver et été se creuse encore si l'on se penche sur le tableau des médailles. Lorsque celui-ci est pondéré, une médaille d'or pèse plus lourd que plusieurs médailles d'argent réunies. Ce type de comparaison est largement répandu dans les médias. Toutefois, il est également possible d'analyser les résultats à l'aide du nombre total de récompenses obtenues. Dans un tel tableau – non pondéré –, toutes les médailles ont la même valeur.

La figure 3.1 compare les résultats de la Suisse avec l'objectif fixé («Top 25») lors des Jeux Olympiques d'été organisés entre 1988 et 2012. Le nombre de médailles d'or à gagner est constant depuis 1996 (trois). Il en va différemment pour le total visé, qui a eu tendance à augmenter depuis 1988, passant de quatre à huit puis à dix. Les performances de la Suisse ont connu un pic lors des Jeux Olympiques d'Atlanta, en 1996, année où l'objectif du «Top 25» a été largement dépassé pour les médailles d'or. Quatre ans plus tard, à Sydney, seul le total des médailles a permis à la Suisse de se classer parmi les 25 premières nations. Elle n'a plus retrouvé cette place depuis, arrivant même parfois loin derrière.

Figure 3.1: Objectifs et résultats de la Suisse aux Jeux Olympiques d'été, 1988-2012

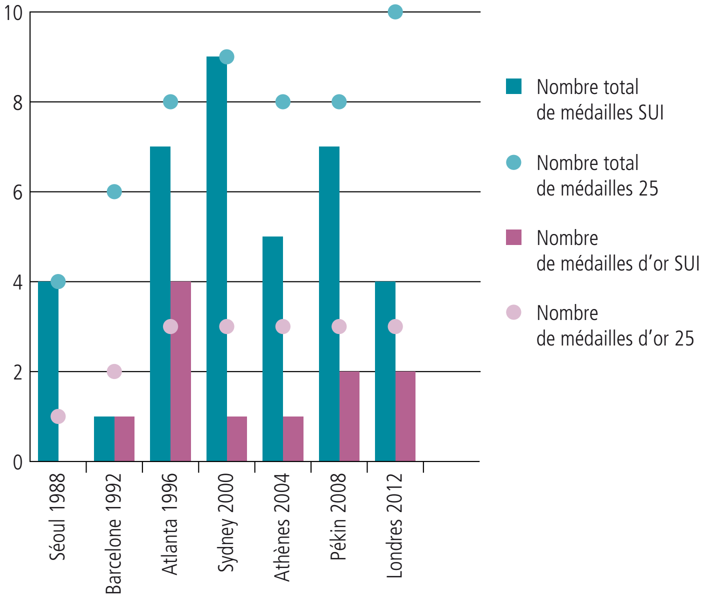

Source: graphique établi par les auteurs d›après les données du CIO (2012)

La figure 3.2 compare les résultats des athlètes suisses à l'objectif fixé pour les Jeux Olympiques d'hiver («Top 8») sur la période 1988-2010. Ici, le nombre de médailles à remporter a eu tendance à augmenter, aussi bien pour l'or (de deux à cinq médailles) que pour les trois métaux confondus (de six à onze, puis à quatorze médailles). Lors des Jeux de Calgary, en 1988, la Suisse a remporté 15 médailles, se classant ainsi parmi les trois premières nations, derrière l'URSS et la RDA. L'objectif du «Top 8» était largement réalisé. Si ce niveau n'a plus jamais été atteint par la suite, la Suisse s'est toutefois imposée dans le milieu olympique. Hormis lors des JO d'Albertville en 1992 et de Nagano en 1998, les sportifs ont toujours atteint l'un des deux objectifs, remportant le nombre soit de médailles d'or, soit de médailles en général qui leur était imposé, voire les deux lors des JO de Lillehammer en 1994 et de ceux de Turin en 2006. Quant aux Jeux de Vancouver, en 2010, ils resteront à jamais une «page dorée» de l'histoire du sport helvétique.

En résumé, si l'on se réfère aux exigences fixées pour la période étudiée, force est de constater que les athlètes suisses doivent remporter plus de médailles, aussi bien en hiver qu'en été.

Figure 3.2: Objectifs et résultats de la Suisse aux Jeux Olympiques d'hiver, 1988-2010

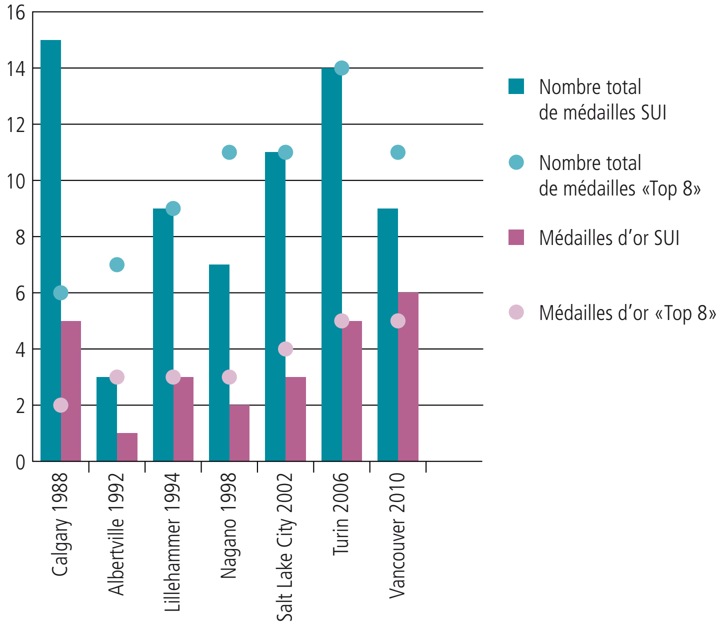

Source: graphique établi par les auteurs d'après les données du CIO (2012)

Tableau 3.1: Médailles suisses par discipline sportive lors des Jeux Olympiques, 1988-2012

Jeux Olympiques d'été, 1988-2012	Nombre	Répartition
Cyclisme (dont VTT)	11	30 %
Sports équestres	6	16 %
Aviron	4	11 %
Triathlon	4	11 %
Autres	12	32 %
**Total**	**37**	**100 %**

Jeux Olympiques d'hiver, 1988-2010	Nombre	Répartition
Ski alpin	25	37 %
Bobsleigh	11	16 %
Snowboard	9	13 %
Curling	5	7 %
Autres	18	27 %
**Total**	**68**	**100 %**

Source: tableau établi par les auteurs d'après les données de Stamm & Lamprecht (2012)

Sports clés pour le succès international

Certains sports jouent un rôle décisif dans la réalisation des objectifs. Lors des Jeux Olympiques d'été organisés entre 1988 et 2012, les athlètes des quatre disciplines sportives les plus performantes (cyclisme, sports équestres, aviron et triathlon) ont remporté à eux seuls 68 % des médailles, le cyclisme se taillant toutefois la part du lion dans ces succès (30 % de toutes les médailles). De même, lors des Jeux Olympiques d'hiver organisés entre 1988 et 2010, les adeptes des quatre spécialités sportives les plus performantes (ski alpin, bobsleigh, snowboard et curling) ont remporté à eux seuls 73 % des médailles suisses, dont une majeure partie grâce au ski alpin (37 % des médailles remportées au cours de cette période). Le tableau 3.1 présente les sports olympiques dans lesquels la Suisse a remporté ses victoires au cours des années passées.

Eté comme hiver, une grande partie des médailles sont décrochées par les athlètes d'un seul et unique sport. La plupart des sports les plus prolifiques en victoires sont des disciplines individuelles qui demandent d'importants moyens financiers, leur pratique nécessitant un matériel et/ou des infrastructures considérables. Parmi les sports où les Suisses excellent, trois ont été ajoutés au programme olympique au cours de la période étudiée, à savoir le triathlon, le curling et le snowboard. La Suisse semble posséder un avantage compétitif. Elle s'adapte en effet très vite lorsqu'il s'agit d'exploiter de nouveaux potentiels pour le tableau des médailles.

Figure 3.3: Présentation schématique des acteurs du sport suisse

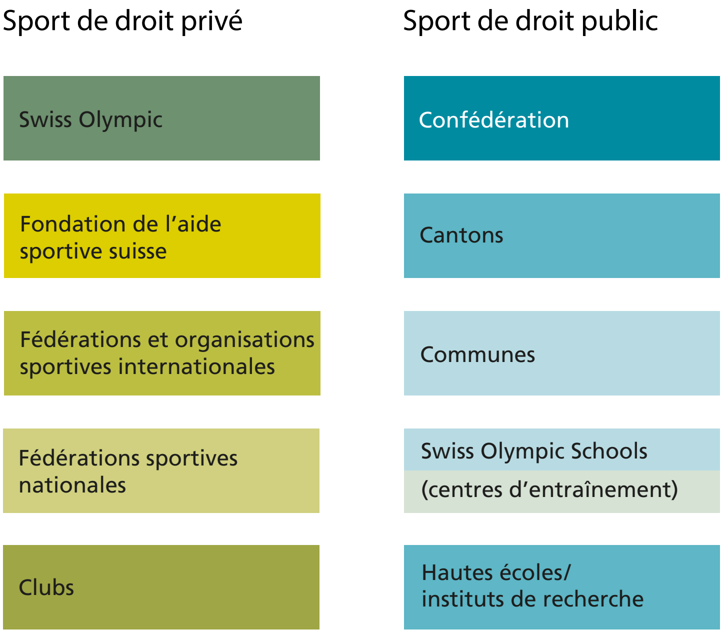

Source: SPLISS-CH 2011

Les principaux acteurs du sport suisse

Que désigne l'expression «sport suisse» dans le présent rapport? Il vaut la peine de s'arrêter un moment sur chacun des acteurs au cœur du système d'encouragement du sport d'élite. Les couleurs employées seront reprises dans le modèle des flux financiers présenté au chapitre 4.

Si l'on se base sur une représentation établissant une distinction nette entre les institutions de droit privé et celles de droit public, on citera en premier lieu Swiss Olympic, la fondation de l'aide sportive suisse, les fédérations internationales et nationales, et les clubs. De l'autre côté, on trouve la Confédération, les cantons et les communes, ainsi que les écoles de sport ou les hautes écoles et les instituts de recherche subventionnés. Ces instances sont représentées de façon schématique dans la figure 3.3.

Les paragraphes suivants permettent de reconstituer les liens complexes qui unissent les différents acteurs. Pour faciliter la compréhension, certains partenaires n'apparaîtront que dans le chapitre 4.

Swiss Olympic, fédérations et clubs

C'est Swiss Olympic qui fixe l'objectif de médailles national. Association faîtière du sport de droit privé, elle regroupe 82 fédérations (état: 2010). Ces dernières forment le Parlement du sport, chargé d'élire le Conseil exécutif de Swiss Olympic. Quant au Secrétariat, il veille à l'application des stratégies adoptées.

Swiss Olympic pilote certains domaines essentiels du sport d'élite suisse. L'association s'efforce d'assurer une coordination nationale et de garantir une certaine qualité. Pour ce faire, elle édicte les conditions à remplir pour l'octroi des subventions versées aussi bien aux clubs qu'aux athlètes et décerne divers labels. Elle soutient en outre les fédérations sportives nationales et – avec l'appui de la fondation de l'aide sportive suisse – les athlètes.

La politique nationale du sport d'élite est portée par les fédérations sportives nationales. Celles-ci sont en effet chargées d'élaborer et de mettre en œuvre le concept du sport d'élite et de la relève dans les sports concernés. En collaboration avec Swiss Olympic, elles sélectionnent les athlètes de haut niveau et les jeunes espoirs les plus doués (Swiss Olympic, 2009). La structure de l'organisation et sa prééminence dans le domaine du sport de droit privé garantissent leur autonomie, fondée sur la conviction que les fédérations sportives nationales sont les plus compétentes pour répondre aux exigences spécifiques à leur sport. Les fédérations nationales sont solidement liées aux organisations faîtières qui les chapeautent au niveau international.

Les fédérations regroupent les clubs, qui sont à la base du système du sport d'élite. La Suisse se distingue par une longue tradition et un très grand nombre de clubs sportifs organisés. Forts de 20 065 clubs et de 1,6 million de membres actifs (sans double comptage), ils sont très répandus et solidement ancrés dans la société (Lamprecht *et al.*, 2011).

En sa qualité d'association faîtière, Swiss Olympic défend les intérêts du sport d'élite de droit privé vis-à-vis de l'opinion publique, des autorités, ainsi que des organisations nationales et internationales. Le CIO est son principal interlocuteur au niveau international. Swiss Olympic a également passé avec l'OFSPO deux conventions sur la coopération et les prestations, régissant l'encouragement du sport et la collaboration des domaines de droit public et privé. De même, le Concept du sport d'élite suisse publié en 2010 a été élaboré sous l'égide de Swiss Olympic, bien que l'OFSPO ait été fortement impliqué afin de garantir la continuité de l'encouragement. Il a en outre été soumis pour consultation aux fédérations sportives nationales.

Confédération, cantons et communes
L'OFSPO représente le sport de droit public sur la scène nationale. Il se compose d'un office (politique du sport), du programme Jeunesse+Sport (J+S), de la Haute école fédérale de sport de Macolin (HEFSM), ainsi que de centres sportifs et de formation pour tout le pays à Macolin et à Tenero (Centro sportivo nazionale della gioventù Tenero [CST]). Avec le concept de politique du sport qu'il a présenté en 2000 et son implication dans l'élaboration du Concept du sport d'élite suisse en 2010, l'OFSPO participe à la définition des directives nationales pour la politique du sport.

L'OFSPO encourage directement les fédérations sportives au moyen de subventions et d'encouragements à la performance, définis dans la convention de prestations passée avec Swiss Olympic. Il apporte également un soutien subsidiaire aux fédérations, clubs et sportifs par le biais de différents vecteurs. Il encourage les fédérations sportives de façon aussi bien directe – en proposant des services et des formations aux entraîneurs et aux sportifs – qu'indirecte, par l'intermédiaire de Swiss Olympic. Il assume ainsi des fonctions de pilotage et de garant de la qualité.

En Suisse, les 2551 communes (état: 2010) et les 26 cantons et demi-cantons constituent les principaux soutiens du sport de droit public. Ils encouragent le sport d'élite en construisant et en entretenant les infrastructures, en organisant des manifestations sportives et en favorisant la promotion de la relève (écoles de sport, centres d'entraînement et subventions).

Les écoles de sport et les centres d'entraînement des fédérations jouent également un rôle important dans ce domaine particulier du sport. Les centres d'entraînement sont soutenus par différents acteurs – bien souvent les communes dans lesquelles ils sont situés, et parfois même les cantons. Ces derniers versent aussi d'importants moyens financiers aux hautes écoles et autres instituts de recherche.

La Confédération promulgue des lois et des ordonnances qui s'appliquent au niveau aussi bien cantonal que régional. Celles-ci servent de point de départ aux lois d'encouragement du sport propres à chaque canton, ainsi qu'aux mesures de mise en œuvre qui en découlent. Si la Confédération met à disposition des cantons les concepts de l'encouragement du sport (infrastructure, manifestations sportives, etc.), ces derniers décident en revanche librement de reprendre ou non ces outils et de les développer. De même, ils répartissent comme ils l'entendent les moyens dont ils disposent (impôts et bénéfices des loteries). C'est à l'école que l'ancrage légal du sport est particulièrement visible, notamment au travers des trois heures de sport imposées tout au long de la scolarité obligatoire. Contrairement au fonctionnement général du système scolaire, cette contrainte est dictée par la loi sur l'encouragement du sport en vigueur au niveau fédéral. Les élèves ont également la possibilité de pratiquer des sports scolaires facultatifs, une offre encouragée par J+S. Un échange formel entre l'OFSPO et Swiss Olympic a lieu par l'intermédiaire des services des sports de chaque canton et de leurs collaborateurs en charge de la promotion de la relève.

La Suisse face au défi de la coopération et du pilotage
Le sport national suisse doit remplir un objectif de médailles très élevé, qu'il ne pourra atteindre sans une coordination renforcée à l'échelle nationale. Les efforts à déployer pour remporter une victoire aux Jeux se sont considérablement accrus, dépassant la seule organisation de la délégation olympique: le développement et la «construction» d'un athlète de pointe représentent plus souvent huit que quatre ans de travail systématique. Le sport suisse s'efforce de s'adapter à ces évolutions, comme le prouvent le concept du sport d'élite suisse de 2010 mentionné plus haut d'une part, et le concept de performance récemment lancé d'autre part.

Pour décrocher des médailles aux Jeux Olympiques, il faut être prêt à investir énormément. La route qui mène à la plus haute marche du podium dure dix ans et demande 10 000 heures d'entraînement aux meilleurs sportifs (Ericsson, 2005). De même, pour bénéficier d'avantages stratégiques dans une discipline sportive, il faut une préparation de longue haleine – c'est particulièrement vrai pour le développement des structures de soutien. Il s'écoule souvent des années avant que les mesures adoptées portent leurs fruits. La stabilité du système peut dans un premier temps paraître un handicap; pourtant, sans elle, il n'y a pas d'investissement à long terme possible. En effet, la stabilité engendre la confiance, indispensable au développement des athlètes et de disciplines entières. Ce n'est donc pas un hasard si la Suisse figure parmi les pays qui comptent le plus grand nombre de participants aux Jeux Olympiques par habitant (Shibli et al., 2013).

Les Suisses accordent une grande valeur à la liberté économique. Dans le domaine de l'économie comme dans celui du sport, les entrepreneurs jouissent d'un grand crédit. Dans le monde sportif, la très grande capacité d'initiative des athlètes, des organisateurs de manifestations et des fédérations n'est plus à démontrer. Par ailleurs, le succès des Jeux Olympiques d'hiver de 1988 a prouvé que la Suisse, avec son système sportif basé quasi exclusivement sur l'initiative privée, pouvait tout à fait rivaliser avec les grandes nations qui confient la gestion de leur système sportif à l'Etat. Ce dernier ne devrait intervenir dans le sport d'élite que là où le marché a échoué, tandis que les solutions privées devraient avoir toute latitude pour s'épanouir.

L'idée d'une direction politique centralisée est tout aussi inhabituelle en Suisse. Les décisions sont prises selon des principes fédéraux et les interventions centralistes de l'Etat sont le plus souvent accueillies avec scepticisme. La Confédération doit mener un long travail de persuasion auprès des cantons et des communes, les décisions fédérales devant pouvoir emporter une adhésion aussi large que possible. C'est de ces particularités que le système tire sa grande stabilité et le fort consensus sur lequel il repose.

Le sport d'élite porté par les médias vit dans l'instant, il exige une action sans compromis. Au contraire, le sport de droit public mise sur la durabilité et le consensus. Les initiatives privées et les traditions fédérales sont toujours menacées par la dilution des mesures qu'entraînent les solutions de compromis, au niveau tant stratégique que financier. L'encouragement du sport d'élite oscille ainsi entre direction imposée, volonté d'encourager et propositions passives d'organisation. Cette complexité est une des caractéristiques du système suisse.

Mais cette situation difficile ne doit pas inciter à sous-estimer les capacités de la Suisse en matière d'encouragement du sport. La Suisse est certes un petit pays dans le domaine du sport, mais elle peut compter sur un excellent réseau. Les informations circulent correctement dans le système. La concurrence internationale contraint les acteurs à l'efficacité. La coordination horizontale, relativement peu dynamique, constitue une garantie contre une orientation trop radicale du système d'encouragement. La coordination verticale, quoique d'une efficacité inégale, assure une certaine émulation entre les différents acteurs. Bien qu'il soit souvent critiqué, notre système peut tout à fait s'avérer efficace à moyen et à long terme.

4. Le financement du succès olympique

Les médailles ne tombent pas du ciel. Depuis les années 1980, les Jeux Olympiques ont été marqués par une croissance phénoménale des budgets, mais aussi du nombre de disciplines sportives, d'athlètes, de CNO, etc. Pour les pays qui se targuent d'être des nations sportives et fixent des objectifs de médailles, il n'y a pas d'autre issue que d'investir les moyens nécessaires pour y parvenir. Quel prix une nation est-elle prête à payer pour remporter une médaille? Cette dépense peut-elle se faire au détriment d'autres disciplines ou de l'encouragement du sport de masse en Suisse? Un rapide coup d'œil aux flux financiers de l'année 2010 fournit des éléments de réponse à ces questions.

Sponsors et médias: les partenaires du sport d'élite
Le sport moderne ne peut se passer des sponsors et de la télévision. Ces derniers n'ayant toutefois aucun intérêt à publier leurs chiffres, il n'est pas facile de trouver des informations sur les activités de sponsoring. Les contrats reposent en effet sur le principe d'une confidentialité réciproque.

La participation des médias au financement du sport est difficile à déterminer avec précision. La Télévision Suisse en est le principal moteur. Il suffit qu'elle filme le monde du sport pour que celui-ci trouve des financeurs parmi les sponsors et les publicitaires. Chaque année, les chaînes de télévision helvétiques paient entre 40 et 60 millions de francs en droits de retransmission, droits d'auteurs et droits médiatiques pour les manifestations sportives. Les dépenses sont plus élevées les années paires, en raison des événements de grande envergure tels que JO et Coupes du monde ou d'Europe de football – en 2010, les droits de retransmission pour les Jeux Olympiques d'hiver de Vancouver et pour la Coupe du monde de football en Afrique du Sud ont ainsi atteint 15,3 millions de francs (SRG SSR, 2011). Une partie de ces fonds est reversée au sport suisse (ligues professionnelles, manifestations sportives et fédérations).

Les loteries nationales, pivot du financement
Les deux grandes loteries nationales Swisslos (Suisse alémanique et Tessin) et la Loterie Romande (Suisse romande) occupent une place centrale dans le sport suisse. Swisslos est une coopérative constituée des cantons de Suisse alémanique, du Tessin et du Liechtenstein, qui gèrent des loteries en commun. La Loterie Romande est organisée selon un modèle similaire. La Confédération octroie à ces sociétés un monopole de fait sur le marché des paris, assurant au sport suisse une source de revenus considérables. Les bénéfices réalisés sont redistribués entre les différents cantons sociétaires selon une clé de répartition spécifique (population du canton et chiffre d'affaires), avant d'être reversés à leur fonds de loterie respectif (Bâle-Ville, 2013). Les cantons décident ensuite de la répartition des gains issus du système de loterie. En 2010, environ 27 % des bénéfices nets de Swisslos et de la Loterie Romande ont été affectés au sport, d'élite ou non. Le reste a servi à financer des projets culturels et d'utilité publique par le biais des cantons.

Les sociétés de loterie nationales distribuent leurs fonds de manière directe et indirecte. Elles financent ainsi indirectement le sport en passant par l'intermédiaire de la Société du Sport-Toto. En revanche, elles soutiennent directement l'association SwissTopSport (STS). De même, la Loterie Romande subventionne directement les sports équestres (Association pour le développement de l'élevage et des courses [ADEC]). La Société du Sport-Toto redistribue les fonds qu'elle reçoit à Swiss Olympic, à la Fondation de l'aide sportive suisse ainsi qu'à certains sports (football et hockey sur glace). Dans le domaine du sport, cet argent sert tout autant à financer le sport d'élite (Swiss Football League [SFL]) que le sport de masse (Association suisse de football [ASF]); concernant le hockey sur glace, les aides vont au sport d'élite (National League [NL]). Les autres disciplines sportives sont financées de façon indirecte par les loteries nationales, via Swiss Olympic.

Tableau 4.1: Financement du sport par le biais des sociétés de loterie nationales en 2010

Fédérations sportives/institutions	En millions de CHF
Swiss Olympic	24,6
Football suisse (ASF, SFL)	4,4
Hockey sur glace suisse (SIHA/NL)	2,2
Fondation de l'aide sportive suisse	1,0
SwissTopSport (STS)	1,2
Sports équestres (ADEC)	3,2
Sous-total 1	36,6
**Fonds cantonaux du sport**	**En millions de CHF**
Zone économique de Swisslos (sans la Principauté du Liechtenstein)	84,4
Zone économique de la Loterie Romande	23,7
Sous-total 2	108,1
**Total**	**144,7**

Source: tableau établi par les auteurs d'après les données de Swisslos (2011a)

Le tableau 4.1 présente le financement du sport par les deux sociétés de loterie nationales. Il établit une distinction entre les fédérations sportives/institutions et les fonds cantonaux du sport. En 2010, les loteries nationales ont versé un total de 144,7 millions de francs au secteur sportif. La majeure partie (108,1 millions de francs) a été distribuée par l'intermédiaire des fonds cantonaux du sport (voir le sous-total 2). Les cantons soutiennent le sport de façon directe ou indirecte, via les communes.

Les fonds disponibles pour le sport sont-ils en augmentation? Entre 2001 et 2010, les sommes versées par les deux grandes loteries nationales (Swisslos et la Loterie Romande) aux fédérations sportives/institutions et aux fonds cantonaux du sport ont augmenté de 61% (Swisslos, 2011a). Les fonds alloués aux seuls fonds du sport ont augmenté de 77%, ceux des fédérations sportives/institutions de 29%. Entre 2003 et 2010, Swisslos a augmenté son bénéfice à distribuer de 58%, tandis que la Loterie Romande augmentait le sien de 15%. Celle-ci avait déjà augmenté ses gains de façon très substantielle (+29%) entre 2001 et 2003 (calcul établi par les auteurs d'après les données de la Loterie Romande, 2011 et de Swisslos 2011b). Les chiffres montrent également que les cantons ont renforcé la part relative des bénéfices issus des activités de loterie reversée aux fonds cantonaux du sport.

Le financement du sport d'élite

Les fonds ainsi attribués servent-ils à l'encouragement du sport en général ou profitent-ils principalement au sport d'élite? Ainsi posée, cette question n'a pas de réponse simple. La part du sport d'élite dans les fonds dévolus à l'encouragement du sport est difficile à déterminer, car il n'est pas rare qu'il bénéficie de ces aides de façon indirecte, après bien des détours. Il vaut cependant la peine de modéliser la répartition des financements au sein du sport d'élite et de tenter une répartition, conformément à la logique de la présente étude.

La complexité du modèle des flux financiers alimentant le sport d'élite en Suisse est représentée comme suit dans la figure 4.1:

- On trouve tout d'abord les parties prenantes, réparties en trois catégories: les bailleurs de fonds, les organes de pilotage subsidiaires et les bénéficiaires proprement dits des moyens financiers versés au sport.
- Les sponsors, les médias et les loteries constituent les principaux bailleurs de fonds du sport de haut niveau. Ils sont représentés dans la partie gauche du schéma. Les organisations faîtières internationales financent elles aussi le sport suisse. La Fondation de l'aide sportive suisse figure également sur le diagramme.
- L'association Swiss Olympic et les pouvoirs publics (Confédération, cantons et communes) pilotent le financement du sport de façon subsidiaire. Ils occupent la partie centrale de la figure.
- Les bénéficiaires des subventions versées au sport d'élite suisse sont les clubs et les fédérations sportives nationales. Ils sont représentés dans la partie droite de la figure.
- Les organisateurs d'événements sportifs et les ligues nationales constituent d'autres grands bénéficiaires, eux aussi placés à droite sur la figure.
- Les athlètes et leurs entraîneurs/encadrement bénéficient *in fine* des différentes allocations financières, mais les sommes attribuées varient énormément d'un destinataire à l'autre. C'est pourquoi les entraîneurs et les athlètes percevant des salaires exceptionnellement élevés n'ont pas été pris en compte ici, afin de ne pas biaiser la modélisation.
- Les ménages privés (dont familles, donateurs, etc.) ne figurent pas dans la représentation, car si le soutien financier qu'ils fournissent au sport d'élite est capital, il est pratiquement impossible à appréhender.
- La qualité variable des données présentées dans la fig. 4.1 est indiquée par des abréviations placées devant les chiffres (env., min.).

Les bailleurs de fonds du sport d'élite

En 2010, le montant total du sponsoring sportif suisse atteignait environ 287 millions de francs. Les bailleurs de fonds ont augmenté leur contribution (Sport+Markt, 2011). Les dépenses effectuées dans le domaine du sport d'élite (à l'exclusion du parrainage d'athlètes) s'élevaient à 250 millions de francs au moins. Les sponsors et les médias constituent les principaux bailleurs de fonds du sport d'élite à l'échelle nationale. Les fédérations sportives nationales ont obtenu au moins 58 de ces 250 millions de francs, les clubs au moins 103 millions (Lamprecht *et al.*, 2011), tandis que les organisateurs d'événements sportifs et les ligues nationales touchaient au moins 89 millions de francs (calcul des auteurs). En revanche, la participation des médias nationaux versée directement aux athlètes au titre du sponsoring est difficile à déterminer.

Les organisations internationales (en tête desquelles la FIFA, l'UEFA, le CIO et la FIS) génèrent des revenus en pratiquant la commercialisation centralisée et la vente de droits médiatiques et marketing. Au moins 26,5 millions de francs ainsi récoltés ont été reversés à des associations sportives suisses en 2010, dont la majeure partie au bénéfice du football. Les montants alloués sont fixés en fonction des victoires remportées et par conséquent variables.

En 2010, les sociétés de loterie nationales ont versé 53 millions de francs au sport d'élite, dont 31,6 millions distribués par l'intermédiaire de la SST, 1,2 million via l'association STS et env. 20 millions par l'intermédiaire des fonds cantonaux du sport.

Figure 4.1: Flux financiers alimentant le système du sport d'élite suisse à l'échelle nationale (chiffres 2010 en millions de CHF)

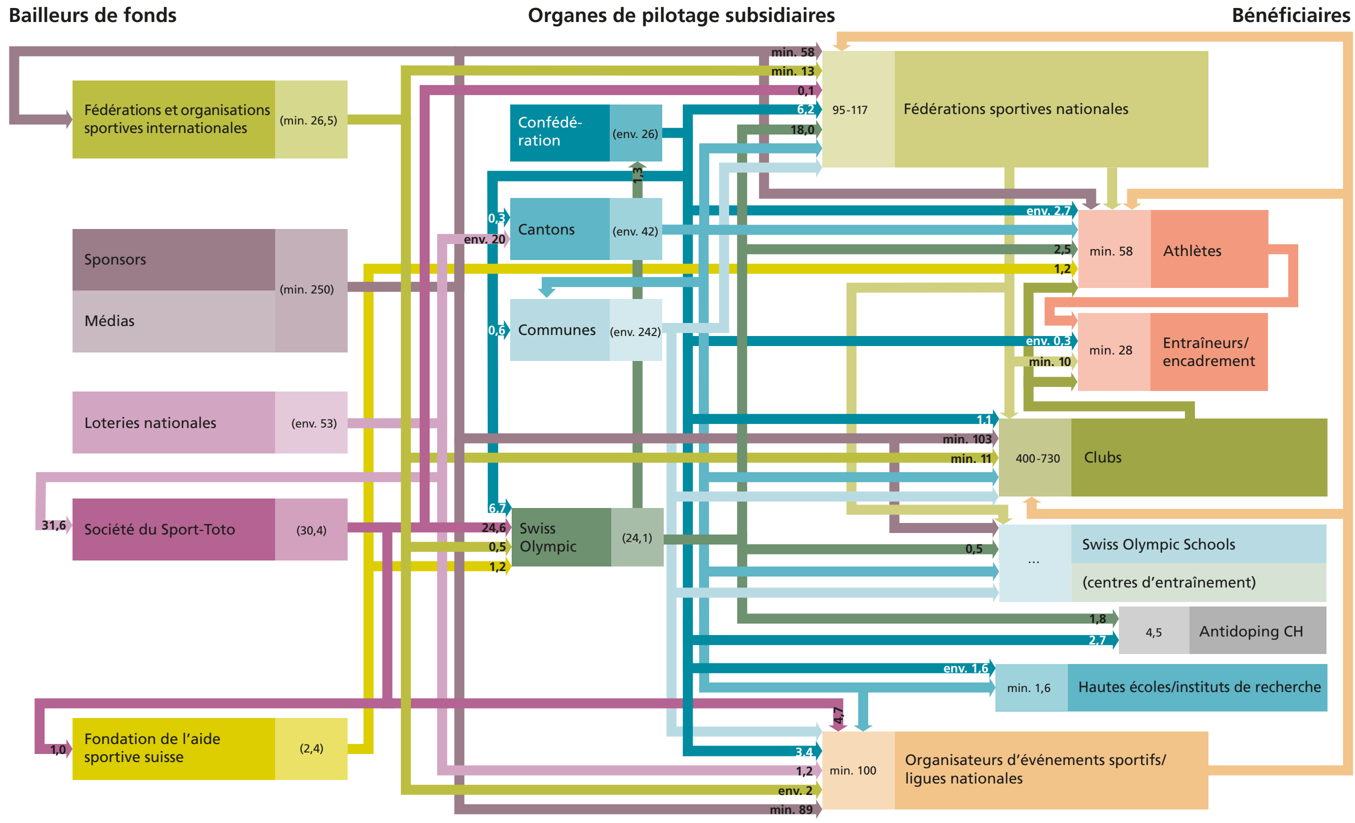

Légende
= financement d'un montant non précisé
18,0 = financement d'un montant précisé
(2,4) = financement versé
4,5 = montant reçu
env. = environ
min. = minimum
… = pas d'indication sur la hauteur du financement reçu

Source: flux établis par les auteurs d'après les données de SPLISS-CH 2011 et de Held (2001)

Les cantons puisent dans les fonds du sport pour promouvoir le sport d'élite et la relève – un peu plus de 18 % des fonds cantonaux présentés dans le tableau 4.1 pour la Suisse alémanique et le Tessin (voir le tableau 4.1: zone économique de Swisslos). Une partie de ce pourcentage est destinée au financement de l'infrastructure du sport d'élite et de la relève. Ce taux est utilisé pour calculer les fonds que les deux sociétés de loterie nationales versent aux cantons au profit du sport d'élite et de la relève. On aboutit ainsi à un résultat d'env. 20 millions de francs.

Concernant la promotion de la relève, c'est d'abord la Fondation de l'aide sportive suisse qui finance les athlètes. Les jeunes talents touchent ainsi 1,2 million de francs par l'intermédiaire de Swiss Olympic et 1,2 million versé directement par la fondation (Fondation de l'aide sportive suisse, 2011).

Financement assuré par des organes de pilotage subsidiaires

Le montant total attribué par Swiss Olympic atteint 43 millions de francs. La figure 4.1 montre que Swiss Olympic subventionne directement le sport d'élite à hauteur de 24,1 millions de francs, dont 18 (incluant la contribution de la Confédération) sont alloués aux fédérations, 2,5 aux athlètes, 1,8 à Antidoping Suisse et 0,5 à des écoles de sport (dont motion Hess). Les principaux financeurs de Swiss Olympic sont la SST (24,6 millions de francs) et la Confédération (6,7 millions de francs) (statistiques établies par les auteurs d'après les données de Swiss Olympic, 2011a).

Le Département fédéral de la défense, de la protection de la population et des sports (DPPS) constitue le principal vecteur par lequel la Confédération encourage le sport de haut niveau. D'autres départements le soutiennent également, mais de façon marginale. Au total, la participation de la Confédération s'élève à près de 190 millions de francs (statistiques établies par les auteurs d'après les données de la Confédération suisse, 2011), dont env. 26 sont attribués au sport d'élite. L'OFSPO en fournit 20, soit la majeure partie (statistiques établies par les auteurs d'après les données de l'OFSPO, 2011a). La Confédération offre également aux athlètes et aux entraîneurs un soutien d'une valeur de 3 millions de francs, sous la forme du Centre d'entraînement pour le sport d'élite de l'armée et du Corps des gardes-frontière (statistiques établies par les auteurs). La recherche sur le sport d'élite est prise en charge par la Confédération via ses divers fonds de recherche, à hauteur de 1,6 million de francs (statistiques établies par les auteurs). Le DPPS apporte aux manifestations sportives organisées en Suisse une aide sous forme de services et de prestations en nature (Ordonnance concernant l'appui d'activités civiles et d'activités hors du service impliquant des moyens militaires – OAM) d'une valeur égale à 1,3 million de francs, à laquelle viennent s'ajouter des contributions marginales accordées par l'OFSPO (DPPS, 2011).

Une grande partie du soutien subsidiaire consenti par l'OFSPO est répartie en interne entre la HEFSM et les centres d'entraînement nationaux. En outre, l'OFSPO propose à certaines fédérations sportives des services dans le domaine des sciences du sport, ainsi que des conseils en matière de management. Ces flux financiers, circulant à l'intérieur de l'office, ne sont pas représentés sur la figure 4.1.

Le financement des prestations fournies par les cantons et les communes est assuré par les fonds cantonaux de loterie alloués au sport et par les impôts communaux et cantonaux. Pour évaluer les dépenses des communes et des cantons en faveur du sport d'élite, on utilise la même clé de répartition que pour les subsides de loterie de Swisslos. Nous partons du principe que la répartition des bénéfices entre le sport de masse et le sport d'élite peut être transposée tout aussi bien à l'échelle du canton qu'à celle de la commune. L'estimation se base sur le total des dépenses en faveur du sport effectuées en 2009 par les cantons (232 millions de francs) et par les communes (1,346 milliard de francs). Ces montants incluent la promotion et l'encouragement d'activités, manifestations et installations sportives (sans prendre en compte les installations dépendant des instituts de formation) (Office fédéral de la statistique [OFS]). Le sport d'élite et la relève perçoivent donc 42 millions de francs de la part des cantons et 242 millions de celle des communes, comme le montre la figure 4.1.

Les bénéficiaires des financements
Les fédérations nationales et les organisateurs d'événements sportifs (ligues et organisateurs STS) sont à la fois bénéficiaires et organes de pilotage. Plus les ressources financières de l'allocataire sont importantes, plus l'indépendance que lui confèrent ses revenus pour influencer le monde du sport d'élite est grande.

En 2010, les revenus de toutes les fédérations confondues s'élevaient à env. 221 millions de francs (Lamprecht *et al.*, 2011), dont 95 à 117 millions ont servi à financer le sport de haut niveau (calcul personnel d'après les données de Lamprecht *et al.*, 2011). Les fédérations doivent arbitrer entre les intérêts du sport d'élite et ceux du sport de masse. Seule une répartition approximative est possible (Association suisse de football [ASF], 2011; Swiss Curling, 2011; Swiss-Ski, 2011; Swiss Ice Hockey Association [SIHA], 2010; Swiss Athletics, 2011; Swiss Tennis, 2011; Fédération suisse de gymnastique, 2011; Fédération suisse des sports équestres, 2011; KPMG, 2010). L'analyse des comptes annuels pour 2011 de l'ASF, de Swiss-Ski, ainsi que de la SIHA et de ses deux ligues professionnelles montre que ces structures ont reçu 17 fois plus de financements de la part de sponsors que Swiss Athletics, Swiss Tennis et la Fédération suisse de gymnastique.

Les clubs constituent la base du sport d'élite suisse. Ensemble, ils disposent de revenus de 1,1 milliard de francs, dont 400 à 730 millions – estime-t-on – sont à la disposition des clubs axés sur le sport de haut niveau (statistiques établies par les auteurs d'après les données de Lamprecht *et al.*, 2011; Rütter *et al.*, 2011). Comme on peut le voir sur la figure 4.1, 103 millions au moins proviennent des sponsors et des médias.

Les recettes des organisateurs d'événements sportifs suisses (ligues professionnelles et manifestations sportives en 2010) présentées dans le diagramme (voir la figure 4.1) atteignent au moins 100 millions de francs, provenant pour une bonne part (min. 89 millions) des sponsors et des médias. Traditionnellement, les deux ligues professionnelles du football (Swiss Football League) et du hockey sur glace (National League) bénéficient massivement du sponsoring, de la couverture médiatique et de la publicité. Les fonds récoltés servent à rémunérer les clubs participants et les fédérations nationales. Chaque année, l'ASF perçoit ainsi 16,7 millions de francs grâce au marketing et aux droits d'auteur versés par la télévision (ASF, 2011). De même, en 2011, le sponsoring et les médias ont rapporté 15,9 millions de francs à la Ligue nationale suisse de hockey sur glace (Ligue nationale suisse de hockey sur glace, 2011). Ces deux structures profitent en outre directement des fonds distribués par les sociétés de loterie nationales, comme on peut le voir sur la figure 4.1.

Les organisateurs de manifestations sportives figurent également parmi les grands partenaires financiers du sport d'élite helvétique. Ainsi, en 2010, le budget consacré à l'ensemble des événements organisés par l'association STS atteignait à lui seul 100 millions de francs (STS, 2011). Les gains issus du sponsoring et de la couverture médiatique s'élevaient à 56,2 millions de francs (calcul établi par les auteurs), soit plus de la moitié du budget.

Le revenu brut des entraîneurs suisses a été estimé sur la base de l'enquête auprès de 464 entraîneurs. La médiane selon le taux d'occupation (plein temps ou temps partiel) est extrapolée aux 800 entraîneurs dans le domaine du sport d'élite. On estime ainsi qu'en 2010, ils ont gagné à eux tous au minimum 28 millions de francs (calcul établi par les auteurs).

Le revenu brut des athlètes a été estimé sur la base d'une enquête auprès de 820 athlètes et de la médiane résultant de ces informations, après correction du taux d'occupation (activité sportive à plein temps, employé/étudiant à temps partiel, employé/étudiant à plein temps) pour 1500 athlètes d'élite suisses. Ensemble, ils ont gagné au moins 58 millions de francs (calcul établi par les auteurs).

Hiérarchie des sports selon Swiss Olympic et l'OFSPO
Pour améliorer les chances de succès, on peut choisir d'augmenter les financements. Mais il est également possible de concentrer les moyens sur les disciplines sportives les plus prometteuses en termes de médailles. Cette stratégie mérite qu'on s'y arrête.

Swiss Olympic établit un classement des fédérations en hiérarchisant les sports en catégories numérotées de 1 à 5 (Swiss Olympic, 2011b). C'est sur cette base qu'elle soutient les fédérations, sous forme de subventions annuelles destinées aux fédérations ou au sport d'élite. Ce classement influe également sur d'autres prestations. Les mesures d'encouragement spécifiques mises en œuvre en vue des Jeux Olympiques sont quant à elles prises en accord avec les fédérations.

Figure 4.2: Concentration des financements alloués au sport d'élite et à la relève par Swiss Olympic et l'OFSPO, en millions de CHF, 2010/11

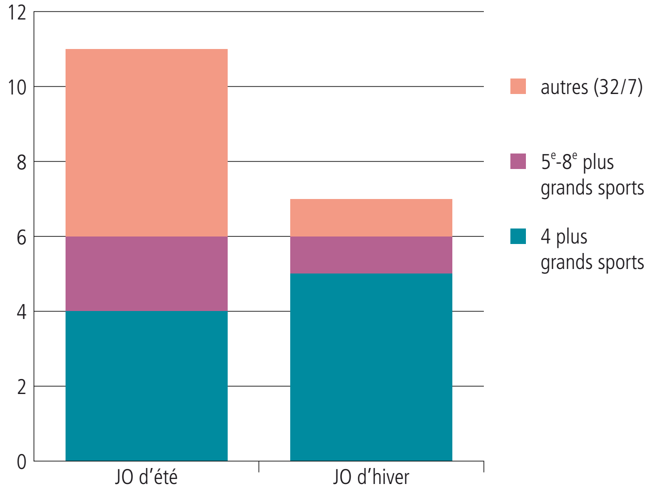

Source: figure établie par les auteurs d'après les données de Swiss Olympic (2011c) et de l'OFSPO (2012)

Swiss Olympic (sans la contribution de base de la Confédération) et l'OFSPO financent directement les disciplines sportives à hauteur de 20,3 millions de francs. Cette somme est inégalement répartie entre les sports olympiques d'été, qui en touchent 54 %, et ceux d'hiver (33 %); les 13 % restants reviennent aux disciplines non olympiques. Contrairement à d'autres nations, la Suisse ne privilégie ni les sports d'été ni ceux d'hiver.

La figure 4.2 modélise la diversification opérée par Swiss Olympic et par l'OFSPO dans le domaine de l'encouragement du sport d'élite et de la relève au sein des disciplines inscrites au programme des JO d'été et d'hiver. Le graphique est sans équivoque: la priorisation des sports est nettement plus forte en hiver (concentration sur 15 sports) qu'en été (40 sports).

En hiver, 70 % des subventions accordées servent à financer quatre sports – ski alpin, hockey sur glace, snowboard et ski de fond –, le ski alpin touchant à lui seul 33 % des subsides. En été, les quatre disciplines que sont la gymnastique artistique, la natation, le tennis et l'athlétisme concentrent 36 % des aides financières. Ces disciplines peuvent donc être qualifiées de «grandes» par rapport au soutien financier accordé. On constate les mêmes écarts lorsque l'on étend l'analyse aux huit plus grands sports, qui se partagent 91 % des financements en hiver et 57 % en été.

Il faut en outre tenir compte du fait qu'en 2010, le football a reçu de la SST 4,4 millions de francs supplémentaires pour le financement du sport d'élite et de masse. Cette même SST a également soutenu le hockey sur glace en subventionnant son sport d'élite à hauteur de 2,2 millions de francs (Société du Sport-Toto, 2012). L'aide versée annuellement par les sociétés de loterie nationales n'est pas prise en compte dans la figure 4.2.

Concentration ou diversification?

Les structures fédéralistes de la Suisse se reflètent également dans le soutien financier accordé au sport. Le système a connu une croissance organique et se caractérise par des structures d'encouragement subsidiaires, ainsi que par une autonomie du sport basée sur le droit privé. La diversité et l'étendue des sports encouragés par rapport aux athlètes et aux entraîneurs qui en bénéficient *in fine* sont typiques de ce système et lui assurent un ancrage solide. Un certain «commitment» garantit la diversité des aides et permet une large activation des moyens financiers au sein du système d'encouragement. Parallèlement, les chances de voir la population approuver les points soumis au vote concernant la politique du sport augmentent. L'encouragement du sport bénéficie ainsi d'un soutien global plus large.

Vaut-il mieux n'encourager qu'un petit nombre de sports ou privilégier au contraire le sport de masse? Les stratégies divergent d'un pays à l'autre. Certains, comme l'Australie, optent pour une forte concentration et obtiennent de très beaux résultats. D'autres, à l'instar de la France, soutiennent de nombreux sports de façon tout aussi heureuse. Les quatre disciplines olympiques les mieux loties se partagent ainsi 22 % de toutes les aides nationales destinées à la promotion du sport d'élite en France, 36 % au Japon, 38 % en Finlande, 42 % au Canada, 44 % au Danemark, 47 % en Australie et 33 % en Suisse. La route vers le succès n'est donc pas une voie unique (De Bosscher et al., 2013b).

La question est de savoir si le soutien financier doit bénéficier avant tout au sport de haut niveau ou s'il est également destiné au sport de masse – et le cas échéant, dans quelle mesure. Le sport d'élite a en effet besoin d'une base large, et vice versa. Les deux fonctionnent en symbiose. Un financement axé essentiellement sur l'un ou l'autre se révèle inefficace à court ou à long terme. Le financement privé fourni par les sponsors et la couverture médiatique bénéficie à une élite restreinte. Les bailleurs de fonds privés hésitent à financer les jeunes espoirs, si prometteurs soient-ils, durant les longues années qui les séparent de leur première victoire internationale. Ils préfèrent miser sur des athlètes de niveau mondial ayant fait leurs preuves, des valeurs beaucoup plus sûres, puisqu'un tel investissement s'avère tout de suite rentable. C'est pour se protéger de cette défaillance du marché que le sport a besoin du soutien subsidiaire que peuvent lui accorder les pouvoirs publics.

Le système du sport suisse répartit les moyens selon des mesures d'encouragement prises en vue des Jeux Olympiques, en s'appuyant sur des concepts de promotion de la relève. Ces moyens financiers permettent d'exploiter de nouveaux potentiels de médailles. La classification des disciplines sportives et la priorisation des subventions s'appuient largement sur les succès du passé, rendant la manne des aides plus difficilement accessible aux nouveaux sports ou aux disciplines à fort potentiel.

La question de la stratégie à adopter est elle aussi fondamentale: est-il efficace d'encourager un grand nombre de disciplines différentes ou vaut-il mieux se concentrer exclusivement sur un petit nombre de sports? La supériorité d'une approche dirigiste «top-down» (de haut en bas), telle qu'en pratiquent par exemple les nations préparant des JO à domicile, est de courte durée. A long terme, une saine émulation au sein du système sportif se révèle aussi bénéfique que celle qui règne entre les différents niveaux hiérarchiques. Elle agit en effet comme un stimulant, favorise la transparence et encourage un contrôle mutuel. Si, au premier abord, l'approche «bottom-up» (de bas en haut) semble présenter des doublons, la hiérarchie de structures ainsi organisées se montre plus performante sur le long terme. Cet état de fait s'est d'ailleurs déjà vérifié par le passé dans d'autres secteurs de l'économie. Les domaines financés par des entreprises privées surpassent les secteurs soumis à une gestion étatique centralisée.

5. Détection et promotion des talents

Pour un sportif, une médaille est le rêve d’une vie. Le chemin qui mène au podium olympique dure en effet de longues années. Dans nombre de disciplines, il est normal de voir un athlète s’entraîner dix ans pour atteindre ce but. Par conséquent, le moment où les athlètes commencent à s’intéresser au sport d’élite et sont orientés vers un entraînement de haut niveau, l’enthousiasme avec lequel ils s’y consacrent, constituent autant de facteurs déterminants pour toute nation souhaitant remplir son objectif de médailles. Il est donc important d’étudier de plus près la détection des talents et les méthodes assurant leur promotion.

Figure 5.1: Age du début de la pratique et de la concentration sur un sport, selon les réponses des athlètes

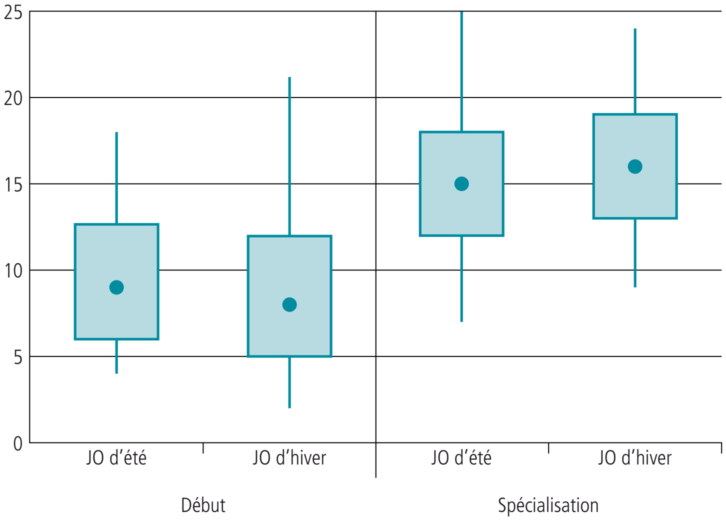

Commentaire: Point = médiane (50 % des valeurs supérieures, 50 % des valeurs inférieures), rectangle = 1er et 3e quartiles (50 % des valeurs à l’intérieur du rectangle, 25 % supérieures, 25 % inférieures), point le plus élevé = 95e centile (5 % de valeurs supérieures), point le plus bas = 5e centile (5 % de valeurs inférieures).

Source: SPLISS-CH 2011, n = 411/297 athlètes

Comment les athlètes jugent rétrospectivement leur entrée dans la carrière sportive

Il peut être intéressant d’aborder le sujet en exposant les réponses données par des sportifs encore en activité interrogés sur l’environnement qui les a orientés vers le sport d’élite. L’échantillon d’athlètes (ainsi que les entraîneurs et les chefs du sport d’élite cités plus bas) sera présenté en détail dans le chapitre 12.

L’enquête SPLISS-CH-2011 a permis de collecter des informations sur l’âge auquel les athlètes ont débuté leur sport et celui auquel ils se sont spécialisés. Les résultats sont repris dans la figure 5.1. Les athlètes pratiquant un sport d’hiver commencent en moyenne un an plus tôt que ceux pratiquant des sports d’été. La concentration sur un seul sport s’opère en revanche un tout petit peu plus tôt pour les sports d’été. Quelle que soit la saison, l’âge varie fortement au sein de chaque catégorie, avant tout en fonction de la discipline et du sexe.

Orientation vers le sport d’élite

Les athlètes ont ensuite été interrogés sur leur décision de se lancer dans le sport d’élite. Leurs réponses montrent que leur entourage proche (famille pour 57 % d’entre eux, amis pour 35 %) a joué un rôle capital dans le choix de la discipline sportive.

Les trois quarts des athlètes interrogés expliquent avoir été motivés par d’autres sportifs de haut niveau. Ceux-ci les ont confortés dans le choix de leur spécialité (39 %), incités à s’entraîner plus (84 %) ou encouragés à se soumettre à un entraînement plus intensif afin de devenir eux-mêmes des sportifs de premier plan (92 %). On peut donc en conclure que l’exemple des grands champions incite moins à commencer à pratiquer une discipline qu’à s’entraîner plus fréquemment et plus intensément. Il est toutefois intéressant de constater que les entraîneurs ayant participé à l’enquête reconnaissent aux vedettes une influence bien supérieure sur la décision de commencer un sport. Il est possible que, concernant le choix de la discipline, le rôle des stars soit surestimé.

Près des deux tiers des athlètes interrogés (62 %) ont cité leur modèle. Hormis Roger Federer et Didier Cuche, les deux champions les plus souvent mentionnés (respectivement 8 % et 2 % des athlètes), les noms donnés sont extrêmement divers (au total, 357 noms pour 555 réponses). De façon assez remarquable, les célébrités nationales n’ont été que rarement évoquées. Les modèles des sportifs de la relève se trouvent souvent dans leur entourage direct.

Les personnes de référence des sportifs de la relève et de l'élite
La figure 5.2 étudie l'influence que certaines personnes et organisations exercent sur les sportifs de la relève et de l'élite. Les individus influençant fortement et positivement les athlètes sont souvent issus de leur entourage direct: parents (88 %), entraîneurs personnels (78 %), entraîneurs de la relève (57 %) et entraîneurs de cadres nationaux (55 %). Par ailleurs, 38 % des athlètes déclarent être fortement et positivement influencés par les sponsors/bailleurs de fonds.

Figure 5.2: Influence exercée par certaines personnes et organisations sur les sportifs de la relève et de l'élite, selon les réponses des athlètes

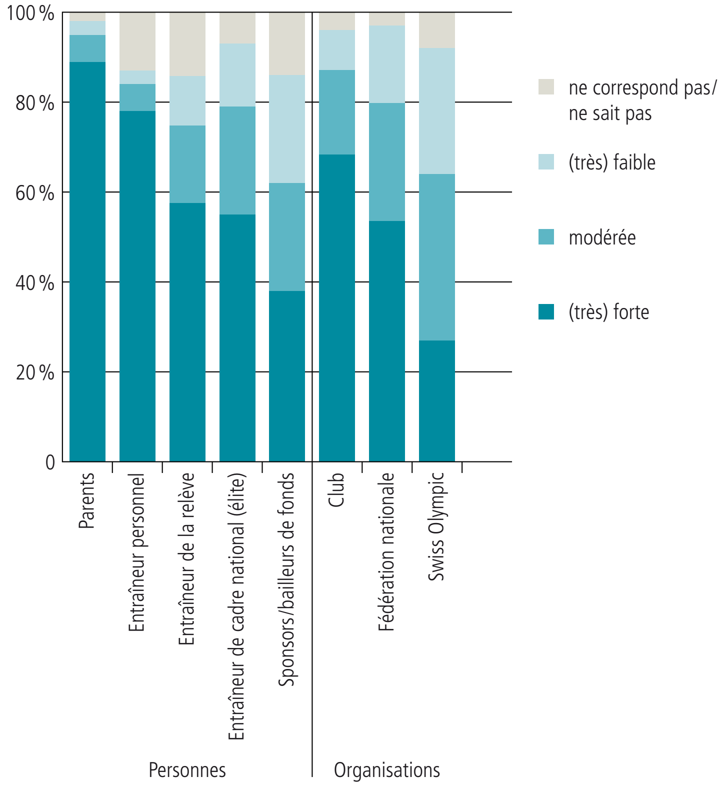

Source: SPLISS-CH 2011, n = 857 athlètes

Du côté des organisations, le club (69 %) et la fédération nationale (53 %) constituent les structures les plus présentes. Swiss Olympic (27 %) et la Fondation de l'aide sportive suisse (22 %) jouent un rôle plus limité. On note ici l'absence d'un organe national qui apporterait aux athlètes un soutien direct et solide. Les parents, les entraîneurs personnels et les clubs restent ainsi les principaux points de repère des sportifs de la relève et de l'élite helvétiques.

Les jeunes espoirs jugent plus sévèrement le soutien qu'ils ont reçu de leur fédération («bon/suffisant» pour 50 % d'entre eux, «insuffisant/mauvais» pour 24 %) que celui dont ils ont bénéficié dans leur club («bon/suffisant» pour 65 %, «insuffisant/mauvais» pour 14 %), ce qui classe ces deux structures très loin derrière les autres soutiens (en particulier les parents: «bon/suffisant» pour 65 %, «insuffisant/mauvais» pour 9 %).

Quand soutenir les jeunes talents?
L'enquête SPLISS-CH-2011 menée auprès des entraîneurs fournit un certain nombre d'informations sur l'âge auquel les athlètes de la relève bénéficient pour la première fois d'un soutien et d'un encadrement spécifiques dans leur sport. Les paragraphes qui vont suivre s'intéressent à la structure organisationnelle au sein des différentes disciplines sportives.

Comparativement aux autres nations, la Suisse dispose d'un des meilleurs et des plus larges systèmes de détection et de promotion des talents. Un grand nombre de ces derniers sont issus d'une détection centralisée. Dans ce domaine, la Suisse se classe parmi les pays les plus performants (De Bosscher et al., 2013c), soutenant les jeunes espoirs de nombreuses disciplines. Mais la mise en œuvre des mesures prises dans cette optique demande beaucoup d'énergie et ne peut être menée à bien sans l'engagement des parents, des clubs et de leurs bénévoles.

Sur la figure 5.3, on voit que le parcours qui mène du club à la fédération nationale n'est pas le même selon que l'athlète pratique un sport d'hiver ou d'été. Les structures sportives (club, association régionale et fédération nationale) n'entrent pas toutes au même moment dans la vie sportive des jeunes athlètes. Les clubs ont ainsi tendance à intervenir plus tôt dans le cas des disciplines hivernales; à mesure que l'on grimpe dans la hiérarchie des structures, la dispersion de l'âge de prise en charge se resserre. Cette dispersion est plus forte pour les disciplines d'été. Cette divergence est vraisemblablement due à la différence des aides accordées à chaque discipline sportive.

Les athlètes, les entraîneurs et les chefs du sport d'élite évaluent différemment le moment de la sélection des talents et de leur prise en charge par la fédération nationale. Les sportifs sont nettement moins satisfaits que les entraîneurs, eux-mêmes beaucoup plus critiques que les chefs, comme le montre la figure 5.4: 52 % des premiers considèrent que la sélection et la prise en charge ont été effectuées «au bon moment», contre 57 % pour les deuxièmes et 82 % pour les derniers. Environ 29 % des sportifs et des entraîneurs estiment que les talents de la génération montante ont bénéficié d'un soutien trop tardif, un avis que les chefs ne partagent que dans une moindre mesure (17 %). Il faut toutefois tenir compte du fait que, concernant la sélection, une partie des athlètes manquent du recul nécessaire pour formuler une évaluation objective, l'événement étant encore relativement récent. En outre, les organisations se réfèrent à l'âge de sélection actuel, qui peut être différent de celui auquel les athlètes interrogés ont été retenus en leur temps. Il semble cependant que l'âge idéal de sélection et de prise en charge ne soit pas aussi net que l'affirment volontiers les chefs du sport d'élite, qui se montrent trop optimistes sur ce point.

Détection, sélection et promotion des talents

Comment notre système sportif fonctionne-t-il? Les clubs constituent une base importante. Ils sont largement soutenus par la Confédération avec le programme J+S de l'OFSPO, destiné aux jeunes de 10 à 20 ans, et le programme «Sport des enfants J+S» (5 à 10 ans), qui permettent aux enfants et aux adolescents suisses de pratiquer une activité sportive.

Figure 5.3: Age de l'encouragement/la prise en chage des athlètes de la relève, selon les réponses des entraîneurs

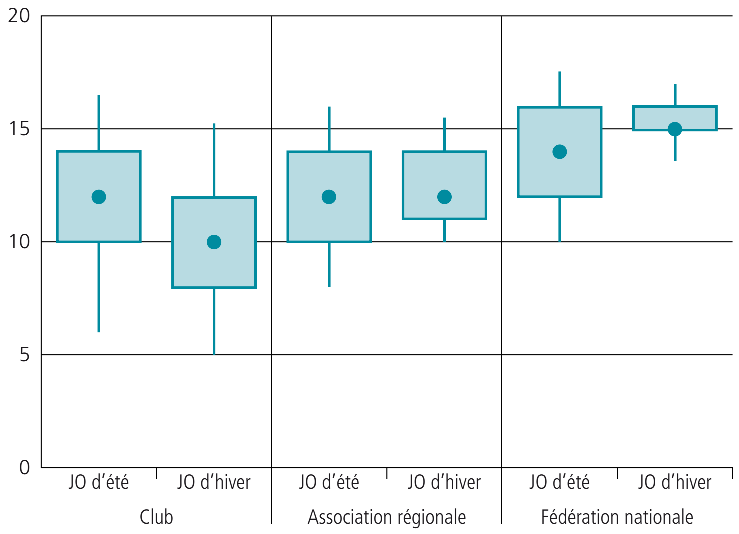

Commentaire: voir la figure 5.1
Source: SPLISS-CH 2011, n = 161-198/90-100 entraîneurs, selon la question

Figure 5.4: Evaluation du moment de la sélection et de la prise en charge spécifique des sportifs de la relève par la fédération nationale, selon les réponses des athlètes, des entraîneurs et des chefs du sport d'élite

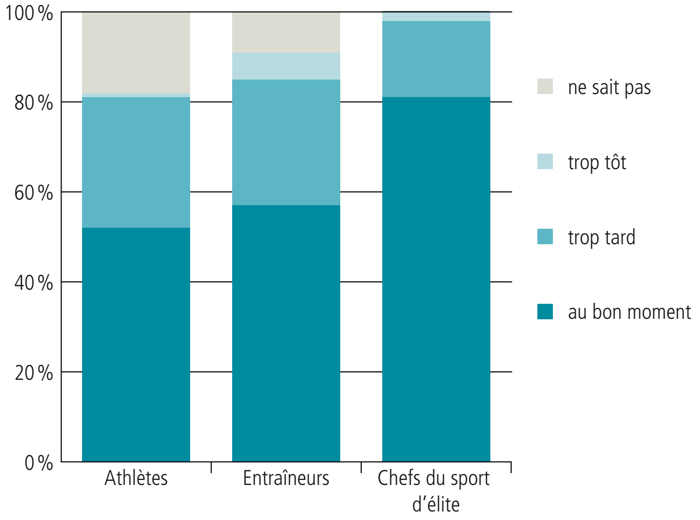

Source: SPLISS-CH 2011, n = 842 athlètes/371 entraîneurs/42 chefs du sport d'élite

Grâce à cette initiative, environ 10 000 clubs proposent chaque annné 5000 cours ou camps dans 70 disciplines. L'encadrement est assuré par 60 000 moniteurs spécialement formés. Environ 700 000 enfants et adolescents (OFSPO, non daté a) bénéficient chaque année des offres proposées par J+S. Avec l'aide subsidiaire de la Confédération, les clubs instaurent les conditions qui permettront de repérer et de sélectionner les jeunes talents. Dans certaines communes, le dispositif est renforcé par le programme «Talent Eye» de Swiss Olympic.

Le système helvétique encourage le sport selon un modèle pyramidal – du club aux Jeux Olympiques (Swiss Olympic, 2010a). Le concept intitulé «Les 12 éléments de la réussite» (Zahner et Babst, 2003) présente les fondements de ce processus de sélection. Swiss Olympic aide à la mise en œuvre du modèle d'encouragement et de sélection en distribuant les Swiss Olympic Cards (ci-après «cartes»). Conformément au modèle d'encouragement, les chefs répartissent les jeunes sportifs sélectionnés en trois niveaux: «talents locaux», «talents régionaux» et «talents nationaux». Les cartes d'«élite nationale» sont ensuite accordées sur cette base. Ce système a été introduit en 2005.

Depuis 2010, Swiss Olympic et l'OFSPO imposent aux fédérations de sélectionner leurs sportifs à l'aide du pronostic intégratif et systématique par l'estimation de l'entraîneur (PISTE), un outil mis au point par la HEFSM en collaboration avec Swiss Olympic (Swiss Olympic, 2008a). Le financement des fédérations est donc directement et indirectement fonction de la détection et de l'encouragement des talents. L'introduction du PISTE a entraîné un changement de paradigme dans le système suisse de sélection des talents, en concentrant davantage l'attention sur le potentiel des sportifs de la relève que sur leurs performances du moment.

Swiss Olympic demande aux fédérations de faire figurer l'utilisation du PISTE dans leur concept de la relève. Elles doivent mettre l'accent sur la sélection, la structure des cadres, le financement, l'école et la formation, ainsi que sur le volume d'entraînement et sur le déroulement. Les fédérations sont en partie classées et indemnisées en fonction de leur concept de la relève. En 2011, 62 disciplines sportives disposaient d'un concept reconnu par Swiss Olympic et par l'OFSPO/J+S, ce qui leur permettra d'obtenir plus de financements à l'avenir.

En 2010, Swiss Olympic recensait 8713 «talents locaux», 3013 «talents régionaux» et 2189 «talents nationaux». Les Swiss Olympic Talents Cards «Régional» et «National» sont valables un an seulement.

6. Les athlètes et le sport d'élite

Qui trouve-t-on sur les marches des podiums olympiques, sinon les athlètes? Ce sont eux qui remportent les médailles. Il est possible d'améliorer le niveau sportif d'une nation si un plus grand nombre d'athlètes accèdent au plus haut niveau ou si ces derniers consacrent plus de temps et d'énergie à leur discipline. Dans le domaine de la compétition internationale, la Suisse s'est fixé des objectifs très ambitieux. Si elle veut avoir une chance de les atteindre, il lui faut viser une professionnalisation régulière dans tous les domaines qui contribuent à la performance. La pratique professionnelle du sport constitue un thème à prendre très au sérieux.

Activité professionnelle des athlètes
Les sportifs de haut niveau suisses sont-ils tous des professionnels? Le degré de professionalisation des athlètes suisses se reflète dans leur taux d'occupation. Parmi les athlètes interrogés, à peine un tiers d'entre eux sont professionnels. Un bon tiers est constitué de sportifs à temps partiel (apprentis/étudiants ou employés/indépendants) et un petit tiers d'amateurs (étudiants à plein temps ou employés/indépendants à 100 %) qui pratiquent leur discipline durant leurs loisirs. Cette structure est la même pour les sports d'hiver que pour les sports d'été, mais change lorsque l'on s'intéresse aux disciplines non olympiques: ces dernières comptent 59 % d'athlètes amateurs et 7 % seulement de professionnels.

Les résultats de l'enquête SPLISS-CH-2011 montrent qu'un sportif s'entraîne en moyenne un peu plus de 17 h 30 par semaine, auxquelles s'ajoutent 5 h 45 de trajet. On note avec intérêt que ce dernier chiffre est pratiquement toujours compris entre cinq et six heures, que l'on parle de sportifs professionnels ou amateurs, d'une discipline d'été ou d'hiver. Dans tous les cas, les trajets empiètent sur le temps d'entraînement et de récupération. En revanche, le nombre moyen d'heures d'entraînement est plus élevé pour les disciplines d'été que pour celles d'hiver (un peu plus de 21 h pour les premières contre 16 h 15 pour les secondes). Comme on pouvait s'y attendre, les sportifs professionnels s'entraînent plus que les amateurs. Ainsi, les professionnels des disciplines olympiques d'été passent en moyenne 25 h 15 à l'entraînement, contre 16 h 45 pour les amateurs. De même, les professionnels qui pratiquent des sports olympiques d'hiver consacrent 19 h 30 à l'entraînement, contre un peu plus de 13 heures pour les amateurs.

Revenu global
Financièrement parlant, les athlètes doivent se battre pour vivre. Ils ont été interrogés sur leur revenu brut dans le cadre de l'enquête. Ce revenu global comporte la rémunération de leur activité professionnelle dans le domaine du sport et/ou dans l'économie.

Comme on le voit sur la figure 6.1, la majorité des sportifs interrogés gagnent relativement peu. En 2010, seuls 16 % des athlètes d'élite suisses ont touché un revenu total supérieur à 70 000 francs. Le diagramme montre également que la situation est plutôt meilleure pour les sportifs d'hiver que pour leurs confrères des disciplines estivales. Cela dit, même dans les sports d'hiver, 70 % des athlètes vivent avec moins de 70 000 francs et 40 % gagnent moins de 14 000 francs par an.

Figure 6.1: Revenu annuel total des athlètes en 2010

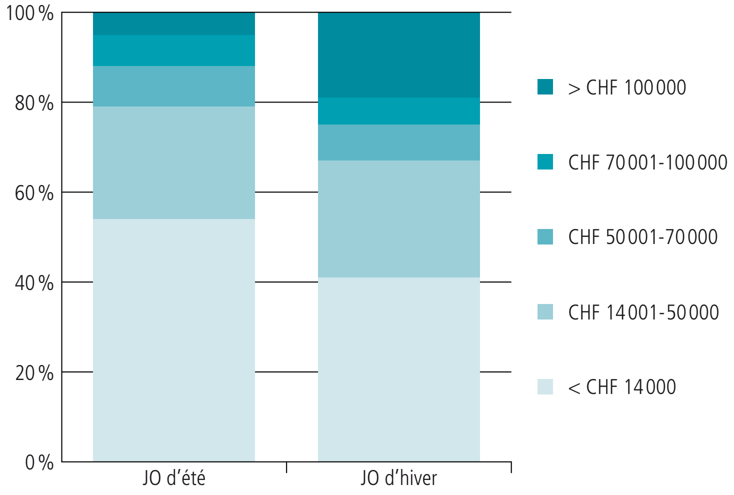

Source: n = 350/269 athlètes

Revenus tirés du sport
Concernant les revenus générés par le seul sport d'élite, les informations collectées montrent d'abord un grand écart de niveau. L'immense majorité des sportifs d'élite sont modestement rémunérés pour leur activité sportive. Le gain médian des athlètes professionnels est de 25 000 francs par an. Cela n'empêche pas de trouver des revenus annuels supérieurs à 100 000 francs dans toutes les catégories de sportifs. Mais ceux qui parviennent à faire fortune grâce au sport sont malgré tout très rares. La figure 6.2 montre les revenus que les sportifs tirent de leur spécialité sportive selon leur taux d'activité.

Figure 6.2: Revenu tiré du sport par les athlètes selon leur taux d'occupation en 2010, en CHF

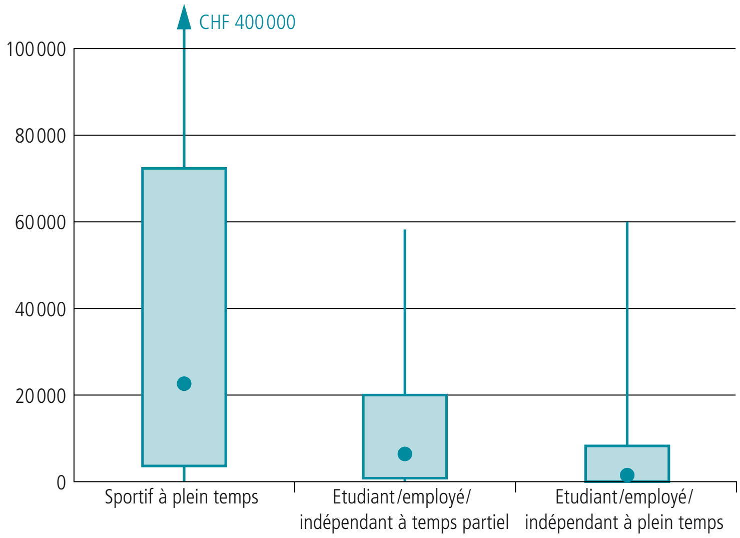

Commentaire: voir la figure 5.1; point le plus élevé de la catégorie «plein temps»: 400 000 francs (95e centile)

Source: SPLISS-CH 2011, n = 231/274/261 athlètes

Figure 6.3: Composition du revenu touché par les athlètes grâce au sport

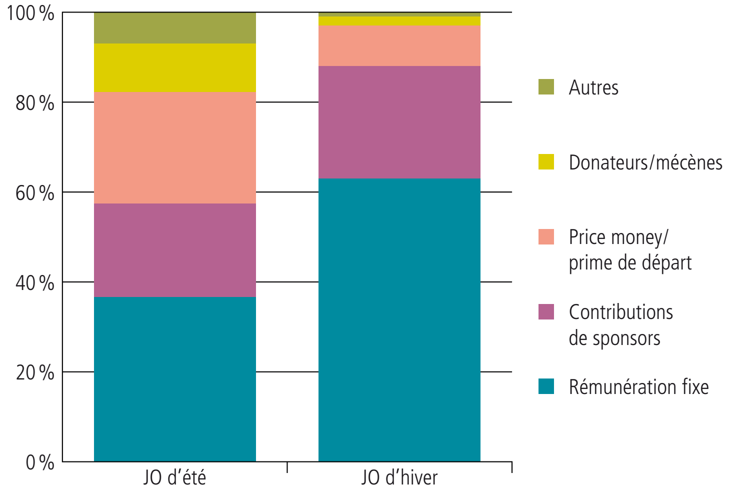

Commentaire: autres = merchandising, service militaire (allocation pour perte de gain) et autres recettes

Source: SPLISS-CH 2011, n = 369/281 athlètes

La structure des revenus générés par la pratique du sport d'élite – considérés comme la somme des revenus de toutes les catégories confondues – fait ressortir de nettes différences entre les diciplines d'été et celles d'hiver. Elles apparaissent clairement sur la figure 6.3: les «rémunérations fixes» sont proportionnellement plus élevées en hiver qu'en été. En revanche, la part de salaires variables que constituent les «price money/primes de départ» est plus forte en été. Cette différence laisse à penser que les athlètes d'hiver bénéficient d'une plus grande sécurité financière pour planifier leur carrière.

Les résultats de l'enquête montrent également que 25 % des sportifs interrogés touchent régulièrement un salaire mensuel pour leur activité sportive. Dans le domaine des sports d'équipe, les principaux employeurs sont les clubs. Ils sont plus diversifiés dans les sports individuels ou en groupe (sponsors, fédérations, etc.).

Prestations de service et soutien fournis par les organes de pilotage subsidiaires

Au moment de l'enquête SPLISS-CH 2011, 80 % des athlètes interrogés étaient titulaires d'une «Swiss Olympic Card» (ci-après «carte»). En 2010, Swiss Olympic a décerné au total 25 cartes «or», 341 cartes «argent», 214 cartes «bronze» et 173 «International Talent Card» (Swiss Olympic, 2012a). En attribuant ces cartes et en reconnaissant aux sportifs leur appartenance à l'élite, Swiss Olympic exerce une influence certaine sur les prestations fournies par l'association Swiss Olympic elle-même, mais aussi par la Confédération et les fédérations sportives. En plus des revenus qu'ils peuvent percevoir, les athlètes bénéficient de services subventionnés, qui constituent autant de dépenses en moins.

Au total, Swiss Olympic soutient 43 athlètes d'élite avec un budget d'un peu plus d'un million de francs, et 542 autres athlètes avec 1,4 million de francs par an (Swiss Olympic, 2011a). Trente athlètes reçoivent de Swiss Olympic et de la Confédération (DDPS et Corps des gardes-frontières) un soutien qui leur permet de pratiquer leur sport à titre professionnel. La Confédération encourage directement les athlètes d'élite en en employant 18 au sein du DPPS, en tant que soldats Sport à mi-temps. A l'avenir, ils toucheront chaque année une généreuse solde équivalant à 100 jours de service/allocation pour perte de gain. Enfin, douze sportifs sont intégrés à plein temps au Corps suisse des gardes-frontières.

Figure 6.4: Services fournis par les associations régionales et les fédérations nationales, selon les réponses des chefs du sport d'élite

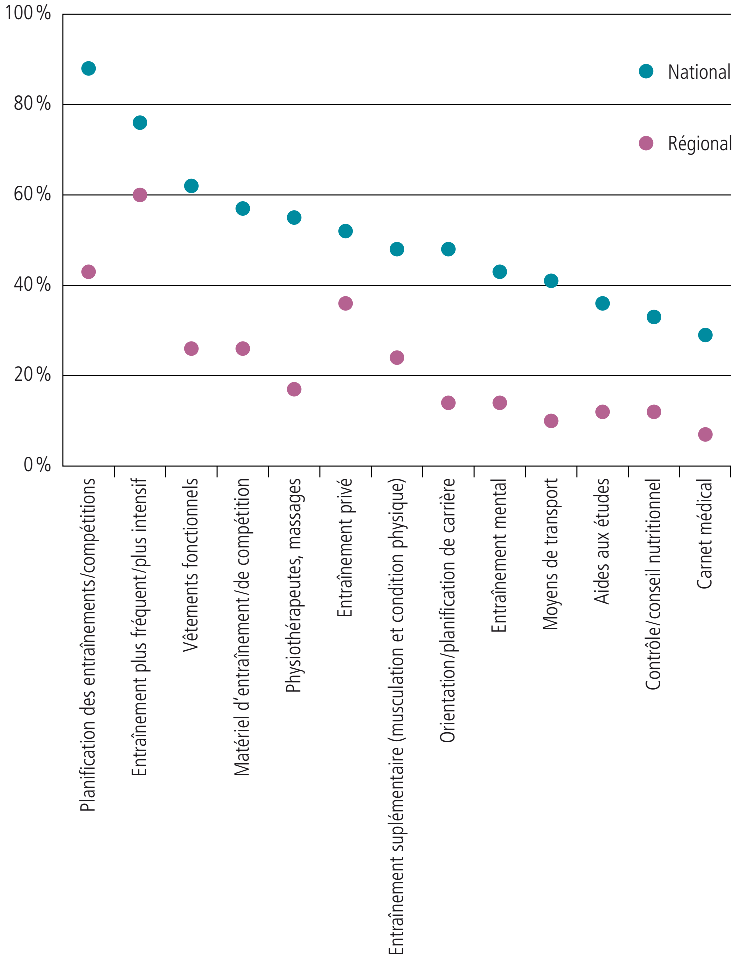

Source: SPLISS-CH 2011, n = 42 chefs du sport d'élites

Les athlètes profitent également de services et de soutiens offerts par d'autres partenaires de Swiss Olympic (orientation de carrière, etc.). Par ailleurs, la possession de cartes constitue de plus en plus souvent une «porte d'entrée» dans les structures de soutien privées comme publiques (fondations, écoles de sport, cantons, etc.). Les cartes attribuées annuellement ne sont pas optimales. Cette pratique est en contradiction avec un travail de fond mené sur le long terme, mais plus agressif en termes d'organisation de l'entraînement.

Services et mesures de soutien proposés par les fédérations et les clubs

Les services fournis par les fédérations et les clubs concernent avant tout l'entraînement. Les réponses des chefs du sport d'élite indiquent que 70 % des fédérations sondées proposent à leurs athlètes une «planification des entraînements/compétitions» ainsi qu'un «entraînement plus fréquent/plus intensif». Les associations régionales se concentrent principalement sur cette dernière offre, proposée par près de 60 % d'entre elles. Moins de 40 % des associations apportent un soutien dans le domaine des études. La figure 6.4 propose une représentation graphique de la situation, confirmée pour l'essentiel par les athlètes.

Les résultats de l'étude SPLISS-CH 2011 montrent que les divers services et mesures de soutien proposés par les fédérations nationales ne descendent pas jusqu'au niveau des associations régionales. Il est en outre possible d'apporter des améliorations pour combler les écarts constatés entre les différentes prestations proposées. De fait, chaque année qui passe sans que la qualité des encouragements dispensés ne soit à la hauteur, les athlètes perdent un temps précieux pour leur carrière post-sportive, qu'il s'agisse de leur formation ou de leur entrée sur le marché du travail.

Les entraîneurs se sont exprimés sur la «qualité» et le «volume» des prestations d'encadrement dont bénéficient les athlètes dans différents domaines au cours des douze mois précédant l'enquête. Le domaine des sciences de l'entraînement, avec le sous-domaine «musculation/endurance», affiche les résultats les plus satisfaisants. A l'inverse, le sous-domaine «biomécanique/analyse de la performance» présente le plus fort potentiel en termes d'élargissement de l'offre des prestations. De même, les entraîneurs considèrent que la «prise en charge psychique» et le «conseil nutritionnel» sont susceptibles d'amélioration. Mais c'est dans le domaine de la «gestion de la carrière» que les sondés discernent le potentiel d'amélioration le plus important.

Sport et formation

La possibilité de concilier sport et école/formation est une question importante pour le développement des sportifs de la relève. Un tiers des athlètes ont effectué les niveaux secondaires I et II dans un établissement spécialisé, adapté aux exigences du sport d'élite. Plus de la moitié des sportifs (55 %) estiment avoir été bien ou suffisamment soutenus durant le secondaire I et leurs années d'école professionnelle/de maturité (12-18 ans). La situation se complique lorsqu'ils intègrent une haute école ou une université (>18 ans). Les traitements spécifiques concernent principalement l'allègement du temps de présence obligatoire et l'aménagement des conditions d'examen ou de l'emploi du temps. La figure 6.5 propose un graphique résumant la situation.

Les athlètes ont suivi une formation moins poussée que le reste des Suisses âgés de 25 à 34 ans. Leur pratique sportive les oblige en effet souvent à allonger la durée de leurs études. Seuls 25 % d'entre eux ont achevé une formation tertiaire, contre 40 % de la population suisse âgée de 25 à 34 ans (OFS, 2012a). Lorsqu'ils suivent une formation tertiaire, celle-ci est repoussée à plus tard.

Le fait que 63 % des athlètes interrogés prévoient de suivre une formation ou une filière de perfectionnement dans les cinq années à venir met en lumière une des grandes faiblesses du système helvétique: dans la mesure où la moitié d'entre eux souhaitent intégrer une haute école ou une université, il serait temps de progresser à ce niveau-là également. On pourrait envisager de prendre en compte les prestations de formation sportive dans ce contexte. Par exemple, si un athlète développe lui-même son ski et son matériel avec des partenaires académiques, cela devrait être reconnu par l'institution de formation. La flexibilité dans les études constitue une exigence minimale dans les milieux sportifs.

Dans quelle mesure faut-il viser une formation parallèle ou sérielle? La réponse dépend des objectifs sportifs. Les victoires sur le plan international ne laissent que peu de place aux compromis.

Figure 6.5: Soutien reçu pour concilier sport d'élite et formation, selon les réponses des athlètes

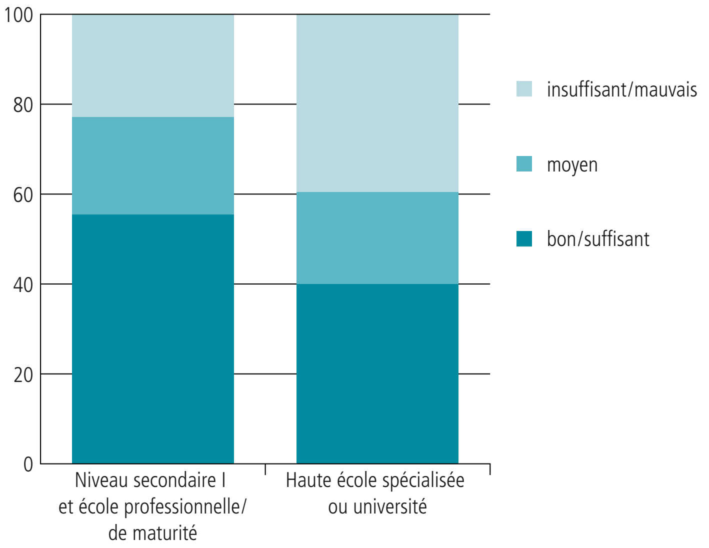

Source: SPLISS-CH 2011, n = 736/445 athlètes

Carrière post-sportive

Le sport constitue une option séduisante pour les athlètes, qui, en cas de succès, peuvent espèrer en retirer des gains financiers et une élévation de leur statut social. L'étude montre que 91% d'entre eux pensent trouver un emploi rapidement après la fin de leur carrière sportive. Ce chiffre peut s'expliquer par le fait que 30 % des athlètes doivent concilier sport et travail, ce qui n'est possible qu'avec le soutien de l'employeur: pour 80 % des athlètes exerçant un emploi parallèlement au sport d'élite, ce soutien est jugé «bon» à «suffisant». On peut supposer qu'ils s'attendent à trouver un autre emploi une fois qu'ils auront achevé leur carrière sportive. Leur confiance est également due à la situation du marché du travail en Suisse, caractérisé par un taux de chômage très bas.

La formation garantit une certaine sécurité. Les chances de décrocher un poste sont en effet plus élevées lorsque le candidat a reçu la formation appropriée. Or, les résultats de l'enquête révèlent que seul un quart des athlètes pensent que le soutien à la reconversion est de bonne qualité en Suisse. Il y a fort à parier que ce quart rassemble les athlètes ayant bénéficié de mesures d'encouragement, parmi lesquelles la constitution du réseau professionnel (33 %), des programmes d'études pour suivre une formation ou un perfectionement (23 %) et un accompagnement pour les aider à gérer leur carrière (22 %). Ces prestations sont censées leur faciliter le retour au quotidien après la fin de leur parcours dans le sport d'élite.

Le manque à gagner subi par les athlètes poursuivant une carrière sportive ne forment qu'un aspect du problème. L'expérience professionnelle qu'ils n'ont pas acquise, la formation qu'ils n'ont pas suivie, ont des répercussions beaucoup plus importantes. Privilégiant l'entraînement, le sportif disposera d'un niveau de formation moindre et plus tard, dans le milieu professionnel, d'un bagage moins important: autant de handicaps qui réduiront ses gains. Ces coûts (d'opportunité) pèsent en partie plus lourd que ceux induits par l'obligation de renoncer à certains revenus et à ses loisirs tout au long de la carrière sportive. L'athlète s'en rendra compte au plus tard lorsqu'il commencera à réfléchir à l'avoir déposé dans sa caisse de pension.

La professionaisation du sport d'élite en Suisse ne se fera pas sans une meilleure reconnaissance du métier d'athlète de haut niveau sur le marché du travail. Si les athlètes bénéficient d'un suivi dans les cadres en qualité d'entreprise individuelle ou sont même embauchés dans le club, le développement et la reconnaissance des compétences doivent être pilotés en connaissance de cause. Il importe également de garder à l'esprit que les systèmes d'encouragement ciblent prioritairement des types d'athlètes différents, dotés de leur personnalité propre. Selon la structure et les connaissances spécifiques à la discipline, l'on développera différentes compétences, qu'il convient de démontrer. Cette qualification aidera les sportifs dans leur future vie professionnelle. L'expérience amassée dans le domaine du sport d'élite doit être valorisée dans le cadre de la carrière post-sportive.

Les Pays-Bas apportent leur soutien à 352 athlètes en leur versant 120 % du revenu minimal légal. Des accords spéciaux peuvent également être signés avec les hautes écoles afin de pouvoir mieux concilier sport et études (Bake, 2013a). La Finlande et le Danemark misent sur un système très développé de bourses pour les étudiants. La Finlande accorde des aides forfaitaires calculées en fonction du niveau de l'athlète (p. ex. 20 600 francs pour un athlète d'élite en 2013). Au Danemark, le montant est négocié individuellement et adapté aux besoins du bénéficiaire (Tofft-Jørgensen, 2013). D'autres pays garantissent directement à leurs athlètes une sécurité financière et professionnelle en les intégrant dans l'armée, la police des frontières, la police ou le corps de pompiers. La Suisse est plutôt mal classée dans le palmarès international des pays apportant un soutien financier direct à leurs athlètes. Le constat vaut également pour les heures d'entraînement déclarées, les athlètes helvétiques s'entraînant relativement peu (De Bosscher et al., 2013d).

7. Entraîneurs, managers et dirigeants du sport

Un entraînement intensif et une bonne préparation à la compétition sont à la base du succès olympique. Les entraîneurs garantissent la qualité de l'entraînement quotidien et mettent les athlètes dans les meilleures conditions pour être performants. Les managers sportifs s'assurent en amont que toutes les conditions-cadres sont réunies pour le bon déroulement de la compétition. Quant aux dirigeants du sport, ils veillent à ce que l'ensemble du système sportif reste compétitif sur le plan international: les victoires ne doivent pas être le fruit du hasard, ni dépendre de quelques athlètes d'exception.

Les compétences de l'entraîneur

Les entraîneurs occupent une fonction-clé dans le sport de haut niveau. Ils influent directement sur les ajustements à apporter durant les phases sensibles de l'entraînement. Ils fixent les priorités et donnent les impulsions nécessaires avant et après la compétition.

Interrogés sur le travail des entraîneurs, les athlètes ont répondu de manière très positive, y compris lorsqu'ils évaluaient l'encadrement de leurs premières années. Pour 81 % des sondés, le niveau des connaissances spécifiques des entraîneurs de la relève est «bon/suffisant». Les entraîneurs de club obtiennent un résultat légèrement inférieur (75 %). Quant aux connaissances des entraîneurs personnels de la relève, elles sont jugées bonnes ou suffisantes par 89 % des sportifs interrogés.

Leur évaluation est plus nuancée lorsqu'ils parlent de leurs années au sein de l'élite. La figure 7.1 montre que dans les trois domaines étudiés, plus de 70 % des athlètes considèrent que leurs entraîneurs disposent de compétences «élevées» à «très élevées». Ils estiment avant tout leurs compétences spécifiques, suivies de celles relevant du domaine technique. En revanche, ils ont tendance à juger plus sévèrement les qualités humaines de leurs coachs.

Figure 7.1: Compétences et aptitudes des entraîneurs évaluées par les athlètes

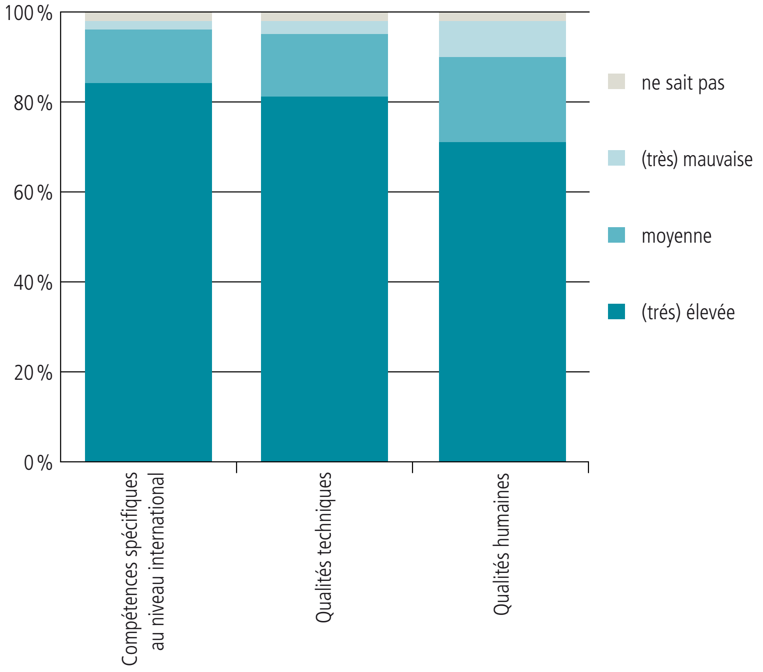

Source: SPLISS-CH 2011, n = 800 athlètes

Figure 7.2: Situation professionnelle des entraîneurs en Suisse, selon les chefs du sport d'élite

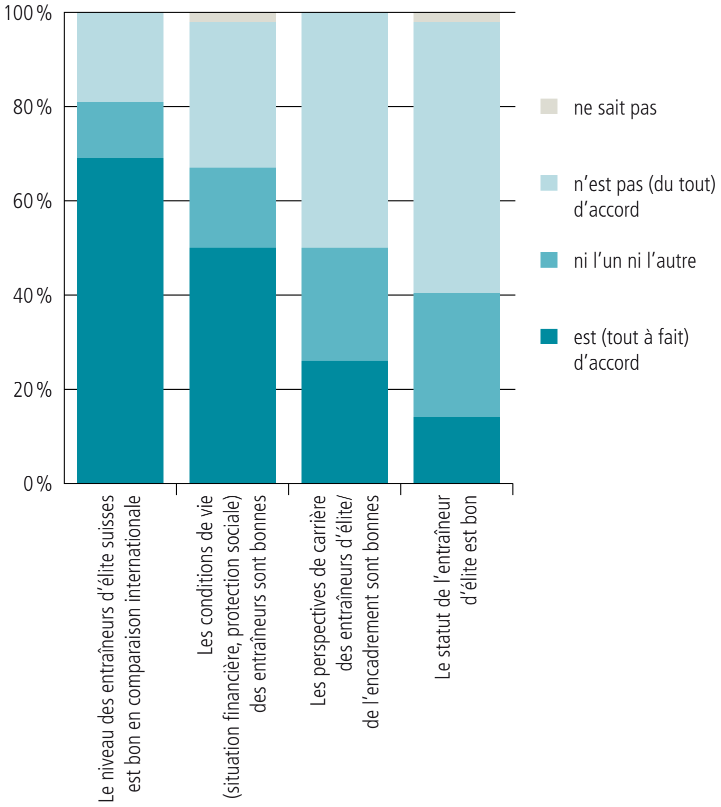

Source: SPLISS-CH 2011, n = 42 chefs du sport d'élite

Une analyse plus approfondie de ces données montre que les entraîneurs des disciplines d'hiver obtiennent des résultats légèrement inférieurs à ceux de leurs confrères d'été dans les deux premiers domaines (compétences spécifiques et techniques), mais se situent au même niveau pour les qualités humaines. Dans l'ensemble, les réponses reflètent une certaine confiance. C'est particulièrement vrai lorsque l'on demande aux athlètes si leur entraîneur du moment est le plus apte à les suivre dans la phase actuelle de leur carrière de sportif d'élite: 72 % des sondés sont «d'accord» ou «tout à fait d'accord» avec cette affirmation. Et ils sont encore plus nombreux (77 %) à déclarer qu'ils peuvent encore beaucoup apprendre de leur entraîneur actuel. De l'avis des athlètes, le travail effectué par les entraîneurs est donc tout à fait précieux.

Le métier d'entraîneur

En 2010, le nombre d'entraîneurs occupant ce poste à plein temps ou à temps partiel en Suisse était estimé à 800. Bien que ce chiffre soit considérable, les résultats de l'enquête SPLISS-CH-2011 montrent que plus de 80 % des chefs du sport d'élite estiment qu'il est insuffisant.

En moyenne, un entraîneur reste en exercice entre neuf (formation de la relève) et dix à onze ans (élite). La durée médiane est plus courte (respectivement six et huit ans). Autrement dit, la moyenne est relevée par la grande fidélité d'une partie des entraîneurs envers leur profession.

Plus de la moitié des entraîneurs interrogés estiment que les possibilités d'évolution offertes par leur travail sont «bonnes», voire «très bonnes». Parallèlement, 50 % d'entre eux considèrent qu'ils sont «insuffisamment» considérés en leur qualité d'entraîneur. De même, 40 % ne partagent pas ou pas du tout l'avis selon lequel les conditions de vie (situation financière, protection sociale) des entraîneurs sont bonnes. Il faut donc en conclure qu'en Suisse, la considération sociale attachée à cette profession et les perspectives de carrière qui lui sont associées sont plutôt limitées.

Parmi les chefs du sport d'élite, 70 % jugent que le niveau des entraîneurs d'élite est bon par rapport à celui des autres pays. De même, 50 % des chefs jugent les conditions de vie des entraîneurs satisfaisantes au regard de la situation internationale. Les chiffres relatifs à la reconnaissance de la profession et aux opportunités de carrière, présentés sur la figure 7.2, donnent à réfléchir. Ils sont même préoccupants si l'on songe que les chefs du sport d'élite sont tout de même les employeurs des entraîneurs.

Figure 7.3: Revenu global annuel des entraîneurs suisses en 2010

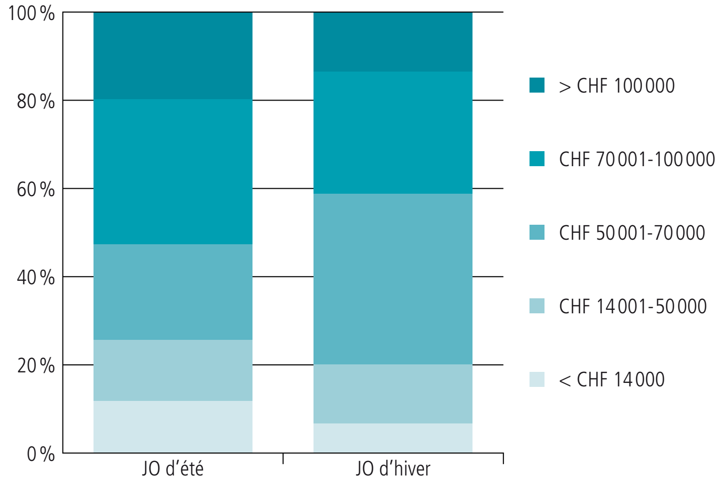

Source: SPLISS-CH 2011, n = 203/119 entraîneurs

Revenu global et revenu tiré du sport

Le revenu global d'un entraîneur, comprenant la rémunération de ses activités (sportives et/ou autres), est relativement modeste. L'étude a à chaque fois pris en compte le revenu brut. Plus de la moitié des entraîneurs ont indiqué un revenu inférieur à 70 000 francs par an (voir la figure 7.3). La situation est plus difficile dans les disciplines olympiques d'hiver que dans celles d'été. A titre de comparaison, on peut faire remarquer qu'en Suisse, en 2011, seuls les employés des secteurs des services et de la vente et les travailleurs auxiliaires touchaient un salaire inférieur (OFS, 2013).

Le profil et la structure des revenus des entraîneurs se caractérisent par leur variété. Un peu moins de la moitié des entraîneurs (47 %) occupent cette fonction à plein temps, les autres l'exerçant à temps partiel. Pour environ 20 % des entraîneurs, le sport représente moins de 30 % de leur temps de travail. Les résultats de l'enquête SPLISS-CH-2011 semblent suggérer que quelques entraîneurs financent leur activité sportive avec les revenus tirés d'une activité parallèle, plus lucrative.

Le sport rapporte aux entraîneurs à plein temps un revenu médian de 60 000 francs (disciplines d'été) et 66 000 francs (disciplines d'hiver). Cette valeur est bien moindre pour les entraîneurs à temps partiel (10 000 francs par an). La rémunération des entraîneurs ne correspond ni aux exigences du sport de haut niveau ni à la durée de la formation. On constate

Figure 7.4: Revenu annuel moyen généré par les activités d'entraîneur en francs

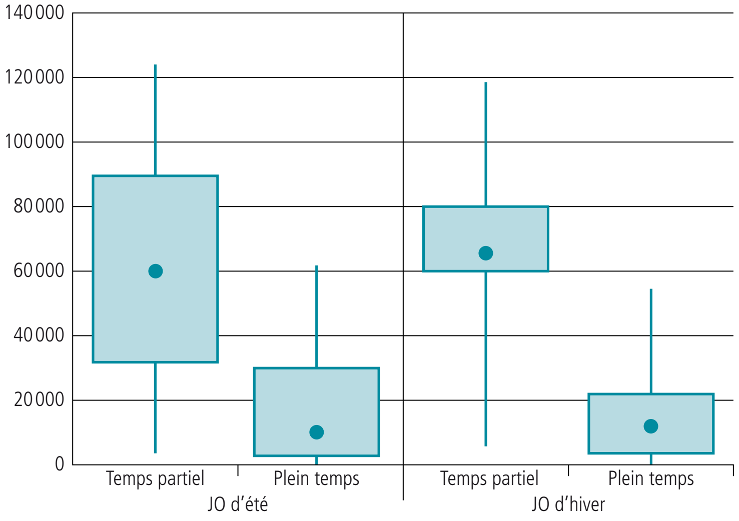

Commentaire: voir la figure 5.1
Source: SPLISS-CH 2011, n = 84/116/81/36 entraîneurs

également que les salaires sont bien plus hétérogènes chez les entraîneurs d'été que d'hiver. L'étude montre en effet que, concernant les disciplines d'été, 50 % des revenus des entraîneurs à plein temps sont compris entre 35 000 et 87 000 francs, tandis qu'en hiver, ces revenus oscillent entre 60 000 et 80 000 francs (voir la figure 7.4).

Les salaires des entraîneurs sont souvent versés par différents employeurs. Les entraîneurs interrogés travaillent principalement pour les clubs et les fédérations.

Formation

La plupart des entraîneurs interrogés dans le cadre de l'enquête SPLISS-CH-2011 disposent d'une bonne formation. Près de 80 % d'entre eux ont suivi la Formation d'entraîneur professionnel avec brevet fédéral (FEP). Environ un tiers sont titulaires de la plus haute qualification de la profession, à savoir le diplôme fédéral, délivré au terme du Cours d'entraîneur (CDE), ou un équivalent. Cette forte proportion est entre autres due au choix de l'échantillon sondé.

En Suisse, la formation des entraîneurs se base sur la structure J+S et sur les cursus proposés par les fédérations. Les entraîneurs de la relève des différents niveaux doivent satisfaire à des exigences déterminées par la Confédération et partiellement complétées par les fédérations nationales. C'est ce qui explique que la formation soit plus ou moins longue selon les fédérations. Le programme J+S prend en charge la plupart des étapes de la formation aux niveaux cantonal et fédéral.

Les parcours de formation sont conçus pour des entraîneurs ayant progressivement acquis leurs compétences en exerçant leurs premières fonctions (souvent bénévoles). Il leur faut d'abord passer par une série de courts modules très variés avant d'être admis à suivre les cours du niveau supérieur. Dans le meilleur des cas, le candidat a besoin de trois à quatre ans pour achever tous les modules des différents niveaux J+S. Si l'un d'entre eux est annulé faute de participants, ou si le candidat ne peut y assister, le cursus dure plus longtemps. Ce n'est qu'une fois franchies toutes ces étapes que le candidat est admis à suivre la FEP proprement dite, qui dure environ un an, voire plus dans certaines fédérations. Il devra compter dix-huit mois supplémentaires s'il veut obtenir le plus haut niveau de formation d'un entraîneur, le diplôme fédéral du CDE.

Il s'écoule donc au mieux quatre années entre les premiers modules du programme J+S et le début de la FEP, voire cinq ans si l'étudiant vise le CDE. La révision de la loi sur

l'encouragement du sport intervenue en 2012 a permis d'harmoniser la formation au niveau des entraîneurs de la relève J+S, allongeant en partie les cursus. Comparée à la durée effective de la formation (un peu plus d'un an), la durée de formation qui permettra au candidat d'obtenir la qualification est extrêmement longue. La figure 7.5 offre un aperçu du parcours.

En Suisse, les directives relatives à la formation prévoient l'octroi de financements. Les parcours proposés sont subventionnés, l'employeur est en partie dédommagé si l'un de ses collaborateurs décide de suivre une formation. Selon le cursus (moniteurs J+S, p. ex.), la fédération peut bénéficier d'une aide financière pour ses formations. Enfin, la qualité des prestations fournies aux athlètes par les entraîneurs doit être régulée par des directives de subventionnement claires.

Figure 7.5: Volume horaire et durée effective de la formation d'entraîneur
(durée de la formation en années)

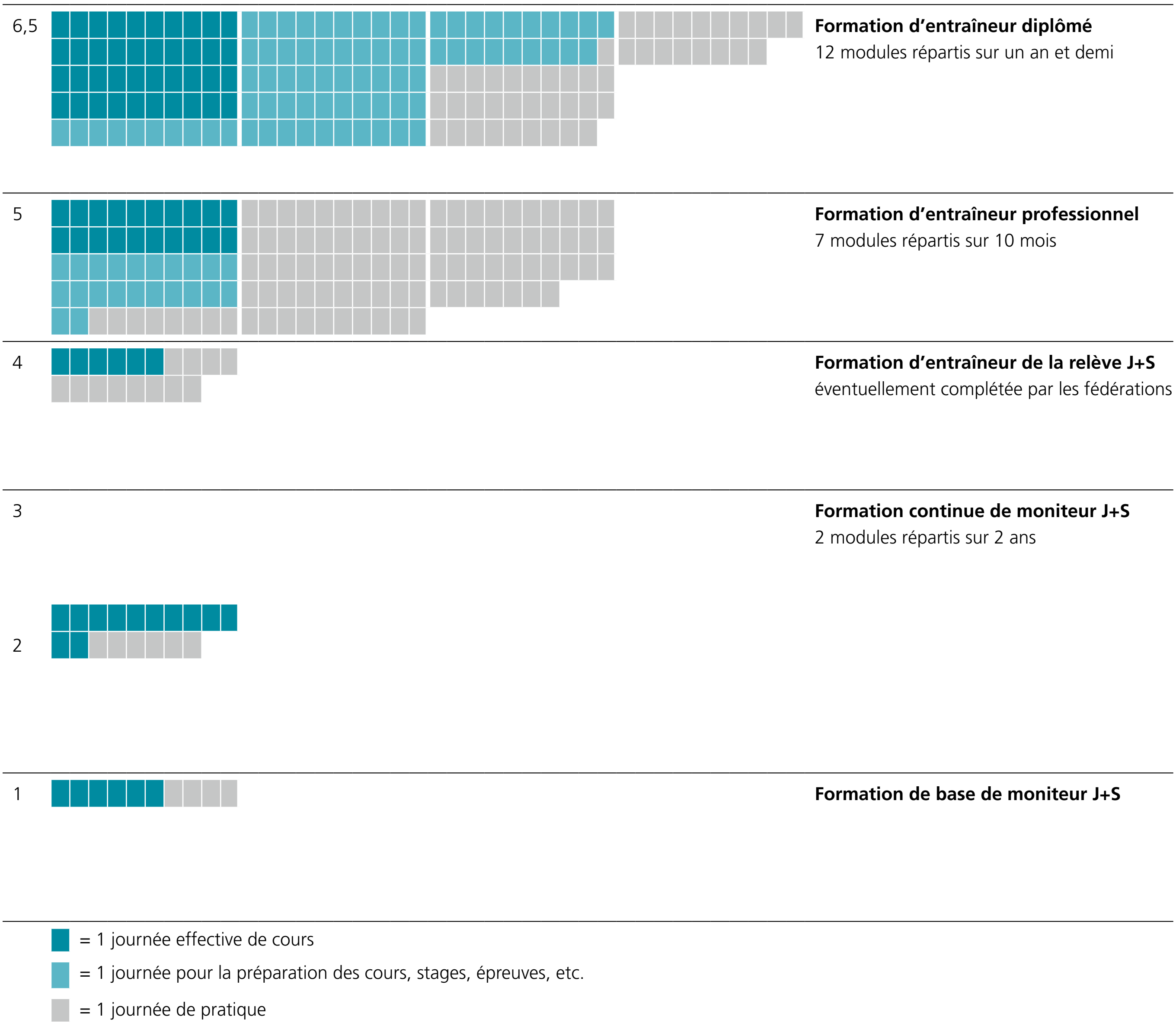

Commentaire: Les volumes horaires ont été estimés à partir des indications fournies par la Formation des entraîneurs Suisse pour une journée de travail de 8,4 heures.

Source: figure établie par les auteurs d'après les données de l'OFSPO (non daté c)

Le marché des entraîneurs en Suisse

Le marché des entraîneurs est sans doute l'un des plus réglementés en Suisse. Son accès est régulé par la Confédération (J+S), le niveau des salaires dépend des subventions et la Formation des entraîneurs Suisse détient un monopole de fait en matière de formations. Les stages obligatoires garantissent un petit bagage aux apprentis, mais assurent également un vivier de main-d'œuvre bon marché aux fédérations. Cette réglementation trouve sa justification dans des raisons d'ordre économique. Les coûts d'information et de transaction sont très élevés, et le marché des entraîneurs loin d'être parfait. Bien que petit, il demande des connaissances très spécifiques et ne suscite qu'une demande limitée pour chacune de ses disciplines. Les entraîneurs de l'élite sont chargés d'un rôle particulier, en raison de leur proximité avec les athlètes et de la présence des médias.

A y regarder de plus près, on distingue quantité de signes montrant que la logique des solutions de marché traditionnelles investit également cette sphère. Le sport a bien perçu la nécessité de tendre vers la professionnalisation, y compris à l'échelle régionale. C'est pourquoi, dans le cadre des nouveaux processus d'encouragement consenti par la Confédération en faveur de la relève du sport d'élite, les subventions sont couplées à la Formation d'entraîneur professionnel. L'époque du bénévolat semble bel et bien révolue – du moins au sein des fédérations en mesure de ne pas financer leur offre de prestations uniquement au moyen des subventions accordées par la Confédération.

La concurrence d'autres professions se fait elle aussi sentir. Comme l'ont montré les chiffres analysés plus haut, les entraîneurs sont souvent mieux rémunérés dans l'économie que dans le sport. Les Suisses qui osent se consacrer exclusivement à leur métier d'entraîneur sont peu nombreux, privant le sport d'un potentiel précieux. De la même façon, il est fort probable que certains électrons libres ne sachent pas comment pénétrer le monde du sport. Voilà pourquoi il est capital que les entraîneurs talentueux soient encouragés de façon ciblée et puissent atteindre rapidement et facilement le plus haut niveau de formation.

La concurrence internationale joue également un rôle. Pour les fédérations, il est souvent plus facile d'embaucher un entraîneur étranger ou, concernant la formation, de demander à ce que les éventuels cursus suivis à l'étranger soient pris en compte. Elles gagnent ainsi un temps précieux. Les résultats de l'enquête montrent que les entraîneurs ayant été formés à l'étranger travaillent plus souvent à plein temps (env. 70 % d'entre eux) que ceux ayant obtenu leur qualification en Suisse (env. 45 %). La moitié des entraîneurs formés à l'étranger détiennent un diplôme analogue à celui délivré à l'issue du CDE, et 18 % seulement une formation similaire à la FEP. Ces certificats obtenus à l'étranger sont complètement reconnus en Suisse. A court terme, la multiplication des compétences importées peut soulager les fédérations. Mais à long terme, l'exportation de nos entraîneurs – celle d'entraîneurs de ski alpin en Autriche, par exemple – serait une preuve de notre succès.

Si l'on compare la durée moyenne d'une carrière d'entraîneur à celle de la formation, l'on bute sur un autre problème: 51 % des entraîneurs formés en Suisse détiennent un diplôme de FEP, contre seulement 34 % pour le CDE. Il se peut tout à fait qu'un certain nombre d'entraîneurs expérimentés jettent l'éponge après être parvenus au plus haut niveau de formation possible. Voilà pourquoi il est urgent de proposer des opportunités de formation continue intéressantes aux entraîneurs. Le sport d'élite moderne dispose encore de larges potentiels d'amélioration.

Un succès durable pour le sport suisse

Le sport d'élite moderne exige des compétences variées. Le partage du travail, la différenciation et la professionnalisation de tous les niveaux constituent des facteurs de succès. Le rôle des entraîneurs est capital. Pour remporter des succès aux Jeux Olympiques, il faut bien plus que les connaissances de l'entraîneur. Les succès olympiques sont le fruit d'une alchimie complexe dans laquelle interviennent des éléments extrêmement divers. Le matériel, la santé, la motivation déterminent tout autant le succès que l'entraînement et la forme du jour. Les sciences de l'entraînement, ou encore le diagnostic des performances et la biomécanique constituent des outils de base pour mesurer la contribution de ces différents éléments au succès. Ce dernier résulte donc d'une collaboration complexe et subtile entre une série de spécialistes des différents domaines concernés, dont l'orchestration exige un certain doigté.

Les compétitions occupent une place importante dans le succès d'une nation sportive. L'envoi de délégations, l'organisation d'épreuves, mobilisent une quantité de protagonistes et de compétences sans lesquels le succès reste hors de portée. Les managers sportifs possèdent bon nombre de ces aptitudes. Il faut également citer les arbitres et les dirigeants, dont l'impartialité garantit l'équité et la conformité réglementaire des compétitions.

Si l'on conçoit les succès olympiques comme l'aboutissement d'un processus de développement du sport nécessitant plusieurs années de travail, le regard se tourne vers de nouveaux champs de compétences. Faut-il concevoir des installations sportives satisfaisant à des exigences spécifiques du sport d'élite, les centres d'entraînement doivent-ils répondre aux véritables besoins de l'équipe? Le savoir-faire spécifique requis dépasse alors largement les connaissances spécialisées. Une certaine stratégie didactique, mais également politique est importante pour permettre aux athlètes de concilier sport d'élite et formation. Les sociologues, économistes, juristes et hommes politiques spécialistes du sport veillent à ce que toutes les pièces de la mosaïque indispensables à un succès durable soient en place, ce qui nécessite une certaine sensibilité aux performances d'élite exigées.

La Suisse abrite le siège principal de 67 fédérations et organisations internationales, notamment dans la région du Léman et dans celle de Zurich. Nombre de nos compatriotes, pionniers du sport, ont contribué à leur implantation dans notre pays. Leur mode de pensée et d'action cosmopolite est allé s'affaiblissant au cours des dernières années. Il serait bon de le ranimer, peut-être en proposant une formation spéciale destinée aux dirigeants du sport. Les règlements seraient élaborés au sein de commissions réunissant des fédérations internationales. Le sport doit être mis en scène et montré sous son meilleur jour à la télévision, et les règles favoriser des compétitions intéressantes et haletantes. Seuls les personnes capables de se mouvoir avec aisance sur la scène sportive peuvent fournir un travail de qualité. Derrière toutes ces exigences se dissimulent des champs de compétences et des professions que le public peine à imaginer.

Offres de formation destinée aux managers et aux dirigeants du sport
Un système sportif performant doit fournir des opportunités de formation – de base ou continue – dans les domaines les plus variés, dont les sciences du sport et de l'entraînement, mais aussi le management sportif.

Concernant cette dernière spécialité, le sport suisse propose différents cursus, tels que la formation en management de club dispensée par Swiss Olympic ou le programme pour les coachs J+S. De même, le Swiss Sport Management Center (SSMC) – fruit d'un projet mené par Swiss Olympic, la HEFSM, le Verbandsmanagementinstitut (VMI) et l'Institut de hautes études en administration publique (IDHEAP) (SSMC, 2011) – constitue une référence dans le domaine de la formation continue. Les cursus proposés par l'Académie Internationale des Sciences et Techniques du Sport (AISTS) de Lausanne, ou encore le «Master international en sciences humaines, management et droit du sport» (FIFA Master), à Neuchâtel, viennent compléter la gamme des formations destinées aux futurs managers et dirigeants du sport.

Du côté des universités et des hautes écoles spécialisées, de nombreux organismes proposent des cursus spécialisés dans les sciences du sport et/ou le management du sport. On citera entre autres les hautes écoles spécialisées de Coire et de Winterthour, ainsi que les universités de Berne, Zurich ou Lausanne (voir également le chapitre 10).

Le master Sport d'élite de la HEFSM apporte une formation permettant de relier les sciences de l'entraînement au management du sport. L'enseignement interdisciplinaire qui y est dispensé fait la part belle à la pratique. La HEFSM et la Formation des entraîneurs Suisse reconnaissent chacune les diplômes délivrés par l'autre (master Sport d'élite et Formation d'entraîneur diplômé), ouvrant ainsi la voie vers l'académisation des professions en lien avec le sport d'élite.

Une question reste cependant ouverte: les formations actuelles transmettent-elles la diversité des champs de compétences sportives? Pour permettre une croissance rapide du sport d'élite et répondre à ses exigences élevées, des connaissances tant internes qu'externes au sport constituent un avantage. Voilà pourquoi le sport helvétique n'a pas besoin de managers, ni d'économistes ou de juristes, mais de managers du sport, d'économistes du sport et de juristes du sport. Connaisseurs du système et de ses besoins, ils suscitent un véritable engagement. Un système sportif prônant un savoir interdisciplinaire dispose d'un outil pour améliorer les performances en compétition et en-dehors.

8. Recherche et développement

Pour réussir aux Jeux Olympiques, des performances de niveau mondial sont nécessaires, l'objectif étant d'avoir une longueur d'avance sur la concurrence. Plusieurs facteurs peuvent y contribuer: un entraînement efficace, un matériel sportif supérieur, mais aussi une meilleure gestion d'équipe et un meilleur système d'encouragement du sport. La recherche, le développement et l'innovation sont à cet égard tout aussi importants. Acquérir de nouvelles connaissances sur le sport d'élite est le meilleur moyen, pour une nation sportive, de s'assurer des médailles pour de nombreuses années.

Figure 8.1: Possibilité pour les athlètes et les entraîneurs d'exploiter les résultats de la recherche et du développement dans leur discipline

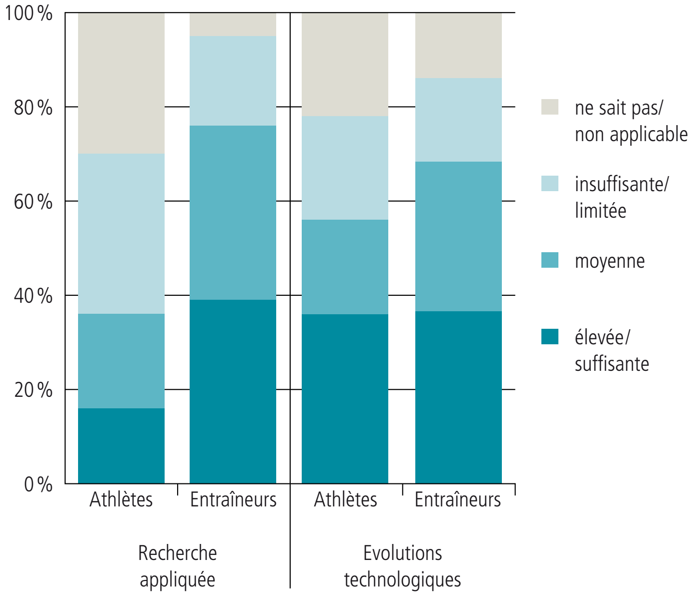

Source: SPLISS-CH 2011, n = 798 athlètes/374 entraîneurs

Perception de la recherche par les entraîneurs et les athlètes

Dans le sport d'élite, c'est l'instant qui compte. La concentration est essentielle, et chaque entraînement a son importance. Le déroulement de la journée est réglé à la minute près. L'athlète, son entraîneur et son coach mettent généralement l'accent sur l'entraînement. Pour eux, la recherche scientifique est rarement prioritaire, quand elle ne leur est pas parfaitement étrangère. Les bénéfices de la recherche doivent donc être évidents si l'on veut que les nouvelles connaissances soient intégrées dans le quotidien des athlètes. La recherche doit être accompagnée, expliquée et rendue accessible.

Dans le cadre de l'étude SPLISS-CH 2011, les athlètes et les entraîneurs ont été interrogés sur les possibilités dont ils disposent pour tirer parti de la recherche dans leur discipline sportive. La figure 8.1 montre que les athlètes exploitent avant tout les évolutions technologiques (matériel d'entraînement/de compétition et vêtements fonctionnels), reléguant au second plan la recherche appliquée (biomécanique, physiologie). Il est très révélateur que près d'un tiers des athlètes sondés répondent «ne sait pas/non applicable» au sujet de la recherche appliquée. Il en va différemment des entraîneurs: presque 40 % d'entre eux estiment que la recherche appliquée et les évolutions technologiques sont importantes pour leur travail.

Egalement interrogés sur la manière dont ils s'informent des évolutions dans leur discipline, les entraîneurs indiquent investir en moyenne deux heures par semaine pour approfondir leurs connaissances dans ce domaine. Ils se perfectionnent principalement en s'entretenant avec d'autres entraîneurs, mais aussi en lisant des revues spécialisées et en assistant à des cours de perfectionnement. La lecture d'articles scientifiques arrive bonne dernière: moins de la moitié des entraîneurs consultent régulièrement de tels articles. Par ailleurs, le comportement varie en fonction des sports pratiqués: les entraîneurs des disciplines des Jeux Olympiques d'été privilégient les revues spécialisées, tandis que ceux des sports pratiqués aux Jeux d'hiver sont plus portés sur la formation continue et le perfectionnement. Pour plus de détails à ce sujet, consulter le tableau 8.1.

Tableau 8.1: Entraîneurs s'informant eux-mêmes des évolutions dans leur discipline

	JO d'été	JO d'hiver
Echange d'expériences avec d'autres entraîneurs	87 %	85 %
Revues spécialisées	65 %	55 %
Formation continue/perfectionnement	53 %	64 %
Articles scientifiques	46 %	49 %

Source: SPLISS-CH 2011, n = 203/119 entraîneurs

Tableau 8.2: Entraîneurs informés sur la recherche et le développement par leur club ou la fédération nationale

	JO d'été	JO d'hiver
Evolutions en matière de méthodes d'entraînement	65 %	78 %
R&D scientifique dans la discipline concernée	53 %	68 %
Informations générales sur le sport d'élite	52 %	67 %
Idées en matière de management du sport	32 %	39 %

Source: SPLISS-CH 2011, n = 236/133 entraîneurs

Les entraîneurs sont aussi régulièrement informés de la recherche et du développement via les clubs et les fédérations nationales. Ces dernières fournissent des informations dans plusieurs domaines, les méthodes d'entraînement arrivant en tête de liste. Les fédérations des sports d'hiver semblent ici plus efficaces que celles des disciplines pratiquées aux Jeux Olympiques d'été, comme le montre le tableau 8.2 (données recueillies pour les douze derniers mois). Les informations sont préparées notamment par la HEFSM et Swiss Olympic avant d'être transmises aux entraîneurs directement ou par l'intermédiaire de la fédération. Elles sont ainsi diffusées lors du forum des chefs du sport d'élite, des Journées d'automne des entraîneurs et de la Conférence nationale pour la promotion de la relève. L'étude SPLISS-CH 2011 révèle que 72 % des entraîneurs ont intégré des résultats de la recherche scientifique dans leur travail au cours des douze derniers mois.

Les entraîneurs s'appuient sur des informations émanant de Swiss Olympic et de la HEFSM. Ils acquièrent dès leur formation d'entraîneur des connaissances scientifiques utiles, qu'ils complètent par la suite lors de cours de perfectionnement. Les experts se réunissent en groupes spécialisés pour échanger régulièrement sur les toutes dernières découvertes scientifiques dans les domaines suivants: force, endurance, psychologie/mental, sports d'équipe, médecine et alimentation. Dans la phase de préparation des Jeux Olympiques, des groupes de travail sont formés pour permettre aux membres des différents groupes spécialisés des domaines concernés (médecine, force, endurance, psychologie, alimentation, etc.) de partager les résultats de leurs recherches scientifiques. C'est ainsi qu'en prévision des Jeux Olympiques de Londres 2012, les entraîneurs se sont vu communiquer pour la première fois de précieuses découvertes des groupes spécialisés et des groupes de travail dans le cadre de programmes spécialement dédiés aux entraîneurs des athlètes olympiques.

Caractéristiques de la place de recherche suisse

La Suisse se distingue au niveau mondial par un environnement propice à la recherche et au développement. La majeure partie de la recherche suisse est financée par le secteur privé: ainsi, en 2008, sur les 16,3 milliards de francs consacrés à la recherche, 68 % provenaient de sources privées, 23 % à peine des pouvoirs publics, 6 % de l'étranger et 3 % de sources diverses (OFS, 2012b).

Bien que l'on possède très peu de données sur le financement de la recherche dans le domaine du sport d'élite, il semble que la part du secteur privé y soit considérable. Les prestataires sportifs et l'industrie travaillent en effet dans différents domaines de recherche, dont l'alimentation, la santé, l'habillement, le matériel sportif, les appareils d'entretien du matériel, le comportement de consommation des spectateurs et des lecteurs, etc.

La coopération avec des athlètes de haut niveau à travers des contrats de sponsoring pourrait être accrue afin de développer de nouveaux produits et appareils. Les équipes et clubs de renom (Team Alinghi, écurie Sauber de F1, etc.) possèdent souvent leur propre pôle de recherche, qui coopère généralement avec des entreprises d'envergure mondiale. Les avantages de ce type de recherche et de développement sont évidents: les nouvelles découvertes peuvent être gardées secrètes et devenir un atout stratégique dans la course aux victoires. De plus, pour peu que les résultats des recherches soient protégés par un brevet, il est possible de refinancer une partie des coûts d'investissement. Toutefois, rares sont les équipes qui peuvent s'offrir ce type de recherche.

Les fédérations sportives internationales (FIFA/UEFA et CIO) s'intéressent à la recherche et financent certains projets triés sur le volet. Les grandes fédérations sportives nationales telles que l'ASF, la SIHA, Swiss-Ski ou encore Swiss Tennis disposent de budgets de recherche fixes bien que modestes. Lorsqu'elles en ont les moyens, comme Swiss-Ski, elles tentent de créer des commissions permanentes chargées de discuter des efforts de recherche de leurs différents partenaires, de les coordonner ou au moins d'en assurer le suivi. Mais la plupart du temps, elles ne possèdent pas les ressources suffisantes pour se doter de leur propre service de recherche. Elles doivent par conséquent formuler des projets et émettre des mandats de recherche.

Certains instituts de recherche exécutent également, en tant qu'entreprises de droit privé, des mandats de recherche pour tous types de partenaires du domaine sportif (p. ex. Centre Suisse d'Electronique et de Microtechnique SA – Recherche et Développement, Lamprecht & Stamm, Sozialforschung und Beratung AG, Rütter + Partner et REMP Recherches et études des médias publicitaires).

La recherche au sein des hautes écoles spécialisées et des universités, financée par les pouvoirs publics, constitue un pilier important de la recherche sportive. Le «sport d'élite» est un sujet de recherche dans nombre de chaires des universités (Ecoles Polytechniques Fédérales de Zurich [EPFZ] et de Lausanne [EPFL], universités de Berne, de Fribourg, de Genève, de Lausanne, de Neuchâtel et de Zurich) et des hautes écoles spécialisées suisses (Haute école de Lucerne, Haute école spécialisée de Zurich, Haute école spécialisée bernoise, etc.), ainsi que dans les instituts rattachés à une haute école (p. ex. Centre international d'étude du Sport [CIES], VMI/SSMC, AISTS). Les budgets de ces institutions dépendent des cantons et, parfois, de la Confédération. La part consacrée au sport d'élite est très mal connue et difficile à estimer. En raison de l'obligation de publication à laquelle les universités sont généralement soumises, les nouvelles découvertes sont accessibles à tous, ce qui diminue leur intérêt pour le milieu sportif, car tout le monde peut profiter des mêmes avantages.

La HEFSM est la seule haute école de Suisse à se consacrer exclusivement à la recherche dans le domaine du sport et du sport d'élite. Elle mène des activités de recherche pour le compte de l'OFSPO dans le but de soutenir le sport de la relève et le sport d'élite axés sur le haut niveau (OFSPO, 2011c). Le travail de recherche de la HEFSM semblent constituer une grande partie de la recherche en sport d'élite encouragée au niveau national.

L'Institut pour l'étude de la neige et des avalanches (SLF) est un centre de recherche et de services interdisciplinaires situé à Davos (SLF, non daté). Traditionnellement, sa collaboration avec les sports d'hiver est essentielle. Il a par exemple assuré l'analyse du manteau neigeux et les prévisions météorologiques pour Swiss Olympic lors des Jeux Olympiques d'hiver de Vancouver en 2010 et de Sotchi en 2014. Enfin, Antidoping Suisse se charge de la recherche dans le cadre de la lutte antidopage (Antidoping Suisse, 2011). Cette fondation bénéficie du soutien financier de la Confédération et de Swiss Olympic.

Projets de recherche de droit public dans le domaine du sport d'élite

L'Etat encourage la recherche de manière ciblée en octroyant chaque année une aide financière aux projets de recherche les plus compétitifs. Il existe en Suisse plusieurs fonds de recherche nationaux auxquels le milieu sportif peut adresser des demandes de subvention, le Fonds national suisse (FNS) et le Fonds de la Commission pour la technologie et l'innovation (CTI) étant ici des acteurs centraux. Le tableau 8.3 indique la répartition des ressources allouées.

Jusqu'en 2010, le sport bénéficiait avec la Commission fédérale du sport (CFS) d'un fonds dédié. Mais la dissolution de la CFS en 2011 a mis fin à ces ressources. Désormais, ce fonds prend la forme d'une activité de recherche au sens strict de l'OFSPO, mais une partie des ressources continuent à être attribuées sur le marché de manière compétitive. Swiss Olympic soutient la recherche des fédérations sportives nationales avec le comité d'experts en sciences du sport de Swiss Olympic, qui dispose d'un budget annuel de 200 000 francs. Les fédérations peuvent confier leurs projets à ce comité, mais en les finançant à hauteur de 5 à 20 % avec leurs fonds propres. Le tableau 8.3 montre que la majeure partie des fonds de recherche est allouée par le FNS, avec les universités (65 %) comme premiers bénéficiaires, le reste étant principalement réparti entre les Domaines des EPF (26 %) et les hautes écoles spécialisées ou autres institutions (7 %). Les 2 % résiduels ne peuvent pas être affectés (SNF, 2011).

Tableau 8.3: Financement public de projets de recherche en 2010

Fonds	2010 (en millions de CHF)
Fonds national suisse (FNS)	726,0
Commission pour la technologie et l'innovation (CTI)	100,4
Commission fédérale du sport (CFS)	2,4
Comité d'experts en sciences du sport de Swiss Olympic	0,2

Source: tableau établi par les auteurs d'après les données du FNS (2011), de la CTI (2011) et de l'OFSPO (2011c)

Pendant la préparation des Jeux Olympiques de Londres 2012, le système sportif britannique s'est doté d'une équipe de gestionnaires de projet et de coordinateurs, appelée «UK Sport Research & Innovation Teams», dont la mission consistait à coordonner la pratique du sport d'élite, la recherche appliquée dans le domaine sportif et la recherche fondamentale à travers les différents types de services et disciplines sportives. Selon S. Drawer, cette équipe disposait pour ce faire d'un fonds annuel de quelque 3,1 millions de francs pour les différents projets pendant le cycle olympique préparant Londres 2012 (e-mail, 2 octobre 2013).

L'analyse des programmes et projets subventionnés montre que le sport n'a que peu bénéficié de l'attribution de ces ressources. En outre, les fonds sont généralement alloués selon des critères scientifiques élevés, requérant beaucoup de temps et de frais de coordination de la part des fédérations et des chercheurs.

Les ressources octroyées par le biais de la CTI à la recherche appliquée dans le domaine du sport d'élite sont elles aussi plutôt modestes, avec 0,7 million de francs en 2010 (CTI, 2012). Si l'on compare les besoins des disciplines sportives qui dépendent fortement de la technologie et du matériel avec les aides nationales accordées en 2010 dans le domaine des évolutions technologiques (100,4 millions de francs), on constate qu'il existe un potentiel considérable. Il suffit de penser à tous les sports requérant beaucoup de matériel qui pourraient bénéficier des connaissances techniques suisses. La difficulté d'accéder à ces ressources s'explique probablement par la part de financement devant être apportée par les entreprises. Ce critère d'attribution permet de contrôler la capacité de commercialisation des produits de recherche subventionnés. En d'autres termes: les résultats de la recherche doivent aussi être vendus. Or, comme il est rare que les nouvelles connaissances dans le sport d'élite se traduisent immédiatement par des produits commercialisables, les représentants des milieux sportifs doivent mettre au point des stratégies pour pouvoir malgré tout prétendre à ces ressources.

En comparaison, les ressources spécifiquement dédiées au domaine sportif sont presque négligeables. La part du fonds de la CFS (2,4 millions de francs) allouée au sport d'élite s'élevait à 0,5 million de francs par an en moyenne (OFSPO, 2011b). Parallèlement, le fonds du comité d'experts en sciences du sport de Swiss Olympic consacré à la recherche appliquée dans le domaine du sport d'élite en collaboration avec les fédérations est certes axé sur la pratique, mais très limité (seulement 0,2 million de francs). Or, même ce fonds ne peut pas être entièrement utilisé, les conditions d'obtention étant pour beaucoup trop strictes, y compris au sein du comité lui-même. Dans le paysage actuel de la recherche subventionnée par l'Etat, les représentants du domaine sportif peinent à fournir les efforts requis pour accéder à ces ressources.

Interdisciplinarité et applicabilité: deux facteurs de réussite
Pour exploiter l'immense pool de connaissances de la Suisse et pour le rendre accessible au sport d'élite, il faut intervenir aux niveaux les plus divers: en accompagnant l'activité de recherche et de développement, en regroupant les disciplines de recherche les plus variées et en garantissant l'accès à la recherche fondamentale.

Un facteur essentiel pour la recherche dans le domaine du sport d'élite est l'échange d'informations sur le terrain. Le chercheur doit noter les questions ouvertes et les attentes des athlètes, des entraîneurs et des coachs envers la recherche. Il doit ensuite les organiser de façon à ce que les thèmes (empiriques) puissent être étudiés et génèrent un bénéfice direct pour le sport. En effet, les exigences scientifiques restreignent souvent (trop) fortement les questions des praticiens.

Le sport en tant qu'objet de recherche se distingue par son interdisciplinarité et par les thèmes transversaux qui en découlent. La performance en compétition résulte d'un mélange de connaissances en matière d'entraînement, de matériel, de tactique et de coaching. Il faut donc coordonner toute une série de spécialistes et de domaines spécialisés dans les diverses «disciplines mères» (biologie, sciences de l'ingénieur, psychologie, sciences économiques, etc.) et les encourager à collaborer. Dans la phase de préparation d'une compétition, des domaines tels que l'organisation de projets et le management interviennent également. Et lorsqu'il s'agit d'organiser les systèmes de promotion de la relève dans le sport d'élite ou d'exploiter et de garantir de nouveaux potentiels de savoir, c'est au tour des questions juridiques et des aspects réglementaires de prendre de l'importance. La diversité des thèmes impliqués pour la recherche dans le domaine sportif est impressionnante.

En fin de compte, l'essentiel est de faire le lien entre la recherche axée sur la pratique et les résultats de la recherche fondamentale. Mais même les sciences appliquées – sans parler de la recherche fondamentale – sont déjà bien éloignées du travail quotidien qu'effectuent les entraîneurs et les coachs avec les athlètes. La Suisse, qui figure parmi les pays les plus innovants de la planète, possède en la matière un potentiel considérable qui peut être exploité pour le sport. En conséquence, il faut faire en sorte que les grandes universités et les écoles polytechniques fédérales (EPFZ/EPFL) deviennent des partenaires pour le milieu sportif.

Le sport suisse doit saisir cette opportunité. Une chose est claire: plus les objectifs sont ambitieux et fixés sur le long terme, plus la recherche interdisciplinaire et l'échange avec les acteurs de la recherche fondamentale sont importants. Même si la coordination des différentes sphères de la recherche est coûteuse en termes de temps et d'argent, il faut la considérer comme un investissement pour l'avenir du sport d'élite.

9. Installations sportives et centres d’entraînement

Les champions sont le fruit d’un entraînement quotidien très poussé. Au sein des camps et des centres d’entraînement, ils bénéficient de différentes synergies: esprit de compétition, environnement favorisant les performances, mutualisation des connaissances, échange entre les athlètes, les entraîneurs et les coachs, peu de trajets et accès aux services les plus divers à des tarifs préférentiels – tous ces éléments constituent la base d’un succès durable. Il est donc important, pour atteindre ses objectifs lors des compétitions internationales, de vérifier que le pays possède un nombre suffisant d’installations et de centres de compétences et de performance de qualité et en bon état.

Figure 9.1: Evaluation par les athlètes de la qualité et de la disponibilité des installations de compétition

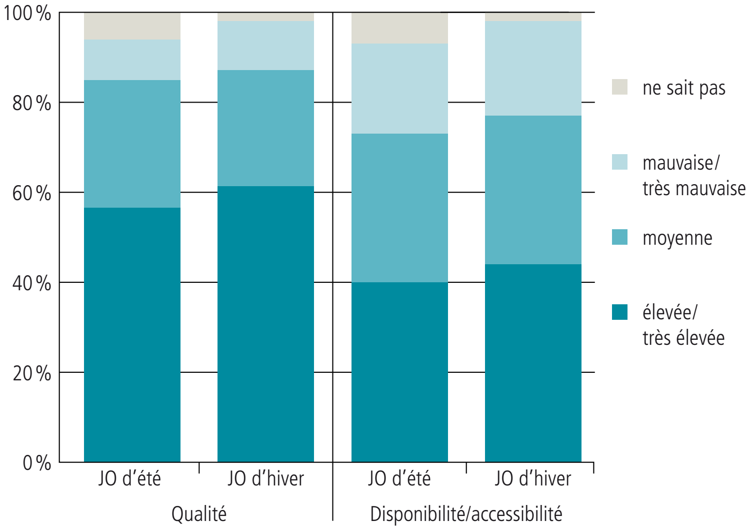

Source: SPLISS-CH 2011, n = 362, 276, 359 et 273 athlètes selon la question

Evaluation des installations d’entraînement et de compétition

Les installations sportives sont l’un des facteurs essentiels de réussite. Les athlètes estiment que les installations actuelles sont relativement bonnes: 60 % d’entre eux jugent leur qualité «élevée» ou «très élevée». En revanche, leur disponibilité ou accessibilité laisse à désirer: près de 20 % des athlètes interrogés la trouvent «mauvaise/très mauvaise». La figure 9.1 montre également que la situation en hiver est légèrement meilleure qu’en été. La mauvaise note attribuée à la disponibilité s’explique peut-être par l’impossibilité, pour certains athlètes, d’utiliser les installations aux moments où ils le souhaitent, ou par le fait que leur domicile se situe très loin de ces dernières. Quoi qu’il en soit, il existe un potentiel d’amélioration pour satisfaire aux exigences très élevées qui caractérisent habituellement le sport d’élite.

Quelle est l’importance des centres pour la qualité de l’entraînement? A cette question, 60 % des entraîneurs ont répondu qu’il y avait effectivement un rapport entre la disponibilité d’un centre d’entraînement et la qualité de ce dernier, qu’il s’agisse des disciplines pratiquées aux Jeux Olympiques d’été ou de celles des JO d’hiver. Il est surprenant que cette valeur ne soit pas plus élevée.

La réponse à la question portant sur l’accès aux centres d’entraînement est intéressante: les athlètes bénéficiant actuellement d’un accès illimité à un centre d’entraînement accordent plus d’importance à un centre d’entraînement national ou à une base d’entraînement régionale que les athlètes ne disposant pas encore d’un tel accès. Toutefois, 70 % des athlètes ne possédant actuellement pas d’accès à un centre national ou à une base régionale seraient ravis d’en avoir un à l’avenir.

Offre d’installations sportives

La Suisse se distingue par sa densité élevée d’installations sportives pour le sport de masse. Près de 80 % des 2117 communes interrogées disposent d’au moins une salle de sport et/ou une salle polyvalente, et 50 % d’entre elles possèdent une installation sportive extérieure pour le complexe concerné (Balthasar, 2009). Une nouvelle étude réalisée en 2012 confirme l’impressionnante diversité d’installations, avec pas moins de 31 000 éléments d’installation identifiés (Balthasar *et al.*, 2013). Le tableau 9.1 montre la répartition des différents types d’installations.

Tableau 9.1: Installations sportives en Suisse en 2012

Type d'installation	Nombre
Installations de plein air	11 736
Salles de gymnastique et de sport	6 779
Piscines (couvertes, extérieures, etc.)	2 084
Installations de sports de glace	411
Installations spécifiques à une discipline sportive	10 979
**Total**	**31 989**

Source: tableau établi par les auteurs d'après les données de Balthasar *et al.* (2013)

En raison de l'organisation fédéraliste de la Suisse, les installations sportives voient généralement le jour suite à une initiative communale. Ce sont des investisseurs privés qui réalisent les projets d'infrastructures sportives, par lesquels les communes cherchent à se profiler. Très souvent, les clubs et les fédérations sportives vont à la rencontre des investisseurs et des communes dans l'espoir de pouvoir faire réaliser leurs installations. La construction des installations a donc lieu selon un processus ascendant, appelé «bottom-up».

Une question faisant l'objet d'âpres débats est la finalité des installations: dans quelle mesure les complexes prévus pour le sport de masse sont-ils appropriés pour le sport d'élite, et comment garantir leur disponibilité pour celui-ci? La plupart des fédérations dépendent, pour la construction de leurs installations et de leurs centres d'entraînement, de la bienveillance privée mais surtout publique envers le sport d'élite.

Conception des installations sportives d'importance nationale

L'inventaire des installations sportives d'importance nationale (Inventaire CISIN) est la seule source fiable répertoriant les infrastructures dédiées au sport d'élite. On y trouve (état en 2010) 15 centres de sport polyvalents, 14 salles de sport polyvalentes, 11 centres et salles monosportifs, 9 installations de sports sur gazon et d'athlétisme, 10 installations de sports aquatiques et nautiques, 12 installations de sports de glace, 16 installations de sports de neige et 14 installations diverses (OFSPO, non daté, b).

Avec sa Conception des installations sportives d'importance nationale (CISIN), la Confédération tente de créer des conditions favorables pour les fédérations sportives nationales en termes d'installations sportives d'importance nationale. Elle subventionne ainsi, dans le cadre des crédits obligatoires de la CISIN, des infrastructures sélectionnées. Par voie d'arrêté fédéral, le Parlement a accepté, en 1998, en 2000 et en 2007, trois crédits d'engagement de respectivement 60 (CISIN 1), 20 (CISIN 2) et 3,14 millions de francs (CISIN 3), soit une aide financière de 94 millions au total, pour financer la réalisation d'installations sportives d'importance nationale. Le Conseil fédéral a adopté en 2012 le message concernant l'octroi d'aides financières pour des installations sportives d'importance nationale (CISIN 4), pour un crédit total de 50 millions de francs. Le Parlement a porté le crédit d'ensemble à 70 millions de francs. Les contributions accordées par la Confédération représentent 5 à 25 % des frais de construction donnant droit à subvention des différents projets concernés (OFSPO, non daté, b).

A travers la CISIN, la Confédération souhaite regrouper et structurer les ressources. Chaque projet CISIN fait ainsi l'objet d'un contrat d'utilisation entre la fédération sportive nationale et l'autorité responsable de l'installation, ainsi que d'un contrat de subvention entre l'exploitant de l'installation et la Confédération. Désormais, il faut également prouver la nécessité de l'installation pour le concept de sport d'élite de la fédération. De nombreux cantons se sont dotés d'une conception des installations sportives d'importance cantonale (CISIC) afin de tenter de piloter les initiatives en la matière. Pour répondre aux besoins spécifiques, les représentants de disciplines sportives «mineures» sont généralement encouragés à centraliser leur infrastructure sportive.

Tableau 9.2: Centres d’entraînement et de performance des fédérations les plus prolifiques en termes de résultats (sports d’été et sports d’hiver)

Sport	Nbre de centres d’entraînement nationaux	Remarques concernant la structure
Cyclisme (VTT, piste, route et BMX) (Swiss Cycling)	–	La fédération exploite un centre d’entraînement national depuis 2013. Les centres régionaux existants disposent chacun de cinq bases d’entraînement régionales et de cadres régionaux associés.
Equitation	–	La fédération ne possède pas son propre centre d’entraînement. Il existe trois centres d’équitation dont l’infrastructure est régulièrement utilisée par la fédération lors des camps d’entraînement. Afin de limiter autant que possible les transports des animaux, les athlètes s’entraînent principalement dans les installations situées à proximité de leur domicile.
Aviron	1	La fédération cite explicitement un seul centre d’entraînement national et aucun centre régional afin de mettre en commun les ressources. Les clubs ont la possibilité de faire reconnaître par la fédération des bases d’entraînement locales.
Triathlon	1	La fédération exploite de fait un centre d’entraînement national, tout en proposant une structure saisonnière: elle met ainsi à disposition une base d’entraînement à Tenero de décembre à juin, et une autre à Davos de juillet à septembre. Les conditions climatiques semblent jouer ici un rôle important. La fédération étudie la possibilité de se concentrer sur un seul centre.
Ski (ski alpin et snowboard) (Swiss-Ski)	3	La fédération exploite trois centres d’entraînement nationaux (régions Est, Centre et Ouest). Concilier sport et études est important. Les centres d’entraînement régionaux sont très répandus et ils ont été mis en place principalement en fonction des besoins des skieurs alpins.
Glisse (bob, luge et skeleton) (Swiss Sliding)	3	La fédération propose trois centres d’entraînement nationaux, axés chacun sur les différentes exigences de l’entraînement (entraînement athlétique, poussée en bob, entraînement sur glace). Seule la luge dispose déjà d’une base d’entraînement locale.
Curling	–	Le curling se caractérise par un ancrage très local. Il existe actuellement un seul contrat d’utilisation pour l’entraînement de la fédération, signé avec la halle de curling de Berne. Un centre d’entraînement national est toutefois prévu.

Source: tableau établi par les auteurs d’après les données de Swiss Olympic (2013)

Centres de sport et de formation de la Confédération

Posséder des installations sportives, c’est bien; mettre à disposition des centres d’entraînement complets, c’est encore mieux. L’OFSPO encourage le sport d’élite à travers le Centre national de sport de Macolin et le Centre sportif national de la jeunesse de Tenero (CST). A Macolin, il propose une infrastructure d’entraînement et d’hébergement, ainsi que des services médicaux et d’information en matière de sciences de l’entraînement. Les installations et l’exploitation sont prises en charge par la Confédération, avec l’aide financière de Swiss Olympic ou de fédérations sportives nationales pour certaines installations. Le CST est le centre sportif de la jeunesse de la Confédération. Il offre des possibilités de formation et d’entraînement pour le sport de masse et le sport d’élite afin d’assurer la relève.

Des contingents d’utilisation et de services ont été convenus avec les fédérations pour les centres de sport et de formation. Des athlètes soigneusement sélectionnés peuvent, en plus de l’infrastructure sportive et de l’hébergement, bénéficier gratuitement des services de la HEFSM en termes de sciences des sports et des prestations du Swiss Olympic Medical Center Macolin-Bienne (OFSPO, non daté, d).

Le Centre d’entraînement sportif de l’armée d’Andermatt sert de centre de formation pour les sports de neige. C’est là qu’a lieu la formation des jeunes et des cadres de J+S, avec les modules correspondants spécifiques aux compétitions.

Le processus d’évaluation des différents sites susceptibles d’accueillir un nouveau centre national de sports de neige dédié au sport scolaire et à la relève sportive devrait être terminé fin 2013. Ce centre complètera efficacement l’infrastructure existante.

Figure. 9.2: Centres sportifs de la Confédération, centres d'entraînement des fédérations les plus prolifiques en termes de résultats (sports d'été et sports d'hiver), Swiss Olympic Medical Centers et Swiss Olympic Schools

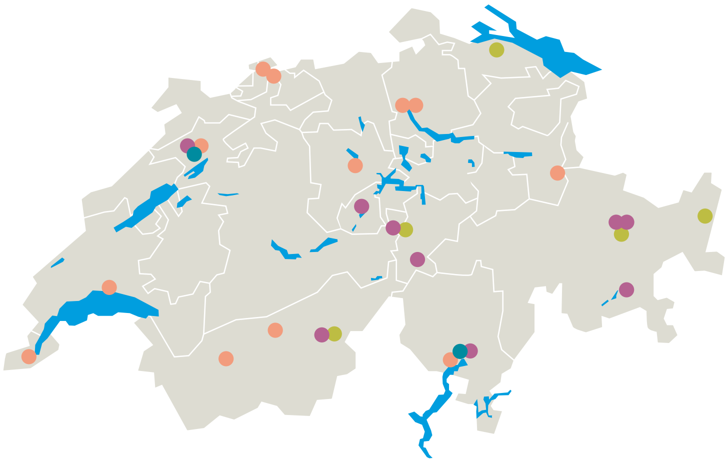

Source: figure établie par les auteurs d'après les données de Swiss Olympic (2010b)

Centres d'entraînement et de performance des fédérations sportives

Les fédérations sportives nationales possédant d'importantes ressources financières se dotent souvent de propres centres d'entraînement et de performance. Destinés à compléter l'offre sur le court terme et à permettre l'entraînement régulier tout au long de l'année, ces centres sont avant tout axés sur les besoins d'un sport précis et ils disposent d'infrastructures spécifiques (p. ex. pour le ski alpin: filets, pistes sécurisées, etc.). La plupart du temps, ils ne sont pas directement gérés par une fédération sportive nationale, mais par toutes sortes d'instances chefs. Ces dernières garantissent les services et l'infrastructure dont a impérativement besoin toute fédération sportive.

La diversité des offres est grande et elle varie d'un sport à l'autre. Pour s'en rendre compte, il est utile de considérer de plus près les sports d'été et d'hiver ayant remporté le plus de médailles entre 1988 et 2012. Le tableau 9.2 établit un état des lieux sur la base des déclarations de ces fédérations.

Le nombre de médailles obtenues est-il lié à la qualité de l'offre en matière de centres d'entraînement pour le sport concerné? Les données ne permettent pas de répondre à cette question. De plus, les fédérations entendent par «centres d'entraînement» des complexes des plus divers. Or, ces centres doivent procurer aux athlètes des offres de services individualisées pour leur entraînement, leur récupération et leur formation. Le sport est au cœur de tout. Des hébergements gratuits, tels que les proposent par exemple Swiss Olympic dans ses centres d'entraînement et l'OFSPO à Macolin et à Tenero, sont utilisés par les fédérations. Pour les différents sports, c'est une procédure pragmatique qui semble être privilégiée, quitte à négliger l'encadrement global.

Swiss Olympic Medical Centers et Sport Schools

La bonne santé des athlètes est une condition *sine qua non* du succès. Le soutien apporté par la médecine et la thérapie du sport est donc essentiel. Swiss Olympic s'efforce de contrôler ce domaine au moyen de certifications. Les Swiss Olympic Medical Centers sont des cliniques et des centres d'examens médicaux de grande taille, dont la mission consiste entre autres à assurer la prise en charge médicale des sportifs d'élite et des jeunes talents au niveau national. Les principales compétences des douze Swiss Olympic Medical Centers (état en 2011) sont les examens de médecine sportive et de laboratoire ainsi que le diagnostic de performance et l'encadrement médical de l'entraînement. Ces centres doivent renouveler leur certification tous les quatre ans pour pouvoir conserver le label Swiss Olympic. En 2011, la Suisse comptait en outre 23 «Medical Bases» certifiées (Swiss Olympic, 2008b).

Afin de soutenir la filière de formation complémentaire (formation et sport d'élite) des athlètes, Swiss Olympic est à la recherche de collaborations avec les organismes de formation (écoles et entreprises formatrices). En contrepartie, celles-ci peuvent demander les labels «Swiss Olympic Partner School», «Swiss Olympic Sport School» et «Entreprise formatrice favorable au sport de performance». Quatre des cinq Swiss Olympic Sport Schools sont en outre chaque année subventionnées à hauteur de 300 000 francs au total, des fonds découlant de la motion Hess. Les 43 Swiss Olympic Partner Schools bénéficient quant à elles d'un petit soutien financier. La vignette «Entreprise formatrice favorable au sport de performance», introduite par Swiss Olympic lors de la saison 2009/10, a été octroyée plus de 100 fois depuis son lancement jusqu'en 2011 (Swiss Olympic, 2011d).

Potentiel de synergie entre les différents systèmes de centres
La figure 9.2 montre la répartition géographique des centres de formation et de performance, des centres médicaux et des écoles de sport. Seuls les établissements de niveau national ou détenteurs d'un label de qualité élevée ont été pris en compte.

Lorsque l'on compare les emplacements des centres de sport et de formation de la Confédération et des fédérations les plus prolifiques en termes de résultats avec ceux des établissements certifiés, on constate un potentiel de synergie. Les centres d'entraînement se trouvent dans l'espace alpin et dans le centre de la Suisse, et les Medical Centers dans les grandes agglomérations qui concentrent également les universités et les hôpitaux. Il en va de même des écoles de sport. De nombreux partenaires intéressants en termes de recherche et de développement dans le sport (voir le chapitre 8) se trouvent également dans les agglomérations. Si la carte répertoriait les installations sportives d'importance nationale (Inventaire CISIN) des fédérations ainsi que les Swiss Olympic Training Bases, l'offre de centres d'entraînement et de performance déclarés couvrirait presque la totalité du territoire Suisse.

Selon D. Bake (2013b), les Pays-Bas disposent d'un réseau de centres d'entraînement polyvalents qui se distinguent les uns des autres par leur spécialisation et leur orientation. Les quatre «centres de sport d'élite et de formation» sont pourvus d'équipements polyvalents, ce qui permet aux jeunes talents de différentes disciplines de s'entraîner dans le même environnement tout en suivant leur formation. Dans les trois «centres d'entraînement nationaux», un seul type de sport est généralement pratiqué, mais des disciplines apparentées sont parfois également représentées. L'infrastructure et les services sont fonction des exigences inhérentes à ces disciplines sportives. A ce niveau, on observe une spécialisation dans un type de sport, sur la base de connaissances scientifiques très spécifiques développées en parallèle. La centralisation, au sein de ces sites, d'offres de formation et d'entraînement propres à un type de sport en améliore la qualité.

Les centres d'entraînement permettent de réduire les trajets et d'accroître ainsi le temps consacré à l'entraînement et au repos, tout en augmentant la qualité de l'entraînement. Ils offrent aux athlètes un environnement propice au sport d'élite (savoir, compétition, etc.), notamment grâce aux services proposés dans les domaines des sciences de l'entraînement, de la technologie appliquée au matériel et du suivi médical. Les besoins en matière de sport et de formation peuvent ainsi être optimisés de manière conjointe. Les centres d'entraînement deviennent encore plus précieux lorsqu'on y exploite en outre les synergies pour la recherche et le développement.

Cependant, la figure 9.2 montre bien la problématique du sport d'élite suisse: la coordination de la politique de la Confédération axée sur l'offre, des solutions spécifiques aux différents sports mises en œuvre par les fédérations et des certifications de Swiss Olympic pourrait être améliorée, mais les moyens financiers font défaut. De plus, les centres ne possèdent pas l'envergure requise pour exploiter au mieux les synergies, ni pour satisfaire de manière optimale les besoins des fédérations et des athlètes.

Il ne serait pas judicieux pour la Suisse de se doter d'une structure trop centralisée. Compte tenu de la fonction d'exemplarité qu'assument les sportifs d'élite pour les jeunes athlètes se trouvant dans leur environnement proche (voir le chapitre 5), des insuffisances constatées au niveau de la promotion sportive intégrative à l'échelle régionale, et des multiples besoins en termes de formation (voir le chapitre 6), il est évident que seul un ingénieux réseau de centres d'entraînement pourra répondre efficacement aux besoins réels des athlètes suisses. En tant que nation sportive, la Suisse doit chercher à évoluer dans cette direction. Une centralisation absolue aurait pour effet l'érosion des connaissances sur le sport d'élite dans les régions.

10. Compétitions internationales

Formidable vitrine du sport, les Jeux Olympiques constituent une véritable opportunité pour les athlètes comme pour les fédérations. L'entraînement des sportifs n'est pas très attrayant en soi: seule la compétition, c'est-à-dire l'affrontement avec les adversaires et la concurrence internationale, permet d'exploiter le potentiel commercial du sport. Et dans ce domaine, l'exposition des disciplines les plus diverses durant les Jeux Olympiques est une vraie réussite puisqu'elle fait ressortir le potentiel du sport en termes de marketing (toutes disciplines confondues), de développement de nouveaux sports et de synergies dans le cadre de la promotion du sport d'élite.

Importance des compétitions pour les athlètes

Gardant toujours à l'esprit les Jeux Olympiques, les athlètes se préparent pendant des années pour le jour J. Les compétitions représentent pour eux le meilleur entraînement qui soit, puisqu'ils peuvent y étalonner leurs performances et obtenir des points de comparaison. Elles leur donnent l'occasion d'observer leurs concurrents et d'en tirer des enseignements. Face aux grands objectifs internationaux, offrir de bonnes compétitions préparatoires est un gage de qualité.

Près de 80 % des athlètes interrogés jugent la fréquence et le niveau des compétitions en Suisse «moyens» à «élevés/très élevés» (voir la figure 10.1). Une appréciation qui vaut tant pour les compétitions d'élite que pour celles de la relève, même si c'est le niveau de ces dernières qui obtient les moins bons résultats.

L'analyse approfondie des données SPLISS-CH 2011 indique que la fréquence et le niveau des compétitions sont critiqués davantage par les athlètes des sports d'été que par ceux pratiquant les disciplines d'hiver, et ce pour les deux types de compétitions.

Offre de compétitions en Suisse

Au total, la Suisse accueille chaque année environ 230 000 manifestations sportives, de toutes tailles et catégories. Cela illustre une nouvelle fois l'ancrage du sport dans ce pays, qui se distingue par une grande diversité et par la couverture de l'ensemble du territoire. La catégorie des grandes manifestations a été examinée de plus près: disposant d'un budget de plus de 1 million de francs, elles sont retransmises en direct à la télévision suisse dans le cadre d'émissions spéciales.

Figure 10.1: Evaluation par les athlètes des compétitions destinées à la relève et à l'élite en Suisse

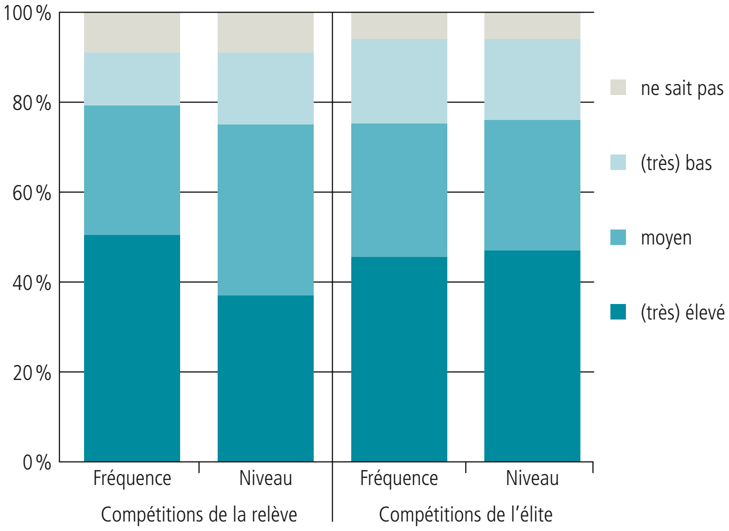

Source: SPLISS-CH 2011, n = 800 athlètes

Pour entrer dans cette catégorie, les événements doivent par ailleurs attirer au minimum 10 000 participants, 1000 dirigeants sportifs ou encore 20 000 spectateurs (Stettler *et al.*, 2008). Les «méga-événements» tels que les Jeux Olympiques, la Coupe du Monde et le Championnat d'Europe de football vont naturellement bien au-delà de ces critères.

La Suisse dispose d'une solide tradition en matière d'organisation de grandes manifestations sportives. Entre 2009 et 2011, pas moins de 18 championnats internationaux de disciplines olympiques s'y sont en effet déroulés tant au niveau du sport d'élite que de la relève. Le pays compte en outre plus de 80 grandes manifestations sportives récurrentes, qui ont lieu chaque année (calcul établi par les auteurs d'après les données de Stettler *et al.*, 2008). Vingt d'entre elles sont organisées dans le cadre de l'association SwissTopSport (STS). Ces manifestations d'envergure renforcent la position de la Suisse en tant que nation sportive et jouent un rôle de vitrine du sport international dans ce pays (STS, 2011).

Les ligues occupent une place particulière au sein du sport d'élite. Elles coordonnent l'organisation du sport professionnel et proposent des compétitions au plus haut niveau. Les deux ligues professionnelles (SFL et NL) constituent un contrepoids face aux fédérations nationales (ASF et SIHA) qui représentent à la fois le sport d'élite et le sport de masse. Les compétitions sont essentielles pour les athlètes comme pour les fédérations: elles jouent un rôle de tremplin pour une carrière internationale dans les ligues majeures. Des stades bien remplis garantissent par ailleurs des revenus et de la publicité.

Au sein des grandes manifestations sportives, on distingue généralement les événements uniques (p. ex. Mondial et Euro de football) et les événements récurrents (p. ex. Coupe du Monde de ski). Les premiers doivent repartir à chaque fois de zéro à l'instar des entreprises start-up dans le secteur économique, et ils ouvrent de nouveaux débouchés en termes de promotion sportive et économique. Ces effets sont alors développés et consolidés par les manifestations récurrentes, qui représentent en outre le fondement d'une candidature réussie pour accueillir de grands événements uniques (voir Stettler *et al.*, 2008, 2011). La collaboration internationale en matière de préparation des grandes manifestations sportives, pour sa part, renforce l'influence et la position de la Suisse pour obtenir le siège des fédérations internationales (Rütter *et al.*, 2013).

Procédure de candidature pour les grandes manifestations sportives

La plupart des manifestations sportives suisses sont solidement ancrées dans le circuit international. Les organisateurs postulent pour accueillir ces événements à différents niveaux de compétition (national, continental et mondial). Ils essaient ainsi d'obtenir l'un des rendez-vous figurant au calendrier de leur discipline (championnats du monde/d'Europe, Coupe du Monde, etc.) tout en respectant les règlements de leur fédération internationale, qui définissent le profil d'exigence et le mode d'organisation des différents formats de compétitions à l'échelle concernée. Pour les fédérations sportives nationales et les organisateurs, la priorité est donc de décrocher des dates dans le calendrier international.

Les autorités communales sont les premiers interlocuteurs des fédérations nationales et/ou des organisateurs lorsque ceux-ci envisagent de déposer leur candidature pour une grande manifestation sportive. En collaboration avec les autorités cantonales, il s'agit alors de vérifier la faisabilité du projet et de clarifier les éventuelles conditions d'autorisation (OFSPO, 2011d).

Les fédérations sportives nationales peuvent adresser des demandes d'aide à Swiss Olympic. Combiné à celui de la Confédération, ce soutien permet d'assurer jusqu'à un certain point la coordination des grandes manifestations sportives en Suisse. Swiss Olympic a par ailleurs introduit une planification pluriannuelle, fournissant ainsi au sport suisse un «calendrier des manifestations sportives internationales» sur un horizon de huit à dix ans.

Depuis 2012, sur le modèle du rythme de planification des fédérations sportives et des organisateurs, les contributions de la Confédération sont fixées dans le cadre d'une planification pluriannuelle coordonnée avec Swiss Olympic. Cette base permet de déterminer non seulement les subventions que la Confédération pourra, selon les prévisions, allouer à la manifestation concernée dans le cadre du budget ordinaire, mais aussi les grands événements pour lesquels une demande de fonds doit être déposée via un message spécial. Si la planification pluriannuelle apporte aux organisateurs davantage de sécurité pour la programmation des événements, la Confédération peut pour sa part, grâce à cet instrument, mener une politique de soutien orientée à moyen et long terme et intégrée à la planification financière globale (OFSPO, 2011d).

Intérêts de l'industrie du sport vis-à-vis des grandes manifestations sportives

Différents facteurs interviennent dans les procédures de candidature et d'organisation des grandes manifestations sportives, qu'elles soient uniques ou récurrentes. Le sport doit ainsi compter dans ses rangs des organisateurs capables de gérer des événements de qualité. En particulier pour la relève et les jeunes athlètes d'élite, les fédérations nationales et les clubs doivent pouvoir s'appuyer sur des compétitions fair-play et réunissant une bonne participation, qui constituent alors des jalons importants pour les athlètes et facilitent le développement des nouveaux talents, ainsi que la sélection de la relève. Il n'est d'ailleurs pas rare que ces compétitions bénéficient d'un financement croisé de la part des fédérations nationales.

Plus le niveau des participants est élevé, plus il y a de chances que le financement des événements soit à nouveau assuré. Lorsque les organisateurs parviennent à occuper des plages de diffusion à forte audience à la télévision (notamment au plan international), un cercle vertueux se met en place: la couverture télévisée garantit la participation de sponsors qui, en communiquant sur leur engagement, augmentent l'intérêt du public pour la manifestation et génèrent par là même davantage de téléspectateurs, ce qui permet d'assurer la retransmission télévisée. Cette logique permet de comprendre l'attractivité des grandes manifestations au sein de l'industrie du sport. Le financement provient de la vente des droits audiovisuels, des contrats de sponsoring ou encore de la billetterie. Les grands événements facilitent également l'exploitation de nouveaux débouchés tels que les réseaux sociaux et les paris sportifs.

Figure 10.2: Evolution des droits de retransmission des Jeux Olympiques 1988-2012 en millions de USD

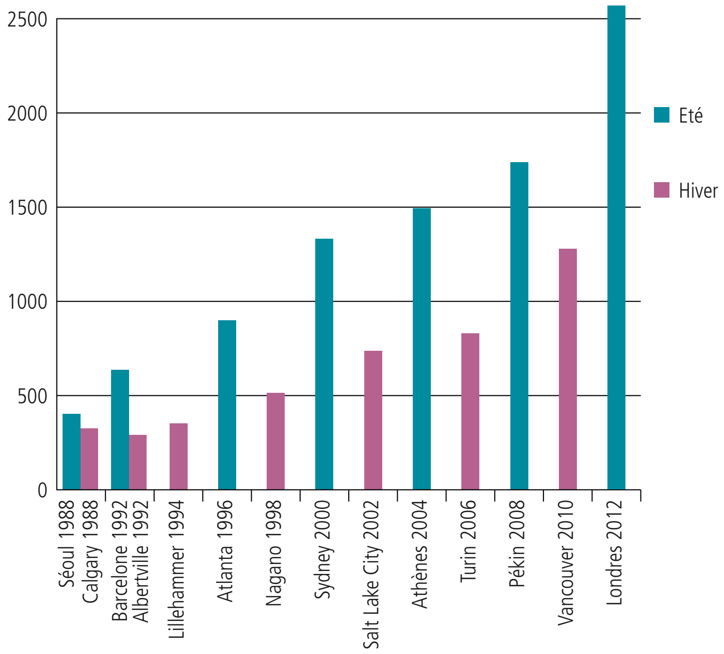

Source: figure établie par les auteurs d'après les données du CIO (2013b)

La force des grandes manifestations sportives réside dans la structuration, le regroupement et, idéalement, dans la commercialisation collective des droits communs à plusieurs organisateurs. Pour leur part, les annonceurs ont la possibilité de soigner leur image et d'accroître leur notoriété de manière efficace et au niveau mondial grâce aux canaux mis à disposition via le sport. Ils s'associent souvent pour ce faire à d'autres sponsors et créent ainsi d'importants effets de réseau rendant les produits spécialement attrayants. Ces principes s'appliquent en particulier aux «méga-événements».

Le rôle moteur des méga-événements

L'organisation du championnat d'Europe de football (EURO) 2008 a permis à la Suisse de faire l'expérience d'un méga-événement, et de ses incidences complexes (voir Müller *et al.*, 2010). En étudiant la croissance du sponsoring et de la publicité en Suisse, on note un transfert des moyens alloués au sport (Müller *et al.*, 2010). De nouveaux sponsors ont également fait leur entrée dans le domaine sportif (Sport+Markt, 2011). Et une croissance supérieure à la moyenne a été enregistrée au sein de l'industrie du sport suisse dans le segment de services correspondant, ce qui s'explique notamment par la conquête de nouveaux marchés à l'étranger (voir Rütter *et al.*, 2011).

Les méga-événements ont depuis longtemps quitté la seule enceinte des stades pour devenir un phénomène sociétal aux multiples effets externes, tant positifs que négatifs. L'engagement de la Confédération pour la promotion des grands événements sportifs internationaux en Suisse repose sur leur importance pour la promotion du sport, mais aussi sur leur influence en termes d'image et d'identité du pays. Les méga-événements favorisent par ailleurs la cohésion sociale, le tourisme, la préservation des infrastructures et l'entretien du réseau de relations de la Suisse (OFSPO, 2011d). Les pouvoirs publics soutiennent généralement l'initiative privée de manière subsidiaire en affectant leurs propres fonds – dans le cas de l'EURO 2008 par exemple – à l'infrastructure, aux transports, à la promotion du pays (tourisme) et à la sécurité (Structure des pouvoirs publics UEFA EURO 2008, 2008).

Les pouvoirs publics encouragent également les mesures d'accompagnement du sport, comme l'illustre l'évolution du soutien financier du sport par l'OFSPO. L'EURO 2008 a ainsi entraîné une hausse des dépenses de l'OFSPO plus importante que les années précédentes: si ces dépenses avaient augmenté de 9,5 % entre 2000 et 2005, le taux est passé à 15 % entre 2006 et 2010 (OFSPO, 2011b). Le système de promotion du sport a donc bénéficié de plus de moyens grâce à l'EURO 2008, et ce sur le long terme; les programmes d'encouragement ont pu être mis en place plus facilement et financés de façon durable. Le projet J+S-Kids, par exemple, a été lancé en 2007 en amont de l'EURO 2008, avant de devenir le programme Sport des enfants J+S, inscrit depuis 2012 dans la loi sur l'encouragement du sport.

Le pouvoir d'attraction des Jeux Olympiques

Les effets des grandes manifestations sportives sont particulièrement marqués dans le cas des Jeux Olympiques (voir Preuss, 2007; Gratton & Preuss, 2003; Chappelet, 2002; Rütter *et al.*, 2013). Le pouvoir d'attraction exercé par cet événement est dû à sa couverture médiatique exceptionnelle, au degré de reconnaissance des anneaux et à la répartition des moyens financiers. Le marketing ciblé, initié dans les années 1980, a permis d'augmenter cette manne. La séparation des Jeux Olympiques d'été et des JO d'hiver, ainsi que les améliorations constantes de la mise en valeur et de la vente des contrats de marketing ont entraîné une croissance très importante. Les recettes engrangées par le CIO pour les droits de retransmission sont en effet passées de 403 millions de dollars (Séoul, 1988) à 2569 millions de dollars (Londres, 2012), et de 325 millions de dollars (Calgary, 1988) à 1280 millions de dollars (Vancouver, 2010) (CIO, 2013b). Cette évolution est illustrée par la figure 10.2. D'autres recettes supplémentaires ont par ailleurs pu être générées, notamment via les partenaires Top des Jeux Olympiques, dont les recettes ont décuplé entre 1988 et 2012 (de 96 millions à 950 millions).

La croissance des Jeux Olympiques a une influence sur le sport d'élite des nations participantes, du fait de la gigantesque machine à marketing qu'ils mettent en mouvement et des énergies libérées par le tourbillon médiatique. Les différents acteurs nationaux unissent en effet leurs forces et mettent des moyens supplémentaires à disposition pour le financement des programmes de promotion et de développement du sport. Les objectifs communs poursuivis par les fédérations, les CNO et les pouvoirs publics favorisent la collaboration sur le territoire national.

Le marché des médailles olympiques, en pleine croissance, est une incitation à la diversification. Tous les sports étant mis sur le devant de la scène, les disciplines mineures obtiennent ainsi une vitrine et la possibilité de faire parler d'elles dans les médias. Pour les athlètes, les Jeux représentent une opportunité unique de se positionner sur le marché du sport: une victoire olympique peut les aider à assurer des revenus sur l'ensemble de leur carrière, tout en accélérant de plusieurs années le développement de leurs disciplines dans leurs pays respectifs.

La couverture médiatique des Jeux Olympiques est colossale. Pendant les seuls Jeux d'été de 2012 à Londres, 5000 heures de compétitions sportives ont été diffusées dans 220 pays (CIO, 2013b). Une telle attention mondiale ne peut être obtenue que par une concentration de différentes disciplines, le football faisant ici figure d'exception avec la Coupe du Monde. Ces plates-formes sont exploitées de manière intensive par les nations: le Brésil, par exemple, compte profiter des Jeux Olympiques 2016 à Rio de Janeiro pour améliorer son image et faire progresser son économie. «Nous allons investir 735 milliards de dollars dans la modernisation de notre pays», a ainsi annoncé le ministre de l'Economie brésilien Miguel Jorge. Ces fonds seront consacrés à la construction de nouveaux logements, au développement du secteur des matières premières, à la modernisation du réseau ferroviaire et à l'approvisionnement en énergie. Le Brésil investit plus de 90 milliards de dollars dans les stades et les infrastructures en vue de la Coupe du Monde 2014 et des Jeux Olympiques 2016 (Fasse & Höpner, 2010).

Le programme des Jeux est fixé par le CIO lors du Congrès olympique. Pour prendre ces décisions, les membres votants privilégient souvent les possibilités offertes par le grand marché télévisuel américain, des considérations politiques entrant aussi certainement en ligne de compte. Le potentiel de croissance et les possibilités de redistribution au niveau national sont généralement un peu surévalués, ces effets positifs dépendant uniquement de la bonne exploitation des effets de synergie et de transfert.

Potentiels de croissance ou effets de synergie pour la réussite de la Suisse

Dans le cadre de sa possible candidature pour l'organisation des Jeux Olympiques d'hiver 2022 dans le canton des Grisons, la Suisse a défini différents scénarios pour savoir comment utiliser les forces dégagées par cet événement dans un but de promotion du sport sur le plan national. Après le rejet de la candidature par le peuple grison, il s'agit maintenant d'en tirer les enseignements nécessaires et de les appliquer dans la vie sportive du pays.

Le sport suisse doit apprendre à exploiter de manière plus systématique les effets des grandes manifestations, tant récurrentes qu'uniques. La priorité doit être donnée aux potentiels de financement et de marketing: une présence collective sur le marché peut-elle avoir un effet positif? Est-il plus facile d'occuper conjointement les nouveaux canaux de commercialisation? Les différents organisateurs ont peut-être intérêt à se regrouper pour obtenir des droits télévisuels et des budgets publicitaires lucratifs au niveau international. Le défi consiste à faciliter l'accès aux moyens financiers.

Il devrait être à la fois plus rapide et plus simple d'exploiter les synergies dans le domaine de la promotion du sport. Le système de financement suisse repose notamment sur la classification des disciplines sportives par la SOA. Mise en place au fil du temps, elle ne contient aucune incitation à la collaboration et aux échanges entre les fédérations et les disciplines sportives concurrentes. Ainsi, au lieu de rechercher des interfaces pour échanger du matériel et du savoir-faire, d'essayer de comparer les cycles d'entraînement et de trouver des similitudes en termes de marketing, de structure des compétitions ou encore d'infrastructures, les différentes disciplines se mettent en concurrence entre elles et essaient de débaucher les talents, les entraîneurs et les ressources.

La promotion ciblée, toutes disciplines confondues, dans le cadre des projets olympiques constituerait une façon de renforcer l'exploitation du potentiel de synergie. Une coopération entre les sports traditionnellement bien implantés et les nouvelles disciplines rapportant beaucoup de médailles permettrait d'accélérer le développement de ces dernières. On pourrait ainsi créer des dynamiques, certes parfois problématiques pour les disciplines traditionnelles, mais qui pourraient envoyer des signaux forts aux sponsors, organisateurs, centres d'entraînement et fédérations. La compétition entre les disciplines serait ainsi portée à un autre niveau, celui des groupes de disciplines sportives. Le système dans son ensemble gagnerait en efficacité dans la course aux médailles olympiques.

11. Perspectives pour la nation sportive suisse

Le nombre de médailles remportées aux Jeux Olympiques est une valeur cible importante pour le système sportif suisse. Entre 1988 et 2012, les objectifs fixés n'ont pas toujours été atteints. Et la démarche manifestement stratégique adoptée par les autres nations laisse penser que les médailles seront toujours plus difficiles à gagner. En tant que nation sportive, la Suisse a tout intérêt à rechercher des pistes d'amélioration.

Classement des domaines d'encouragement par les entraîneurs, les athlètes et les chefs du sport d'élite

Le présent rapport expose diverses réflexions à ce sujet, étudie les potentiels d'amélioration et soumet de premières propositions rudimentaires, selon une structure inspirée du modèle SPLISS élaboré par De Bosscher *et al.* (2008). Ce modèle distingue, au sein d'un système national de sport d'élite, dix domaines ayant un impact sur la réussite internationale (voir le chapitre 12). Pour classer par ordre de priorité les domaines d'action, le mieux est de commencer par interroger les personnes directement concernées. L'enquête portait sur l'importance des différents domaines et sur leur potentiel d'amélioration.

Figure 11.1: Importance des domaines d'encouragement pour la réussite internationale d'un pays, selon les athlètes, les entraîneurs et les chefs du sport d'élite

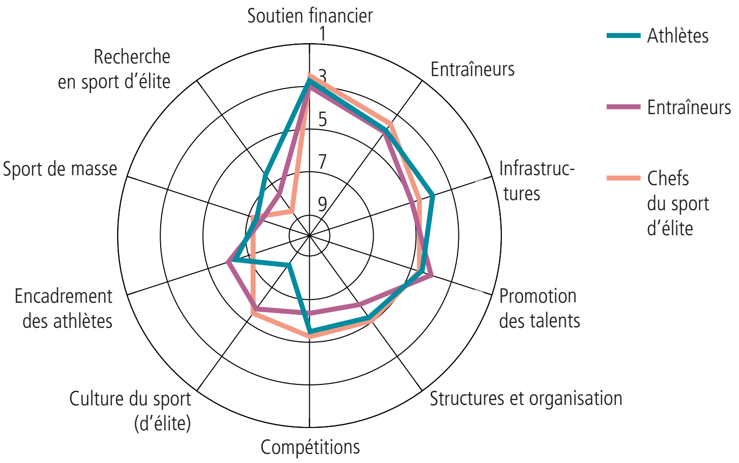

Source: SPLISS-CH 2011, n = 774 athlètes/366 entraîneurs/41 chefs du sport d'élite

La figure 11.1 fait apparaître le résultat suivant: le domaine «Soutien financier» est considéré comme celui qui influence le plus la réussite sportive internationale d'un pays. Il est suivi par les domaines «Entraîneurs», «Infrastructures», «Promotion des talents» et «Structures et organisation» nationales. Les domaines «Culture du sport (d'élite)», «Encadrement des athlètes» et «Recherche en sport d'élite» présentent les écarts les plus importants entre les trois groupes de personnes interrogées. Les athlètes, par exemple, accordent à la recherche dans le domaine du sport d'élite une plus grande importance que les entraîneurs et les chefs du sport d'élite. La culture du sport (d'élite), en revanche, est jugée moins importante par les athlètes.

Figure 11.2: Potentiel d'amélioration des domaines d'encouragement pour la réussite internationale d'un pays, selon les athlètes, les entraîneurs et les chefs du sport d'élite

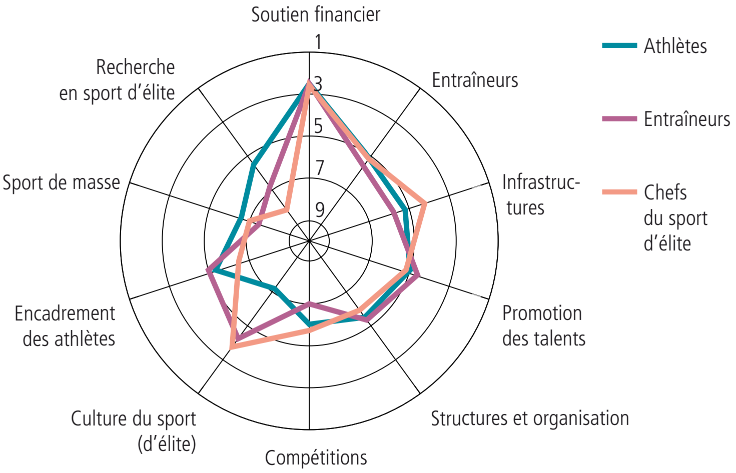

Source: SPLISS-CH 2011, n = 774 athlètes/364 entraîneurs/41 chefs du sport d'élite

Les athlètes, les entraîneurs et les chefs du sport d'élite ont également été interrogés sur le potentiel d'amélioration des différents domaines. Les résultats sont représentés par la figure 11.2. Les athlètes placent en tête les domaines «Soutien financier» et «Promotion des talents», tandis que pour les entraîneurs, le plus grand besoin d'action se situe dans ces deux mêmes domaines ainsi que dans «Culture du sport (d'élite)». Ce dernier domaine divise les entraîneurs: plus de 25 % d'entre eux le considèrent comme «très important», et la même proportion le juge «pas important du tout». Dans ce contexte, la question suivante se pose: est-ce la culture du sport (d'élite) qui influence positivement les autres domaines ou est-ce l'inverse?

Pistes de réflexion sur l'organisation du système du sport d'élite

Dans l'étude SPLISS-CH 2011, le point de vue des principaux acteurs du système du sport d'élite (athlètes, entraîneurs et chefs du sport d'élite) est comparé de manière approfondie avec les résultats d'autres analyses. L'inventaire du sport d'élite, les entretiens avec les experts et, pour finir, la comparaison internationale permettent ainsi de tirer des conclusions plus précises sur le potentiel d'amélioration du système:

- Le marché des médailles est en pleine croissance, mais aussi toujours plus convoité. Les nations sportives, à l'instar des entreprises dans le secteur économique, se voient contraintes d'adopter une démarche stratégique pour s'assurer des parts de marché. Le potentiel de médailles doit ainsi être développé dans des disciplines sélectionnées (voir le chapitre 2).
- Pour espérer s'imposer dans des compétitions internationales, les athlètes doivent s'entraîner pendant 10 ans ou 10 000 heures. La stabilité du contexte d'encouragement du sport joue un rôle central pour les sportifs de haut niveau, en garantissant une sécurité indispensable pour réduire le risque des investissements. Avec leurs structures, les fédérations assument en effet des risques importants. Les multiples imbrications – horizontales et verticales – du système d'encouragement suisse sont un gage de stabilité: l'émulation entre les disciplines, les institutions et les différents niveaux de hiérarchie permet d'assurer une certaine efficacité, dont la Suisse a besoin pour exister sur la scène sportive internationale (voir le chapitre 3).
- La nation sportive suisse poursuit les objectifs de médailles suivants: se classer dans le top 8 des nations au sein du tableau des médailles des Jeux Olympiques d'hiver, et dans le top 25 pour les Jeux d'été. Si elle mise avant tout sur ses disciplines traditionnellement fortes, elle a remporté entre 1988 et 2012 de très nombreuses médailles dans les nouveaux sports et nouvelles disciplines. Ce bilan illustre l'une des forces du sport suisse: le rôle de l'initiative privée. Malgré les différentes mesures prises par les organes de pilotage subsidiaires (Swiss Olympic, OFSPO, etc.) dans le but d'optimiser les résultats sportifs, la Suisse doit continuer à compter sur les initiatives privées (voir le chapitre 4).
- Pour les enfants et les jeunes, l'environnement immédiat est déterminant dans le choix d'un sport et la spécialisation dans une seule discipline. Les parents et les entraîneurs des clubs jouent ainsi un rôle de piliers dans la relève du sport d'élite. La sensibilisation de ces personnes aux problématiques du sport d'élite constitue par conséquent un facteur important pour la réussite durable d'une nation sportive (voir le chapitre 5).
- Le moment opportun pour lancer les mesures d'encouragement par les associations régionales ou les fédérations nationales diffère selon les disciplines. Les offres ont généralement pour conséquence d'accroître le volume d'entraînement, ce qui est bien sûr extrêmement important. L'entraînement moderne est en effet très complexe: récupération, école et formation doivent être bien gérées. Lorsque les jeunes athlètes ne bénéficient pas d'un encouragement suffisant sur le long terme, ils ne parviennent souvent pas, par la suite, à développer tout leur potentiel. Et ils perdent un temps précieux. Les erreurs commises dans l'encouragement précoce des

sportifs se paient lorsqu'ils sont en âge de concourir avec l'élite. Ce principe concerne également la Suisse, c'est pourquoi il est nécessaire d'améliorer le soutien destiné aux sportifs de la relève (voir le chapitre 6).

- La sélection par les fédérations et l'attribution des cartes par Swiss Olympic ont été nettement améliorées. Seule la sélection annuelle continue de poser problème: censée stimuler et discipliner les athlètes chaque année, elle s'avère peu judicieuse pour la planification à long terme de leur préparation. Les systèmes d'encouragement des écoles de sport et de l'armée, basés sur une période donnée, sont peut-être plus efficaces en la matière (voir le chapitre 6).
- Les athlètes doivent être considérés comme des entrepreneurs et leur carrière doit être bâtie en conséquence. Même si la première tentative de se lancer dans le sport d'élite professionnel a échoué, la Suisse, en tant que nation sportive, a tout intérêt à mieux exploiter et reconnaître les liens entre les prestations de formation du sport d'élite et la vie professionnelle. C'est la seule solution pour que les décideurs du monde sportif puissent appeler de leurs vœux la professionnalisation des athlètes (voir le chapitre 6).
- Le métier d'entraîneur est important. Ce champ professionnel et sa valorisation, qui passe notamment par les salaires, doivent encore être consolidés. La croissance rapide du sport d'élite – un domaine complexe et hétéroclite – exige davantage d'offres de formation interdisciplinaires. Une collaboration entre spécialistes autour du thème du sport permettrait de faire avancer la professionnalisation à tous les niveaux (voir le chapitre 7).
- «Nous arrive-t-il aussi de gagner des médailles dans des disciplines où l'on n'est pas assis?», s'interrogeait autrefois un célèbre ministre des sports. Ce qui soulève la question suivante: pourquoi avons-nous presque honte des médailles remportées dans les disciplines où le matériel joue un grand rôle? La recherche etle développement sont des facteurs essentiels pour la réussite internationale. La puissance de la Suisse dans ce domaine peut être utilisée à bon escient, mais il faudrait avant tout mettre ce savoir à la disposition du sport d'élite, et cela coûte cher (voir le chapitre 8).
- Les centres d'entraînement recèlent un potentiel évident. Véritable concentration de compétences pour les différents sports, ils représentent un facteur de réussite capital. Un encouragement complet des athlètes et le développement des disciplines passent nécessairement par une mise en relation efficace des divers acteurs du sport. En outre, l'harmonisation des procédures et le renforcement des incitations financières peuvent aider à tisser en Suisse un réseau dense de centres de compétences (voir le chapitre 9).
- Les grandes manifestations sportives constituent des piliers incontournables du financement et de la promotion du sport d'élite. Le regroupement des disciplines entraîne non seulement la création d'un potentiel commercial supplémentaire, mais aussi la libération d'effets de synergie en vue d'un encouragement systématique. En effet, des approches marketing individuelles, la hiérarchisation des disciplines et le soutien financier direct envoient des signaux non négligeables au marché. Le système pourrait donc être renforcé et mieux contribuer à atteindre l'objectif de médailles si la recherche et la distribution des moyens financiers s'effectuaient davantage au niveau des groupes de disciplines sportives (voir le chapitre 10).

La seule constante que l'on peut observer dans le sport d'élite suisse est un perpétuel changement. L'évolution des 15 dernières années, retracée dans l'annexe 1, est impressionnante. Les pistes d'optimisation présentées ci-dessus constituent une liste déjà longue. Face aux mutations rapides auxquelles est confronté le secteur, les prochaines séries de réformes devront être mûrement réfléchies.

Les objectifs de la Suisse ne se limitent pas aux médailles
L'objectif de médailles sert à évaluer le système national du sport d'élite par rapport aux autres pays. Mais en soutenant les fédérations, la Suisse entend aussi poursuivre bien d'autres objectifs: outre le succès international de la discipline et la promotion de la relève, c'est l'importance de chacun de ces sports au sein de la société qui est en jeu.

Le marché des médailles est souvent abordé de manière très agressive par les pays. Différentes stratégies sont connues au niveau international pour se faire une place dans la course aux médailles: on peut citer notamment les systèmes de détection des talents et d'encouragement, qui dirigent de façon ciblée les enfants et les jeunes vers des disciplines précises; les programmes d'encouragement spécifiques pour les femmes et destinés à des sports où la concurrence est moindre; la recherche internationale d'émigrés et enfin la naturalisation simplifiée des athlètes d'élite. D'autres possibilités existent aussi, comme la modification des règlements et les restrictions de qualification en vue d'assurer un avantage concurrentiel au pays concerné ou de créer des entraves à la participation. La Charte olympique résulte d'une volonté de sagesse, comme en témoigne la règle 57 sur le tableau d'honneur édicté par le CIO. Chaque nation doit décider elle-même de placer certaines valeurs au-dessus de la simple obtention de médailles. C'est donc à la Suisse, en tant que nation sportive, de déterminer quelles médailles brillent plus que les autres à ses yeux.

Tableau 11.1: Financement par Swiss Olympic et l'OFSPO, taille de la délégation, nombre d'épreuves et nombre de médailles remportées – comparaison entre les sports soutenus pour les Jeux Olympiques d'été

	Financement 2010	Délégation 2012	Epreuves 2012	Médailles 2004	Médailles 2008	Médailles 2012
Gymnastique (disciplines olymp.)	14 %	2	18	–	–	–
Natation (disciplines olymp.)	12 %	9	46	–	–	–
Tennis	8 %	2	5	–	1	1
Cyclisme (disciplines olymp.)	8 %	11	18	2	4	1
Athlétisme	7 %	14	47	–	–	–
Volleyball (les deux disciplines olymp.)	6 %	6	4	1	–	–
Triathlon	6 %	4	2	1	–	1
Football*	**6 %	18	2	–	–	–
Autres	33 %	35	160	1	2	1
Total	100 %	102	302	5	7	4

* L'équipe masculine s'est qualifiée en 2012. L'équipe nationale féminine ne s'est pas qualifiée en 2004, 2008 et 2012.

** Le football perçoit 4,4 millions de francs suisses directement des loteries cantonales. Cette somme dépasse très nettement l'aide accordée par Swiss Olympic et par l'OFSPO. Elle n'est cependant pas prise en compte dans le tableau 11.1.

Source: tableau établi par les auteurs d'après les données de Swiss Olympic (2010c, 2011c, 2012b); OFSPO (2012); Stamm & Lamprecht (2012)

Tableau 11.2: Financement par Swiss Olympic et l'OFSPO, taille de la délégation, nombre d'épreuves et nombre de médailles remportées – comparaison entre les sports soutenus pour les Jeux Olympiques d'hiver

	Financement 2010	Délégation 2010	Epreuves 2010	Médailles 2002	Médailles 2006	Médailles 2010
Ski alpin	33 %	15	10	1	3	3
Hockey sur glace*	**17 %	44	2	–	–	–
Snowboard	12 %	16	6	2	4	1
Ski de fond	9 %	12	12	1	–	1
Curling	9 %	10	2	2	1	1
Ski acrobatique	4 %	14	6	–	1	1
Saut à ski	4 %	2	3	2	–	2
Bob	4 %	13	3	2	2	–
Autres	9 %	20	42	1	3	–
Total	100 %	146	86	11	14	9

* L'équipe masculine s'est qualifiée en 2002, 2006 et 2010. L'équipe féminine s'est qualifiée en 2006 et 2010.

** Le hockey sur glace perçoit 2,2 millions de francs suisses directement des loteries cantonales. Cette somme dépasse l'aide accordée par Swiss Olympic et par l'OFSPO. Elle n'est cependant pas prise en compte dans le tableau 11.2.

Source: tableau établi par les auteurs d'après les données de Swiss Olympic (2010c, 2011c, 2012b); OFSPO (2012); Stamm & Lamprecht (2012)

Pour illustrer la façon dont le système d'encouragement fonctionne dans les détails, les disciplines qui bénéficient du soutien financier le plus important sont à nouveau comparées. L'ordre de classement correspond aux priorités définies pour le financement par Swiss Olympic et par l'OFSPO, et renseigne ainsi sur les sports prioritaires au niveau national. La colonne d'à côté indique la taille de la délégation olympique de chaque discipline, c'est-à-dire le nombre d'athlètes qui concourent pour atteindre les objectifs fixés. Le tableau présente ensuite le nombre d'épreuves inscrites au programme olympique dans la discipline concernée, et enfin la réussite obtenue lors des trois dernières éditions des Jeux Olympiques, à savoir le nombre de médailles gagnées.

Le tableau 11.1, qui concerne les sports d'été, affiche un bilan de réussite – sur la base du nombre d'athlètes qualifiés (composition de la délégation) et des médailles remportées – qui ne coïncide que de manière limitée avec l'aide subsidiaire accordée par Swiss Olympic et par l'OFSPO. On peut raisonnablement penser que la concurrence internationale est importante dans ces disciplines et/ou que les ressources disponibles (en ce qui concerne les facteurs déterminants pour la performance) sont insuffisantes. Peut-être même que des mesures s'imposent pour accroître l'efficacité du soutien accordé. Mais il est également possible que la réussite ne se profile que sur le long terme. La décision de soutenir ou non ces disciplines sportives ne peut donc être motivée uniquement par le critère des médailles.

La comparaison entre la taille des délégations et le nombre d'épreuves fait apparaître, par exemple en natation et en gymnastique, qu'une grande partie des épreuves des Jeux Olympiques d'été peuvent être disputées par quelques athlètes seulement, c'est-à-dire qu'un petit nombre de sportifs convoitent une proportion non négligeable des médailles. En cyclisme, c'est l'inverse qui se produit: les athlètes sont nombreux à se qualifier pour une poignée d'épreuves. Les données présentées ici ne permettent pas de savoir s'il s'agit d'une décision stratégique délibérée.

Lorsque, comme en 2012, la sélection olympique de football se qualifie pour les Jeux d'été, beaucoup de sportifs concourent pour remporter une seule médaille. Mais le rapport entre les moyens engagés et l'objectif poursuivi ne revêt pas la même priorité en raison de la place particulière qu'occupent les Jeux Olympiques par rapport à la Coupe du Monde et à l'Euro. En effet, les meilleurs joueurs du monde ne participent pas au tournoi olympique de football.

Pour les Jeux Olympiques d'hiver, la même analyse (voir le tableau 11.2) révèle dans quelles conditions de pression financière la Suisse parvient à remporter des médailles en ski alpin, mais aussi en snowboard et en curling. Le saut à ski démontre qu'un athlète d'exception peut contribuer de manière importante à atteindre l'objectif de médailles. En comparant, pour les disciplines d'hiver, la taille de la délégation au nombre d'épreuves, on remarque qu'en ski de fond, un petit nombre d'athlètes doivent remporter un grand nombre de médailles. Ce phénomène est encore plus évident en saut à ski. L'édition des Jeux Olympiques de 2014 à Sotchi permettra de savoir si cette stratégie s'avère payante sur le long terme.

Les sports d'équipe ont naturellement une influence majeure sur la taille des délégations. Néanmoins, lorsque les équipes féminines et masculines parviennent à se qualifier pour un tournoi olympique, cela profite à toutes les autres disciplines grâce à un plus large contingent de personnel d'encadrement. En outre, si l'équipe nationale suisse de hockey sur glace, par exemple, venait à remporter une médaille, la situation serait la suivante: une telle réussite n'aurait qu'un impact limité dans le tableau des médailles du fait du grand nombre d'athlètes concerné, mais elle aurait en revanche un retentissement extrêmement fort dans l'opinion publique.

L'analyse critique du sport d'élite suisse a montré que de très bonnes choses sont faites dans ce domaine. Toutefois, la Suisse en tant que nation sportive devra veiller encore davantage à l'avenir à agir de la manière la plus efficace possible. La concentration des mesures sur la base de la réussite obtenue apportera des améliorations, et l'optimisation des processus contribue également à la réalisation de l'objectif de médailles. La discussion s'impose de manière plus urgente pour les sports d'été que pour ceux d'hiver.

Disposer de moyens supplémentaires est, avec un degré de professionnalisation croissant, une condition *sine qua non* pour réussir au niveau international. Les athlètes, les entraîneurs et les chefs du sport d'élite sont unanimes sur la question. La focalisation sur certaines disciplines se fait généralement au détriment de la diversité ou de la durabilité. Et le statut professionnel des uns n'est possible que grâce à l'activité bénévole des autres. Les moments de bonheur des médaillés et l'enthousiasme collectif suscité par les Jeux Olympiques devraient toutefois être une juste récompense pour ces efforts.

12. Concept d'étude et méthode

L'état des lieux du système suisse du sport d'élite 2011 a été réalisé dans le cadre de l'étude comparative internationale des systèmes nationaux de sport d'élite (SPLISS 2011-2012). SPLISS signifie «Sport Policy factors Leading to International Sporting Success». Cette étude internationale examine les éléments des systèmes nationaux de sport d'élite qui contribuent à créer des conditions propices au succès des athlètes et des équipes.

Objectif de l'étude sur la Suisse

L'étude SPLISS-CH 2011 (SPLISS-CH 2011) vise, au moyen d'un inventaire systématique, à accroître la transparence du système suisse du sport d'élite et à mieux comprendre son mode de fonctionnement. Elle peut ainsi servir de base aux décideurs publics (Confédération, cantons et communes) et privés (Swiss Olympic, fédérations et clubs) pour améliorer l'efficience et l'efficacité du système d'encouragement. L'étude SPLISS est conçue comme une étude comparative internationale, à laquelle participent au total 15 pays. Les résultats nationaux peuvent ainsi être comparés entre les pays participants.

Organisation du projet

L'étude SPLISS internationale est dirigée par un consortium de recherche, qui se compose des personnes suivantes: V. De Bosscher (Vrije Universiteit Brussel), S. Shibli (Sheffield Hallam University), H. Westerbeek (Victoria University Melbourne) et M. van Bottenburg, (Universiteit Utrecht).

La section Sport et société de la HEFSM a procédé en 2011-2012 à la collecte des données, à leur traitement et à leur interprétation pour la Suisse. Un groupe national d'accompagnement, réunissant A. Bürgi (Formation des entraîneurs Suisse), C. Hollenstein (Swiss Olympic), H.P. Stamm (Observatoire du sport) et P.A. Weber (OFSPO), l'a épaulée pour le processus de recueil et de validation des données.

Le présent rapport expose quelques résultats révélateurs extraits de l'inventaire 2011 du système suisse du sport d'élite et il résume les conclusions tirées de cette enquête exhaustive. De premiers résultats de l'étude internationale sont également présentés dans les commentaires.

Modèle de l'étude SPLISS-CH 2011

L'étude SPLISS-CH 2011 repose sur le modèle d'input-throughput-output de De Bosscher *et al.* (2008). Par «input», on entend les ressources financières injectées au niveau national. L'output est quant à lui mesuré à l'aune des médailles olympiques remportées, hors Jeux Paralympiques. Le throughput inclut les différentes étapes du parcours d'athlète ainsi que des prestations de soutien décisives. Le modèle met en relation le financement (input), les services de soutien fournis aux athlètes (throughput) et le succès international (output).

Le modèle montre quels sont les domaines d'un système national de sport d'élite déterminants pour la réussite internationale, domaines sur lesquels les décideurs chefs au niveau national peuvent influer. Le présent rapport analyse ce modèle dans les différents chapitres. Les domaines du système du sport d'élite ont été évalués à l'aide de 136 facteurs critiques de succès, sur la base de 219 questions principales et d'un grand nombre de sous-questions. Ces questions ont été élaborées par le consortium SPLISS international, tandis que les réponses correspondantes ont été recueillies et complétées par les partenaires de recherche nationaux.

Collecte des données

Deux instruments ont été employés pour collecter les données relatives au système suisse du sport d'élite: une liste de 219 questions principales sur la situation actuelle, et une liste de 60 à 70 questions pour l'interrogation des athlètes, des entraîneurs et des chefs du sport d'élite au sein des fédérations sportives nationales.

L'inventaire s'est appuyé sur des documents et publications des principales parties prenantes nationales du système suisse du sport d'élite, en s'inspirant également d'ouvrages de référence (Digel *et al.*, 2006; Emrich & Güllich, 2005; Houlihan & Green, 2008; Kempf, 2004) et d'études scientifiques sur des sous-domaines du système sportif suisse (Heinzmann, 2007; Schafer, 2011). En complément, des interviews constitués de questions ouvertes ou fermées ont été effectués auprès de 68 experts: 52 d'entre eux ont été interrogés sur leur domaine de spécialité, et 16 sur les atouts et les faiblesses du système suisse du sport d'élite dans l'un de ces domaines. L'annexe 4 indique le nom de tous les experts consultés. Dans certains domaines (p. ex. l'infrastructure), les questions ont été approfondies et affinées.

Tableau 12.1: Echantillon et classification des personnes interrogées

	Echantillon			Classification des personnes interrogées			
	Interrogés	Ayant répondu	Taux de répondants	JO d'été	JO d'hiver	Hors JO	Inclassables
Chefs du sport d'élite	58	48	83 %	26	11	9	2
Entraîneurs	682	464	68 %	237	135	55	37
Athlètes	*959	897	94 %	411	297	189	–

*nombre approximatif

Source: SPLISS-CH 2011

Tableau 12.2: Information sur les athlètes et entraîneurs ayant répondu

	Moyenne	Pourcentages				
	Age	Femmes	Etrangers	Sports d'équipe	Classification 1 (Swiss Olympic)	Niveau élite
Entraîneurs	43 ans	15 %	24 %	20 %	56 %	40 %
Athlètes	25 ans	42 %	0 %	22 %	43 %	88 %

Source: SPLISS-CH 2011

Les questionnaires élaborés par le consortium SPLISS ont été adaptés au système suisse, traduits et complétés par des questions spécifiques. L'enquête réalisée auprès des athlètes comportait 63 questions approfondies par des sous-questions. Elle a été effectuée sur place au moyen d'i-pads ou en ligne à partir d'un lien transmis par courriel. L'enquête destinée aux athlètes comprenait 70 questions avec des sous-questions, et celle pour les chefs du sport d'élite 60 questions avec des sous-questions. Tous deux se sont déroulés en ligne.

L'échantillon d'athlètes a été constitué parmi les sportifs possédant une «Swiss Olympic Card» catégorie or, argent ou bronze ou une Swiss Olympic Talents Card catégorie internationale de 2010. Comme certaines fédérations de sports représentés aux JO (handball, basketball, etc.) ne comptaient pas d'athlètes remplissant ce critère, l'échantillon a été complété en conséquence afin de représenter fidèlement la situation du sport d'élite suisse. L'échantillon d'entraîneurs regroupait des personnes titulaires de la «Swiss Olympic Card» entraîneur 2010 ainsi que des entraîneurs des niveaux 2 et 3 de promotion de la relève (OFSPO, 2011e). Enfin, l'échantillon des chefs du sport d'élite était composé des chefs des fédérations prioritaires (classification Swiss Olympic 1-3). Les chefs des diverses disciplines des fédérations sélectionnées ont également répondu à l'enquête. Le tableau 12.1 montre la taille des enquêtes et le bon taux de réponse obtenu. Le tableau 12.2 fournit des informations complémentaires sur les athlètes et entraîneurs interrogés.

Aucune pondération différenciée n'a été appliquée pour l'analyse des données. Celles-ci permettent d'émettre des affirmations solides sur le sport d'élite suisse du point de vue des personnes directement concernées.

Validation

Afin d'éclaircir les opinions parfois contradictoires, le concept de l'enquête et les résultats des entretiens avec les experts ont été discutés lors de deux ateliers nationaux regroupant chacun 21 participants. Un atelier de comparaison internationale rassemblant des représentants scientifiques de pays comparables (Danemark, Finlande, France et Pays-Bas) a également été organisé pour comprendre les résultats internationaux. Les noms des participants figurent eux aussi à l'annexe 4. Ces différents ateliers ont montré à quel point il était complexe et délicat d'établir une représentation structurée et une vision homogène des systèmes de sport d'élite. A cela s'ajoute le fait qu'en Suisse, le système de sport d'élite, déjà complexe, fait sans cesse l'objet de modifications.

Annexe

Annexe 1: Grandes étapes de l'encouragement du système suisse du sport d'élite

1996	Adoption de la Conception des installations sportives d'importance nationale (CISIN) par le Conseil fédéral.
1997	Fusion du Comité olympique suisse (COS), du Comité national pour le sport d'élite (CNSE) et de l'Association suisse du sport (ASS) pour donner naissance à la Fédération olympique suisse (FOS), devenue Swiss Olympic Association (SOA) en 2001.<br>Adoption de la base légale «Ordonnance réglant l'engagement de moyens militaires dans le cadre d'activités civiles et d'activités hors du service (OEMC)».
1998	Création et intégration de l'Office fédéral du sport (OFSPO) suite au réaménagement du Département fédéral de la défense, de la protection de la population et des sports (DDPS).<br>Transformation de l'Ecole fédérale de sport de Macolin en Haute école fédérale de sport de Macolin (aujourd'hui HEFSM) et rattachement à l'OFSPO.
1999	Publication de l'ouvrage «Les 12 éléments de la réussite. Concept de base de Swiss Olympic Association pour l'encouragement de la relève» par la FOS.
2000	Adoption, par l'ensemble du Conseil fédéral, du «Concept du Conseil fédéral pour une politique du sport en Suisse».<br>Instauration de la grille de classification des spécialités sportives par la FOS.<br>Création de SwissTopSport (STS), l'association des plus importantes manifestations sportives suisses.
2001	Préparation de la mise en œuvre concrète du concept pour une politique du sport et des mesures politiques et financières y afférentes.<br>Lancement du camp «Tous les talents à Tenero» comme mesure de mise en œuvre dans le domaine de l'encouragement de la relève.
2002-2003	Lancement du programme Coachs J+S et formation de 10 000 coachs J+S au sein des clubs.
2003	Championnats du monde de ski alpin à Saint-Moritz.<br>Introduction de la promotion de la relève J+S et augmentations successives des aides versées aux fédérations et aux clubs. Octroi de ces aides en fonction des qualifications des entraîneurs. Reconnaissance professionnelle du métier d'entraîneur au moyen d'un brevet fédéral délivré par l'Office fédéral de la formation professionnelle et de la technologie (OFFT, aujourd'hui Secrétariat d'Etat à la formation, à la recherche et à l'innovation [SEFRI]).<br>Augmentation du budget dans le plan directeur de recherche 2004-2007 de l'OFSPO.
2004	Instauration par Swiss Olympic d'une comptabilité unique pour les fédérations sportives nationales (Swiss Sport GAAP).<br>Label Swiss Olympic décerné pour la première fois à des écoles.
2005	Organisation d'une conférence extraordinaire par Swiss Olympic et l'OFSPO sur le thème de la promotion de la relève afin d'éviter que l'échec de Bormio ne se reproduise.<br>Instauration de la «Swiss Olympic Talents Card» (catégories nationale et régionale).
2006	Introduction de la mesure d'encouragement «Swiss Olympic Top Athletes».<br>Première réalisation des cours de répétition en vue des JO et des championnats du monde et projet pilote avec 14 postes pour militaires contractuels sportifs d'élite (2006-2010); 18 postes à compter de 2010.
2007	Signature d'un accord de coopération et d'une convention de prestations entre Swiss Olympic et l'OFSPO.<br>Projets pilotes J+S-Kids (pour les enfants de 5 à 10 ans) dans le cadre de l'EURO 2008.
2008	EURO 2008 en Suisse et en Autriche.<br>Assemblée constituante de la «Société suisse des sciences du sport (4S)».
2009	Création des bases pour l'instruction au sport de l'armée via le nouveau règlement «Sport dans l'armée».
2010	Entrée en vigueur du «Concept du sport d'élite suisse – Continuité de la promotion relève-élite».<br>Prise de position officielle du Conseil fédéral sur la candidature suisse aux Jeux Olympiques d'hiver 2022.<br>Adoption, par le Conseil fédéral, du contre-projet direct à l'initiative populaire fédérale de 2009 intitulée «Pour des jeux d'argent au service du bien commun» afin de consacrer au sport les recettes issues des jeux de hasard.<br>Mise en œuvre de l'outil de sélection PISTE pour le repérage, la sélection et l'encouragement des jeunes talents.
2010 - 2011	Elaboration du concept de la Confédération pour les manifestations sportives internationales organisées en Suisse.
2011	Elaboration par les fédérations sportives d'un concept de la relève servant de support supplémentaire à Swiss Olympic pour mettre au point un concept stratégique général.<br>Adoption, par le Parlement, de la loi fédérale sur l'encouragement du sport et de l'activité physique (loi fédérale sur l'encouragement du sport), fruit de la révision de la loi fédérale encourageant la gymnastique et les sports.
2012	Adaptation du système de classification des spécialités sportives par Swiss Olympic, avec une meilleure prise en compte de l'importance du sport concerné au sein de la société.<br>Entrée en vigueur de la nouvelle loi sur l'encouragement du sport et des ordonnances afférentes émises par le Conseil fédéral.<br>Lancement d'un concept national du sport d'élite par l'OFSPO.

Annexe 2: Evolution des épreuves des Jeux Olympiques d'été entre 1988 et 2012 (sans les sports de démonstration)

		Séoul 1988	Barcelone 1992	Atlanta 1996	Sydney 2000	Athènes 2004	Pékin 2008	Londres 2012	Total
Principales disciplines trad.	Athlétisme	42	43	44	46	46	47	47	315
	Natation	31	31	32	32	32	34	34	226
	Tir	13	13	15	17	17	15	15	105
	Gymnastique artistique	14	14	14	14	14	14	14	98
	Aviron	14	14	14	14	14	14	14	98
	Judo	7	14	14	14	14	14	14	91
	Haltérophilie	10	10	10	15	15	15	15	90
	Canoë-kayak: régate	12	12	12	12	12	12	12	84
Autres disciplines trad.	Boxe	12	12	12	12	11	11	13	83
	Lutte libre	10	10	10	8	11	11	11	71
	Voile	8	10	10	11	11	11	10	71
	Escrime	8	8	10	10	10	10	10	66
	Cyclisme sur piste	6	7	8	12	12	10	10	65
	Lutte gréco-romaine	10	10	10	8	7	7	7	59
	Plongeon	4	4	4	8	8	8	8	44
	Tennis	4	4	4	4	4	4	5	29
	Tir à l'arc	4	4	4	4	4	4	4	28
	Tennis de table	4	4	4	4	4	4	4	28
	Cyclisme sur route	3	3	4	4	4	4	4	26
	Basketball	2	2	2	2	2	2	2	14
	Equitation: dressage	2	2	2	2	2	2	2	14
	Equitation: concours complet	2	2	2	2	2	2	2	14
	Equitation: saut	2	2	2	2	2	2	2	14
	Handball	2	2	2	2	2	2	2	14
	Hockey sur gazon	2	2	2	2	2	2	2	14
	Volleyball	2	2	2	2	2	2	2	14
	Pentathlon moderne	2	2	1	2	2	2	2	13
	Natation synchronisée	2	2	1	2	2	2	2	13
	Football	1	1	2	2	2	2	2	12
	Gymnastique rythmique	1	1	2	2	2	2	2	12
	Water-polo	1	1	1	2	2	2	2	11
Nouvelles disciplines	Taekwondo				8	8	8	8	32
	Badminton		4	5	5	5	5	5	29
	Canoë-kayak: slalom		4	4	4	4	4	4	24
	Beach-volley			2	2	2	2	2	10
	Cyclisme: VTT			2	2	2	2	2	10
	Trampoline				2	2	2	2	8
	Triathlon				2	2	2	2	8
	Cyclisme: BMX						2	2	4
	Total*	237	256	269	298	299	300	302	

* Une épreuve de baseball a eu lieu entre 1992 et 2008 et une épreuve de softball a eu lieu entre 1996 et 2008.

Annexe 3: Evolution des épreuves des Jeux Olympiques d'hiver entre 1988 et 2014

		Calgary 1988	Albertville 1992	Lillehammer 1994	Nagano 1998	Salt Lake City 2002	Turin 2006	Vancouver 2010	Sotchi 2014	Total
Principales disciplines trad.	Ski de fond	8	10	10	10	12	12	12	12	86
	Patinage de vitesse	10	10	10	10	10	12	12	12	86
	Ski alpin	10	10	10	10	10	10	10	10	80
	Biathlon	3	6	6	6	8	10	10	11	60
	Patinage artistique	4	4	4	4	4	4	4	5	33
Autres disciplines trad.	Luge	3	3	3	3	3	3	3	4	25
	Saut à ski	3	3	3	3	3	3	3	4	25
	Bobsleigh	2	2	2	2	3	3	3	3	20
	Combiné nordique	2	2	2	2	3	3	3	3	20
	Hockey sur glace	1	1	1	2	2	2	2	2	13
Nouvelles disciplines	Patinage de vitesse – short track		4	6	6	8	8	8	8	48
	Ski acrobatique		2	4	4	4	4	6	10	34
	Snowboard				4	4	6	6	10	30
	Curling				2	2	2	2	2	10
	Skeleton					2	2	2	2	8
	Total	46	57	61	68	78	84	86	98	

Annexe 4: Liste des experts et des participants aux ateliers

Experts techniques

Balthasar, A. (Interface), Bär, P. (Conférence des répondants cantonaux du sport), Baumberger, M. (Swiss Olympic), Belz, Ch. (OFSPO), Berwert, A. (CTI), Birrer, H.J. (OFSPO), Brogle, J. (SRG SSR), Burch, T. (Swiss Olympic), Conrad, J. (Swiss Olympic), Conz, C. (OFSPO), Edmond, I. (ASF), Egli, D. (OFSPO), Fischer, A. (Swiss Tennis), Fischer, F. (OFSPO), Fuchslocher, J. (OFSPO), Gmür, E. (Administration fédérale des douanes), Gogoll, A. (OFSPO), Gygax, M. (OFSPO), Heer, Ch. (DDPS), Heini, R. (Swiss Olympic), Hollenstein, C. (Swiss Olympic), Kempf, H. (OFSPO), Kindle, A. (EPFZ), Lamprecht, M. (Lamprecht und Stamm), Lütolf, A. (OFSPO), Mäder, U. (OFSPO), Mahler, N. (OFSPO), Nützi, Ch. (OFSPO), Raemy, H. (OFSPO), Rauber, K. (Swiss Olympic), Rauch, W. (OFSPO), Ray, J.-C. (Swiss Olympic), Rechsteiner, D. (Swiss Olympic), Romann, M. (OFSPO), Rüdisüli, R. (OFSPO), Schneider, Ch. (RUAG), Schwendimann, M. (OFSPO), Schwizgebel, St. (OFSPO), Sottas, G. (Office fédéral des assurances sociales), Stopper, M. (OFSPO), Ulrich, W. (OFSPO), Ursprung, L. (OFSPO), Walther, U. (DDPS), Weber, P.-A. (OFSPO), Wernli, B. (DDPS), Werthmüller, L. (Swiss Olympic), Wiedmer, R. (Swisslos), Wiegand, D. (Lamprecht und Stamm), Wolf, M. (OFSPO), Wolfsperger, F. (SLF), Zahnd, S. (DDPS), Zehr, S. (DDPS)

Experts seniors

Babst, H. (Swiss Olympic), Conzelmann, A. (Université de Berne), Feller, M. (OFSPO/Candidature suisse aux Jeux Olympiques 2022), Gilli, G. (Swiss Olympic), Hanselmann, E. (ex-OFSPO), Hoppeler, H. (Commission fédérale du sport, groupe de recherche), Junod, T. (UEFA), Lichtsteiner, H. (VMI/SSMC), Marti, B. (OFSPO), Mengisen, W. (OFSPO), Müller, H.R. (Université de Berne), Nagel, S. (Université de Berne), Rütter, H. (Rütter + Partner), Stettler, J. (Université de Lucerne), von Mühlenen, T. (ex-OFSPO), Winkler, U. (Gymnase sportif de Davos)

Participants à l'atelier national sur la méthode en mars 2010 à Macolin

Belz, Ch. (OFSPO), Bürgi, A. (OFSPO), De Bosscher, V. (Vrije Universiteit Brussel), Fuchslocher, J. (OFSPO), Gnädinger, J. (OFSPO), Stamm, H. (Lamprecht und Stamm), Heyer, L. (Swiss Athletics), Hollenstein, C. (Swiss Olympic), Kempf, H. (OFSPO), Läuppi, P. (Swiss Olympic), Liess, S. (Swiss Olympic), Mahler, N. (OFSPO), Marti, B. (OFSPO), Romann, M. (OFSPO), Rüdisüli, R. (Swiss Olympic), Schafer, J. (VMI), Shibli, S. (Sheffield Hallam University), Stopper, M. (OFSPO), van Bottenburg, M. (Universiteit Utrecht), Weber, P.-A. (OFSPO), Wolf, M. (OFSPO)

Participants à l'atelier national en février/mars 2012 à Macolin

Bürgi, A. (OFSPO), De Bosscher, V. (Vrije Universiteit Brussel), Egli, D. (OFSPO), Fuchslocher, J. (OFSPO), Hässig, Ch. (OFSPO), Held, L. (OFSPO), Hollenstein, C. (Swiss Olympic), Jeanneret, T. (Amt für Volksschule und Sport Graubünden), Kempf, H. (OFSPO), Knäbel, P. (ASF), Lambert, T. (Commission des athlètes Swiss Olympic), Läuppi, P. (Swiss Ski), Mäder, U. (OFSPO), Mengisen, W. (OFSPO), Müller, L. (Swiss Olympic), Stöckli, R. (Swiss Olympic), Stopper, M. (OFSPO), van Bottenburg, M. (Universiteit Utrecht), Weber, A.Ch. (OFSPO), Weber, P.-A. (OFSPO), Wegmann, U. (House of Sport Uster)

Participants à l'atelier de comparaison internationale en février 2013 à Macolin

Bagdasarjanz, V. (OFSPO), Bürgi, A. (OFSPO), Dijk, B. (Universiteit Utrecht), Fuchslocher, J. (OFSPO), Hollenstein, C. (Swiss Olympic), Kempf, H. (OFSPO), Lämsä, J. (KIHU – Research Institute for Olympic Sports), Müller, L. (Swiss Olympic), Renaud, A. (OFSPO), Salmi, J. (Finnish Orienteering Federation), Stopper, M. (OFSPO), Tofft-Jørgensen, L. (Idrættens Analyseinstitut), Weber, A.Ch. (OFSPO), Weber, P.-A. (OFSPO)

Bibliographie

Antidoping Suisse (2011), Rapport annuel 2010; document consulté sur http://www.antidoping.ch/download/4074/fr/

Association Suisse de Football [ASF] (2011), *Rapport annuel de l'Association Suisse de Football* (ASF) 2010; Muri (BE): Ast & Fischer AG

Bake, D. (2013a, février), *Pillar 1;* présentation à l'occasion de l'atelier de comparaison internationale, Macolin

Bake, D. (2013b, février), *Pillar 6 & 8;* présentation à l'occasion de l'atelier de comparaison internationale, Macolin

Bâle-Ville (2013), *So funktioniert der Swisslos-Fonds;* document consulté sur http://www.swisslosfonds.bs.ch/swisslos-fonds.htm

Balthasar, A. (2009), *Die Versorgung der Schweiz mit Sportstätten – Auswertung und Interpretation einer Befragung der Schweizer Gemeinden;* Lucerne: Interface

Balthasar, A., Bieri, O., Laubereau, B., Arnold, T., Rütter, H., Höchli, C., Rieser, A., Stettler, J. & Wehrli, R. (2013), *Installations sportives en Suisse: statistiques 2012, Rapport de synthèse;* Macolin: Office fédéral du sport

Chappelet, J.-L. (2002), *From Lake Placid to Salt Lake City. The Incredible Growth of the Olympic Winter Games since 1980;* document consulté sur http://olympicstudies.uab.es/pdf/chapelet_extra.pdf

Comité International Olympique [CIO] (2012), *Résultats officiels des Jeux Olympiques;* document consulté sur http://www.olympic.org/resultats-olympiques

Comité International Olympique [CIO] (2013a), *Charte olympique. Etat en vigueur au 9 septembre 2013;* document consulté sur http://www.olympic.org/Documents/olympic_charter_fr.pdf

Comité International Olympique [CIO] (2013b), *Olympic Marketing Fact File,* 2013 Edition; document consulté sur http://www.olympic.org/Documents/IOC_Marketing/OLYMPIC_MARKETING_FACT_FILE.PDF

Commission pour la technologie et l'innovation [CTI] (2011), *Rapport annuel. Agence pour la promotion de l'innovation CTI 2010;* Berne: CTI Agence pour la promotion de l'innovation

Commission pour la technologie et l'innovation [CTI] (2012), *Bewilligte Sportprojekte 2010;* document non publié

Confédération suisse (2011), *Compte d'Etat 2010. Unités administratives. Chiffres;* document consulté sur http://www.efv.admin.ch/f/downloads/finanzberichterstattung/rechnung/2010/RG2A_2010-f.pdf

De Bosscher, V., Bingham, J., Shibli, S., van Bottenburg, M. & De Knop, P. (2008), *The Global Sporting Arms Race. An International Comparative Study on Sport Policy factors Leading to International Sporting Success;* Oxford: Meyer & Meyer Sport

De Bosscher, V., van Bottenburg, M., Shibli, S. & Westerbeek, H. (2013a), *Winning The Gold War. Policy Lessons from an International Comparative Study SPLISS,* présentation à l'occasion de la «4th International Conference on Sports and Exercise Science», à Bangkok

De Bosscher, V., van Bottenburg, M., Shibli, S. & Westerbeek, H. (2013b), *Pillar 2: Governance, Organisation and Structure of (Elite) Sport Policies. An Integrated Approach to Policy Development;* document non publié

De Bosscher, V., van Bottenburg, M., Shibli, S. & Westerbeek, H. (2013c), *Pillar 4: Talent Identification and Development;* document non publié

De Bosscher, V., van Bottenburg, M., Shibli, S. & Westerbeek, H. (2013d), *Pillar 5: Athletic Career and Post Career Support;* document non publié

Département fédéral de la défense, de la protection de la population et des sports [DDPS] (2011), *Secrétariat général, Finances du DDPS;* document non publié

Digel, H., Burk, V. & Fahrner, M. (2006), *Le sport de haut niveau. Une comparaison internationale;* Weilheim/Teck: Bräuer

Emrich, E. & Güllich, A. (2005), *Zur «Produktion» sportlichen Erfolges. Organisationsstrukturen, Förderbedingungen und Planungsannahmen in kritischer Analyse;* Cologne: Sport & Buch Strauss

Ericsson, K. A. (2005), Development of Elite Performance and Deliberate Practice. An Update from the Perspective of the Expert Performance Approach, in Starkes, J. L. & Ericsson, K. A. (Editors), *Expert Performance in Sports. Advances in Research on Sport Expertise* (p. 49-84); Champaign: Human Kinetics Pub Inc.

Fasse, M. & Höpner, A. (2010, 1er juin), Wie Deutsche Firmen von der WM in Brasilien profitieren, *Handelsblatt;* document consulté sur http://www.handelsblatt.com/unternehmen/industrie/milliardenprogramm-wie-deutsche-firmen-von-der-wm-in-brasilien-profitieren-seite-all/3450156-all.html

Fédération suisse de gymnastique [FSG] (2011), *Rapport de gestion 2010;* document consulté sur http://www.stv-fsg.ch/fileadmin/user_upload/stvfsgch/dokumente/verband/ueber_uns/downloads/geschaeftsbericht_turnjahr_2010.pdf

Fédération Suisse des Sports Equestres (2011), Budget 2012. *Bulletin. Elevage chevalin et sports équestres, 13,* p. 33-38

Fondation de l'Aide Sportive Suisse (2011), *Rapport annuel 2010;* document non publié

Fonds national suisse [FNS] (2011), *Statistiques 2010;* document consulté sur http://www.snf.ch/SiteCollectionDocuments/por_fac_sta_kurz_jb10_f.pdf

Gratton, Ch. & Preuss, H. (2008), Maximizing Olympic Impacts by Building Up Legacies, *The International Journal of the History of Sport*, 25(14); 1922-1938

Heinzmann, F. (2007), *Nachwuchsförderung im Sport. Ein Beitrag zur Analyse und Entwicklung der Nachwuchsförderung in der Schweiz aus systemtheoretischer Sicht* (Dissertation, Université de Zurich); Wiesbaden: Deutscher Universitäts-Verlag

Held, T. (2001), *Analyse Mittelfluss im Schweizer Sport;* Berne: Swiss Olympic Association

Hogan, K. & Norton, K. (2000), The 'Price' of Olympic Gold, *Journal of Science and Medicine in Sport* 3(2), 203-218

Houlihan, B. & Green, M. (2008), *Comparative Elite Sport Development. Systems, Structure and Public Policy,* Oxford: Elsevier

Institut pour l'étude de la neige et des avalanches [SLF] (non daté), *Le SLF;* document consulté sur http://www.slf.ch/ueber/index_FR

Kempf, H. (2004). *Sport: Zwischen Sturz und Euphorie. Eine vergleichende Analyse der institutionellen Vorkehrungen für den Sport als Spiel und jenem als Ware* (Dissertation, Université de Fribourg); Fribourg: Editions universitaires Fribourg

KPMG (2010), *Fédération Suisse des Sociétés d'Aviron, Sarnen, Bericht des Wirtschaftsprüfers zur Jahresrechnung an die Delegiertenversammlung, Comptes annuels 2009/2010;* rapport présenté à l'Assemblée des délégués en 2010, à Soleure

Lamprecht, M., Fischer A. & Stamm, H.P. (2011), *Clubs sportifs en Suisse. Etude sur le sport organisé;* Macolin: Office fédéral du sport OFSPO

Lämsä, J. (2013, février), *Funding of elite sport (Pillar 1);* présentation à l'occasion de l'atelier de comparaison internationale, Macolin

Ligue nationale suisse de hockey sur glace Sàrl (2011), *12e assemblée ordinaire des sociétaires de la Ligue Nationale Suisse de Hockey sur Glace Sàrl à Lucerne, Communiqué de presse du 9 juin 2011;* document consulté sur http://www.nationalleague.ch/media/native/pdf/nl/ueberuns/09-06-2011_ausgezeichneter_geschaeftsgang_der_national_league_franz.pdf

Loterie Romande (2011), *Rapport annuel. Bilan social 2010;* Lausanne: Société de la Loterie de la Suisse Romande

Müller, H., Rütter, H. & Stettler, J. (2010), *UEFA EURO 2008TM und Nachhaltigkeit – Erkenntnisse zu Auswirkungen und Einschätzungen in der Schweiz;* Berne: Forschungsinstitut für Freizeit und Tourismus

Office fédéral de la statistique [OFS] (2011), *Statistique financière 2009 de la Suisse;* document consulté sur http://www.bfs.admin.ch/bfs/portal/fr/index/themen/18/22/publ.html?publicationID=4580

Office fédéral de la statistique [OFS] (2012a), *Niveau de formation 1) de la population résidante selon l'âge et le sexe en 2011;* document consulté sur http://www.bfs.admin.ch/bfs/portal/fr/index/themen/15/17/blank/01.indicator.406101.4086.html

Office fédéral de la statistique [OFS] (2012b), *Financement de la R-D;* document consulté sur http://www.bfs.admin.ch/bfs/portal/fr/index/themen/15/09/key/ind2.indicator.20203.202.html

Office fédéral de la statistique [OFS] (2013), *Revenu professionnel brut par année (1) des personnes actives occupées selon la situation dans la profession, les groupes de professions CITP 88 (COM)(2), le taux d'occupation et le sexe;* document consulté sur http://www.bfs.admin.ch/bfs/portal/fr/index/themen/03/04/blank/key/erwerbseinkommen.html

Office fédéral du sport [OFSPO] (2011a), *Aufteilung Kosten und Erlöse BASPO 2010 auf den Spitzen- und Breitensport (per 31. 12. 2010);* document non publié

Office fédéral du sport [OFSPO] (2011b), *Forschungskonzept Sport und Bewegung 2008-11. Stand Aug. 2011,* projets mis au concours sur une base compétitive; document non publié

Office fédéral du sport [OFSPO] (2011c), *Konzept des Bundes für internationale Sportanlässe in der Schweiz. Funktion, Verfahren und Unterstützung;* document non publié

Office fédéral du sport [OFSPO] (2011d), *Aufwand des BASPO 2001–2010;* document non publié

Office fédéral du sport [OFSPO] (2011e), *Jeunesse+Sport, Moniteurs actifs;* document non publié

Office fédéral du sport [OFSPO] (2012), *Jeunesse+Sport, Groupe d'utilisateurs 7, Promotion des espoirs, Paiements pour les disciplines sportives;* document non publié

Office fédéral du sport [OFSPO] (non daté, a), *Jeunesse+Sport;* document consulté sur http://www.jugendundsport.ch

Office fédéral du sport [OFSPO] (non daté, b), *Conception des installations sportives d'importance nationale;* document consulté sur http://www.baspo.admin.ch/internet/baspo/fr/home/themen/politik/nasak.html

Office fédéral du sport [OFSPO] (non daté, c), *Formation des entraîneurs Suisse, Formation d'entraîneur diplômé (FED);* document consulté sur http://www.baspo.admin.ch/internet/baspo/fr/home/dienstleistungen/bildung/beruf/trainerbildung/0/ausbildung/diplomtrainerlehrgang.parsysrelated1.74790.downloadList.48318.DownloadFile.tmp/ausschreibungdtafed1416fr.pdf

Office fédéral du sport [OFSPO] (non daté, d), *Le centre national d'entraînement de Macolin;* document consulté sur http://www.baspo.admin.ch/internet/baspo/fr/home/themen/foerderung/spitzensport/nat__leistungszentrum.html

Preuss, H. (2007), The Conceptualisation and Measurement of Mega Sport Event Legacies; *Journal of Sport & Tourism,* 12(3-4), 207-227

Rütter, H., Höchli, C., Schmid, C., Beck, A., Holzhey, M. (2011), *Wirtschaftliche Bedeutung des Sports in der Schweiz* – 2008; étude commandée par l'Office fédéral du sport OFSPO, document consulté sur http://www.baspo.admin.ch/internet/baspo/de/home/aktuell/documents/2008.parsys.88324.downloadList.34723.DownloadFile.tmp/berichtsportwirtschaft2008231211.pdf

Rütter, H., Stettler, J. & Kempf, H. (2013), *Volkswirtschaftliche Bedeutung Olympische Winterspiele Graubünden 2022;* document consulté sur http://www.ruetter.ch/cm/de/aktuell/news/193-volkswirtschaftliche-bedeutung-der-olympischen-winterspiele-graubuenden-2022-ermittelt14

Schafer, J. (2011), *Erfolgsfaktoren der Nachwuchsförderung in Schweizer Sportverbänden* (dissertation, Université de Fribourg); document consulté sur http://ethesis.unifr.ch/theses/SchaferJ.pdf

Shibli, S., De Bosscher V., van Bottenburg, M. & Westerbeek, H. (2013, septembre), *Alternative Measures of Performance in the Olympic Games;* présentation à l'occasion du Congrès EASM à Istanbul

Société du Sport-Toto [SST] (2012), *Origine et distribution des fonds;* document consulté sur http://www.sport-toto.ch/10-1-Soutiens-Financiers.html

Sport+Markt (2011), *Sponsoring-Barometer Schweiz 2010/2011,* Cologne: Sport+Markt AG

SRG SSR (2011), *Rapport de gestion SRG SSR 2010. Comptes annuels SSR;* document consulté sur http://www.srgssr.ch/fileadmin/rg2010/pdf/SRG_SSR_RG_2010.pdf

Stamm, H.P. & Lamprecht, M. (2012), *Observatoire Sport et activité physique en Suisse, Erfolgsbilanz im internationalen Spitzensport;* document consulté sur http://www.sportobs.ch/ind3_100.html?&L=1

Stettler, J., Linder, Ph., Stofer, Ch., Erni Baumann, C. & Mehr R. (2008), *Wirtschaftliche Bedeutung der Sportveranstaltungen in der Schweiz;* Macolin: Office fédéral du sport OFSPO

Stettler, J., Kempf H., Rütter, H., Gnädinger J., Hausmann, C. & Herzer, C. (2011), *Analyse der Sporteventförderung der öffentlichen Hand;* Macolin: Office fédéral du sport OFSPO

Structure des pouvoirs publics UEFA EURO 2008, Office fédéral du sport [OFSPO] (2008), *Rapport final EURO 2008;* Macolin: Office fédéral du sport OFSPO

Swisslos (2011a), *Mittel an den nationalen Sport und die kantonalen Sportfonds, Jahre 2001-2010;* document non publié

Swisslos (2011b), *Rapport de gestion 2010, Faire le bien n'est pas dû au hasard;* document consulté sur https://www.swisslos.ch/media/fr/swisslos/dokumentation/publikationen/Swisslos_GB_2010_d.pdf

Swiss Athletics (2011), *Rapport annuel 2010;* document consulté sur https://www.swiss-athletics.ch/files/ueber_swiss_athletics/jahresberichte/fr_rapport_annuel_2010_web.pdf

Swiss Curling (2011), *Jahresbericht und Rechnung 2009/10. Budget 2010/11;* Berthoud: Steinhof PrintMedia AG

Swiss Football League [SFL] (2011), *Rapport annuel 2009/2010;* document consulté sur http://www.sfl.ch/uploads/media/Jahresbericht_SFL_2009-10.pdf

Swiss Ice Hockey Association [SIHA] (2010), *Geschäftsbericht. Saison 2009/2010;* document consulté sur http://www.swiss-icehockey.ch/media/native/pdf/siha/medien/siha_geschaeftsbericht_0910.pdf

Swiss Olympic (2008a), *Manuel «Détection et sélection des talents»;* Ittigen (BE): Swiss Olympic Association

Swiss Olympic (2008b), *Swiss Olympic Medical Center;* document consulté sur http://www.swissolympic.ch/fr/desktopdefault.aspx/tabid-3255/4417_read-25204/

Swiss Olympic (2009), *Directives concernant la remise de la Swiss Olympic Card et de la Swiss Olympic Talents Card* (version modifiée); Ittigen (BE): Swiss Olympic Association

Swiss Olympic (2010a), *«Concept du sport d'élite suisse», Continuité de la promotion relève-élite, Version: 01.05.2010;* document consulté sur http://www.swissolympic.ch/fr/Portaldata/41/Resources/03_sport/verbaende/spitzensport_konzept/Spitzensport-Konzept_Schweiz_2010_05_01_FR.pdf

Swiss Olympic (2010b), *SOMC-Bases;* document non publié

Swiss Olympic (2010c), *Swiss Olympic Team 2010;* document consulté sur http://www.swissolympic.ch/Portaldata/41/Resources/02_olympisches/winterspiele/vancouver_2010/selektionen/Liste_SwissOlympicTeam2010.doc

Swiss Olympic (2011a), *Rapport annuel, Exercice 2010/Budget 2012;* Berne: printgraphic AG

Swiss Olympic (2011b), *Directives de classification des spécialités sportives, valable à partir du 1er janvier 2012;* document consulté sur http://www.swissolympic.ch/Portaldata/41/Resources/03_sport/verbaende/einstufung_der_sportarten/Richtlinien_Einstufung_2012_Final_FR.pdf

Swiss Olympic (2011c), *Finanzierung der Sportarten durch Swiss Olympic;* document non publié

Swiss Olympic (2011d), *Programmes scolaires adaptés aux talents sportifs. Sport de performance et formation;* Ittigen (BE): Swiss Olympic

Swiss Olympic (2012a), *Compte rendu sur les «Swiss Olympic Talents Cards» 2005-2011;* document consulté sur http://www.swissolympic.ch/Portaldata/41/Resources/03_sport/athleten/swiss_olympic_card_alt/talents_cards/2012/Report_Talents_Cards_2011_f.pdf

Swiss Olympic (2012b), *Les 102 sportives et sportifs sélectionnés pour Londres 2012;* document consulté sur http://www.swissolympic.ch/Portaldata/41/Resources/02_olympisches/sommerspiele/london_2012/selektionen/Selektion_alle.pdf

Swiss Olympic (2013), *Concepts de promotion de la relève;* document consulté sur http://www.swissolympic.ch/fr/desktopdefault.aspx/tabid-3513/4382_read-45334/

Swiss-Ski (2011), *Rapport annuel 2010/11;* document consulté sur http://www.swiss-ski.ch/fileadmin/media/swissski/Documents_2012/201107_WEB_SwissSkiGB10_fr.pdf

Swiss Sport Management Center [SSMC] (2011), *Formation continue. L'offre en un coup d'œil;* document consulté sur http://www.ssmc.ch/pub/fr/weiterbildung_aufbau_fr.php

Swiss Tennis (2011), *Rapport de l'exercice 2010;* document consulté sur http://www.swisstennis.ch/upload/docs/uber_uns/Geschaftsbericht2010_f_Einzelseiten_low.pdf

SwissTopSport [STS] (2011), *Association. Histoire. SwissTopSport – un exemple de solidarité;* document consulté sur http://www.swisstopsport.ch/Geschichte.9.0.html

Tofft-Jørgensen, L. (2013, février), *Pillar 1;* présentation à l'occasion de l'atelier de comparaison internationale, Macolin

Weber, A. Ch., Kempf, H., Renaud, A. & De Bosscher V. (2013, novembre), *Development of Rivalry in Olympic Winter Sports;* présentation à l'occasion de la Conférence SPLISS à Anvers

Zahner, L. & Babst, H. (2003), *Les 12 éléments de la réussite. Concept de base de Swiss Olympic Association pour l'encouragement de la relève (3e édition);* Ittigen (BE): Swiss Olympic Association

Remerciements

Grâce au taux élevé de réponse aux enquêtes, nous avons pu disposer d'une source de données fiable: un grand merci aux athlètes, aux entraîneurs et aux chefs du sport d'élite qui ont participé.

Nous souhaitons remercier tout particulièrement les experts et le groupe d'accompagnement, à savoir MM. Adrian Bürgi, Pierre-André Weber (Office fédéral du sport), Cornell Hollenstein (Swiss Olympic) et Hanspeter Stamm (Lamprecht und Stamm, Sozialforschung und Beratung AG), pour les précieuses informations, suggestions et pistes d'amélioration qu'ils ont apportées et qui ont permis l'accomplissement de ce travail.

Nous tenons également à témoigner notre reconnaissance aux collaborateurs de la section Sport et société et du service Médiathèque et médias didactiques de la Haute école fédérale de sport de Macolin pour leur soutien actif. Sans leur énergie et leur implication, le présent rapport n'aurait en effet pas pu être terminé dans les délais.

Un produit de la Haute école fédérale de sport de Macolin HEFSM

Auteurs: Hippolyt Kempf, Andreas Ch. Weber, Anne Renaud, Marco Stopper

Traduction: CB Service, Lausanne

Photo: Patrick B. Krämer

Edition: 2014, 2e édition remaniée

Editeur: Office fédéral du sport OFSPO
Internet: www.ofspo.ch, www.hefsm.ch
Courriel: sportoekonomie@baspo.admin.ch

Tous droits réservés. Reproduction ou diffusion partielle ou intégrale interdite
sans l'accord écrit de l'éditeur et la mention de la source.
