# Supplementary material for: Reviewing the role of the environment in the talent development of a professional soccer club
Source: PLoS One. 2021 Feb 25;16(2):e0246823. doi: 10.1371/journal.pone.0246823 (PMC7906372; doi:10.1371/journal.pone.0246823)
Supplement: S1 Appendix — (DOCX) [file pone.0246823.s004.docx]

**S1. Appendix**

**The 5-Factor Talent Development Environment Questionnaire**

| **Item content** | |
| --- | --- |
| **1.** | **Je suis rarement encouragé à anticiper la façon avec laquelle je devrais réagir en cas de problème** |
|  | *I am rarely encouraged to plan for how I would deal with things that might go wrong* |
| **2.** | **Je passe la plupart de mon temps à développer des capacités que mon entraîneur dit être nécessaires pour réussir à performer au plus haut niveau** |
|  | *I spend most of my time developing skills and attributes that my coach tells me I will need if I am to compete successfully at the top/professional level* |
| **3.** | **Mon entraîneur explique comment mon entraînement et mon programme de compétition sont liés pour m'aider à progresser** |
|  | *My coach explains how my training and competition programme work together to help me develop* |
| **4.** | **Mon entraîneur parle régulièrement avec les autres personnes qui m’accompagnent dans mon projet sportif à propos des objectifs que j’essaie d'atteindre (par exemple : physiothérapeute, psychologue du sport, nutritionniste, préparateur physique, coach mental)** |
|  | *My coaches talk regularly to the other people who support me in my sport about what I am trying to achieve (e.g., physiotherapist, sport psychologist, nutritionist, strength and conditioning coach, lifestyle advisor)* |
| **5.** | **La marche à suivre concernant ce que je dois faire pour progresser dans mon sport n'est pas très claire** |
|  | *The guidelines in my sport regarding what I need to do to progress are not very clear* |
| **6.** | **Même s'il y avait un creux dans mes performances, on continuerait à m'offrir des opportunités pour me faire progresser** |
|  | *I would be given good opportunities even if I experienced a dip in performance* |
| **7.** | **On ne m’a pas suffisamment appris comment gérer l'équilibre entre les entraînements, les compétitions et la récupération** |
|  | *I am not taught that much about how to balance training, competing, and recovery* |
| **8.** | **Les conseils que mes parents me donnent correspondent bien aux conseils que je reçois de mon/mes entraîneur-s** |
|  | *The advice my parents give me fits well with the advice I get from my coaches* |
| **9.** | **Mon entraîneur et moi parlons régulièrement des choses dont j'ai besoin pour progresser vers le haut-niveau dans mon sport (par exemple : bonne habitude de travail, les performances en compétition, les aspects physiques, mentaux, techniques ou tactiques)** |
|  | *My coach and I regularly talk about things I need to do to progress to the top level in my sport (e.g., training ethos, competition performances, physically, mentally, technically, tactically)* |
| **10.** | **Les personnes qui m'accompagnent dans mon projet sportif semblent être tous sur la même longueur d'onde lorsqu'on aborde ce qui est le mieux pour moi (par exemple : entraîneurs, physiothérapeute, psychologue, préparateur physique, nutritionniste, coach mental)** |
|  | *Those who help me in my sport seem to be on the same wavelength as each other when it comes to what is best for me (e.g., coaches, physiotherapists, sport psychologists, strength trainers, nutritionists, lifestyle advisors)* |
| **11.** | **Mon entraîneur prend rarement le temps de parler avec les autres entraîneurs qui travaillent avec moi** |
|  | *My coach rarely takes the time to talk to other coaches who work with me* |
| **12.** | **Mon entraîneur ne semble pas très intéressé par ma vie en dehors du sport** |
|  | *My coach doesn’t appear to be that interested in my life outside of sports* |
| **13.** | **J'ai actuellement accès à différents professionnels pour m'accompagner dans ma progression sportive (par exemple : physiothérapeute, psychologue du sport, préparateur physique, nutritionniste, coach mental)** |
|  | Currently, I have access to a variety of different types of professionals to help my sports development (e.g., physiotherapist, sport psychologist, strength trainer, nutritionist, lifestyle advisor). |
| **14.** | **Si j’en ai besoin et à tout moment, je peux passer voir mon entraîneur ou un membre du staff (par exemple : physiothérapeute, psychologue, préparateur physique, nutritionniste, coach mental)** |
|  | I can pop in to see my coach or other support staff whenever I need to (e.g., physiotherapist, psychologist, strength trainer, nutritionist, lifestyle advisor). |
| **15.** | **Mon/Mes entraîneur-s prend/prennent le temps de parler de moi et des objectifs que j'essaie d'atteindre à mes parents** |
|  | My coaches make time to talk to my parents about me and what I am trying to achieve |
| **16.** | **Je ne reçois pas beaucoup d'aide pour améliorer ma force mentale dans mon sport** |
|  | I don’t get much help to develop my mental toughness in sport effectively |
| **17.** | **Mon entraîneur et moi essayons souvent d'identifier quel sera mon prochain challenge avant qu'il ne se présente** |
|  | My coach and I often try to identify what my next big test will be before it happens |
| **18.** | **Mon entraîneur me permet d'apprendre de mes erreurs** |
|  | My coach allows me to learn through making my own mistakes |
| **19.** | **Mon entraînement est spécifiquement pensé pour m'aider à progresser efficacement sur le long-terme** |
|  | My training is specifically designed to help me develop effectively in the long term |
| **20.** | **Mes progrès et mes performances sont régulièrement évalués en comparaison par rapport à mes propres références (i.e., mes performances passées, mon niveau actuel)** |
|  | My progress and personal performance are reviewed regularly on an individual basis |
| **21.** | **Mon entraîneur dit toujours que la qualité de ce qui est fait lors des entraînements et des compétitions est bien plus important que le résultat en lui-même** |
|  | My coach emphasises that what I do in training and competition is far more important than winning |
| **22.** | **Mon entraîneur et moi discutons de ce que les sportifs de classe mondiale actuels et/ou passés ont fait pour réussir** |
|  | My coach and I talk about what current and/or past world-class performers did to be successful |
| **23.** | **On m'implique dans la plupart des décisions concernant mon projet de progression sportive** |
|  | I am involved in most decisions about my sport development |
| **24.** | **Mon entraîneur évoque rarement la question de mon bien-être** |
|  | My coach rarely talks to me about my well-being |
| **25.** | **Je fixe régulièrement avec mon coach des objectifs qui sont spécifiques à ma progression individuelle** |
|  | I regularly set goals with my coach that are specific to my individual development |
